# Pesticide residue in commonly consumed vegetables in selected districts of Jimma Zone, Southwest Ethiopia

**DOI:** 10.1371/journal.pone.0343871

**Published:** 2026-03-02

**Authors:** Hawi Hussen Ahimed, Higemengist Astatkie, Seblework Mekonen, Fitsum Demissie, Samuel Fekadu

**Affiliations:** 1 Department of Environmental Health Science and Technology, Jimma University, Jimma, Ethiopia; 2 Department of Environmental Health, College of Medicine and Health Sciences, Bahir Dar University, Bahir Dar, Ethiopia; 3 Ethiopian Institute of Water Resources, Water and Health, Addis Ababa University, Addis Ababa, Ethiopia; 4 Armauer Hansen Research Institute, Traditional and Modern Medicine Research and Development, Ethiopia; Universitas Airlangga, INDONESIA

## Abstract

**Background:**

Pesticides are essential in agriculture for protecting crops from pests, diseases, and weeds, but improper use can lead to health issues like neurological disorders and carcinogenic effects. Strengthening regulatory frameworks, promoting integrated pest management strategies, and raising farmer awareness can mitigate pesticide misuse. In Ethiopia, widespread pesticide use in vegetables raises concerns about consumer exposure to pesticide residues. This study determined the pesticide residue in vegetables in Southwest Ethiopia.

**Methods:**

The study was conducted in randomly selected districts of the Jimma zone. Samples of tomatoes, potatoes, cabbage, and onions were collected from vegetable farmers. The modified QuEChERS (Quick, Easy, Cheap, Effective, Rugged, and Safe) methods were used for sample preparation and extraction. The analysis of pesticide residues was performed using gas chromatography coupled with a mass spectrometer (GC-MS) and an ion trap analyzer with an automatic injection. The pesticide detection levels among types of vegetables and locations were analyzed using one-way Analysis of variance (ANOVA) with statistical significance set at *p* < 0.05.

**Results:**

Pesticide residues were detected in vegetables from Seka Chokorsa, Goma, and Dedo districts. Detected pesticides include Lindane, Aldrin, Chlorpyrifos, 4,4-DDE, Hexachlorobenzene, and Endosulfan II. The highest concentrations were found in the Dabo Gibe onion sample (Seka Chokorsa), the Waro Kolobo potato sample (Dedo), and the Ganji Dalacho cabbage sample (Goma). Lindane residues were found in onion and potato (Seka Chokorsa), exceeding the Maximum Residue Limit (MRL) of 10–50 µg/kg. Chlorpyrifos residues were detected below MRLs across all districts, while 4,4-DDE residues were also detected in some vegetables, indicating historical use of banned pesticides. ANOVA results showed small variation between groups, and there was no statistically significant difference across the four groups (p-value = 0.305). The study highlights the need for stricter regulation, farmer education, and residue monitoring to ensure food safety.

## Introduction

Pesticides are crucial for crop protection from pests, diseases, and weeds, thereby enhancing productivity and ensuring food security [1]. The use of pesticides has significantly contributed to increased crop yields by minimizing losses caused by pests and diseases, which, in turn, supports economic stability for farmers [2]. In developing countries like Ethiopia, pesticides are extensively used to boost vegetable production [3]. However, improper use of pesticides can cause acute and chronic health problems and environmental contamination.

Studies indicate that excessive pesticide residues in food can lead to acute and chronic health issues, including neurological disorders, endocrine disruption, and even carcinogenic effects [4]. Farmers and agricultural workers are also at high risk due to direct exposure, which may result in poisoning and long-term health complications [5]. Moreover, pesticide runoff and leaching contribute to environmental degradation by contaminating soil, water bodies, and non-target organisms, disrupting ecosystems and biodiversity [6].

In Ethiopia, the widespread use of pesticides in vegetable farming is raising concerns about the potential pesticide residues in consumed vegetables. Pesticide application practices among Ethiopian farmers often lack proper knowledge of dosage, application frequency, and safety precautions, increasing the risk of excessive residues in food [7]. Given the increasing reliance on pesticides, it is essential to conduct regular assessments of pesticide residue levels in vegetables to ensure food safety and protect public health. Strengthening regulatory frameworks, promoting integrated pest management (IPM) strategies, and increasing awareness among farmers can help mitigate the adverse effects of pesticide misuse while maintaining agricultural productivity.

Southwest Ethiopia, known for its fertile lands and favorable climate, is a major producer of various vegetables [8]. Despite this, there is a lack of comprehensive monitoring systems to regulate pesticide use and ensure compliance with international safety standards [9]. The absence of robust monitoring and enforcement mechanisms increases the likelihood of pesticide residues exceeding safe limits [10], posing potential health hazards to consumers. Therefore, this study aimed to determine the level of pesticide residues in commonly consumed vegetables in the selected districts of the Jimma zone in the Oromia region of Ethiopia. By examining the types and levels of pesticide residues, this research provides insights into the potential health risks associated with consuming these vegetables. The findings of this study will contribute to developing strategies for improving pesticide management practices and enhancing food safety in the region.

## Methods and materials

### Study area

The study was conducted in the Jimma zone of the Oromia region in southwestern Ethiopia, focusing on Gomma, Dedo, and Seka Chokorsa districts. The area has a population of 3.4 million and a total area of 15,569 km^2^, has a low drought risk rating [11], and is home to 85% of the inhabitants who rely on subsistence farming [12]. The study focuses on smallholder farmers who grow cash crops like coffee, tea, fruits, and vegetables [13], which are the most preferred due to their high productivity and quick production cycle, which reduces unemployment [14].

### Study design and period

The study was a cross-sectional laboratory-based study, and the samples were collected during the rainy season in August 2024. It’s important to note that pesticide contamination is significantly greater during the wet season than in the dry season [15]. The contamination may be attributed to pesticides from different sources that can be washed into existing areas, resulting in higher contamination levels.

### Source and study population

The study involved three districts of the Jimma zone in the Oromia region: Dedo, Seka Chokorsa, and Gomma. From each district, three kebeles were selected, and vegetables were collected from farms for their respective vegetable production areas.

### Sampling and sampling technique

A total of 36 vegetable samples were collected (9 each of onion, tomato, potato, and cabbage). During sample collection, a random sample of the vegetable farmers was taken by a zigzag pattern in purposively selected districts. These are Dedo, Goma, and Seka chokers. According to the agricultural office of each district, the highest production of vegetables in three kebeles was selected. Each one kg sample was sealed, labeled, and placed in a clean paper envelope in polyethylene plastic bags. Then the samples were transported to the Laboratory of Adama Science and Technology University and frozen at negative 10 degrees Celsius until analysis. The sample handling was carried out according to the Food and Agriculture Organization (FAO) and the World Health Organization (WHO) guidelines [16]. The study included only vegetable samples from the Jimma district, after informed consent from farmers and their willingness to participate in research during sample collection and field surveys. The vegetables were not fresh; the study excluded those not cultivated in the study area.

### Reagents and Materials

All chemicals were reagents or pesticide-grade. Standards included α-Lindane, 4,4’-DDE (dichlorodiphenyldichloroethylene), endosulfan II, 4,4’-DDD (dichlorodiphenyldichloroethane), 4,4’-DDT (dichlorodiphenyltrichloroethane), and chlorpyrifos, aldrin, HCB, and deltamethrin. Chemicals used in all tests were obtained from Sigma-Aldrich (Germany). QuEChERS kits and dispersive solid-phase extraction (d-SPE) materials (Agilent SampliQ) were used for extraction and clean-up.

### Sample extraction and clean-up

Modified QuEChERS methods [17–19], was used for sample preparation and extraction. Pesticide residues in vegetable samples were extracted using a modified QuEChERS (Quick, Easy, Cheap, Effective, Rugged, and Safe) method. The modification primarily involved adjustments to the extraction and clean‑up procedures to enhance extract purity and compatibility with gas chromatographic analysis. Following the initial acetonitrile‑based extraction and dispersive solid‑phase clean‑up, the resulting extract was subjected to solvent evaporation under controlled conditions. The dried residue was subsequently reconstituted in a mixture of n‑hexane and acetone (9:1, v/v) before instrumental analysis. This additional solvent exchange step improved the removal of coextracted matrix components and enhanced analyte compatibility with the gas chromatography system, thereby improving chromatographic performance and detection efficiency.

The modified procedure proved suitable for the determination of pesticide residues in vegetable matrices. These methods are recommended for matrices with 75% water content [20], such as onion, tomato, potato, and cabbage, with respective water contents of 89% [21], 95% [22], 79% [23], and 76% [24]. Onion, tomato, potato, and cabbage were selected as representative matrices for the validation experiments. Each commodity was spiked with pesticide solutions at concentrations of 5, 10, 20, 30, 40, 50, and 100 μg/kg, with three replicates prepared for each level. In every analytical batch, a matrix blank, a method blank, and a solvent blank were also included for quality control.

For sample preparation, 10 g of finely homogenized material was weighed into a 50 mL centrifuge tube. Samples were fortified with pesticide solutions to obtain concentrations of 10, 20, 30, 40, 50, and 100 μg/kg and allowed to stand for 20 minutes to facilitate interaction between the pesticide and the matrix. Subsequently, 10 mL of acetonitrile was added, and the mixture was vortexed for 1 minute. A salt mixture containing 4 g Magnesium sulfate (MgSO₄), 1 g NaCl, 1 g trisodium citrate dihydrate, and 0.5 g disodium hydrogen citrate was then introduced, followed by immediate vortexing for 1 minute and centrifugation at 3,000 rpm for 5 minutes. From the supernatant, 6 mL of extract was transferred into a 15 mL centrifuge tube preloaded with 150 mg Primary-Secondary Amine (PSA) and 900 mg MgSO₄. The tube was sealed, vigorously shaken, vortexed for 30 seconds, and centrifuged again for 5 minutes at 3,000 rpm. A 4 mL portion of the cleaned extract was then placed in a round-bottom flask, and the pH was rapidly adjusted to 5 by adding 40 mL of a 5% formic acid solution in acetonitrile. The solution was evaporated to near dryness using a rotary evaporator under reduced pressure with the water bath maintained below 40°C. Finally, the residue was reconstituted with 1 mL of ethyl acetate and transferred into a 1.5 mL vial for quantification using GC-MS.

### Sample analysis

The analysis of pesticide residues was conducted using an integrated gas chromatography system and a mass spectrometry system [S2 File]. The separation of pesticides was achieved with a 30-meter length, 0.25 mm internal diameter, and 0.25 µm film thickness coated with a 5% phenyl-95% methylpolysiloxane Varian VF-5MS column. The retention times of the peaks in the sample chromatogram were compared to standard curves [25]. The sample equivalent (µg/kg) extract was calculated using the formula


$$Y=\left(\frac{a}{b}\right)X\left(\frac{x}{z}\right)$$ (1)

where Y = µg of pesticide per kg of vegetable, a = sample mass, b = solvent volume added, x = cleaned extract volume, and z = amount of hexane added for solvent exchange.

### Method validation

The study followed standard validation, which involved the analysis of blank vegetables spiked at multiple concentrations (10–100 µg/kg) in triplicate. The LOD and LOQ were determined using a signal-to-noise ratio (3:1 and 10:1, respectively) [17]. Precision was assessed by calculating the relative standard deviation of the lowest concentration capable of demonstrating linearity in a blank vegetable sample. Linearity was assessed by analyzing a mixture of pesticide standards at 10, 20, 30, 40, 50, and 100 μg/kg [17]. Routine quality control was carried out by including heptachlor as an internal standard in each analytical sample and calculating the percentage recovery [18].

### Data analysis

The study responses were statistically analyzed using MS Excel and SPSS version 20 [SPSS dataset.sav]. Pesticide residue concentrations were compared against the maximum residue limits (MRLs) established by the European Union [19]. Analysis of variance (ANOVA) was applied to evaluate differences in residue levels across vegetable varieties, with statistical significance set at *p* < 0.05. The validity of ANOVA was ensured by considering its key assumptions: independence of observations, normality, and homogeneity of variances. Where ANOVA showed significant differences, Tukey’s HSD (honestly significant difference) post-hoc test was applied to identify specific group differences.

### Ethical Consideration

The study’s ethical protocol was approved by Jimma University’s Institutional Review Board (IRB), and all records were kept anonymous and secured in data handling and analysis processes.

## Results

### Method validation results

The method validation result demonstrated strong reliability for the quantification of eight pesticides, with calibration curve linearity excellent for all compounds. Seven calibration levels were used, ensuring a robust model. The accuracy, expressed as recovery rates, was generally acceptable across the working range, with most compounds falling within the typical 70–120% window for pesticide residue analysis. However, notable issues appeared at the lower limit of quantification (LLOQ, 5 ppb), with Aldrine and Chlorpyrifos showing slightly high recoveries, while Endosulfan II displayed low recovery. Specific anomalies were observed for 4,4-DDE and 4,4-DDT, indicating possible matrix effects, interferences, or stability problems. The estimated Limit of Detection (LOD) is around 1–2 ppb, while the Limit of Quantification (LOQ) is estimated at approximately 3–5 ppb, corresponding to the lowest calibration point used for quantification. This level of sensitivity is adequate for regulatory monitoring, but the variable accuracy at the lowest levels highlights the need for a more rigorous experimental determination of LOD and LOQ.

The method demonstrated good specificity, with retention times for all analytes matching those of the calibration standards and ion ratio confirmation from multiple monitored ions supporting correct peak identification. However, occasional deviations in ion ratios or “Not Found” annotations suggested minor matrix interferences, which were correctly flagged and managed by the software, minimizing the risk of false positives [S1]. The analytical method is fit for purpose, providing strong linearity and generally acceptable accuracy for the quantification of tested pesticides. Improvements are needed at the lower range, where matrix effects and outliers impact performance [Table 1].

**Table 1:** Summary of method validation parameters for pesticide residue analysis of vegetable samples in selected districts of Jimma Zone, Ethiopia, 2024.

Pesticide	LOD (µg/Kg)	LOQ (µg/Kg)	Linearity (R^2^)	Range (µg/Kg)	Recovery (%)	Precision (%RSD)
*Lindane*	1.2	3.5	0.999	5-100	95.2-107.0	3.2-7.1
*Hexachlorobenzene*	1.0	3.0	0.999	5-100	95.0-104.9	2.8-6.5
*Aldrine*	1.5	4.0	0.999	5-100	93.4-120.7	4.1-8.3
*Chlorpyrifos*	1.8	5.0	0.999	5-100	92.0-124.9	3.5-9.2
*Endosulfan II*	1.3	4.2	0.999	5-100	77.9-107.3	5.0-10.1
*4,4-DDE*	1.6	4.5	0.998	5-100	91.5-125.3	4.5-9.8
*4,4-DDD*	1.4	4.3	0.998	5-100	93.8-118.5	3.9-8.7
*4,4-DDT*	2.0	5.5	0.999	10-100	67.7-104.2	6.2-12.4

### Pesticide residues

The study analyzed vegetable samples collected from selected woredas in the Jimma Zone, including Dedo, Goma, and Seka Chokorsa. Four commonly consumed vegetables were selected for analysis. The investigation targeted pesticide residues, including α-Lindane, 4,4’-DDE, Endosulfan II, Dichlorodiphenyldichloroethane (DDD), 4,4’-DDT, chlorpyrifos, aldrin, hexachlorobenzene (HCB), and deltamethrin.

The results showed that Lindane was detected in onion samples from Seka Chokorsa (Ushan Kache) at 1831.94 µg/kg and in potato samples from Dabo Gibe at 162.08 µg/kg. Aldrin was found in a potato sample from Goma (Chemi Chago) at 4.36 µg/kg. Chlorpyrifos was detected in several samples: cabbage from Goma (Jimate Daru) at 8.99 µg/kg, tomato from Dedo (Ofkole Kebele) at 6.97 µg/kg, tomato from Seka Chokorsa (Dabo Gibe) at 8.19 µg/kg, and potato from Goma (Chemi Chago) at 6.87 µg/kg.

The detection levels of 4,4’-DDE varied across districts. Onion samples from Dedo (Waro Kolobo), Goma (Ganji Dalacho), and Seka Chokorsa (Ushan Kache) showed 1.69 µg/kg, 1.57 µg/kg, and 1.67 µg/kg, respectively. Additionally, potatoes from Goma (Chemi Chago) contained 2.17 µg/kg, while tomatoes from Dedo (Waro Kolobo) contained 1.64 µg/kg.

Hexachlorobenzene (HCB) was also detected, with levels of 3.44 µg/kg in potato from Goma (Chemi Chago) and 2.16 µg/kg in tomato from Dedo (Waro Kolobo). Endosulfan II was found in onion from Dedo (Ofkole) at 29.21 µg/kg and in cabbage from Goma (Jimate Daru) at 6.49 µg/kg.

The study revealed that Endosulfan II residues were detected in the majority of samples collected from different kebeles of the Jimma Zone, specifically in the districts of Goma, Seka Chokorsa, and Dedo. The concentration of residues varied considerably across locations and crop types. Notably, onion from Dabo Gibe contained 40.88 µg/kg, potato from Waro Kolobo 34.56 µg/kg, potato from Ofkole 2.02 µg/kg, and potato from Ushan Kache 32.06 µg/kg. Cabbage samples showed concentrations of 8.79 µg/kg (Ganji Dalacho), 6.22 µg/kg (Jimate Daru), 8.44 µg/kg (Ofkole), 29.45 µg/kg (Dabo Gibe), and 69.92 µg/kg (Ganji Dalacho). Tomato samples exhibited 11.68 µg/kg (Waro Kolobo) and 14.77 µg/kg (Dabo Gibe). These findings indicate widespread contamination with Endosulfan II across multiple vegetable types and locations, with the highest concentration observed in cabbage from Ganji Dalacho (69.92 µg/kg). All samples were analysed in triplicate, and standard deviations are provided in Table 2 [Table 2].

**Table 2:** Pesticide residue in samples of vegetables in selected districts of Jimma Zone, Ethiopia*, 2024.*

Pesticide residues	District	Site/Location	Vegetable	Detected (µg/kg)	Standard deviation (µg/kg)	MRL (µg/kg)
Lindane (Lindane (γ-HCH))	Seka Chokorsa	Ushan Kache	Onion	1831.94	45.2	10–50**
	Seka Chokorsa	Dabo Gibe	Potato	162.08	12.6	10–50**
Aldrin	Goma	Chemi Chago	Potato	4.36	0.21	50-100
Chlorpyrifos	Goma	Jimte Daru	Cabbage	8.995	0.34	50–100
	Dedo	Ofkole Kebele	Tomato	6.976	0.28	50–100
	Seka Chokorsa	Dabo Gibe	Tomato	8.193	0.31	50–100
	Goma	Chemi Chago	Potato	6.8732	0.29	50–100
4,4-DDE	Dedo	Waro Kolobo	Onion	1.691	0.08	50
	Goma	Ganji Dalacho	Onion	1.569	0.07	50
	Seka Chokorsa	Ushan Kache	Onion	1.6783	0.09	50
	Goma	Chemi Chago	Potato	2.1735	0.11	50
	Dedo	Waro Kolobo	Tomato	1.6467	0.08	50
Hexachlorobenzene	Goma	Chemi Chago	Potato	3.4423	0.14	10–20
	Dedo	Waro Kolobo	Tomato	2.163	0.10	10–20
Endosulfan II	Dedo	Ofkole	Onion	29.21	1.20	50–100
	Goma	Jimte Daru	Cabbage	6.496	0.25	50–100
	Seka Chokorsa	Dabo Gibe	Onion	40.8853	1.50	50–100
	Dedo	Waro Kolobo	Potato	34.5661	1.30	50–100
	Dedo	Ofkole	Potato	2.0207	0.09	50–100
	Seka Chokorsa	Ushan Kache	Potato	32.0586	1.20	50–100
	Goma	Ganji Dalacho	Cabbage	8.7871	0.35	50–100
	Dedo	Waro Kolobo	Tomato	11.6826	0.45	50–100
	Dedo	Ofkole	Tomato	9.1162	0.38	50–100
	Goma	Jimte Daru	Cabbage	6.2162	0.24	50–100
	Seka Chokorsa	Dabo Gibe	Tomato	14.767	0.58	50–100
	Dedo	Ofkole	Cabbage	8.4452	0.33	50–100
	Goma	Ganji Dalacho	Potato	16.611	0.65	50–100
	Seka Chokorsa	Dabo Gibe	Cabbage	29.4546	1.10	50–100
	Goma	Ganji Dalacho	Cabbage	69.9216	2.80	50–100**

**Key**: **: *Above Limit,* ***1 ppm*** *= 1,000 µg/kg,* ***MRLs****: maximum residue limits* [20].

A one-way ANOVA was used to compare pesticide detection levels among four sample groups. The results showed that the variation between groups was relatively small compared to within groups. The F-ratio of 1.273, corresponding to a p-value of 0.305, was greater than the conventional significance threshold of 0.05. This suggests that the observed differences in pesticide levels could have arisen by chance, and there is no statistically significant difference across the four groups. The variability in pesticide levels is primarily due to differences within each group (i.e., variation among replicate samples of the same vegetable type and district), not systematic differences between groups. Tukey’s HSD post-hoc test confirmed no pairwise significant differences [Table 3].

**Table 3:** Analysis of variance (ANOVA) results of differences in residue levels across vegetable varieties in selected districts of Jimma Zone, Ethiopia.

Source of Variation	Sum of Squares	df	Mean Square	F	Sig.
Between Groups	424,293.9	3	141,431.3	1.273	0.305
Within Groups	2,777,016.2	25	111,080.6	–	–
Total	3,201,310.2	28	–	–	–

## Discussion

The study examines pesticide residues in vegetables from Seka Chokorsa, Goma, and Dedo districts, focusing on onion, potato, tomato, and cabbage. Common pesticides detected include Lindane, Aldrin, Chlorpyrifos, 4,4-DDE, Hexachlorobenzene, and Endosulfan II. The residue levels are compared against Maximum Residue Limits to assess potential health risks.

The study found *endosulfan II* pesticide residues were detected in Dabo Gibe onion, Waro Kolobo potato, and Ganji Dalacho cabbage. The highest concentrations were found in the Seka Chokorsa, Waro Kolobo, and Goma districts. Endosulfan II residues were detected in all types of vegetables across multiple sites, with concentrations ranging from 2.0207 to 69.9216 µg/kg. Most samples were within the MRL range of 50–100 µg/kg, except for one cabbage sample from Goma (69.9216 µg/kg), which slightly exceeds the upper MRL limit. This study’s finding shows that the presence of *endosulfan II* pesticide residues in vegetables aligns with findings from other African regions, like Kaduna State, Nigeria [21], Ibadan, Nigeria [22], and Central/Eastern Ethiopia [23]. The relatively high frequency and concentration of endosulfan II residues suggest widespread use and possible misuse, warranting stricter regulation and farmer education.

*Lindane* residues were found in onion and potato from Seka Chokorsa, exceeding the MRL range of 10–50 µg/kg. This level of detection has been reported from similar studies from Ethiopia [3] and Nigeria [24]. These high concentrations are concerning due to Lindane’s toxicity and potential bioaccumulation. The detected concentrations suggest recent or excessive application, poor adherence to pre-harvest intervals, or possible illegal use, given its banned or restricted status in many countries.

Aldrin was detected in potatoes from Goma (4.36 µg/kg), just below the MRL of 5 µg/kg. While this result does not exceed regulatory limits, Aldrin is another persistent organic pollutant with known adverse health effects, including neurotoxicity and potential carcinogenicity. Its presence, even at levels below the MRL, suggests ongoing use or environmental persistence, which is problematic due to its long-term environmental and health implications.

Chlorpyrifos residues were found in cabbage, tomato, and potato across all districts, with concentrations ranging from 6.8732 to 8.995 µg/kg. These values are well below the MRL range of 50–100 µg/kg, indicating compliance with safety standards. Regular monitoring remains crucial, as cumulative exposure from multiple food sources can still pose health risks. 4,4-DDE, a breakdown product of DDT, was detected in all sampled vegetables at levels between 1.569 and 2.1735 µg/kg, significantly below the MRL of 50 µg/kg. The presence of 4,4-DDE indicates historical use of DDT, as it is a persistent environmental contaminant. While current levels are low, their detection underscores the long-lasting impact of banned pesticides and the need for ongoing surveillance. Hexachlorobenzene was detected in potato and tomato samples, with concentrations of 3.4423 and 2.163 µg/kg, respectively, both below the MRL range of 10–20 µg/kg. Although the current residues are within safe limits, hexachlorobenzene is a persistent organic pollutant with carcinogenic and endocrine-disrupting properties. Its detection, even at low levels, serves as a reminder of the chemical’s environmental persistence.

Detected residues of persistent organochlorine pesticides reveal a concerning history of potentially illegal usage, particularly evidenced by significantly elevated levels of Lindane that exceed Maximum Residue Limits (MRLs), which pose health risks. The ongoing environmental persistence of Endosulfan II across multiple locations raises additional concerns. These findings correlate with recent research into pesticide residues in developing regions [25,26], underscoring the need for improved regulatory enforcement and intervention strategies.

High levels of Lindane specifically indicate a substantial public health risk, necessitating immediate action. Furthermore, the presence of banned or heavily restricted pesticides indicates regulatory non-compliance and a higher likelihood of illegal use. To mitigate these issues, it is crucial to enhance enforcement mechanisms, educate farmers on the safe application of pesticides, advocate for integrated pest management (IPM) techniques, and implement regular monitoring of pesticide residues to ensure public health is safeguarded. Comparative analysis of pesticide levels found no statistically significant differences among samples, suggesting that exposure levels were relatively uniform across the populations studied.

## Conclusions

In conclusion, this study identified various pesticide residues in vegetable samples from selected districts of the Jimma Zone. Endosulfan II was the most frequently detected, with some concentrations exceeding Maximum Residue Limits (MRLs), while high lindane levels pose significant public health risks, even though most of the analyzed pesticides were generally within MRLs. The presence of banned or heavily restricted pesticides highlights potential regulatory non-compliance, emphasizing the importance of stricter enforcement, education on safe pesticide practices, and promotion of integrated pest management. Comparative analysis showed no significant differences in pesticide levels across districts, indicating relatively uniform exposure. These findings suggest the need for continuous monitoring, strengthened regulations, and public awareness to ensure food safety, protect public health, and reduce environmental contamination. Future studies should expand geographically and assess dietary exposure and seasonal variation.

## Supporting information

S1 File SPSS data set. (SAV)

S2 File Supportive document. (PDF)
