# Supplementary material for: Pesticide residue in commonly consumed vegetables in selected districts of Jimma Zone, Southwest Ethiopia
Source: PLoS One. 2026 Mar 2;21(3):e0343871. doi: 10.1371/journal.pone.0343871 (PMC12952595; doi:10.1371/journal.pone.0343871)

Quantitative Analysis Complete Report

**Batch Path**	C:\Users\USER\OneDrive\Desktop\JU_Pesticide\MA\MA\QuantResults\Mohammed_Pest.batch.bin		
**Analysis Time**	13 Dec 2024 11:34	**Analyst Name**	DESKTOP-MRRPPC4\USER
**Report Time**	13 Dec 2024 11:37:19	**Reporter Name**	DESKTOP-MRRPPC4\USER
**Last Calib Update**	13 Dec 2024 11:34	**Batch State**	Processed
**Quant Batch Version**	10.0	**Quant Report Version**	10.0

Data File	Sample Name	Sample Type	Position	Inj Vol	Level	Acq. Method
10 ppb.D	Pest	Cal	1	1	2	pesticide std 12.11.2024
20 ppb.D	Pest	Cal	2	1	3	pesticide std 12.11.2024
40 ppb.D	Pest	Cal	4	1	5	pesticide std 12.11.2024
50 ppb.D	Pest	Cal	5	1	6	pesticide std 12.11.2024
100 ppb.D	Pest	Cal	6	1	7	pesticide std 12.11.2024
30-1ppb.D	Pest	Cal	3	1	4	pesticide std 12.11.2024
S2 DWO.D	Pest	Sample	7	1		pesticide std 12.11.2024
S1 GGDO.D	Pest	Sample	8	1		pesticide std 12.11.2024
S2 SUO.D	Pest	Sample	9	1		pesticide std 12.11.2024
S1 DOO.D	Pest	Sample	10	1		pesticide std 12.11.2024
S2 GJJ.D	Pest	Sample	11	1		pesticide std 12.11.2024
S1 SDO.D	Pest	Sample	12	1		pesticide std 12.11.2024
S1 SDP.D	Pest	Sample	13	1		pesticide std 12.11.2024
S2 DWP.D	Pest	Sample	14	1		pesticide std 12.11.2024
S1 DOP.D	Pest	Sample	15	1		pesticide std 12.11.2024
S2 SUP.D	Pest	Sample	16	1		pesticide std 12.11.2024
S1 DOP-1.D	Pest	Sample	15	1		pesticide std 12.11.2024
S1 GGC.D	Pest	Sample	1	1		pesticide std 12.11.2024
S1 GGP.D	Pest	Sample	2	1		pesticide std 12.11.2024
S1 DWT.D	Pest	Sample	3	1		pesticide std 12.11.2024
S1 DOT.D	Pest	Sample	4	1		pesticide std 12.11.2024
S2 GJC.D	Pest	Sample	5	1		pesticide std 12.11.2024
S1 SDT.D	Pest	Sample	6	1		pesticide std 12.11.2024
S1 DDC.D	Pest	Sample	7	1		pesticide std 12.11.2024
S1 DOC.D	Pest	Sample	8	1		pesticide std 12.11.2024
S2 GGP.D	Pest	Sample	9	1		pesticide std 12.11.2024
S1 SDC.D	Pest	Sample	10	1		pesticide std 12.11.2024
S1 GGC-1.D	Pest	Sample	1	1		pesticide std 12.11.2024
5 PPb.D	Pest	Cal	11	1	1	pesticide std 12.11.2024
S1 DDO S.D	Pest	Sample	12	1		pesticide std 12.11.2024
S1 DOC S1.D	Pest	Sample	2	1		pesticide std 12.11.2024
S1 DDP S.D	Pest	Sample	3	1		pesticide std 12.11.2024
S1 DWT S.D	Pest	Sample	4	1		pesticide std 12.11.2024

Quantitation Results

Agilent Trusted Answers

Lindane

Data File	Compound	Sample Type	RT	Resp.	Final Conc	Exp. Conc	Accuracy
10 ppb.D	Lindane	Calibration	4,987	6	10,5719	10,0000	105,7
20 ppb.D	Lindane	Calibration	4,982	12	19,0400	20,0000	95,2
40 ppb.D	Lindane	Calibration	4,982	27	41,4877	40,0000	103,7
50 ppb.D	Lindane	Calibration	4,976	32	48,7511	50,0000	97,5
100 ppb.D	Lindane	Calibration	4,976	67	100,2960	100,0000	100,3
30-1ppb.D	Lindane	Calibration	4,976	19	29,5022	30,0000	98,3
S2 DWO.D	Lindane	Sample	5,159	0	ND		
S1 GGDO.D	Lindane	Sample	5,164	0	ND		
S2 SUO.D	Lindane	Sample	4,955	1241	1831,9407		
S1 DOO.D	Lindane	Sample	5,159	0	ND		
S2 GJJ.D	Lindane	Sample	5,578	0	ND		
S1 SDO.D	Lindane	Sample	5,159	0	ND		
S1 SDP.D	Lindane	Sample	4,955	109	162,0754		
S2 DWP.D	Lindane	Sample	5,159	0	ND		
S1 DOP.D	Lindane	Sample	5,159	0	ND		
S2 SUP.D	Lindane	Sample	5,159	0	ND		
S1 DOP-1.D	Lindane	Sample	5,154	0	ND		
S1 GGC.D	Lindane	Sample	5,159	0	ND		
S1 GGP.D	Lindane	Sample	5,154	0	ND		
S1 DWT.D	Lindane	Sample	5,154	0	ND		
S1 DOT.D	Lindane	Sample	5,153	0	ND		
S2 GJC.D	Lindane	Sample	5,153	0	ND		
S1 SDT.D	Lindane	Sample	5,154	0	ND		
S1 DDC.D	Lindane	Sample	5,153	0	ND		
S1 DOC.D	Lindane	Sample	5,154	0	ND		
S2 GGP.D	Lindane	Sample	5,154	0	ND		
S1 SDC.D	Lindane	Sample	5,153	0	ND		
S1 GGC-1.D	Lindane	Sample	5,153	0	ND		
5 PPb.D	Lindane	Calibration	4,966	2	5,3511	5,0000	107,0
S1 DDO S.D	Lindane	Sample	5,154	0	ND		
S1 DOC S1.D	Lindane	Sample	5,148	0	ND		
S1 DDP S.D	Lindane	Sample	5,148	0	ND		
S1 DWT S.D	Lindane	Sample	5,148	0	ND		

Hexachlorobenzene

Data File	Compound	Sample Type	RT	Resp.	Final Conc	Exp. Conc	Accuracy
10 ppb.D	Hexachlorobenzene	Calibration	7,444	102	9,4986	10,0000	95,0
20 ppb.D	Hexachlorobenzene	Calibration	7,434	232	19,2419	20,0000	96,2
40 ppb.D	Hexachlorobenzene	Calibration	7,428	518	40,6516	40,0000	101,6
50 ppb.D	Hexachlorobenzene	Calibration	7,428	669	51,9929	50,0000	104,0
100 ppb.D	Hexachlorobenzene	Calibration	7,428	1298	99,1728	100,0000	99,2

Agilent Trusted Answers

Hexachlorobenzene

Data File	Compound	Sample Type	RT	Resp.	Final Conc	Exp. Conc	Accuracy
30-1ppb.D	Hexachlorobenzene	Calibration	7,428	365	29,1991	30,0000	97,3
S2 DWO.D	Hexachlorobenzene	Sample	7,342	0	ND		
S1 GGDO.D	Hexachlorobenzene	Sample	7,003	0	ND		
S2 SUO.D	Hexachlorobenzene	Sample	6,443	0	ND		
S1 DOO.D	Hexachlorobenzene	Sample	6,976	0	ND		
S2 GJJ.D	Hexachlorobenzene	Sample	7,740	0	ND		
S1 SDO.D	Hexachlorobenzene	Sample	7,078	0	ND		
S1 SDP.D	Hexachlorobenzene	Sample	6,438	0	ND		
S2 DWP.D	Hexachlorobenzene	Sample	7,746	0	ND		
S1 DOP.D	Hexachlorobenzene	Sample	6,965	0	ND		
S2 SUP.D	Hexachlorobenzene	Sample	7,078	0	ND		
S1 DOP-1.D	Hexachlorobenzene	Sample	7,073	0	ND		
S1 GGC.D	Hexachlorobenzene	Sample	7,563	0	ND		
S1 GGP.D	Hexachlorobenzene	Sample	7,466	0	ND		
S1 DWT.D	Hexachlorobenzene	Sample	6,971	0	ND		
S1 DOT.D	Hexachlorobenzene	Sample	7,756	0	ND		
S2 GJC.D	Hexachlorobenzene	Sample	7,590	0	ND		
S1 SDT.D	Hexachlorobenzene	Sample	6,965	0	ND		
S1 DDC.D	Hexachlorobenzene	Sample	7,568	0	ND		
S1 DOC.D	Hexachlorobenzene	Sample	8,015	0	ND		
S2 GGP.D	Hexachlorobenzene	Sample	6,432	0	ND		
S1 SDC.D	Hexachlorobenzene	Sample	6,438	0	ND		
S1 GGC-1.D	Hexachlorobenzene	Sample	6,970	0	ND		
5 PPb.D	Hexachlorobenzene	Calibration	7,412	46	5,2431	5,0000	104,9
S1 DDO S.D	Hexachlorobenzene	Sample	7,547	0	ND		
S1 DOC S1.D	Hexachlorobenzene	Sample	7,493	0	ND		
S1 DDP S.D	Hexachlorobenzene	Sample	7,401	22	3,4423		
S1 DWT S.D	Hexachlorobenzene	Sample	7,407	5	2,1630		

Aldrine

Data File	Compound	Sample Type	RT	Resp.	Final Conc	Exp. Conc	Accuracy
10 ppb.D	Aldrine	Calibration	11,365	39	9,9877	10,0000	99,9
20 ppb.D	Aldrine	Calibration	11,344	84	18,6841	20,0000	93,4
40 ppb.D	Aldrine	Calibration	11,344	200	40,6013	40,0000	101,5
50 ppb.D	Aldrine	Calibration	11,334	252	50,5119	50,0000	101,0
100 ppb.D	Aldrine	Calibration	11,334	511	99,9468	100,0000	99,9
30-1ppb.D	Aldrine	Calibration	11,334	140	29,2307	30,0000	97,4
S2 DWO.D	Aldrine	Sample	11,365	0	ND		
S1 GGDO.D	Aldrine	Sample	11,490	0	ND		
S2 SUO.D	Aldrine	Sample	11,125	0	ND		
S1 DOO.D	Aldrine	Sample	11,303	0	ND		

Aldrine

Data File	Compound	Sample Type	RT	Resp.	Final Conc	Exp. Conc	Accuracy
S2 GJJ.D	Aldrine	Sample	11,281	0	ND		
S1 SDO.D	Aldrine	Sample	11,114	0	ND		
S1 SDP.D	Aldrine	Sample	11,125	0	ND		
S2 DWP.D	Aldrine	Sample	11,469	0	ND		
S1 DOP.D	Aldrine	Sample	11,323	0	ND		
S2 SUP.D	Aldrine	Sample	11,334	0	ND		
S1 DOP-1.D	Aldrine	Sample	11,292	0	ND		
S1 GGC.D	Aldrine	Sample	11,125	0	ND		
S1 GGP.D	Aldrine	Sample	11,459	0	ND		
S1 DWT.D	Aldrine	Sample	11,177	0	ND		
S1 DOT.D	Aldrine	Sample	11,125	0	ND		
S2 GJC.D	Aldrine	Sample	11,156	0	ND		
S1 SDT.D	Aldrine	Sample	11,282	0	ND		
S1 DDC.D	Aldrine	Sample	11,407	0	ND		
S1 DOC.D	Aldrine	Sample	11,334	0	ND		
S2 GGP.D	Aldrine	Sample	11,146	0	ND		
S1 SDC.D	Aldrine	Sample	10,801	0	ND		
S1 GGC-1.D	Aldrine	Sample	11,104	0	ND		
5 PPb.D	Aldrine	Calibration	11,302	18	6,0374	5,0000	120,7
S1 DDO S.D	Aldrine	Sample	10,739	0	ND		
S1 DOC S1.D	Aldrine	Sample	11,261	0	ND		
S1 DDP S.D	Aldrine	Sample	11,323	9	4,3612		
S1 DWT S.D	Aldrine	Sample	11,292	0	ND		

Chlorpyrifos

Data File	Compound	Sample Type	RT	Resp.	Final Conc	Exp. Conc	Accuracy
10 ppb.D	Chlorpyrifos	Calibration	11,605	16	9,6897	10,0000	96,9
20 ppb.D	Chlorpyrifos	Calibration	11,584	36	18,3902	20,0000	92,0
40 ppb.D	Chlorpyrifos	Calibration	11,595	83	39,5701	40,0000	98,9
50 ppb.D	Chlorpyrifos	Calibration	11,584	107	50,3146	50,0000	100,6
100 ppb.D	Chlorpyrifos	Calibration	11,584	217	100,0974	100,0000	100,1
30-1ppb.D	Chlorpyrifos	Calibration	11,584	63	30,6936	30,0000	102,3
S2 DWO.D	Chlorpyrifos	Sample	11,553	0	ND		
S1 GGDO.D	Chlorpyrifos	Sample	11,334	0	ND		
S2 SUO.D	Chlorpyrifos	Sample	11,574	0	ND		
S1 DOO.D	Chlorpyrifos	Sample			ND		
S2 GJJ.D	Chlorpyrifos	Sample	11,563	15	8,9950		
S1 SDO.D	Chlorpyrifos	Sample	11,323	0	ND		
S1 SDP.D	Chlorpyrifos	Sample	11,125	0	ND		
S2 DWP.D	Chlorpyrifos	Sample	11,522	0	ND		
S1 DOP.D	Chlorpyrifos	Sample	10,916	0	ND		

Chlorpyrifos

Data File	Compound	Sample Type	RT	Resp.	Final Conc	Exp. Conc	Accuracy
S2 SUP.D	Chlorpyrifos	Sample	11,135	0	ND		
S1 DOP-1.D	Chlorpyrifos	Sample			ND		
S1 GGC.D	Chlorpyrifos	Sample	11,281	0	ND		
S1 GGP.D	Chlorpyrifos	Sample	12,065	0	ND		
S1 DWT.D	Chlorpyrifos	Sample			ND		
S1 DOT.D	Chlorpyrifos	Sample	11,553	10	6,9763		
S2 GJC.D	Chlorpyrifos	Sample	12,096	0	ND		
S1 SDT.D	Chlorpyrifos	Sample	11,553	13	8,1930		
S1 DDC.D	Chlorpyrifos	Sample			ND		
S1 DOC.D	Chlorpyrifos	Sample	12,326	0	ND		
S2 GGP.D	Chlorpyrifos	Sample	11,135	0	ND		
S1 SDC.D	Chlorpyrifos	Sample			ND		
S1 GGC-1.D	Chlorpyrifos	Sample	11,135	0	ND		
5 PPb.D	Chlorpyrifos	Calibration	11,553	9	6,2445	5,0000	124,9
S1 DDO S.D	Chlorpyrifos	Sample	11,240	0	ND		
S1 DOC S1.D	Chlorpyrifos	Sample	11,542	0	ND		
S1 DDP S.D	Chlorpyrifos	Sample	11,532	0	ND		
S1 DWT S.D	Chlorpyrifos	Sample	11,543	10	6,8732		

Endosulfan II

Data File	Compound	Sample Type	RT	Resp.	Final Conc	Exp. Conc	Accuracy
10 ppb.D	Endosulfan II	Calibration	14,754	16	9,8852	10,0000	98,9
20 ppb.D	Endosulfan II	Calibration	14,716	25	19,6624	20,0000	98,3
40 ppb.D	Endosulfan II	Calibration	14,700	45	39,6360	40,0000	99,1
50 ppb.D	Endosulfan II	Calibration	14,695	55	50,2322	50,0000	100,5
100 ppb.D	Endosulfan II	Calibration	14,689	103	99,5096	100,0000	99,5
30-1ppb.D	Endosulfan II	Calibration	14,695	38	32,1805	30,0000	107,3
S2 DWO.D	Endosulfan II	Sample	13,774	0	ND		
S1 GGDO.D	Endosulfan II	Sample	14,689	0	ND		
S2 SUO.D	Endosulfan II	Sample	14,641	0	ND		
S1 DOO.D	Endosulfan II	Sample	14,625	35	29,2100		
S2 GJJ.D	Endosulfan II	Sample	14,608	12	6,4961		
S1 SDO.D	Endosulfan II	Sample	14,619	46	40,8853		
S1 SDP.D	Endosulfan II	Sample	14,630	0	ND		
S2 DWP.D	Endosulfan II	Sample	14,625	40	34,5661		
S1 DOP.D	Endosulfan II	Sample	14,625	8	2,0207		
S2 SUP.D	Endosulfan II	Sample	14,624	37	32,0586		
S1 DOP-1.D	Endosulfan II	Sample	14,630	8	2,2285		
S1 GGC.D	Endosulfan II	Sample	14,619	15	8,7871		
S1 GGP.D	Endosulfan II	Sample	14,635	0	ND		
S1 DWT.D	Endosulfan II	Sample	14,635	18	11,6826		

Endosulfan II

Data File	Compound	Sample Type	RT	Resp.	Final Conc	Exp. Conc	Accuracy
S1 DOT.D	Endosulfan II	Sample	14,619	15	9,1162		
S2 GJC.D	Endosulfan II	Sample	14,635	12	6,2162		
S1 SDT.D	Endosulfan II	Sample	14,603	21	14,7670		
S1 DDC.D	Endosulfan II	Sample	14,598	0	ND		
S1 DOC.D	Endosulfan II	Sample	14,614	5	ND		
S2 GGP.D	Endosulfan II	Sample	14,625	22	16,6110		
S1 SDC.D	Endosulfan II	Sample	14,614	35	29,4546		
S1 GGC-1.D	Endosulfan II	Sample	14,608	74	69,9216		
5 PPb.D	Endosulfan II	Calibration	14,609	10	3,8941	5,0000	77,9
S1 DDO S.D	Endosulfan II	Sample	14,732	0	ND		
S1 DOC S1.D	Endosulfan II	Sample	14,608	14	8,4452		
S1 DDP S.D	Endosulfan II	Sample	14,614	0	ND		
S1 DWT S.D	Endosulfan II	Sample	14,609	18	11,8424		

4,4-DDE

Data File	Compound	Sample Type	RT	Resp.	Final Conc	Exp. Conc	Accuracy
10 ppb.D	4,4-DDE	Calibration	16,244	166	12,5322	10,0000	125,3
20 ppb.D	4,4-DDE	Calibration	16,233	251	18,3012	20,0000	91,5
40 ppb.D	4,4-DDE	Calibration	16,222	560	39,2885	40,0000	98,2
50 ppb.D	4,4-DDE	Calibration	16,217	720	50,1795	50,0000	100,4
100 ppb.D	4,4-DDE	Calibration	16,217	1459	100,4760	100,0000	100,5
30-1ppb.D	4,4-DDE	Calibration	16,211	414	29,3769	30,0000	97,9
S2 DWO.D	4,4-DDE	Sample	16,211	7	1,6910		
S1 GGDO.D	4,4-DDE	Sample	16,222	5	1,5690		
S2 SUO.D	4,4-DDE	Sample	16,206	7	1,6783		
S1 DOO.D	4,4-DDE	Sample			ND		
S2 GJJ.D	4,4-DDE	Sample	16,985	0	ND		
S1 SDO.D	4,4-DDE	Sample	16,759	0	ND		
S1 SDP.D	4,4-DDE	Sample			ND		
S2 DWP.D	4,4-DDE	Sample	16,201	0	ND		
S1 DOP.D	4,4-DDE	Sample	16,985	0	ND		
S2 SUP.D	4,4-DDE	Sample			ND		
S1 DOP-1.D	4,4-DDE	Sample	16,195	0	ND		
S1 GGC.D	4,4-DDE	Sample			ND		
S1 GGP.D	4,4-DDE	Sample			ND		
S1 DWT.D	4,4-DDE	Sample	16,324	0	ND		
S1 DOT.D	4,4-DDE	Sample	17,205	0	ND		
S2 GJC.D	4,4-DDE	Sample	16,963	0	ND		
S1 SDT.D	4,4-DDE	Sample	17,039	0	ND		
S1 DDC.D	4,4-DDE	Sample	16,791	0	ND		
S1 DOC.D	4,4-DDE	Sample	16,217	0	ND		

4,4-DDE

Data File	Compound	Sample Type	RT	Resp.	Final Conc	Exp. Conc	Accuracy
S2 GGP.D	4,4-DDE	Sample	15,975	0	ND		
S1 SDC.D	4,4-DDE	Sample			ND		
S1 GGC-1.D	4,4-DDE	Sample	17,001	0	ND		
5 PPb.D	4,4-DDE	Calibration	16,174	53	4,8457	5,0000	96,9
S1 DDO S.D	4,4-DDE	Sample	17,055	0	ND		
S1 DOC S1.D	4,4-DDE	Sample	16,937	0	ND		
S1 DDP S.D	4,4-DDE	Sample	16,141	14	2,1735		
S1 DWT S.D	4,4-DDE	Sample	16,158	6	1,6467		

4,4-DDD

Data File	Compound	Sample Type	RT	Resp.	Final Conc	Exp. Conc	Accuracy
10 ppb.D	4,4-DDD	Calibration	18,194	57	11,8470	10,0000	118,5
20 ppb.D	4,4-DDD	Calibration	18,172	97	19,0677	20,0000	95,3
40 ppb.D	4,4-DDD	Calibration	18,178	197	37,5003	40,0000	93,8
50 ppb.D	4,4-DDD	Calibration	18,178	267	50,2745	50,0000	100,5
100 ppb.D	4,4-DDD	Calibration	18,172	543	100,8968	100,0000	100,9
30-1ppb.D	4,4-DDD	Calibration	18,173	155	29,7876	30,0000	99,3
S2 DWO.D	4,4-DDD	Sample	18,248	13	3,7659		
S1 GGDO.D	4,4-DDD	Sample	18,253	16	4,2064		
S2 SUO.D	4,4-DDD	Sample	18,344	1727	317,9851		
S1 DOO.D	4,4-DDD	Sample	18,237	17	4,4821		
S2 GJJ.D	4,4-DDD	Sample	18,237	15	4,0885		
S1 SDO.D	4,4-DDD	Sample	18,242	15	4,1848		
S1 SDP.D	4,4-DDD	Sample	18,226	19	4,8826		
S2 DWP.D	4,4-DDD	Sample	18,237	8	2,8902		
S1 DOP.D	4,4-DDD	Sample	18,237	26	6,1581		
S2 SUP.D	4,4-DDD	Sample	18,248	22	5,3525		
S1 DOP-1.D	4,4-DDD	Sample	18,237	15	4,1526		
S1 GGC.D	4,4-DDD	Sample	18,258	23	5,5163		
S1 GGP.D	4,4-DDD	Sample	18,226	15	4,0388		
S1 DWT.D	4,4-DDD	Sample	18,232	12	3,4701		
S1 DOT.D	4,4-DDD	Sample	18,221	15	4,1917		
S2 GJC.D	4,4-DDD	Sample	18,231	14	3,8790		
S1 SDT.D	4,4-DDD	Sample	18,237	13	3,7660		
S1 DDC.D	4,4-DDD	Sample	18,226	19	4,8692		
S1 DOC.D	4,4-DDD	Sample	18,221	15	4,0880		
S2 GGP.D	4,4-DDD	Sample	18,226	17	4,4756		
S1 SDC.D	4,4-DDD	Sample	18,226	16	4,2089		
S1 GGC-1.D	4,4-DDD	Sample	18,221	14	4,0023		
5 PPb.D	4,4-DDD	Calibration	18,129	23	5,6262	5,0000	112,5
S1 DDO S.D	4,4-DDD	Sample	18,232	36	8,0349		

4,4-DDD

Data File	Compound	Sample Type	RT	Resp.	Final Conc	Exp. Conc	Accuracy
S1 DOC S1.D	4,4-DDD	Sample	18,221	20	4,9808		
S1 DDP S.D	4,4-DDD	Sample	18,226	24	5,8155		
S1 DWT S.D	4,4-DDD	Sample	18,210	16	4,3225		

4,4-DDT

Data File	Compound	Sample Type	RT	Resp.	Final Conc	Exp. Conc	Accuracy
10 ppb.D	4,4-DDT	Calibration	19,542	9	10,2601	10,0000	102,6
20 ppb.D	4,4-DDT	Calibration	19,526	12	13,5304	20,0000	67,7
40 ppb.D	4,4-DDT	Calibration	19,526	32	38,4243	40,0000	96,1
50 ppb.D	4,4-DDT	Calibration	19,516	41	49,3541	50,0000	98,7
100 ppb.D	4,4-DDT	Calibration	19,510	83	100,5583	100,0000	100,6
30-1ppb.D	4,4-DDT	Calibration	19,505	27	31,1950	30,0000	104,0
S2 DWO.D	4,4-DDT	Sample			ND		
S1 GGDO.D	4,4-DDT	Sample	19,763	0	ND		
S2 SUO.D	4,4-DDT	Sample	19,639	53	63,5603		
S1 DOO.D	4,4-DDT	Sample	19,763	0	ND		
S2 GJJ.D	4,4-DDT	Sample			ND		
S1 SDO.D	4,4-DDT	Sample	19,763	0	ND		
S1 SDP.D	4,4-DDT	Sample	19,269	0	ND		
S2 DWP.D	4,4-DDT	Sample			ND		
S1 DOP.D	4,4-DDT	Sample			ND		
S2 SUP.D	4,4-DDT	Sample	19,854	0	ND		
S1 DOP-1.D	4,4-DDT	Sample			ND		
S1 GGC.D	4,4-DDT	Sample			ND		
S1 GGP.D	4,4-DDT	Sample			ND		
S1 DWT.D	4,4-DDT	Sample			ND		
S1 DOT.D	4,4-DDT	Sample			ND		
S2 GJC.D	4,4-DDT	Sample	19,258	0	ND		
S1 SDT.D	4,4-DDT	Sample			ND		
S1 DDC.D	4,4-DDT	Sample	20,327	0	ND		
S1 DOC.D	4,4-DDT	Sample	20,031	0	ND		
S2 GGP.D	4,4-DDT	Sample			ND		
S1 SDC.D	4,4-DDT	Sample			ND		
S1 GGC-1.D	4,4-DDT	Sample	20,166	0	ND		
5 PPb.D	4,4-DDT	Calibration	19,494	5	5,2082	5,0000	104,2
S1 DDO S.D	4,4-DDT	Sample	19,473	0	ND		
S1 DOC S1.D	4,4-DDT	Sample	19,849	0	ND		
S1 DDP S.D	4,4-DDT	Sample			ND		
S1 DWT S.D	4,4-DDT	Sample	18,833	0	ND		

**Batch Path**	C:\Users\USER\OneDrive\Desktop\JU_Pesticide\MA\MA\QuantResults\Mohammed_Pest.batch.bin		
**Analysis Time**	13 Dec 2024 11:34	**Analyst Name**	DESKTOP-MRRPPC4\USER
**Report Time**	13 Dec 2024 11:37:20	**Reporter Name**	DESKTOP-MRRPPC4\USER
**Last Calib Update**	13 Dec 2024 11:34	**Batch State**	Processed
**Quant Batch Version**	10.0	**Quant Report Version**	10.0

Lindane

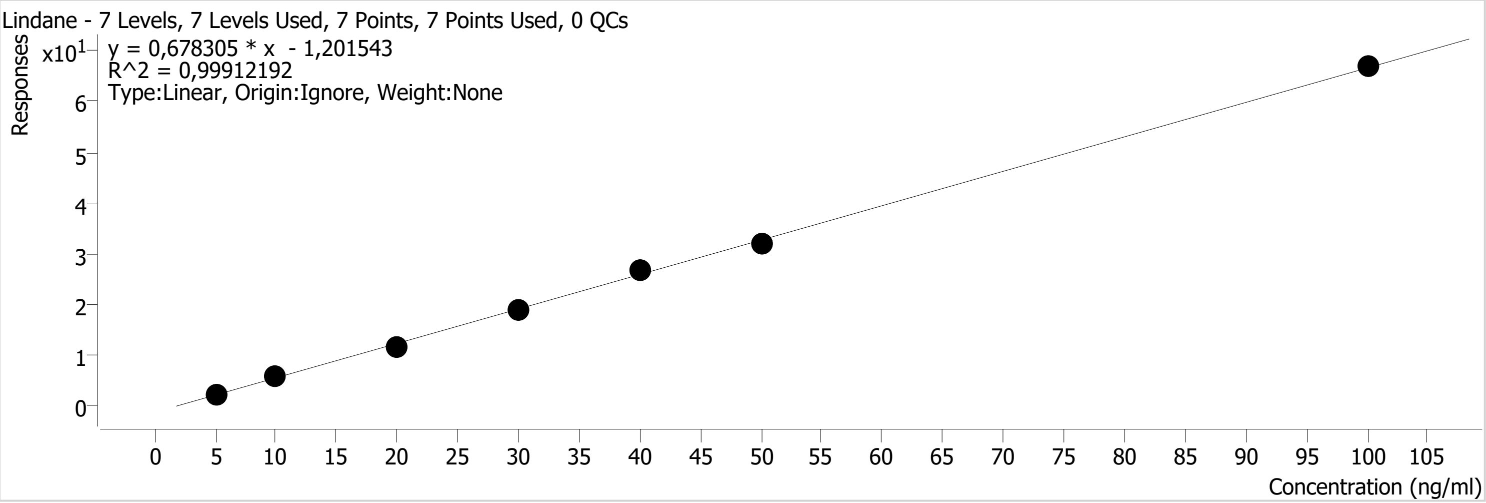

Calibration STD Path	Cal Type	Level	Enabled	Resp.	Exp. Conc	Resp. Factor
C:\Users\USER\OneDrive\Desktop\JU_Pesticide\MA\MA\5 PPb.D	Calibration	1	x	2	5,0000	0,4856
C:\Users\USER\OneDrive\Desktop\JU_Pesticide\MA\MA\10 ppb.D	Calibration	2	x	6	10,0000	0,5969
C:\Users\USER\OneDrive\Desktop\JU_Pesticide\MA\MA\20 ppb.D	Calibration	3	x	12	20,0000	0,5857
C:\Users\USER\OneDrive\Desktop\JU_Pesticide\MA\MA\30-1ppb.D	Calibration	4	x	19	30,0000	0,6270
C:\Users\USER\OneDrive\Desktop\JU_Pesticide\MA\MA\40 ppb.D	Calibration	5	x	27	40,0000	0,6735
C:\Users\USER\OneDrive\Desktop\JU_Pesticide\MA\MA\50 ppb.D	Calibration	6	x	32	50,0000	0,6373
C:\Users\USER\OneDrive\Desktop\JU_Pesticide\MA\MA\100 ppb.D	Calibration	7	x	67	100,0000	0,6683

**Batch Path**	C:\Users\USER\OneDrive\Desktop\JU_Pesticide\MA\MA\QuantResults\Mohammed_Pest.batch.bin		
**Analysis Time**	13 Dec 2024 11:34	**Analyst Name**	DESKTOP-MRRPPC4\USER
**Report Time**	13 Dec 2024 11:37:21	**Reporter Name**	DESKTOP-MRRPPC4\USER
**Last Calib Update**	13 Dec 2024 11:34	**Batch State**	Processed
**Quant Batch Version**	10.0	**Quant Report Version**	10.0

Hexachlorobenzene

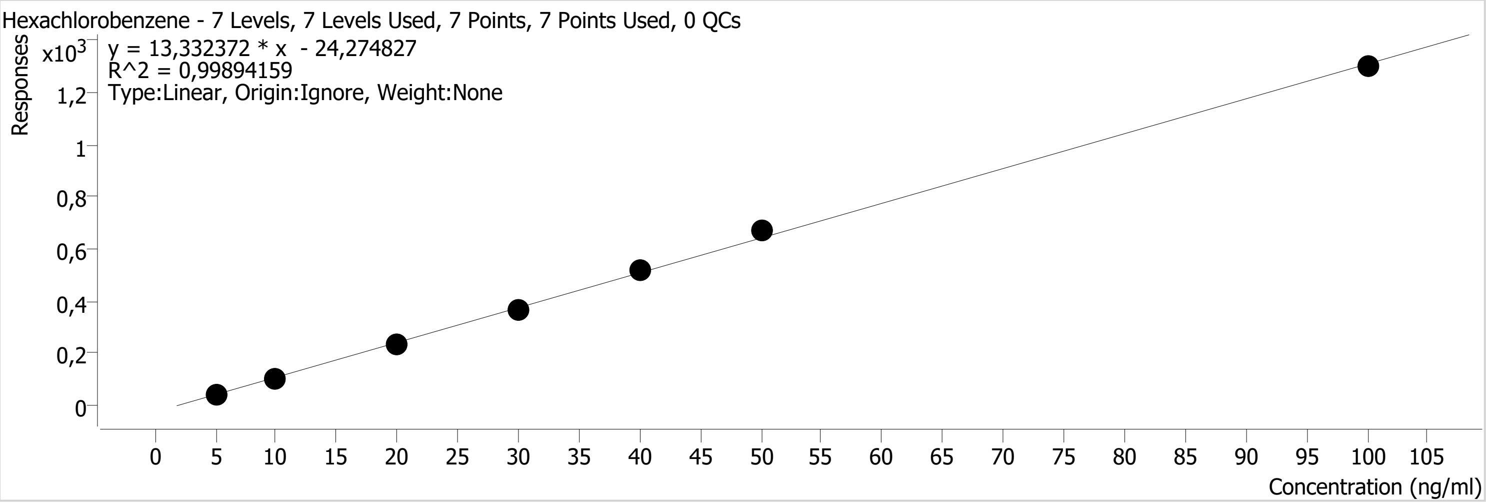

Calibration STD Path	Cal Type	Level	Enabled	Resp.	Exp. Conc	Resp. Factor
C:\Users\USER\OneDrive\Desktop\JU_Pesticide\MA\MA\5 PPb.D	Calibration	1	x	46	5,0000	9,1256
C:\Users\USER\OneDrive\Desktop\JU_Pesticide\MA\MA\10 ppb.D	Calibration	2	x	102	10,0000	10,2364
C:\Users\USER\OneDrive\Desktop\JU_Pesticide\MA\MA\20 ppb.D	Calibration	3	x	232	20,0000	11,6133
C:\Users\USER\OneDrive\Desktop\JU_Pesticide\MA\MA\30-1ppb.D	Calibration	4	x	365	30,0000	12,1673
C:\Users\USER\OneDrive\Desktop\JU_Pesticide\MA\MA\40 ppb.D	Calibration	5	x	518	40,0000	12,9427
C:\Users\USER\OneDrive\Desktop\JU_Pesticide\MA\MA\50 ppb.D	Calibration	6	x	669	50,0000	13,3783
C:\Users\USER\OneDrive\Desktop\JU_Pesticide\MA\MA\100 ppb.D	Calibration	7	x	1298	100,0000	12,9793

**Batch Path**	C:\Users\USER\OneDrive\Desktop\JU_Pesticide\MA\MA\QuantResults\Mohammed_Pest.batch.bin		
**Analysis Time**	13 Dec 2024 11:34	**Analyst Name**	DESKTOP-MRRPPC4\USER
**Report Time**	13 Dec 2024 11:37:21	**Reporter Name**	DESKTOP-MRRPPC4\USER
**Last Calib Update**	13 Dec 2024 11:34	**Batch State**	Processed
**Quant Batch Version**	10.0	**Quant Report Version**	10.0

Aldrine

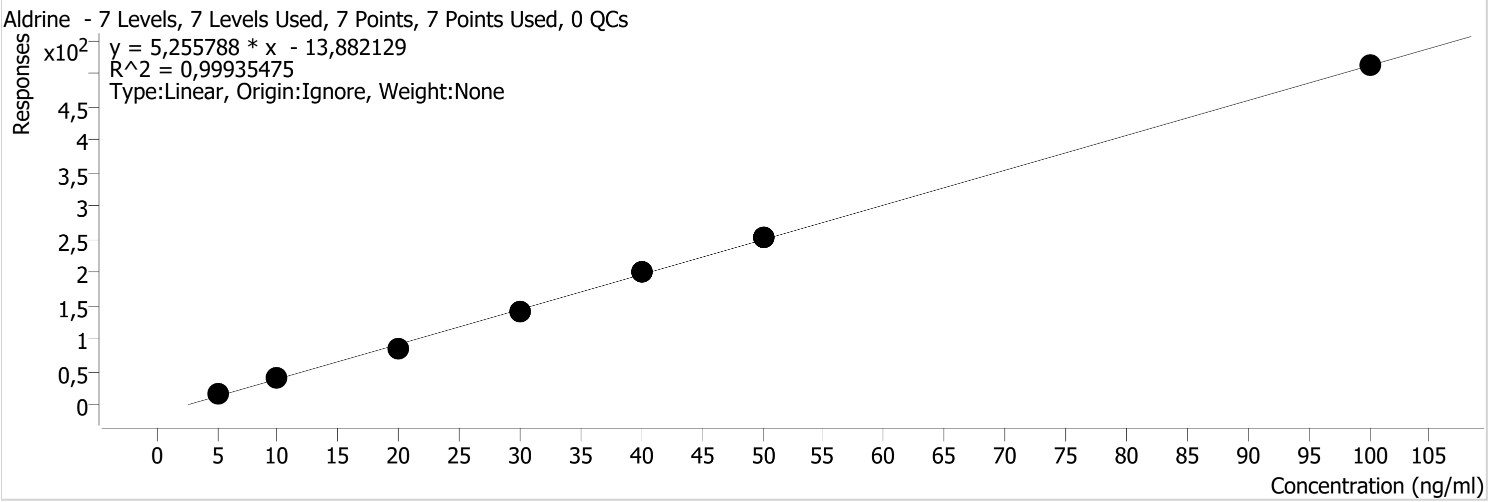

Calibration STD Path	Cal Type	Level	Enabled	Resp.	Exp. Conc	Resp. Factor
C:\Users\USER\OneDrive\Desktop\JU_Pesticide\MA\MA\5 PPb.D	Calibration	1	x	18	5,0000	3,5699
C:\Users\USER\OneDrive\Desktop\JU_Pesticide\MA\MA\10 ppb.D	Calibration	2	x	39	10,0000	3,8611
C:\Users\USER\OneDrive\Desktop\JU_Pesticide\MA\MA\20 ppb.D	Calibration	3	x	84	20,0000	4,2159
C:\Users\USER\OneDrive\Desktop\JU_Pesticide\MA\MA\30-1ppb.D	Calibration	4	x	140	30,0000	4,6583
C:\Users\USER\OneDrive\Desktop\JU_Pesticide\MA\MA\40 ppb.D	Calibration	5	x	200	40,0000	4,9877
C:\Users\USER\OneDrive\Desktop\JU_Pesticide\MA\MA\50 ppb.D	Calibration	6	x	252	50,0000	5,0320
C:\Users\USER\OneDrive\Desktop\JU_Pesticide\MA\MA\100 ppb.D	Calibration	7	x	511	100,0000	5,1142

**Batch Path**	C:\Users\USER\OneDrive\Desktop\JU_Pesticide\MA\MA\QuantResults\Mohammed_Pest.batch.bin		
**Analysis Time**	13 Dec 2024 11:34	**Analyst Name**	DESKTOP-MRRPPC4\USER
**Report Time**	13 Dec 2024 11:37:22	**Reporter Name**	DESKTOP-MRRPPC4\USER
**Last Calib Update**	13 Dec 2024 11:34	**Batch State**	Processed
**Quant Batch Version**	10.0	**Quant Report Version**	10.0

Chlorpyrifos

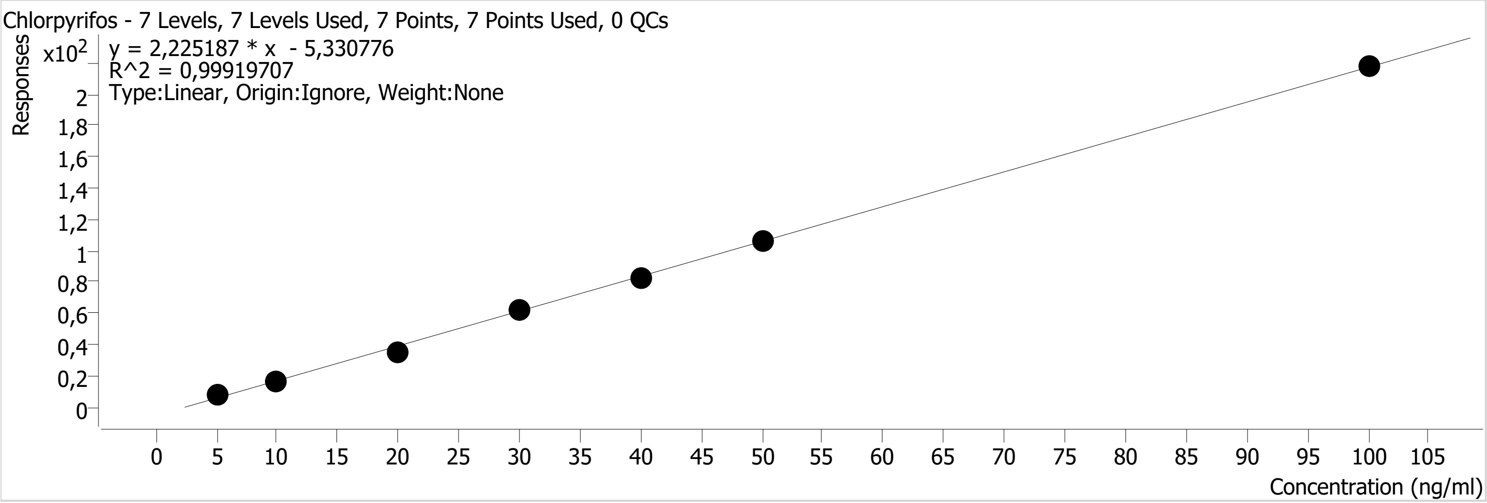

Calibration STD Path	Cal Type	Level	Enabled	Resp.	Exp. Conc	Resp. Factor
C:\Users\USER\OneDrive\Desktop\JU_Pesticide\MA\MA\5 PPb.D	Calibration	1	x	9	5,0000	1,7129
C:\Users\USER\OneDrive\Desktop\JU_Pesticide\MA\MA\10 ppb.D	Calibration	2	x	16	10,0000	1,6231
C:\Users\USER\OneDrive\Desktop\JU_Pesticide\MA\MA\20 ppb.D	Calibration	3	x	36	20,0000	1,7795
C:\Users\USER\OneDrive\Desktop\JU_Pesticide\MA\MA\30-1ppb.D	Calibration	4	x	63	30,0000	2,0989
C:\Users\USER\OneDrive\Desktop\JU_Pesticide\MA\MA\40 ppb.D	Calibration	5	x	83	40,0000	2,0680
C:\Users\USER\OneDrive\Desktop\JU_Pesticide\MA\MA\50 ppb.D	Calibration	6	x	107	50,0000	2,1326
C:\Users\USER\OneDrive\Desktop\JU_Pesticide\MA\MA\100 ppb.D	Calibration	7	x	217	100,0000	2,1740

Quantitative Analysis Complete Report

**Batch Path**	C:\Users\USER\OneDrive\Desktop\JU_Pesticide\MA\MA\QuantResults\Mohammed_Pest.batch.bin		
**Analysis Time**	13 Dec 2024 11:34	**Analyst Name**	DESKTOP-MRRPPC4\USER
**Report Time**	13 Dec 2024 11:37:22	**Reporter Name**	DESKTOP-MRRPPC4\USER
**Last Calib Update**	13 Dec 2024 11:34	**Batch State**	Processed
**Quant Batch Version**	10.0	**Quant Report Version**	10.0

Endosulfan II

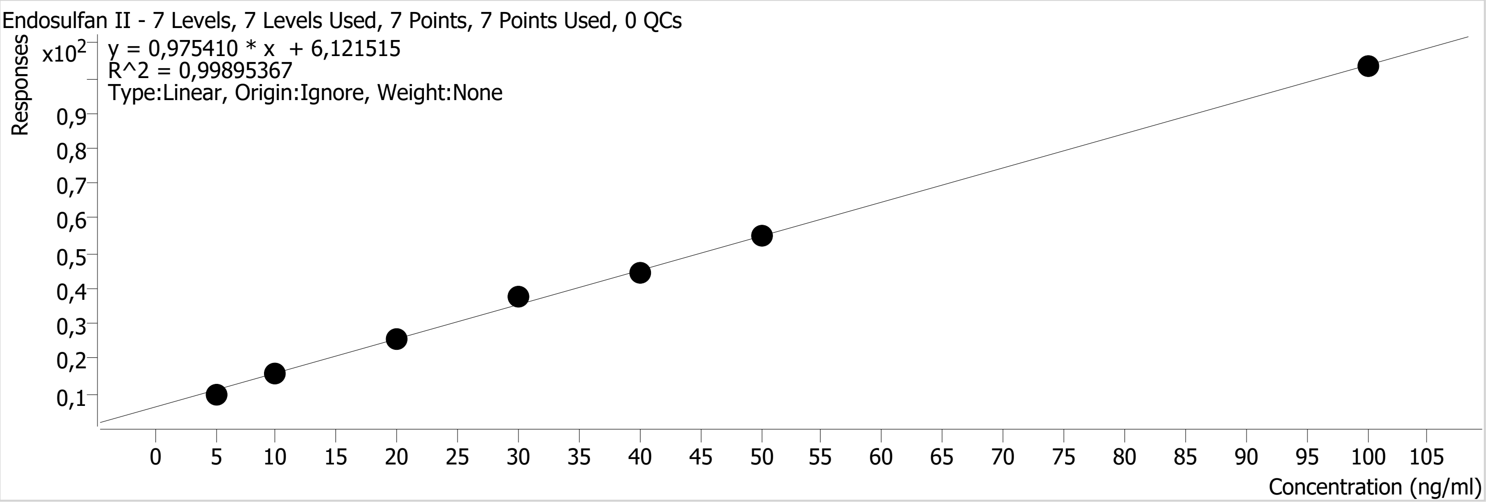

Calibration STD Path	Cal Type	Level	Enabled	Resp.	Exp. Conc	Resp. Factor
C:\Users\USER\OneDrive\Desktop\JU_Pesticide\MA\MA\5 PPb.D	Calibration	1	x	10	5,0000	1,9840
C:\Users\USER\OneDrive\Desktop\JU_Pesticide\MA\MA\10 ppb.D	Calibration	2	x	16	10,0000	1,5764
C:\Users\USER\OneDrive\Desktop\JU_Pesticide\MA\MA\20 ppb.D	Calibration	3	x	25	20,0000	1,2650
C:\Users\USER\OneDrive\Desktop\JU_Pesticide\MA\MA\30-1ppb.D	Calibration	4	x	38	30,0000	1,2504
C:\Users\USER\OneDrive\Desktop\JU_Pesticide\MA\MA\40 ppb.D	Calibration	5	x	45	40,0000	1,1196
C:\Users\USER\OneDrive\Desktop\JU_Pesticide\MA\MA\50 ppb.D	Calibration	6	x	55	50,0000	1,1024
C:\Users\USER\OneDrive\Desktop\JU_Pesticide\MA\MA\100 ppb.D	Calibration	7	x	103	100,0000	1,0318

**Batch Path**	C:\Users\USER\OneDrive\Desktop\JU_Pesticide\MA\MA\QuantResults\Mohammed_Pest.batch.bin		
**Analysis Time**	13 Dec 2024 11:34	**Analyst Name**	DESKTOP-MRRPPC4\USER
**Report Time**	13 Dec 2024 11:37:22	**Reporter Name**	DESKTOP-MRRPPC4\USER
**Last Calib Update**	13 Dec 2024 11:34	**Batch State**	Processed
**Quant Batch Version**	10.0	**Quant Report Version**	10.0

4,4-DDE

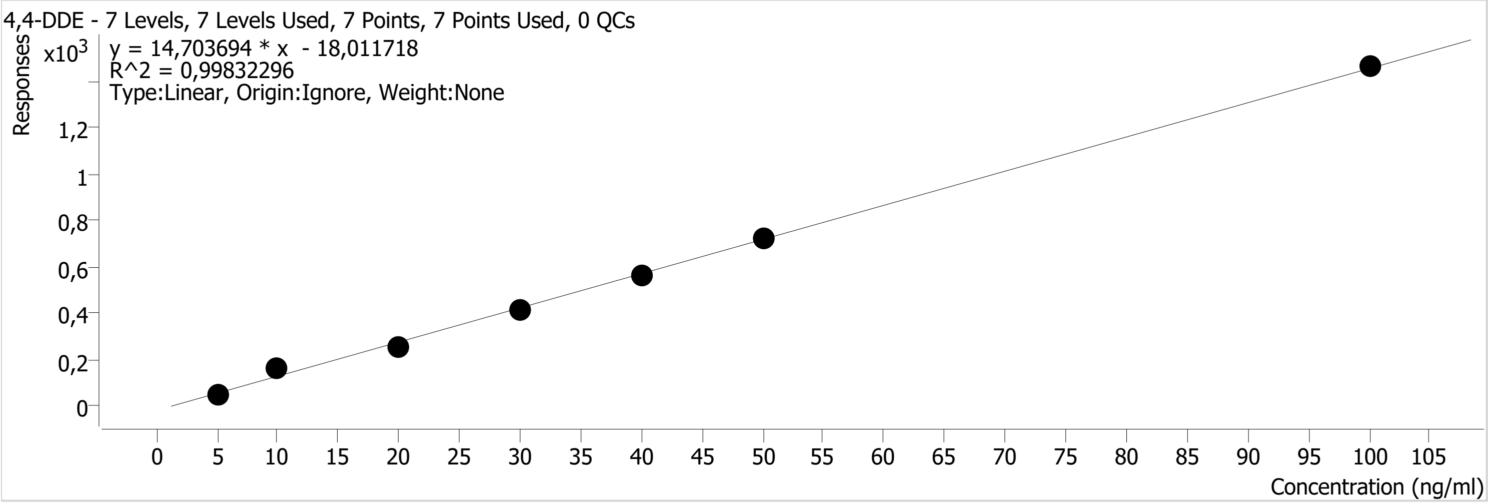

Calibration STD Path	Cal Type	Level	Enabled	Resp.	Exp. Conc	Resp. Factor
C:\Users\USER\OneDrive\Desktop\JU_Pesticide\MA\MA\5 PPb.D	Calibration	1	x	53	5,0000	10,6475
C:\Users\USER\OneDrive\Desktop\JU_Pesticide\MA\MA\10 ppb.D	Calibration	2	x	166	10,0000	16,6258
C:\Users\USER\OneDrive\Desktop\JU_Pesticide\MA\MA\20 ppb.D	Calibration	3	x	251	20,0000	12,5542
C:\Users\USER\OneDrive\Desktop\JU_Pesticide\MA\MA\30-1ppb.D	Calibration	4	x	414	30,0000	13,7979
C:\Users\USER\OneDrive\Desktop\JU_Pesticide\MA\MA\40 ppb.D	Calibration	5	x	560	40,0000	13,9918
C:\Users\USER\OneDrive\Desktop\JU_Pesticide\MA\MA\50 ppb.D	Calibration	6	x	720	50,0000	14,3962
C:\Users\USER\OneDrive\Desktop\JU_Pesticide\MA\MA\100 ppb.D	Calibration	7	x	1459	100,0000	14,5936

**Batch Path**	C:\Users\USER\OneDrive\Desktop\JU_Pesticide\MA\MA\QuantResults\Mohammed_Pest.batch.bin		
**Analysis Time**	13 Dec 2024 11:34	**Analyst Name**	DESKTOP-MRRPPC4\USER
**Report Time**	13 Dec 2024 11:37:22	**Reporter Name**	DESKTOP-MRRPPC4\USER
**Last Calib Update**	13 Dec 2024 11:34	**Batch State**	Processed
**Quant Batch Version**	10.0	**Quant Report Version**	10.0

4,4-DDD

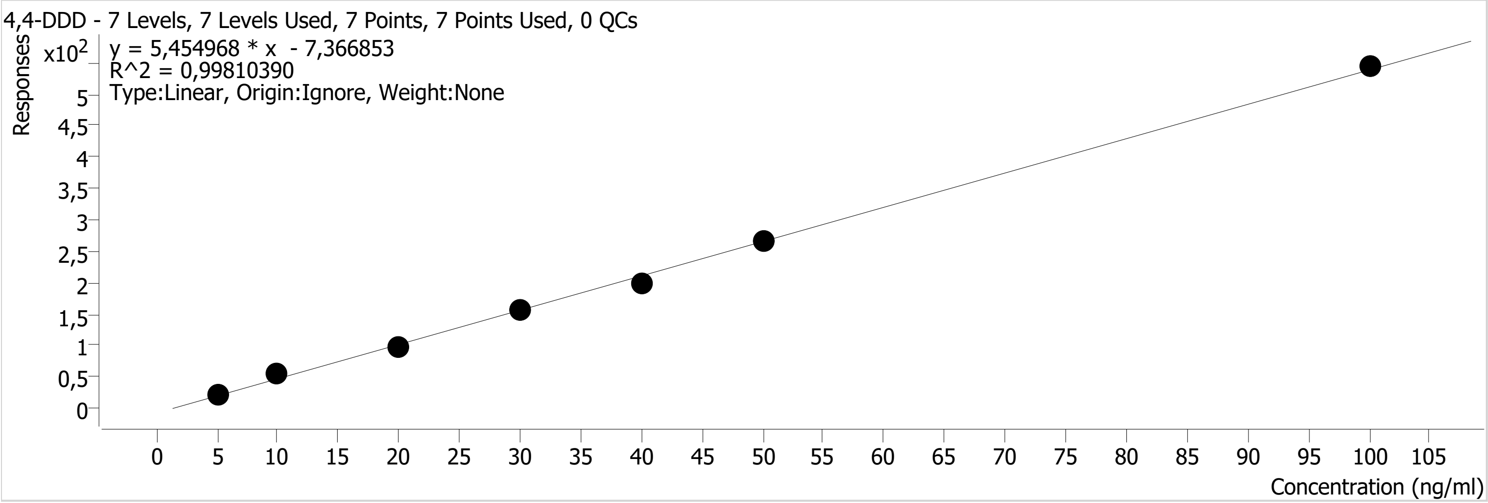

Calibration STD Path	Cal Type	Level	Enabled	Resp.	Exp. Conc	Resp. Factor
C:\Users\USER\OneDrive\Desktop\JU_Pesticide\MA\MA\5 PPb.D	Calibration	1	x	23	5,0000	4,6648
C:\Users\USER\OneDrive\Desktop\JU_Pesticide\MA\MA\10 ppb.D	Calibration	2	x	57	10,0000	5,7258
C:\Users\USER\OneDrive\Desktop\JU_Pesticide\MA\MA\20 ppb.D	Calibration	3	x	97	20,0000	4,8323
C:\Users\USER\OneDrive\Desktop\JU_Pesticide\MA\MA\30-1ppb.D	Calibration	4	x	155	30,0000	5,1708
C:\Users\USER\OneDrive\Desktop\JU_Pesticide\MA\MA\40 ppb.D	Calibration	5	x	197	40,0000	4,9299
C:\Users\USER\OneDrive\Desktop\JU_Pesticide\MA\MA\50 ppb.D	Calibration	6	x	267	50,0000	5,3376
C:\Users\USER\OneDrive\Desktop\JU_Pesticide\MA\MA\100 ppb.D	Calibration	7	x	543	100,0000	5,4302

**Batch Path**	C:\Users\USER\OneDrive\Desktop\JU_Pesticide\MA\MA\QuantResults\Mohammed_Pest.batch.bin		
**Analysis Time**	13 Dec 2024 11:34	**Analyst Name**	DESKTOP-MRRPPC4\USER
**Report Time**	13 Dec 2024 11:37:22	**Reporter Name**	DESKTOP-MRRPPC4\USER
**Last Calib Update**	13 Dec 2024 11:34	**Batch State**	Processed
**Quant Batch Version**	10.0	**Quant Report Version**	10.0

4,4-DDT

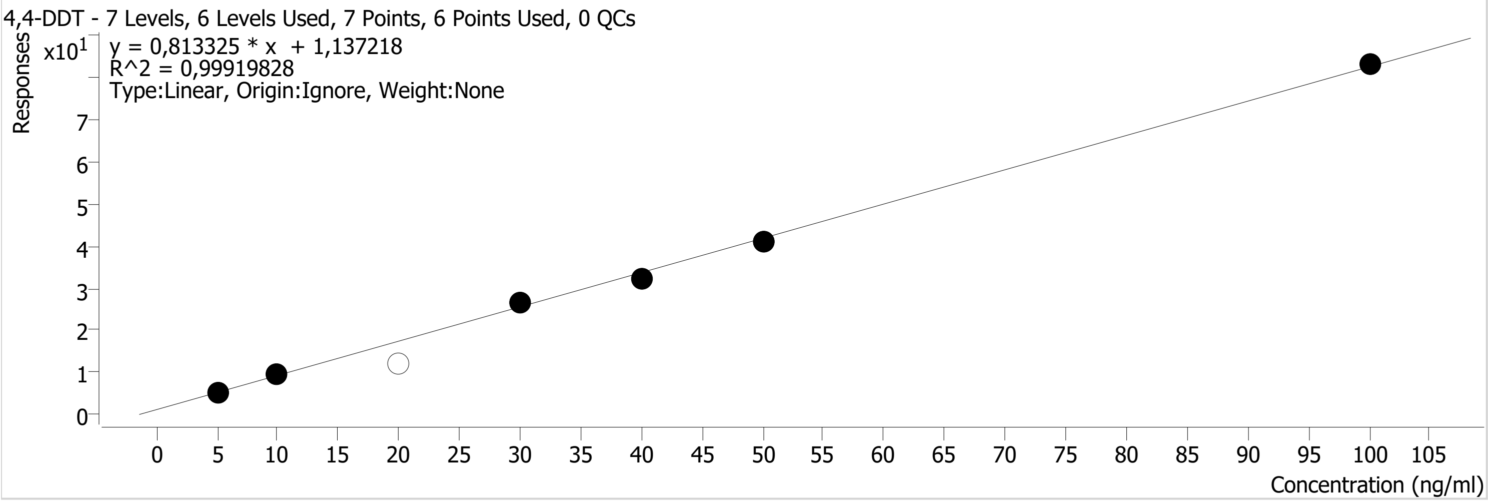

Calibration STD Path	Cal Type	Level	Enabled	Resp.	Exp. Conc	Resp. Factor
C:\Users\USER\OneDrive\Desktop\JU_Pesticide\MA\MA\5 PPb.D	Calibration	1	x	5	5,0000	1,0746
C:\Users\USER\OneDrive\Desktop\JU_Pesticide\MA\MA\10 ppb.D	Calibration	2	x	9	10,0000	0,9482
C:\Users\USER\OneDrive\Desktop\JU_Pesticide\MA\MA\20 ppb.D	Calibration	3		12	20,0000	0,6071
C:\Users\USER\OneDrive\Desktop\JU_Pesticide\MA\MA\30-1ppb.D	Calibration	4	x	27	30,0000	0,8836
C:\Users\USER\OneDrive\Desktop\JU_Pesticide\MA\MA\40 ppb.D	Calibration	5	x	32	40,0000	0,8097
C:\Users\USER\OneDrive\Desktop\JU_Pesticide\MA\MA\50 ppb.D	Calibration	6	x	41	50,0000	0,8256
C:\Users\USER\OneDrive\Desktop\JU_Pesticide\MA\MA\100 ppb.D	Calibration	7	x	83	100,0000	0,8292

Quantitative Analysis Complete Report

**Batch Path**	C:\Users\USER\OneDrive\Desktop\JU_Pesticide\MA\MA\QuantResults\Mohammed_Pest.batch.bin		
**Analysis Time**	13 Dec 2024 11:34	**Analyst Name**	DESKTOP-MRRPPC4\USER
**Report Time**	13 Dec 2024 11:37:22	**Reporter Name**	DESKTOP-MRRPPC4\USER
**Last Calib Update**	13 Dec 2024 11:34	**Batch State**	Processed
**Quant Batch Version**	10.0	**Quant Report Version**	10.0

**Acq. Time**	12 Nov 2024 17:17	**Data File**	10 ppb.D
**Sample Type**	Cal	**Sample Name**	Pest
**Dilution**	1	**Acq. Method**	pesticide std 12.11.2024

Sample Chromatogram

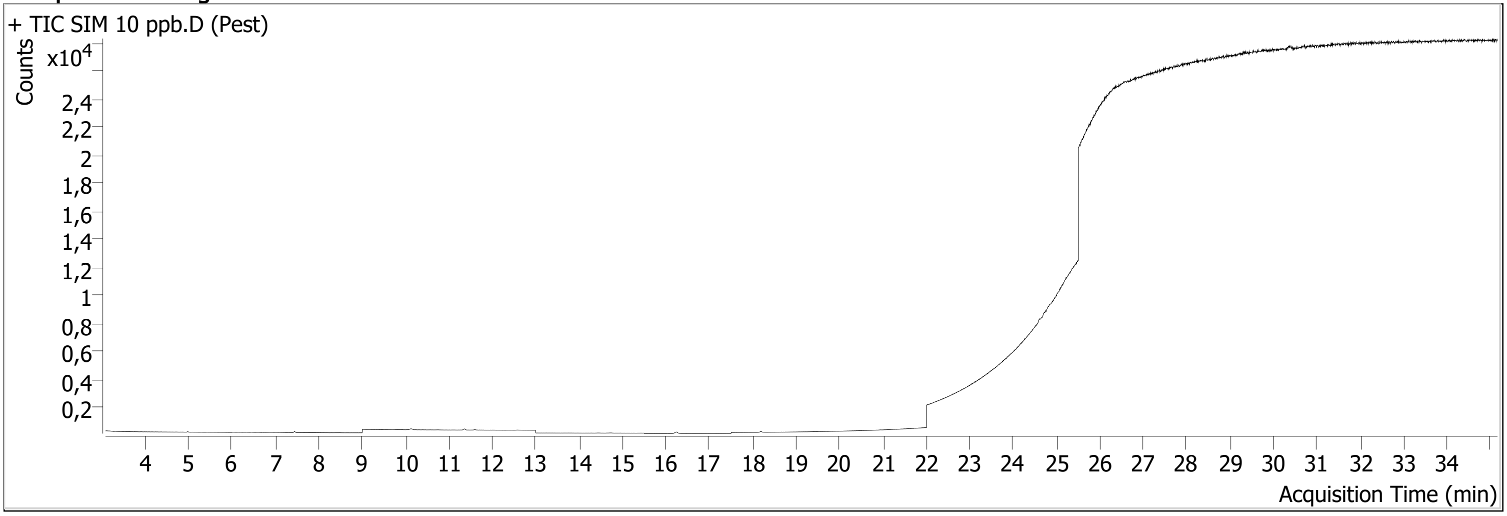

Compound	Transition	RT	Resp.	Final Conc	Units
Lindane	219,0	4,987	6	10,5719	ng/ml
Hexachlorobenzene	284,0	7,444	102	9,4986	ng/ml
Aldrine	293,0	11,365	39	9,9877	ng/ml
Chlorpyrifos	314,0	11,605	16	9,6897	ng/ml
Endosulfan II	339,0	14,754	16	9,8852	ng/ml
4,4-DDE	318,0	16,244	166	12,5322	ng/ml
4,4-DDD	237,0	18,194	57	11,8470	ng/ml
4,4-DDT	237,0	19,542	9	10,2601	ng/ml

Lindane

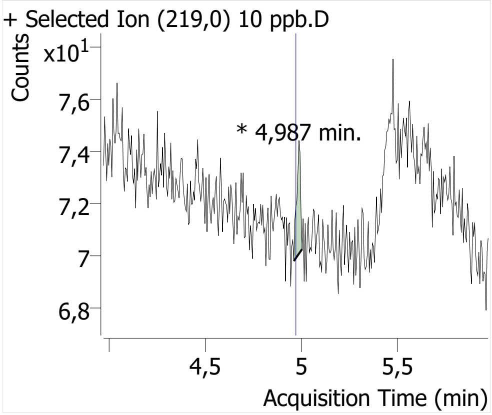

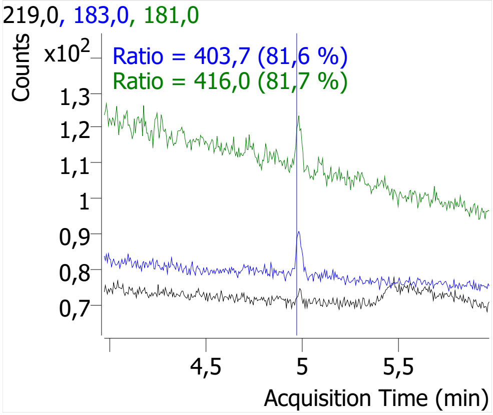

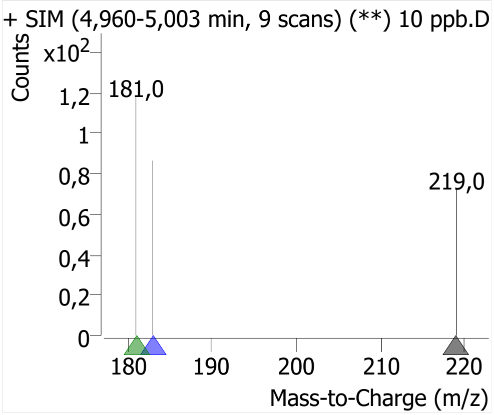

Hexachlorobenzene

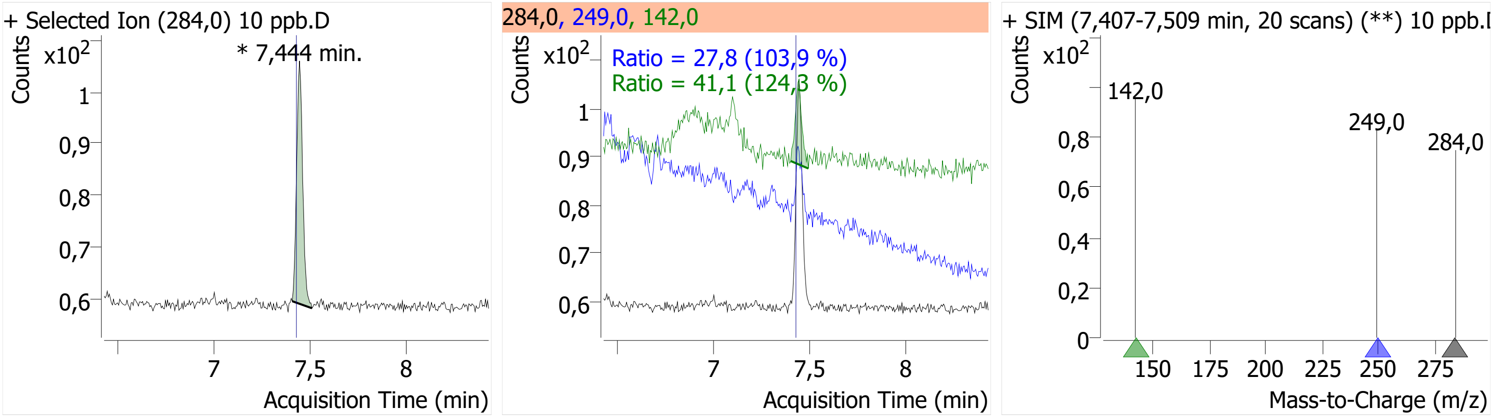

Aldrine

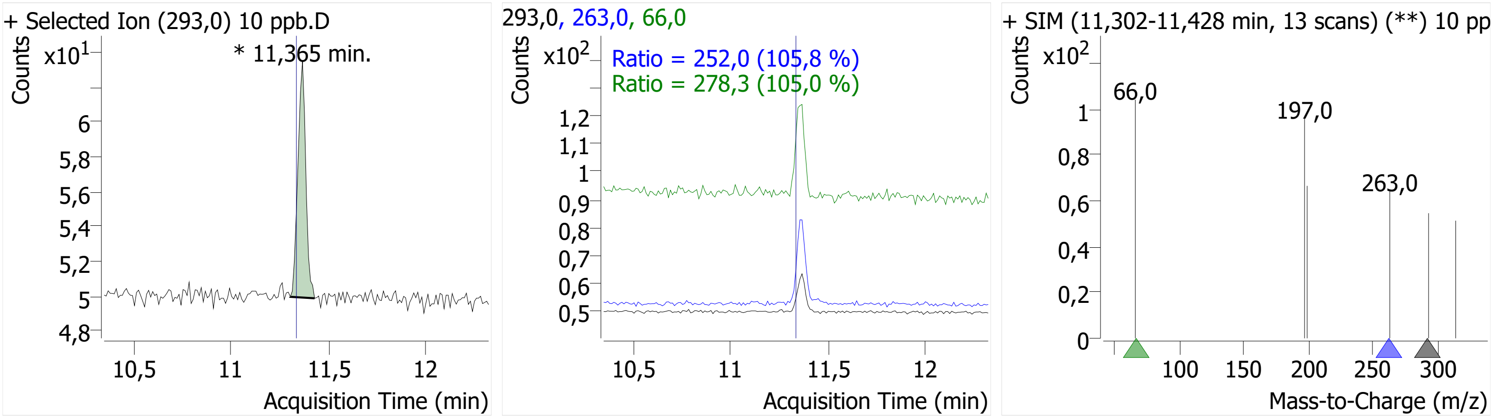

Chlorpyrifos

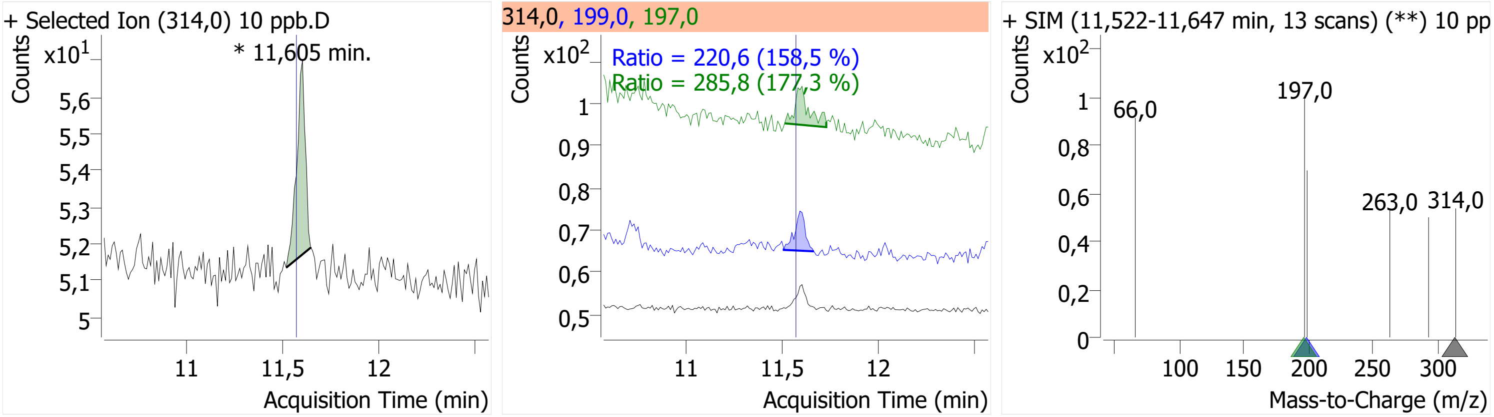

Endosulfan II

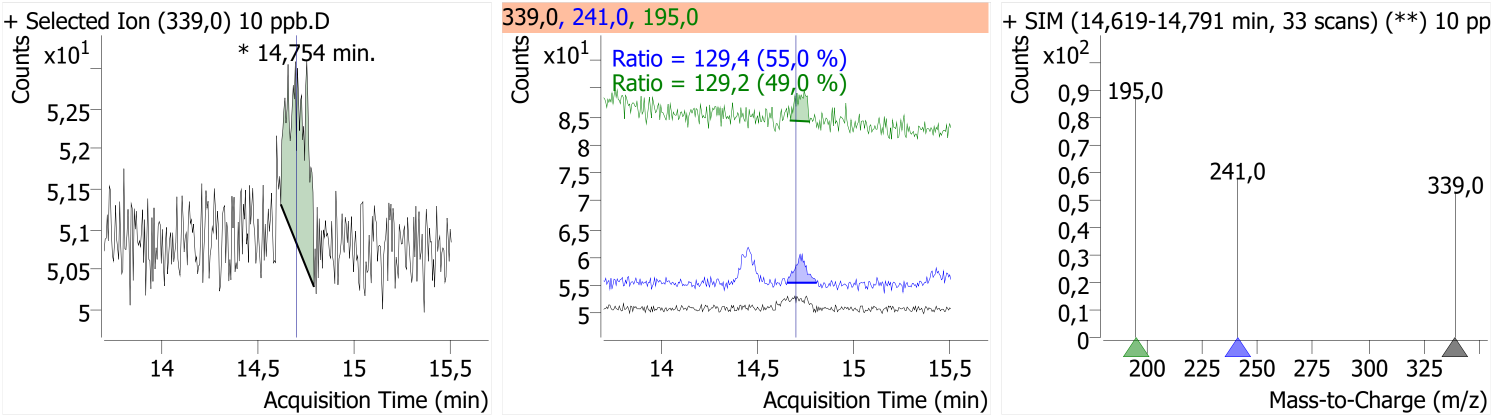

 Generated at 11:37 on 13 Dec 2024

4,4-DDE

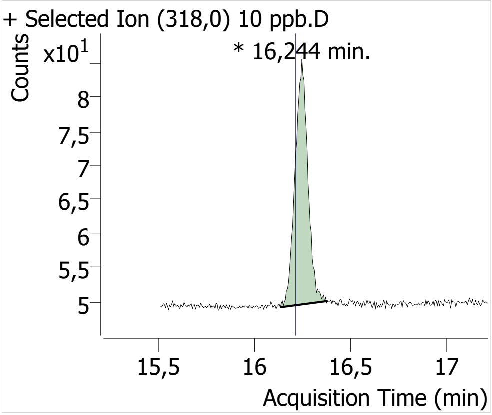

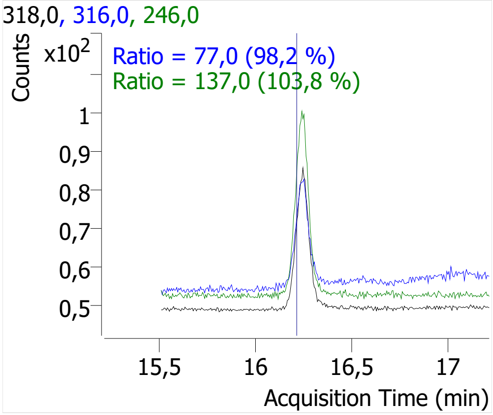

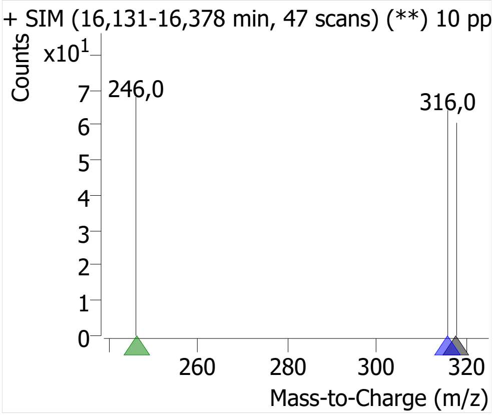

4,4-DDD

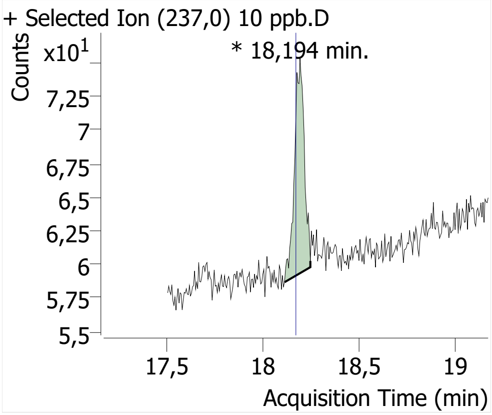

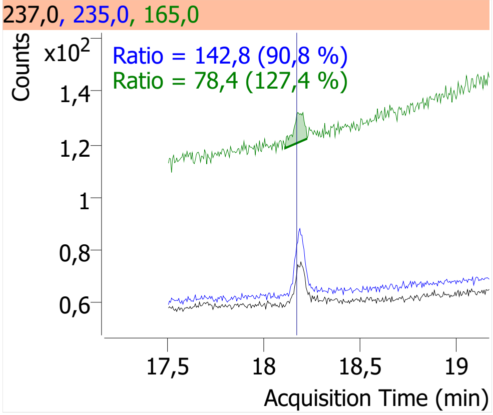

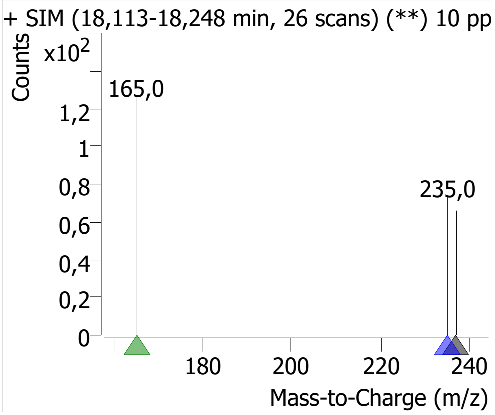

4,4-DDT

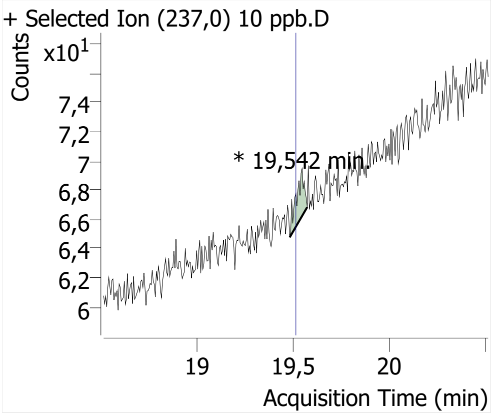

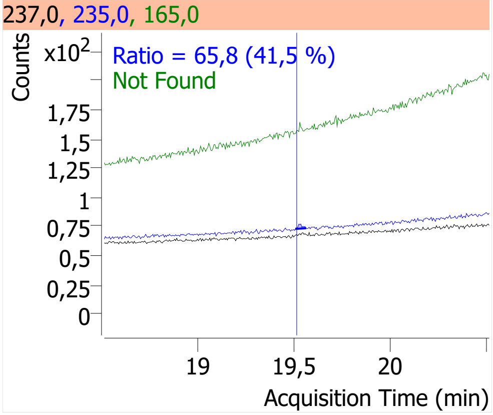

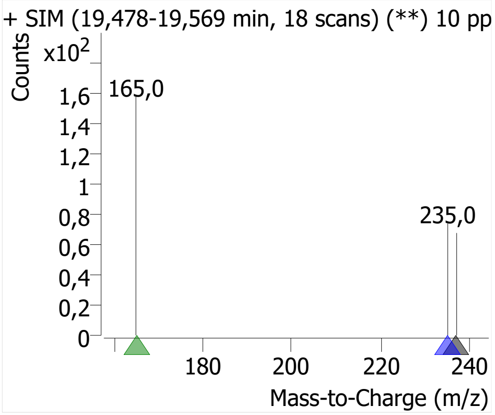

Quantitative Analysis Complete Report

**Batch Path**	C:\Users\USER\OneDrive\Desktop\JU_Pesticide\MA\MA\QuantResults\Mohammed_Pest.batch.bin		
**Analysis Time**	13 Dec 2024 11:34	**Analyst Name**	DESKTOP-MRRPPC4\USER
**Report Time**	13 Dec 2024 11:37:23	**Reporter Name**	DESKTOP-MRRPPC4\USER
**Last Calib Update**	13 Dec 2024 11:34	**Batch State**	Processed
**Quant Batch Version**	10.0	**Quant Report Version**	10.0

**Acq. Time**	12 Nov 2024 17:58	**Data File**	20 ppb.D
**Sample Type**	Cal	**Sample Name**	Pest
**Dilution**	1	**Acq. Method**	pesticide std 12.11.2024

Sample Chromatogram

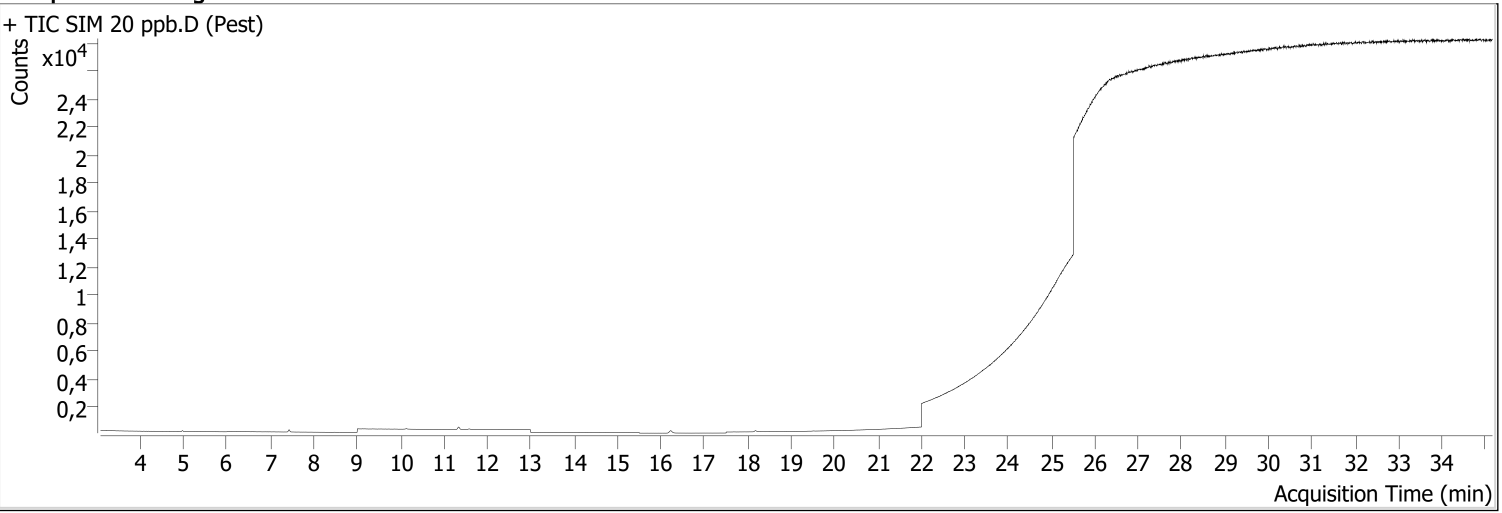

Compound	Transition	RT	Resp.	Final Conc	Units
Lindane	219,0	4,982	12	19,0400	ng/ml
Hexachlorobenzene	284,0	7,434	232	19,2419	ng/ml
Aldrine	293,0	11,344	84	18,6841	ng/ml
Chlorpyrifos	314,0	11,584	36	18,3902	ng/ml
Endosulfan II	339,0	14,716	25	19,6624	ng/ml
4,4-DDE	318,0	16,233	251	18,3012	ng/ml
4,4-DDD	237,0	18,172	97	19,0677	ng/ml
4,4-DDT	237,0	19,526	12	13,5304	ng/ml

Lindane

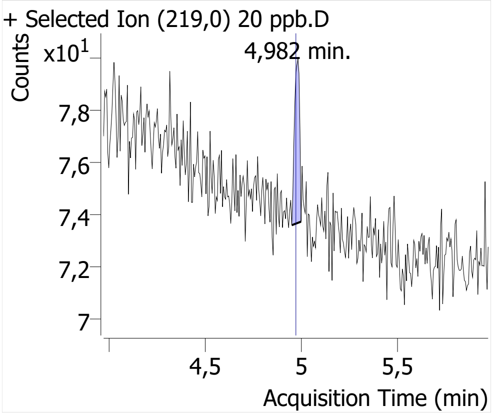

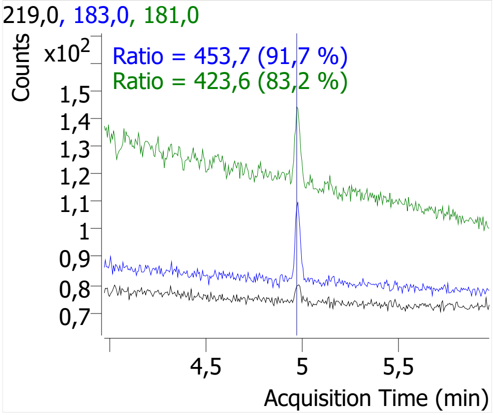

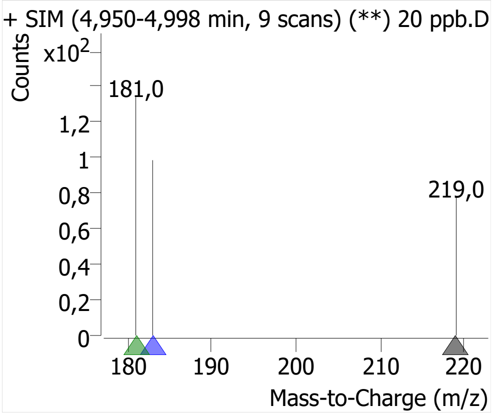

Hexachlorobenzene

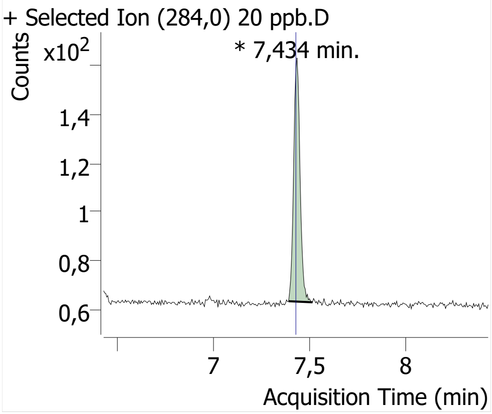

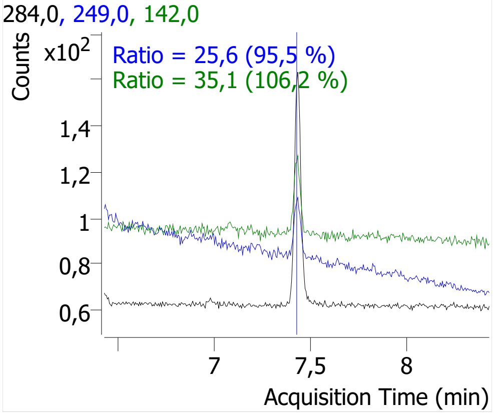

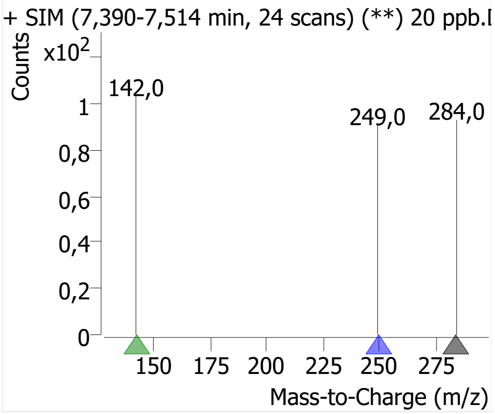

Aldrine

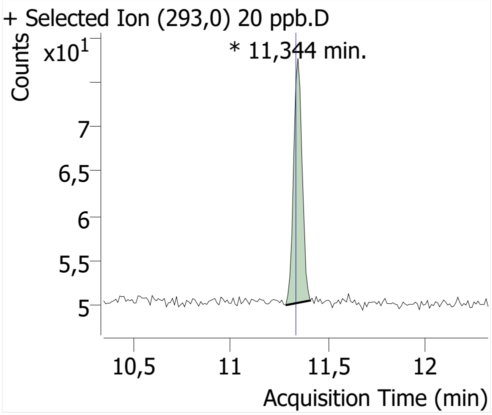

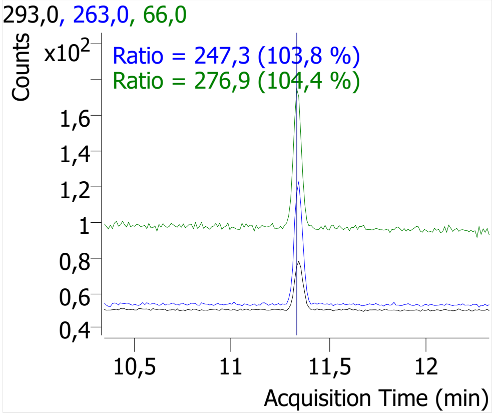

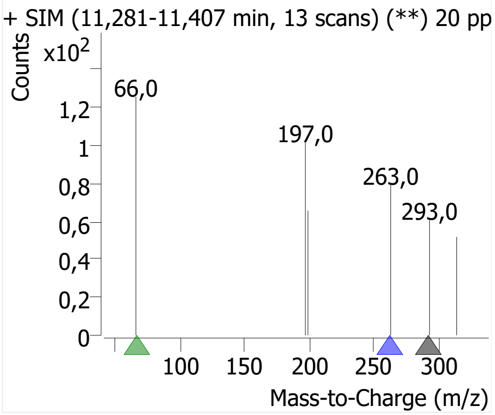

Chlorpyrifos

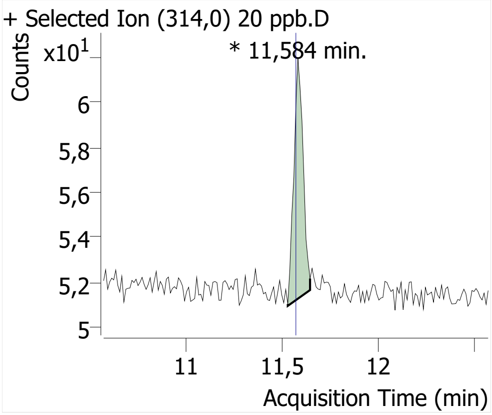

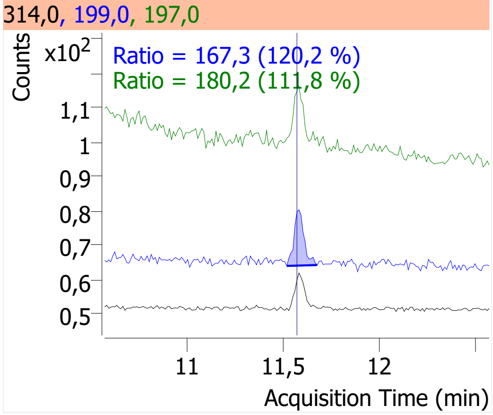

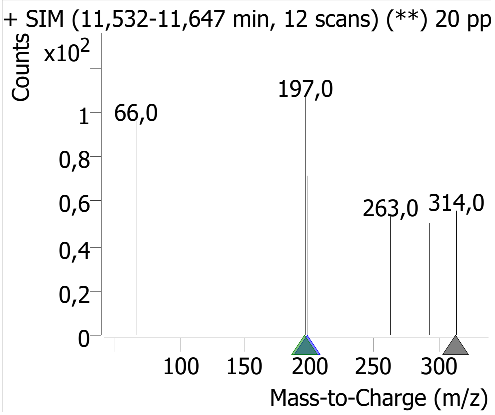

Endosulfan II

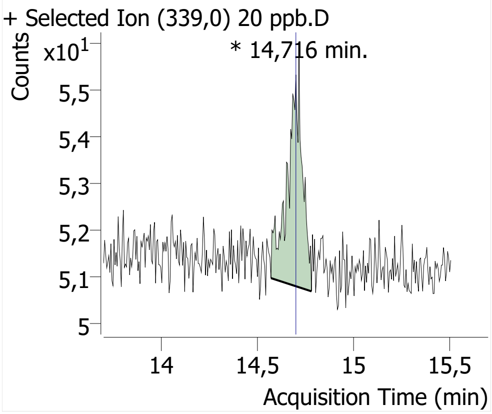

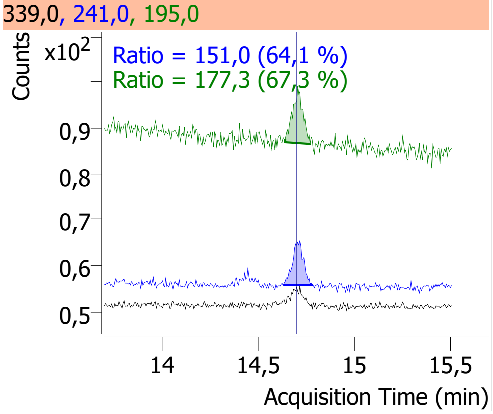

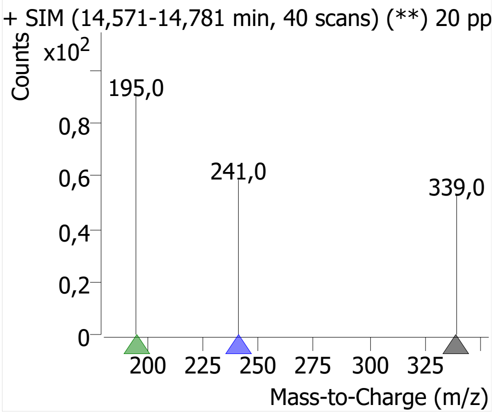

4,4-DDE

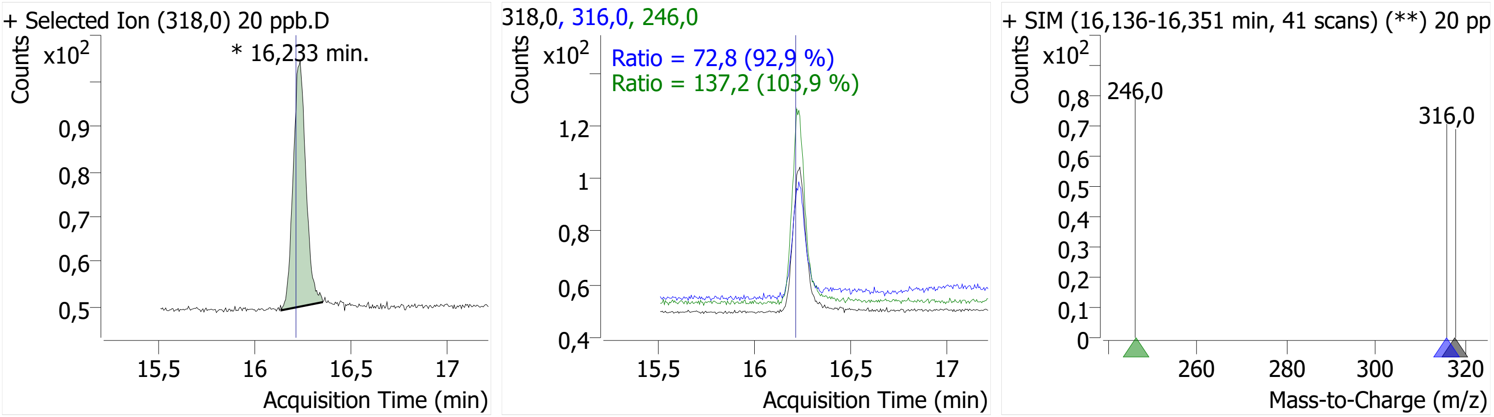

4,4-DDD

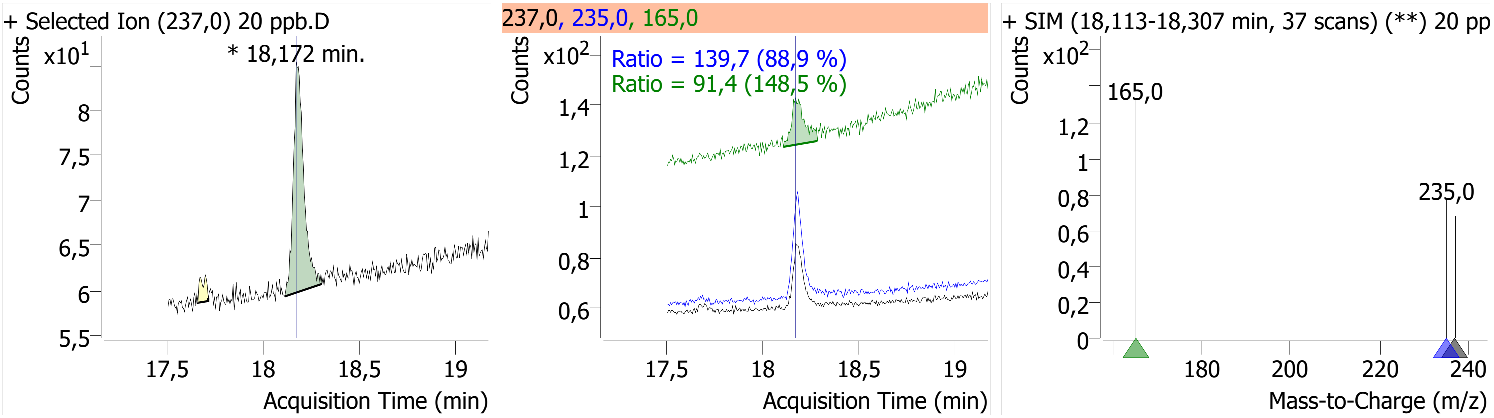

4,4-DDT

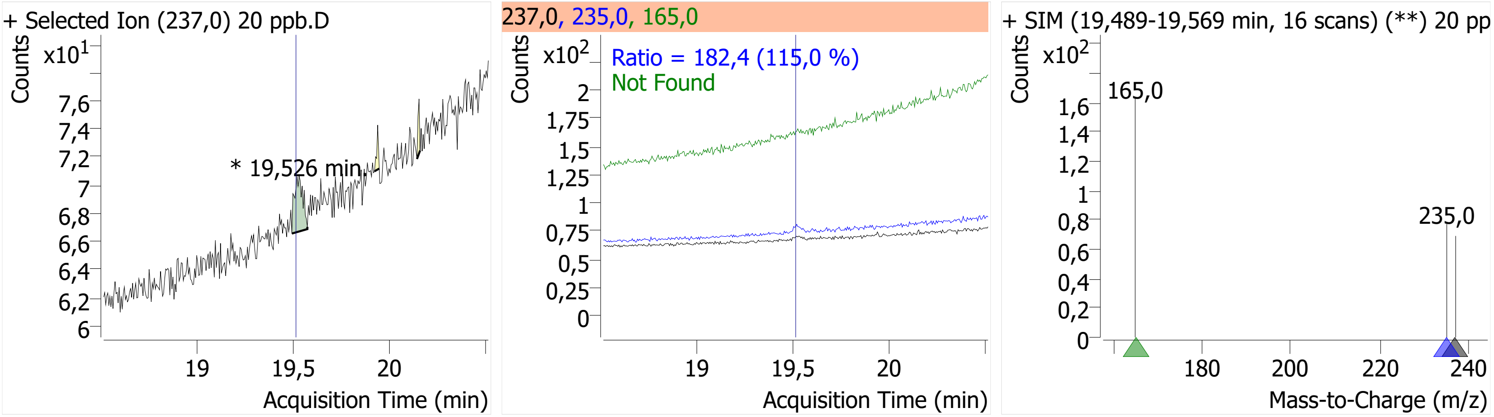

Quantitative Analysis Complete Report

**Batch Path**	C:\Users\USER\OneDrive\Desktop\JU_Pesticide\MA\MA\QuantResults\Mohammed_Pest.batch.bin		
**Analysis Time**	13 Dec 2024 11:34	**Analyst Name**	DESKTOP-MRRPPC4\USER
**Report Time**	13 Dec 2024 11:37:24	**Reporter Name**	DESKTOP-MRRPPC4\USER
**Last Calib Update**	13 Dec 2024 11:34	**Batch State**	Processed
**Quant Batch Version**	10.0	**Quant Report Version**	10.0

**Acq. Time**	12 Nov 2024 19:19	**Data File**	40 ppb.D
**Sample Type**	Cal	**Sample Name**	Pest
**Dilution**	1	**Acq. Method**	pesticide std 12.11.2024

Sample Chromatogram

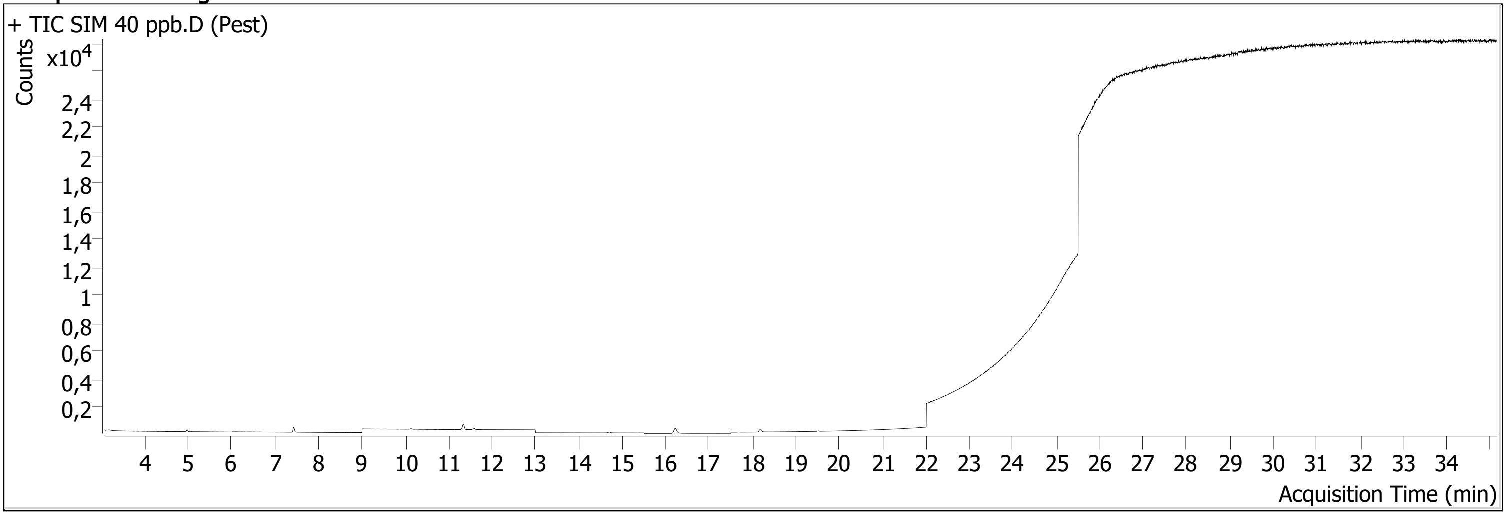

Compound	Transition	RT	Resp.	Final Conc	Units
Lindane	219,0	4,982	27	41,4877	ng/ml
Hexachlorobenzene	284,0	7,428	518	40,6516	ng/ml
Aldrine	293,0	11,344	200	40,6013	ng/ml
Chlorpyrifos	314,0	11,595	83	39,5701	ng/ml
Endosulfan II	339,0	14,700	45	39,6360	ng/ml
4,4-DDE	318,0	16,222	560	39,2885	ng/ml
4,4-DDD	237,0	18,178	197	37,5003	ng/ml
4,4-DDT	237,0	19,526	32	38,4243	ng/ml

Lindane

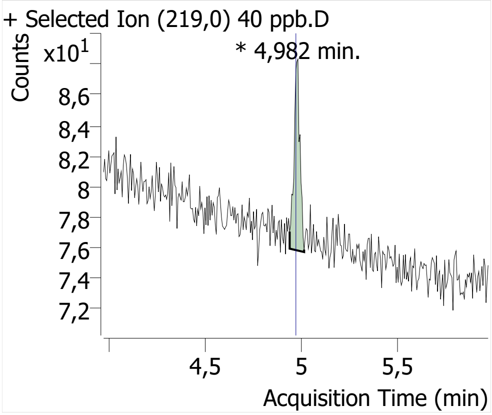

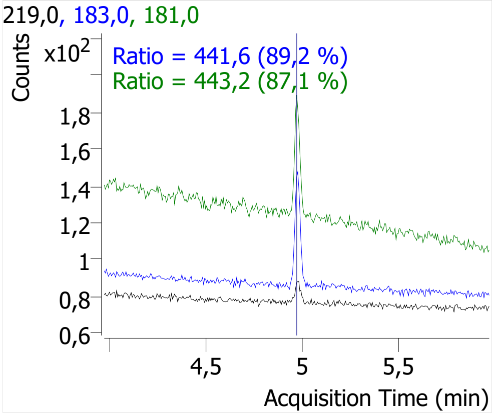

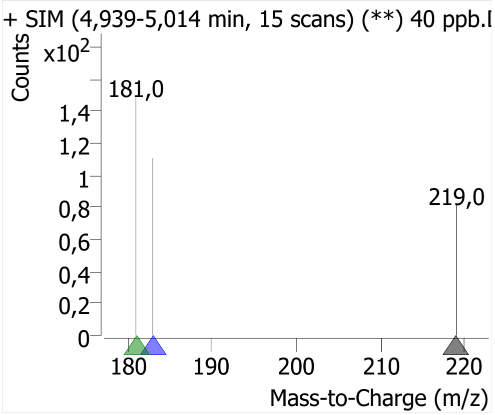

Hexachlorobenzene

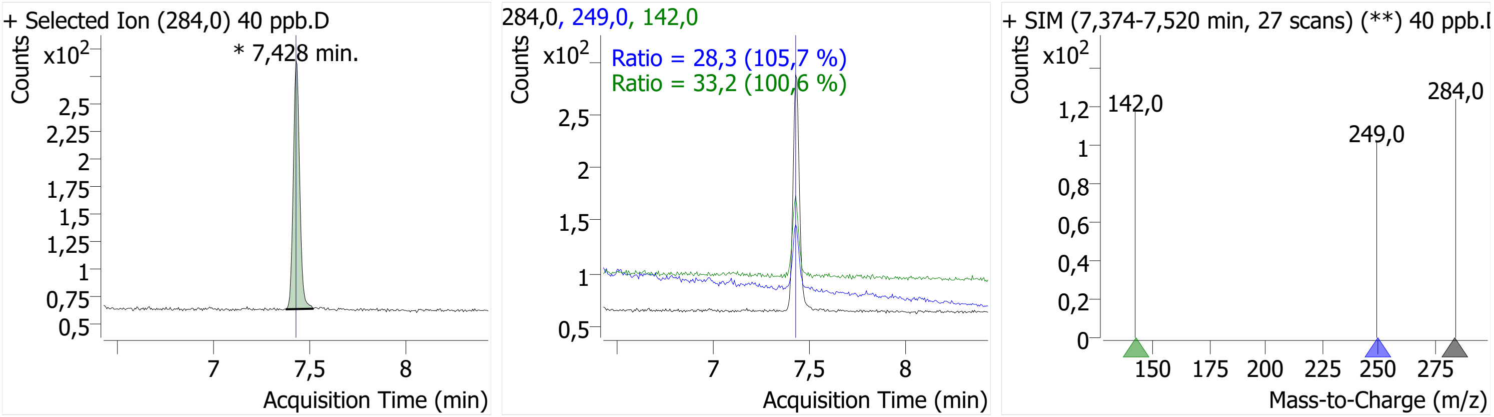

Aldrine

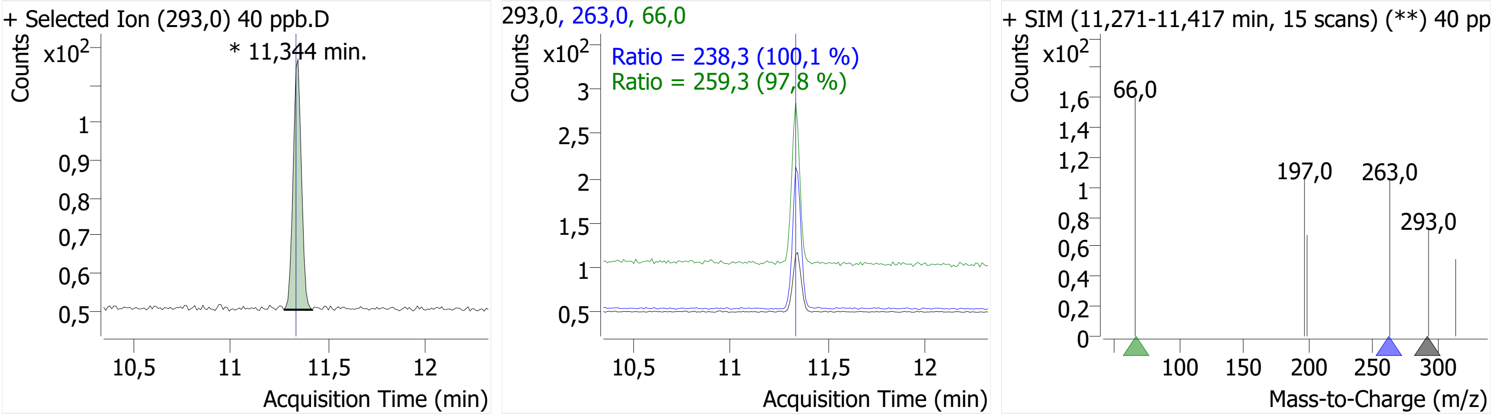

Chlorpyrifos

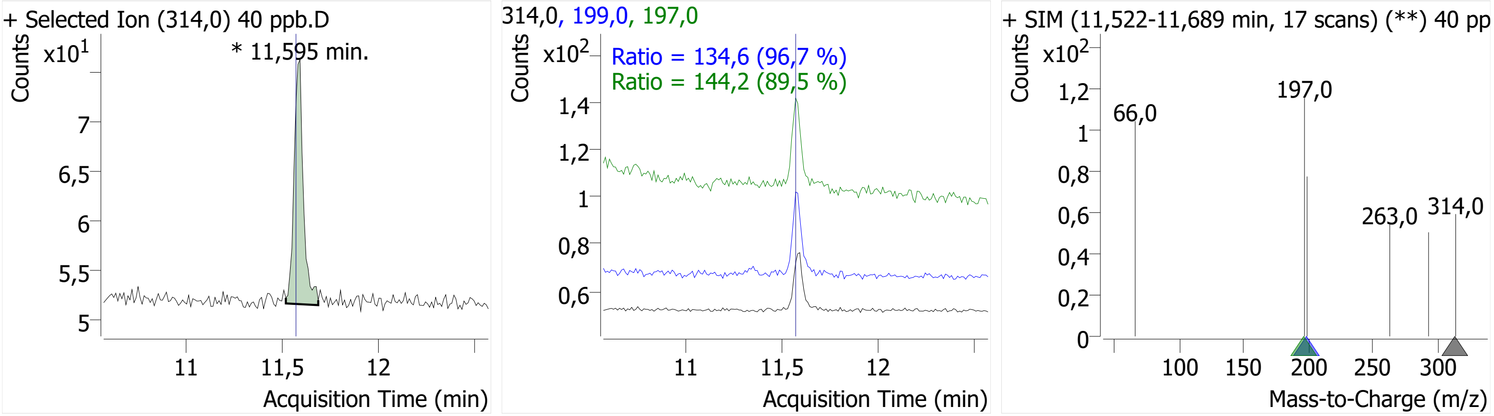

Endosulfan II

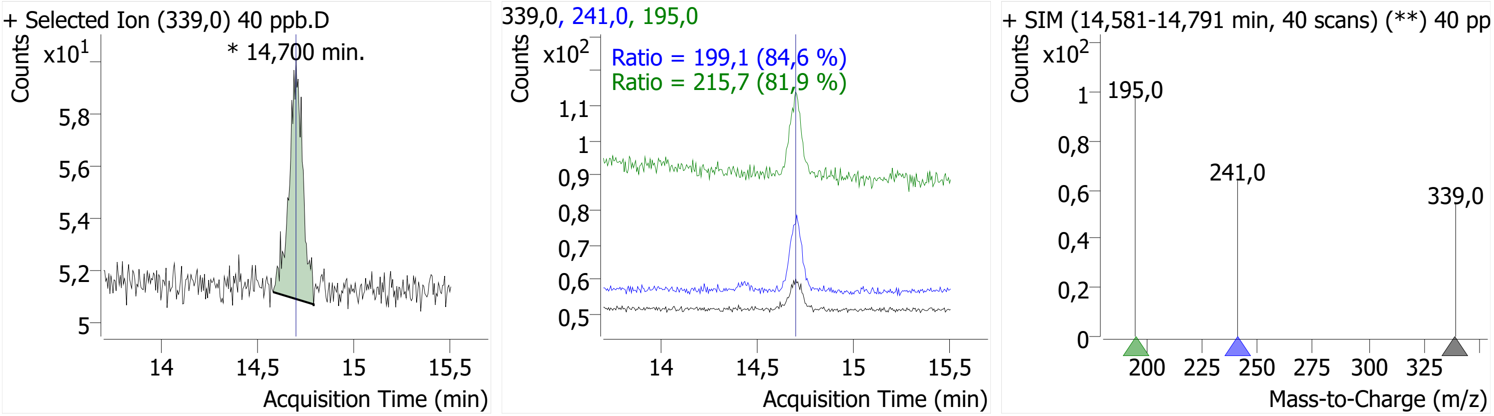

4,4-DDE

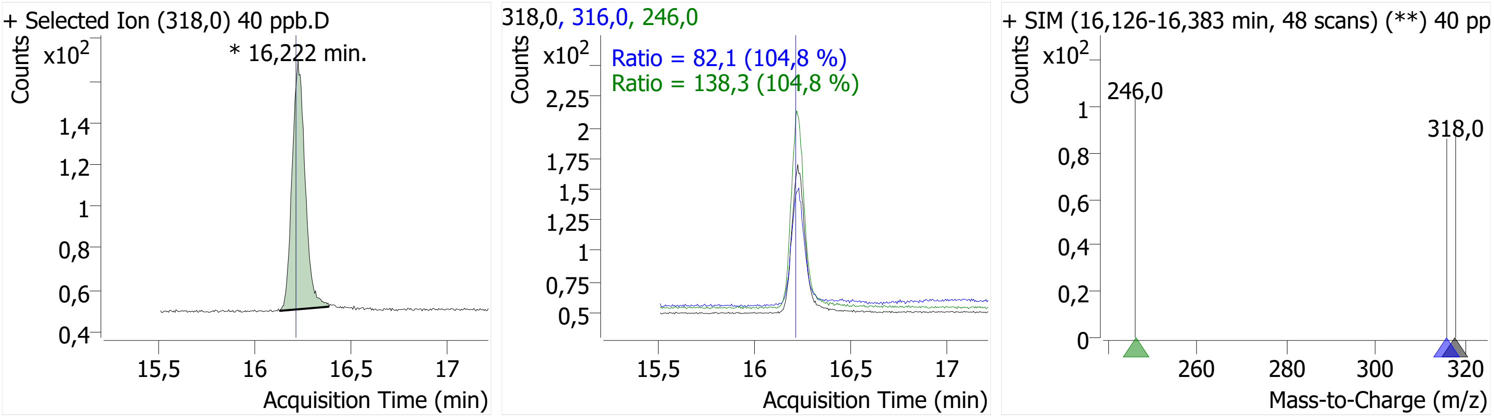

4,4-DDD

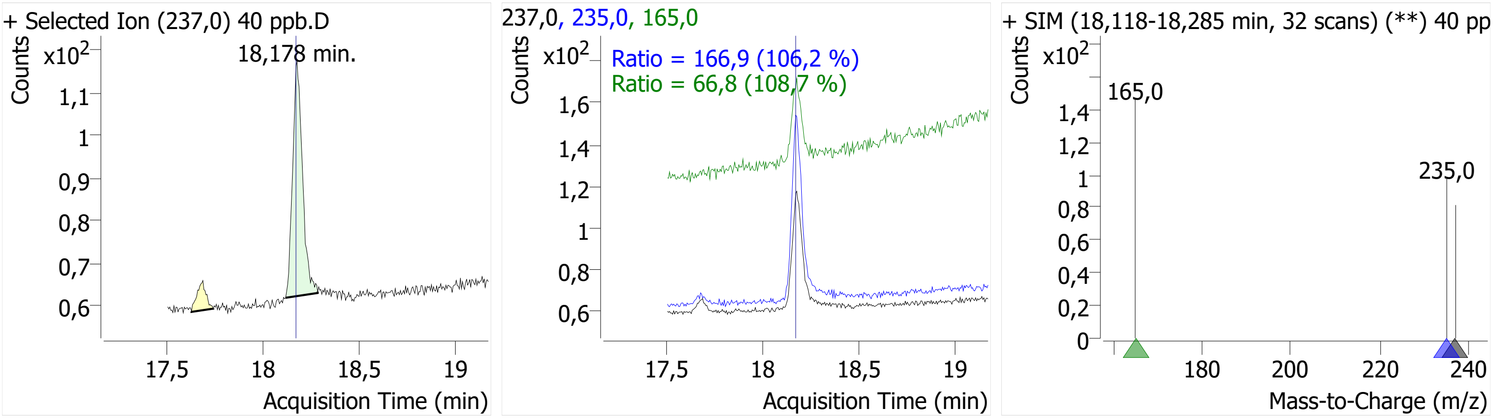

4,4-DDT

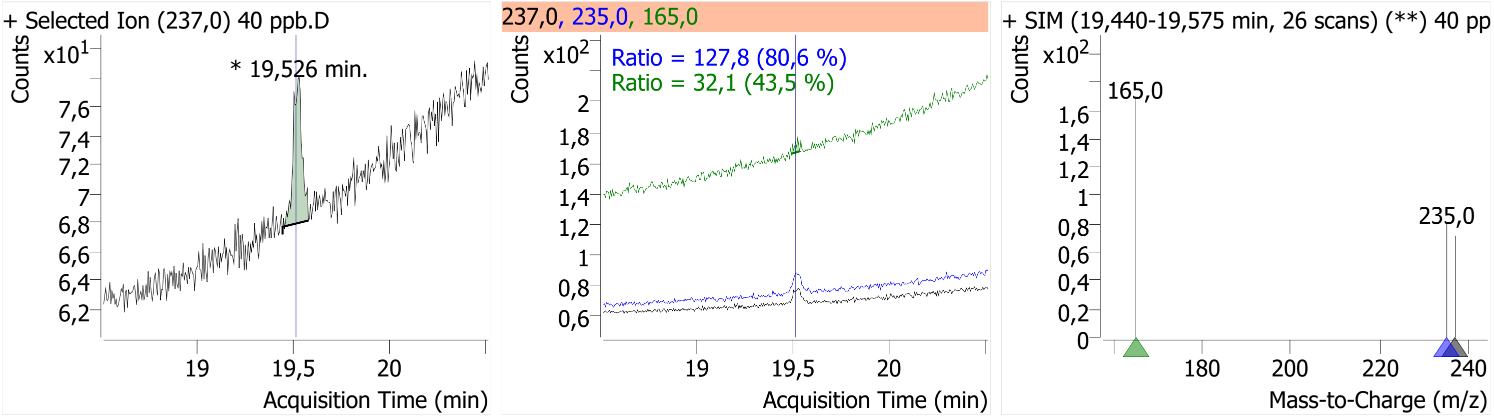

Quantitative Analysis Complete Report

**Batch Path**	C:\Users\USER\OneDrive\Desktop\JU_Pesticide\MA\MA\QuantResults\Mohammed_Pest.batch.bin		
**Analysis Time**	13 Dec 2024 11:34	**Analyst Name**	DESKTOP-MRRPPC4\USER
**Report Time**	13 Dec 2024 11:37:25	**Reporter Name**	DESKTOP-MRRPPC4\USER
**Last Calib Update**	13 Dec 2024 11:34	**Batch State**	Processed
**Quant Batch Version**	10.0	**Quant Report Version**	10.0

**Acq. Time**	12 Nov 2024 20:00	**Data File**	50 ppb.D
**Sample Type**	Cal	**Sample Name**	Pest
**Dilution**	1	**Acq. Method**	pesticide std 12.11.2024

Sample Chromatogram

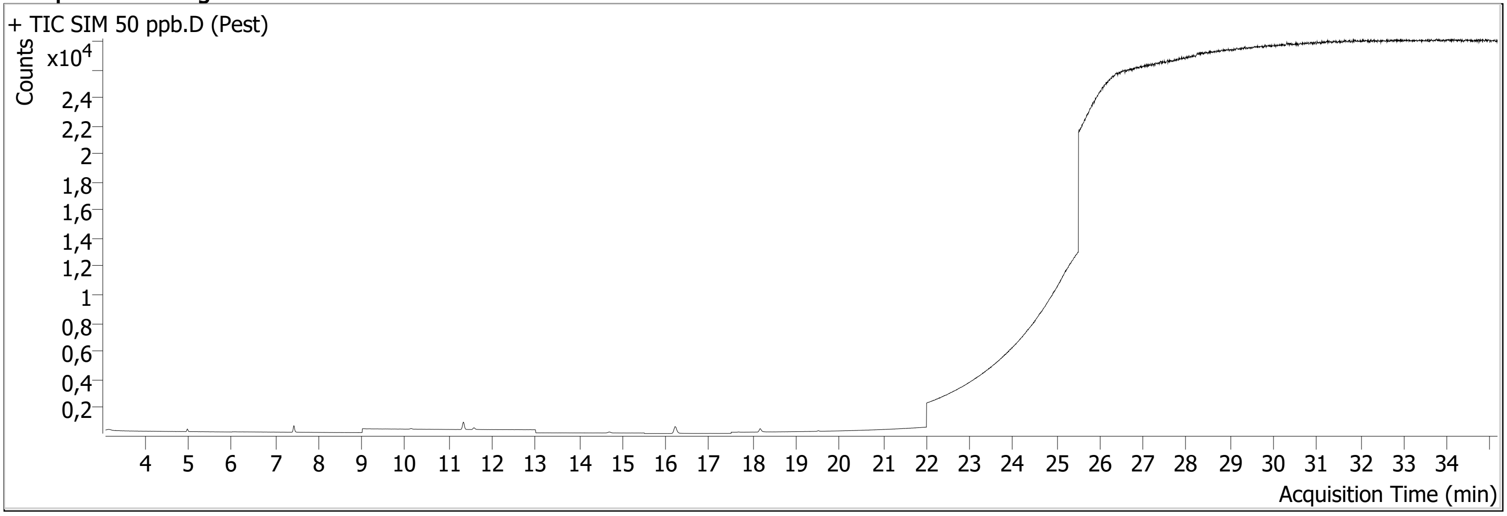

Compound	Transition	RT	Resp.	Final Conc	Units
Lindane	219,0	4,976	32	48,7511	ng/ml
Hexachlorobenzene	284,0	7,428	669	51,9929	ng/ml
Aldrine	293,0	11,334	252	50,5119	ng/ml
Chlorpyrifos	314,0	11,584	107	50,3146	ng/ml
Endosulfan II	339,0	14,695	55	50,2322	ng/ml
4,4-DDE	318,0	16,217	720	50,1795	ng/ml
4,4-DDD	237,0	18,178	267	50,2745	ng/ml
4,4-DDT	237,0	19,516	41	49,3541	ng/ml

Lindane

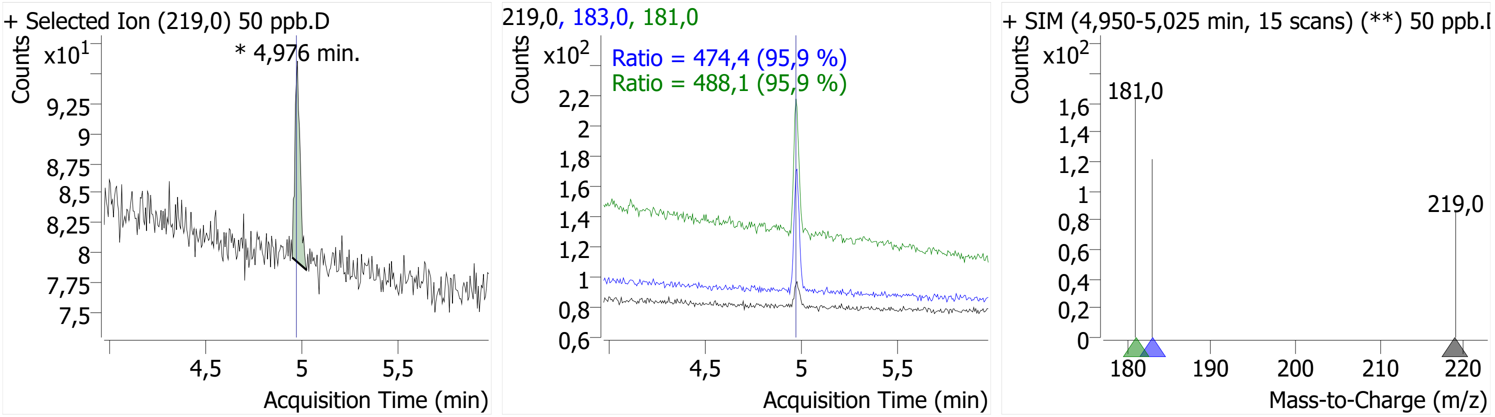

Hexachlorobenzene

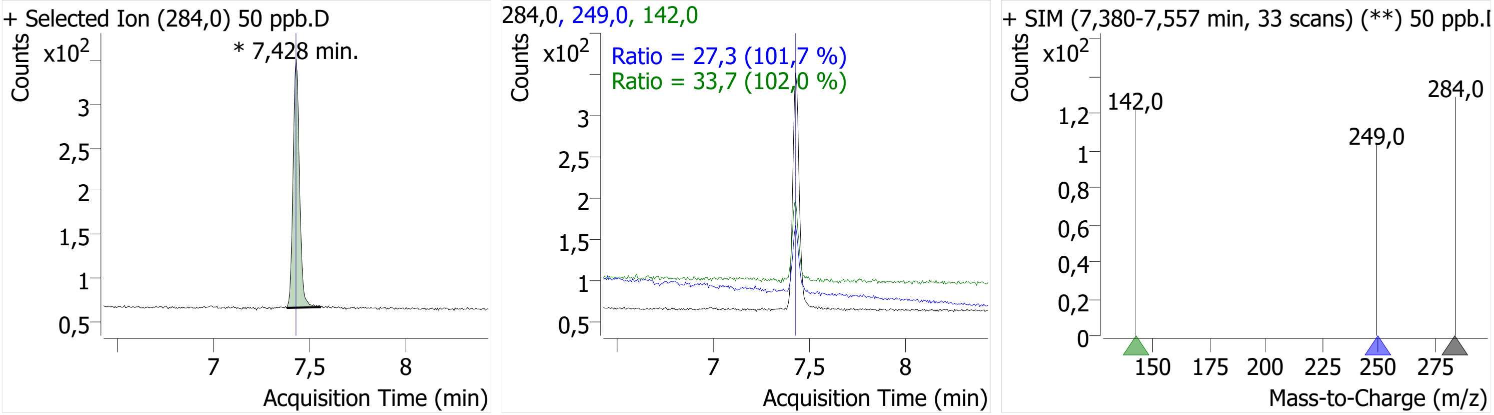

Aldrine

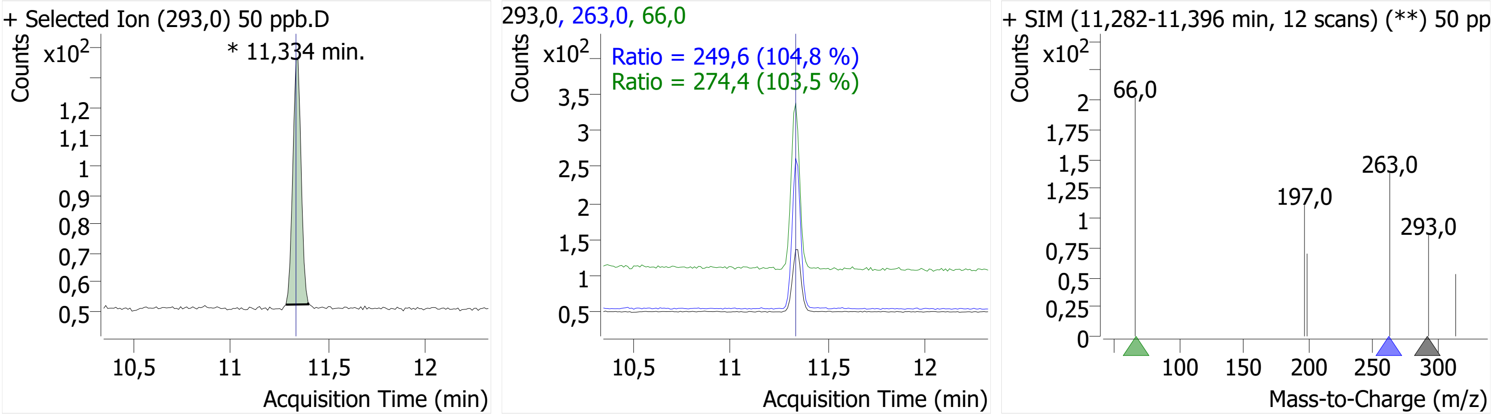

Chlorpyrifos

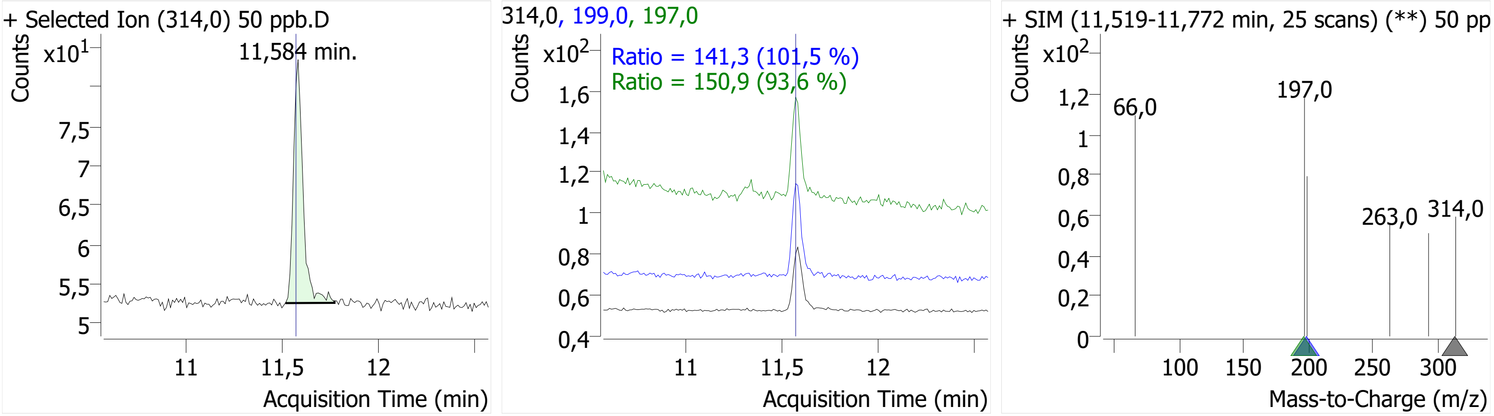

Endosulfan II

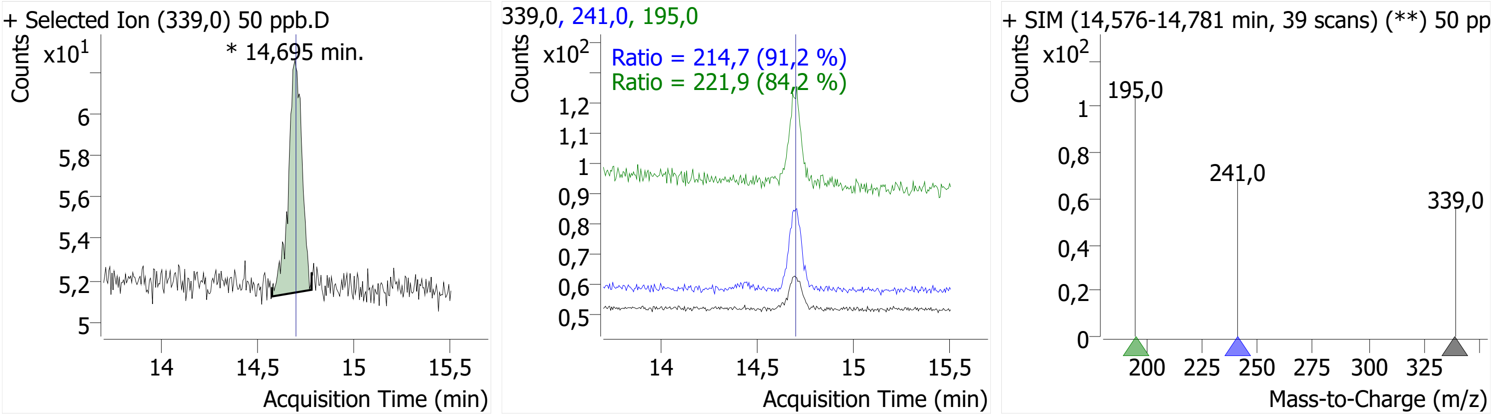

4,4-DDE

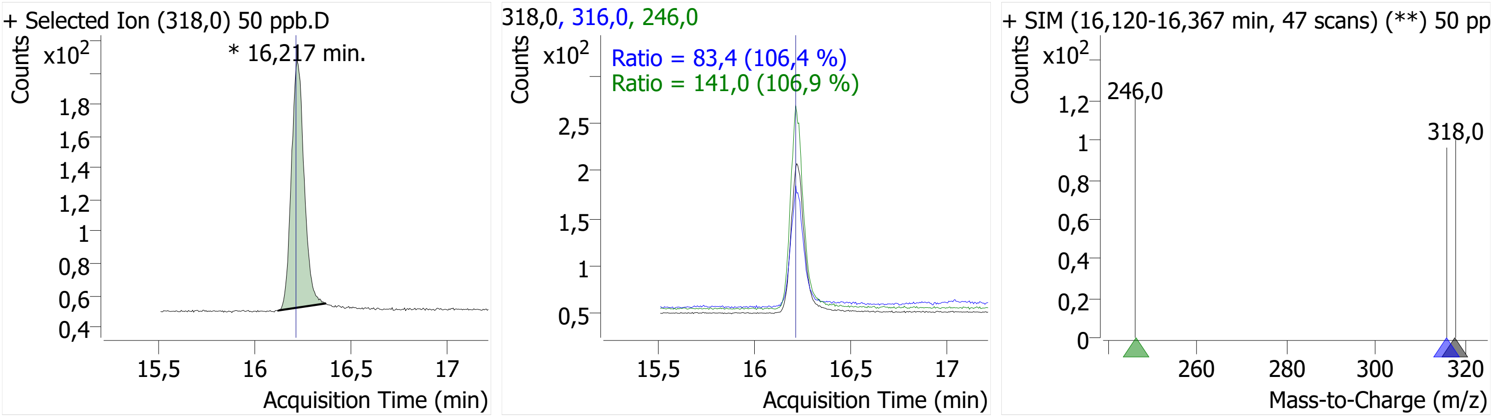

4,4-DDD

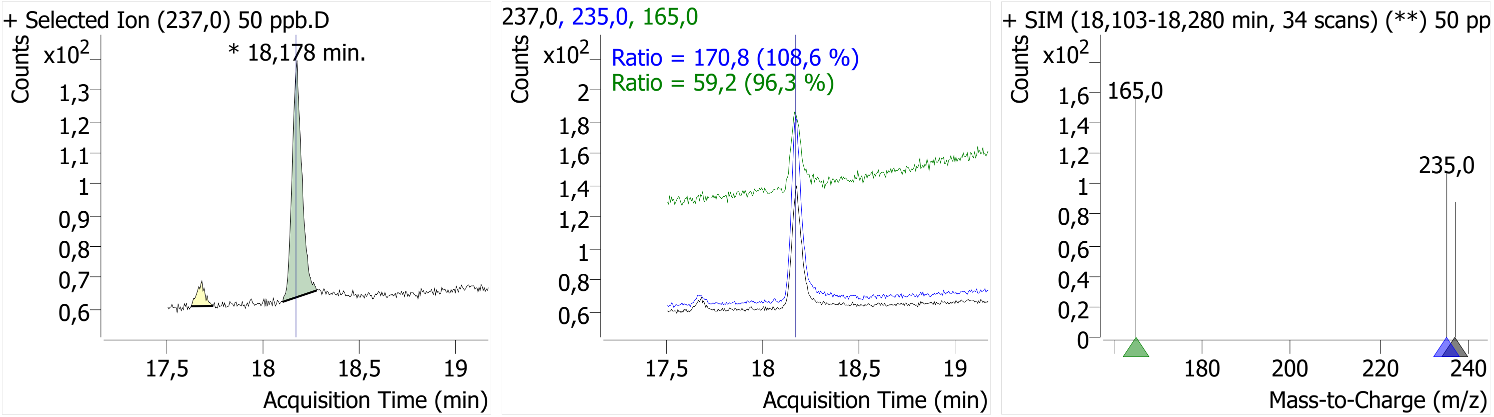

4,4-DDT

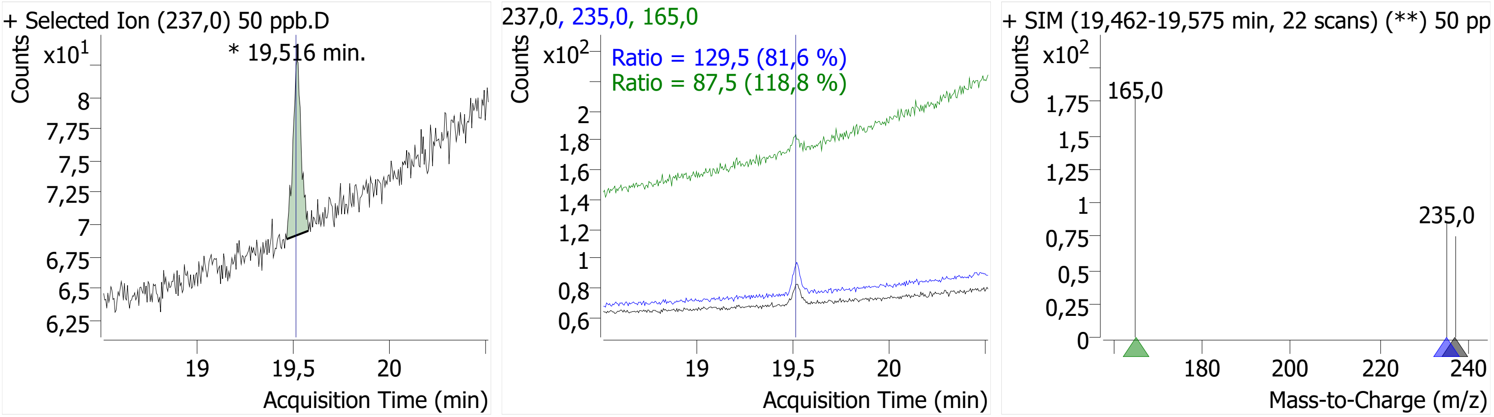

Quantitative Analysis Complete Report

**Batch Path**	C:\Users\USER\OneDrive\Desktop\JU_Pesticide\MA\MA\QuantResults\Mohammed_Pest.batch.bin		
**Analysis Time**	13 Dec 2024 11:34	**Analyst Name**	DESKTOP-MRRPPC4\USER
**Report Time**	13 Dec 2024 11:37:26	**Reporter Name**	DESKTOP-MRRPPC4\USER
**Last Calib Update**	13 Dec 2024 11:34	**Batch State**	Processed
**Quant Batch Version**	10.0	**Quant Report Version**	10.0

**Acq. Time**	12 Nov 2024 20:41	**Data File**	100 ppb.D
**Sample Type**	Cal	**Sample Name**	Pest
**Dilution**	1	**Acq. Method**	pesticide std 12.11.2024

Sample Chromatogram

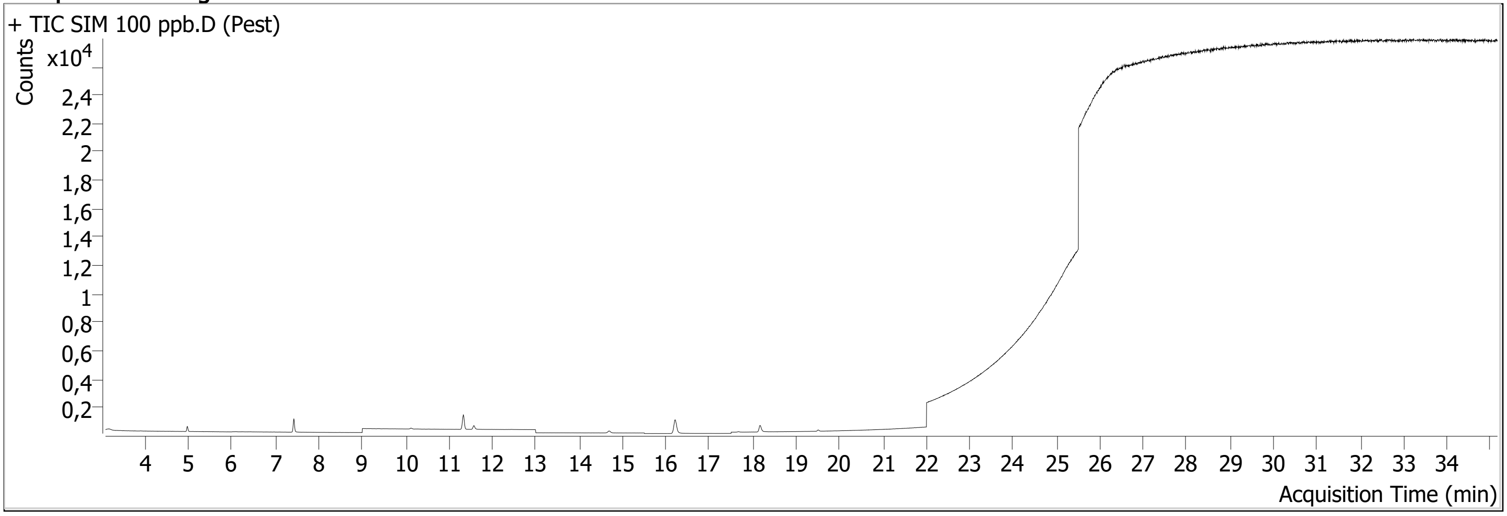

Compound	Transition	RT	Resp.	Final Conc	Units
Lindane	219,0	4,976	67	100,2960	ng/ml
Hexachlorobenzene	284,0	7,428	1298	99,1728	ng/ml
Aldrine	293,0	11,334	511	99,9468	ng/ml
Chlorpyrifos	314,0	11,584	217	100,0974	ng/ml
Endosulfan II	339,0	14,689	103	99,5096	ng/ml
4,4-DDE	318,0	16,217	1459	100,4760	ng/ml
4,4-DDD	237,0	18,172	543	100,8968	ng/ml
4,4-DDT	237,0	19,510	83	100,5583	ng/ml

Lindane

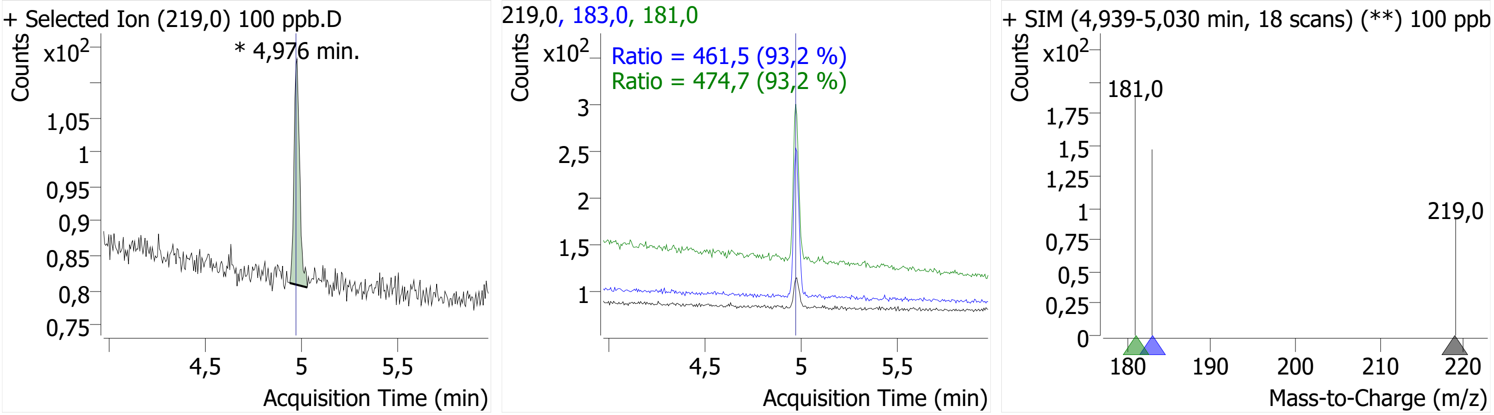

Hexachlorobenzene

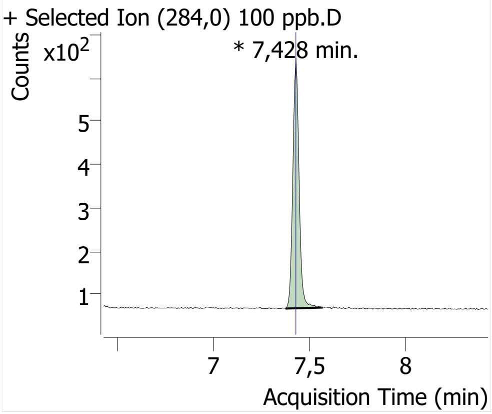

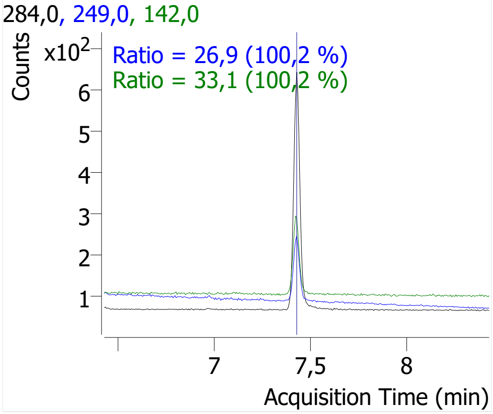

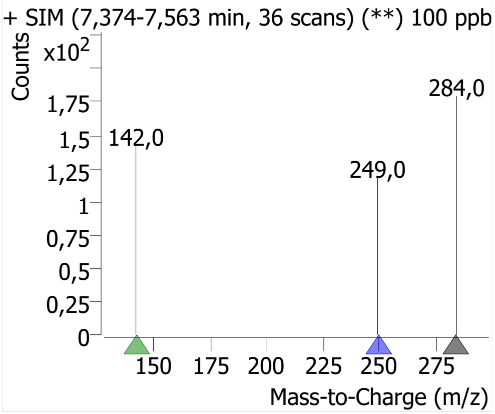

Aldrine

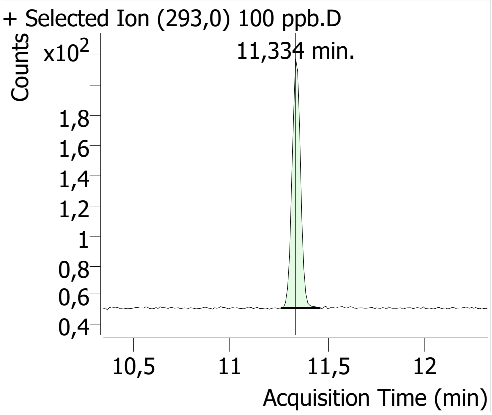

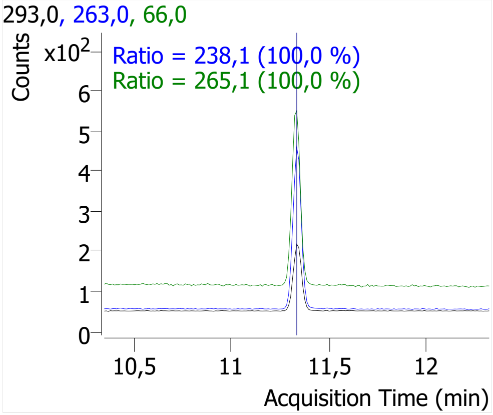

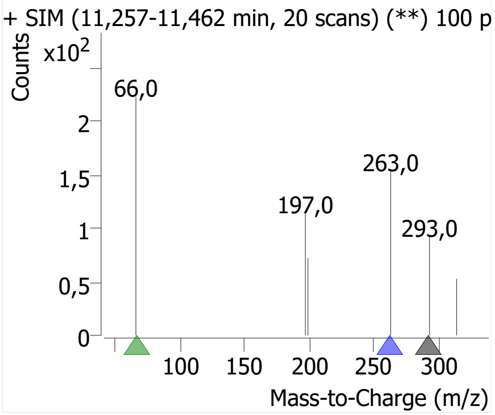

Chlorpyrifos

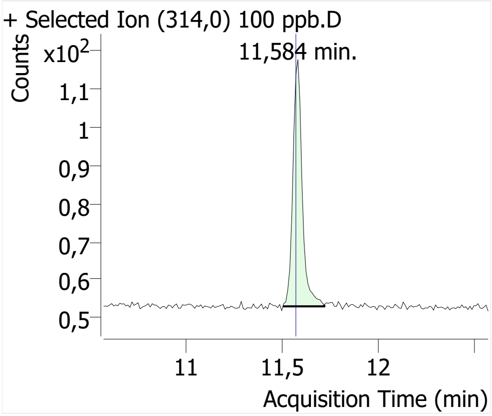

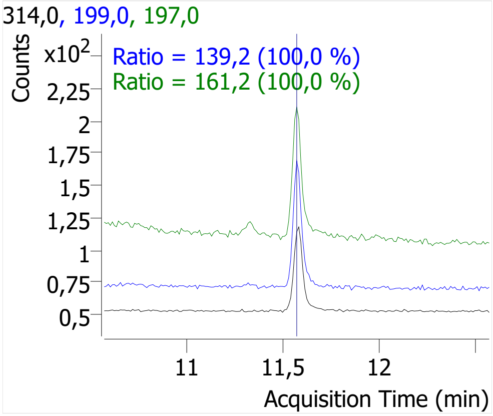

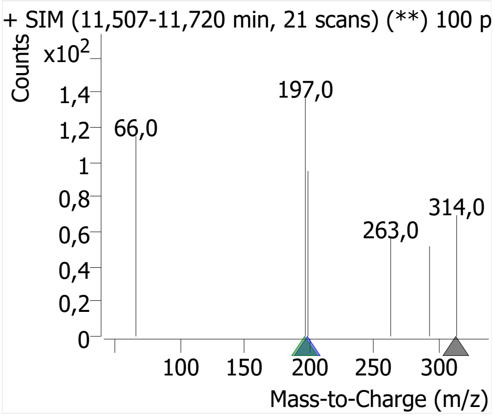

Endosulfan II

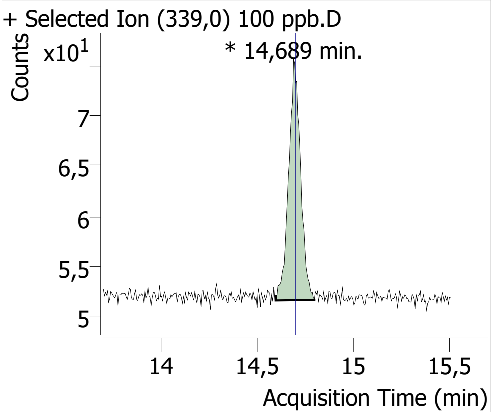

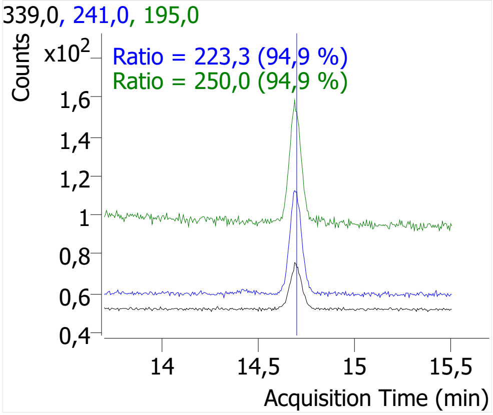

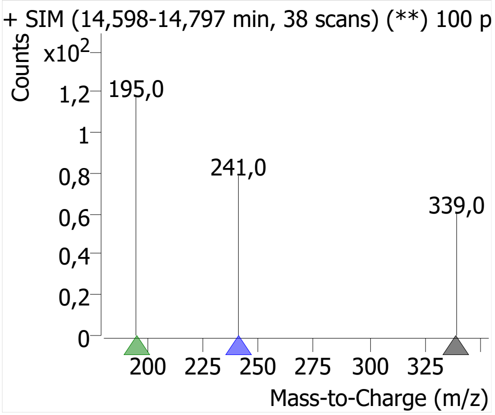

Agilent Trusted Answers

4,4-DDE

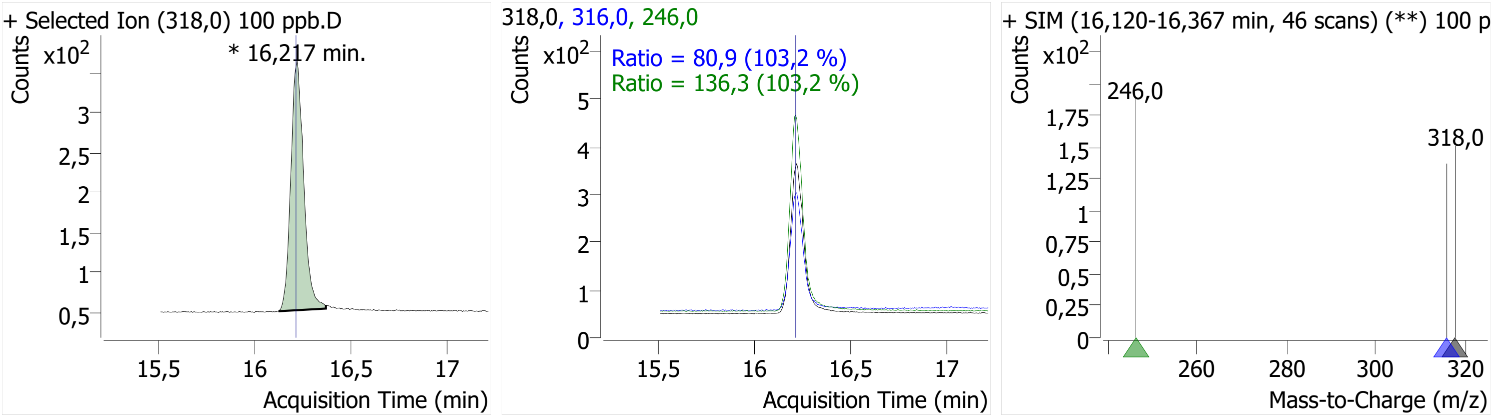

4,4-DDD

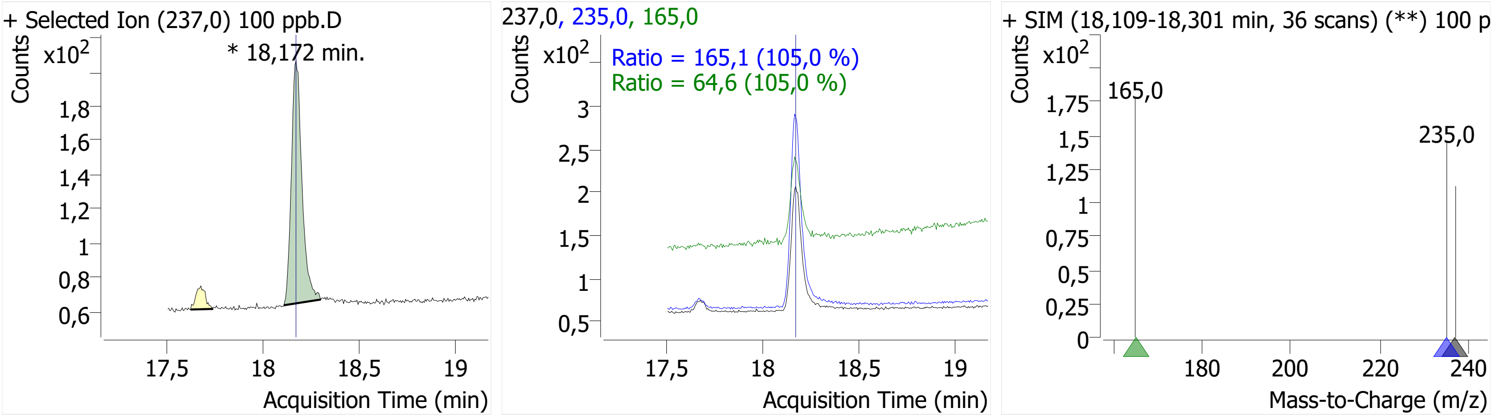

4,4-DDT

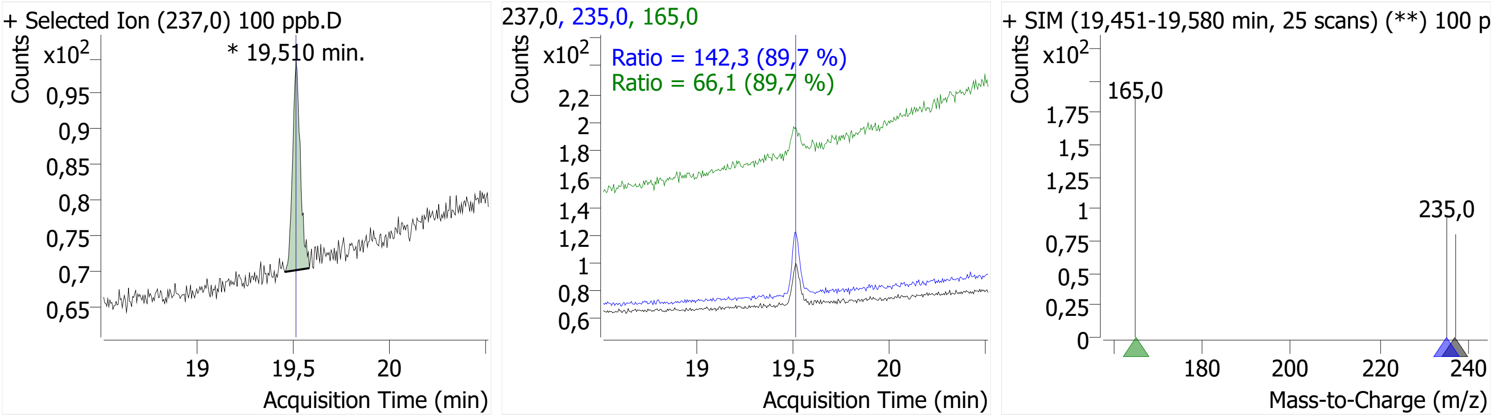

 Generated at 11:37 on 13 Dec 2024

Quantitative Analysis Complete Report

**Batch Path**	C:\Users\USER\OneDrive\Desktop\JU_Pesticide\MA\MA\QuantResults\Mohammed_Pest.batch.bin		
**Analysis Time**	13 Dec 2024 11:34	**Analyst Name**	DESKTOP-MRRPPC4\USER
**Report Time**	13 Dec 2024 11:37:27	**Reporter Name**	DESKTOP-MRRPPC4\USER
**Last Calib Update**	13 Dec 2024 11:34	**Batch State**	Processed
**Quant Batch Version**	10.0	**Quant Report Version**	10.0

**Acq. Time**	12 Nov 2024 21:21	**Data File**	30-1ppb.D
**Sample Type**	Cal	**Sample Name**	Pest
**Dilution**	1	**Acq. Method**	pesticide std 12.11.2024

Sample Chromatogram

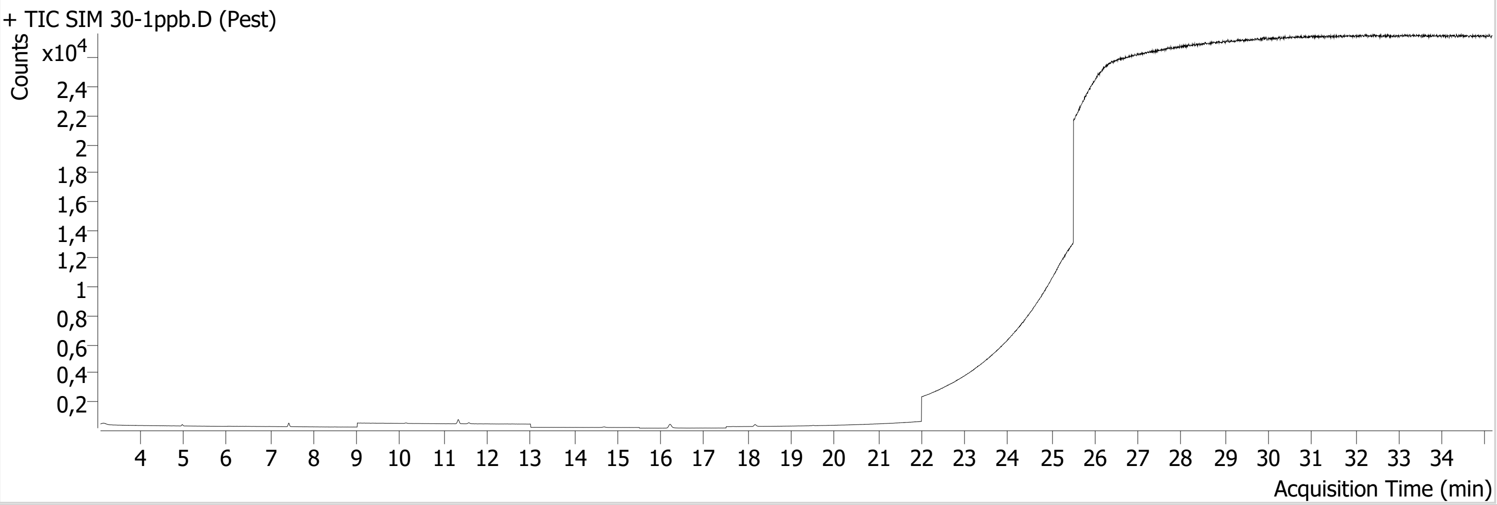

Compound	Transition	RT	Resp.	Final Conc	Units
Lindane	219,0	4,976	19	29,5022	ng/ml
Hexachlorobenzene	284,0	7,428	365	29,1991	ng/ml
Aldrine	293,0	11,334	140	29,2307	ng/ml
Chlorpyrifos	314,0	11,584	63	30,6936	ng/ml
Endosulfan II	339,0	14,695	38	32,1805	ng/ml
4,4-DDE	318,0	16,211	414	29,3769	ng/ml
4,4-DDD	237,0	18,173	155	29,7876	ng/ml
4,4-DDT	237,0	19,505	27	31,1950	ng/ml

Lindane

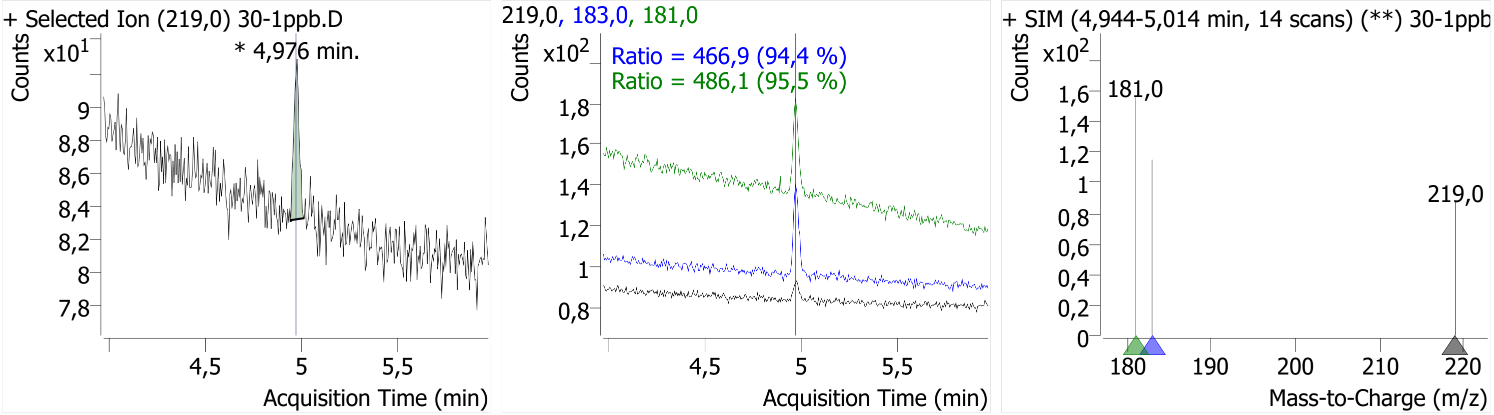

Hexachlorobenzene

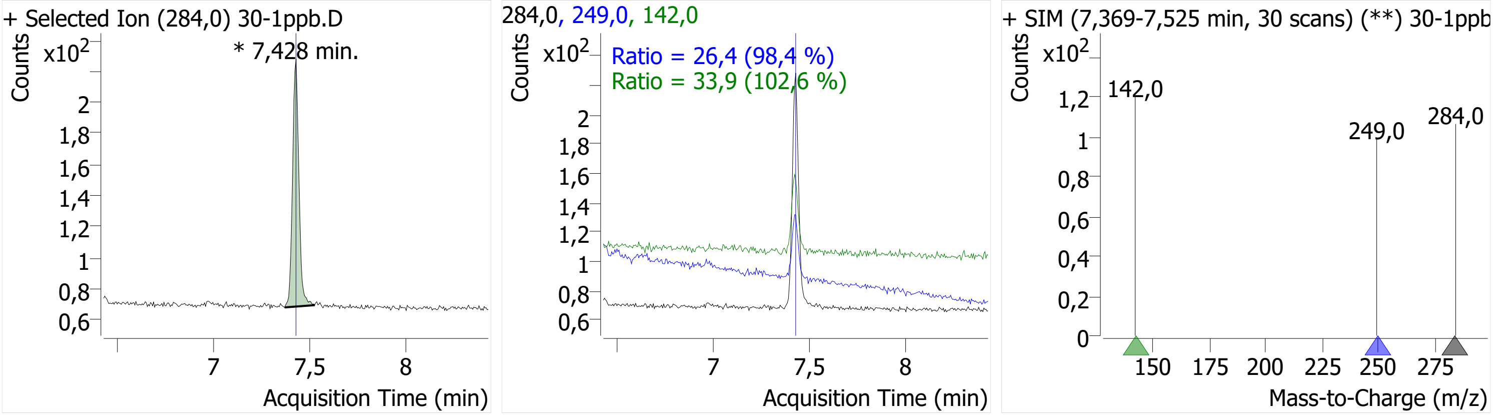

Aldrine

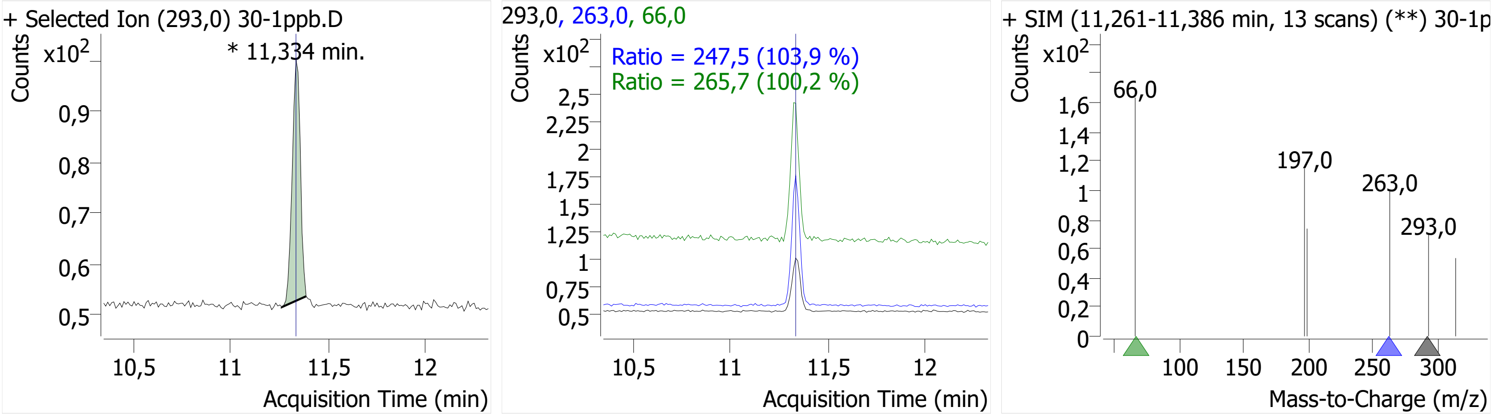

Chlorpyrifos

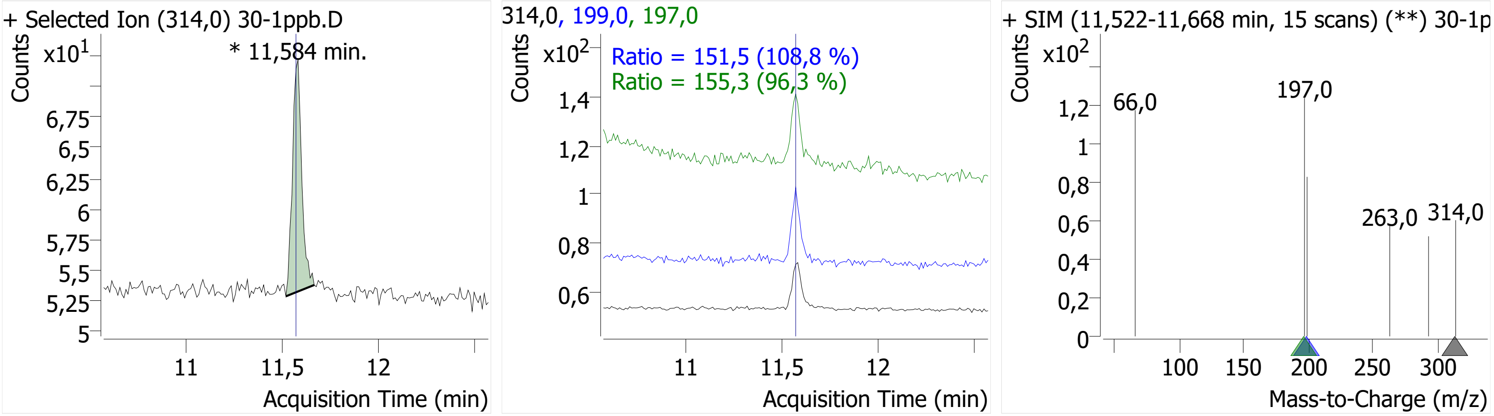

Endosulfan II

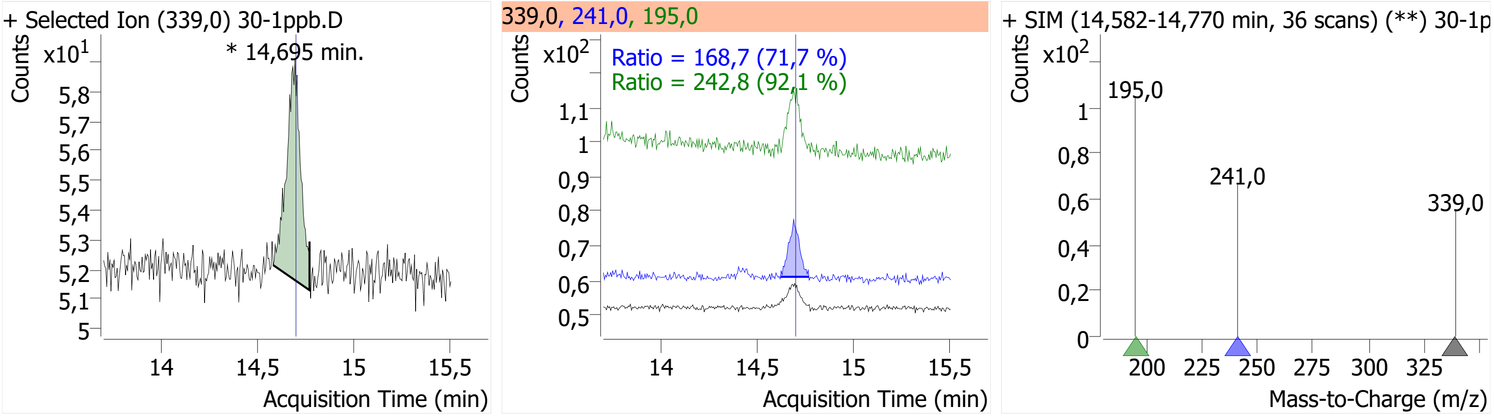

4,4-DDE

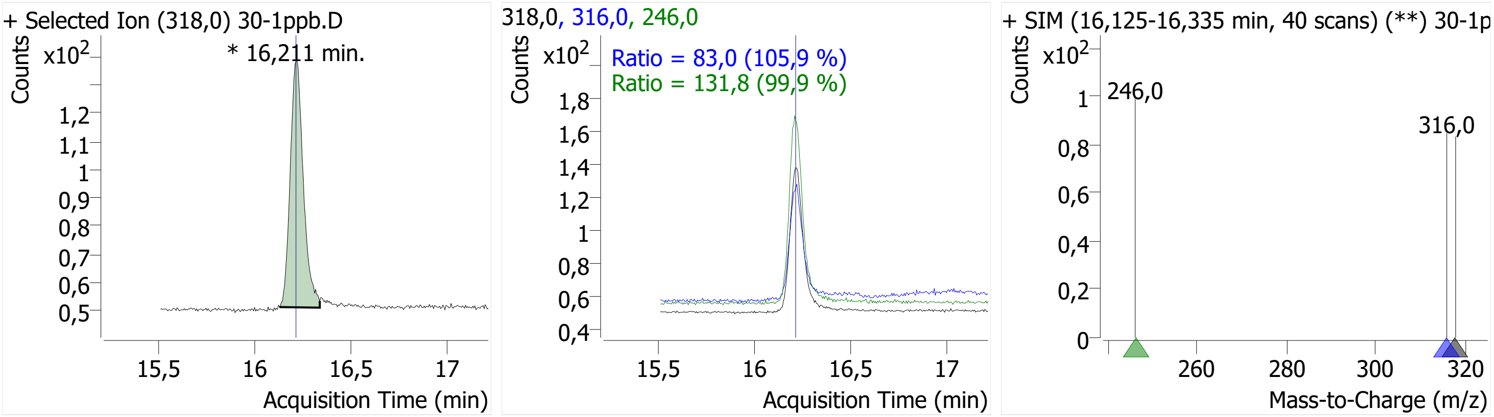

4,4-DDD

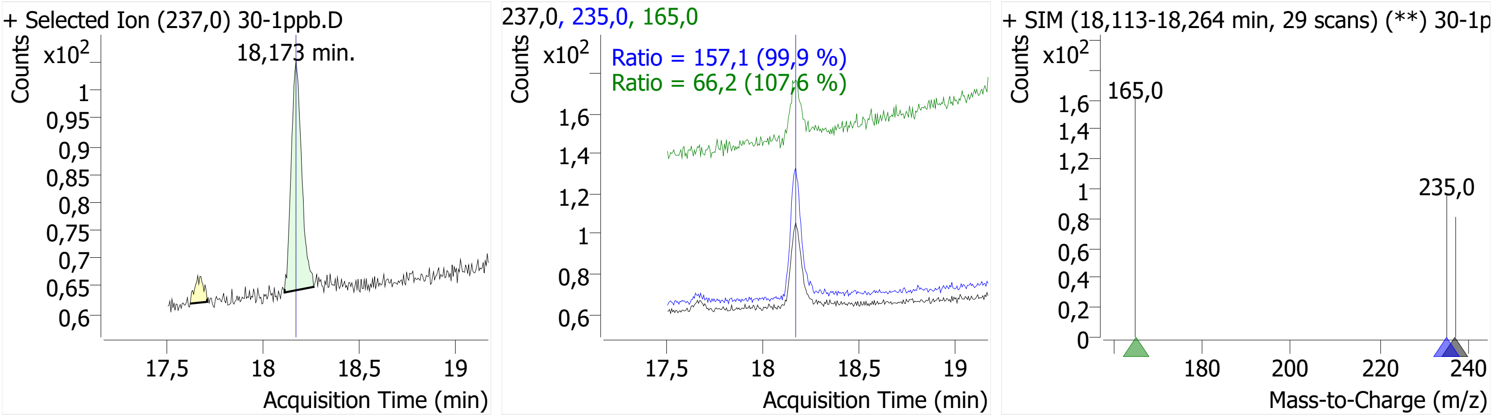

4,4-DDT

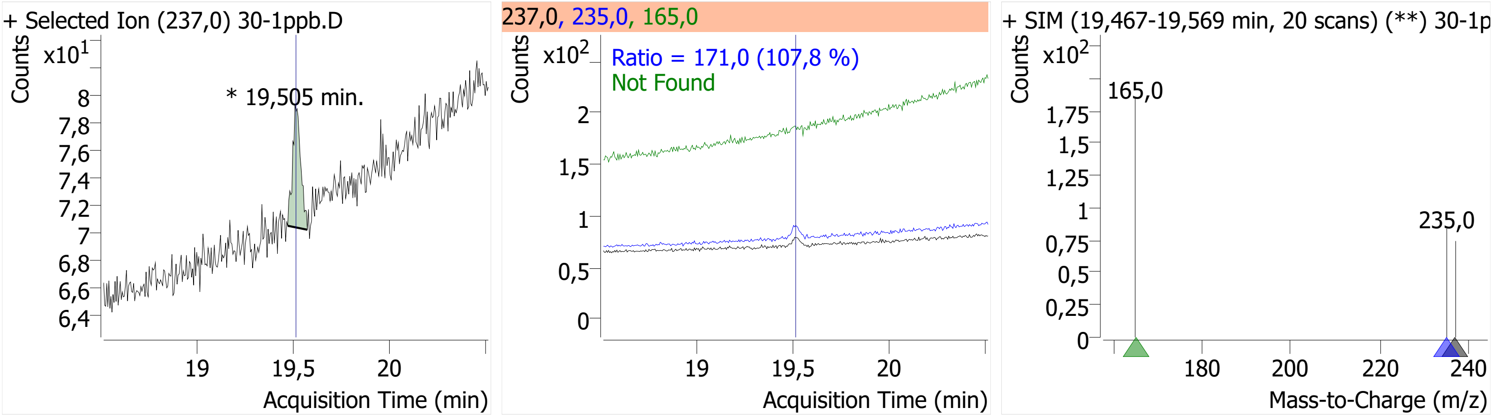

**Batch Path**	C:\Users\USER\OneDrive\Desktop\JU_Pesticide\MA\MA\QuantResults\Mohammed_Pest.batch.bin		
**Analysis Time**	13 Dec 2024 11:34	**Analyst Name**	DESKTOP-MRRPPC4\USER
**Report Time**	13 Dec 2024 11:37:28	**Reporter Name**	DESKTOP-MRRPPC4\USER
**Last Calib Update**	13 Dec 2024 11:34	**Batch State**	Processed
**Quant Batch Version**	10.0	**Quant Report Version**	10.0

**Acq. Time**	12 Nov 2024 22:02	**Data File**	S2 DWO.D
**Sample Type**	Sample	**Sample Name**	Pest
**Dilution**	1	**Acq. Method**	pesticide std 12.11.2024

Sample Chromatogram

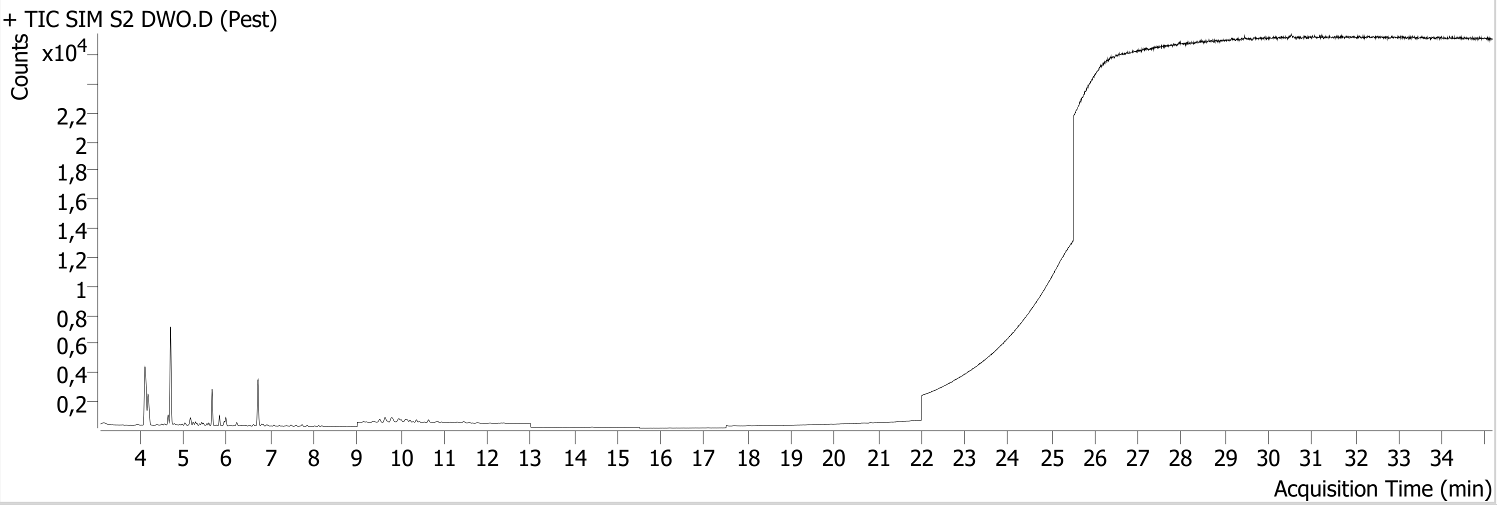

Compound	Transition	RT	Resp.	Final Conc	Units
Lindane	219,0	5,159	0	ND	ng/ml
Hexachlorobenzene	284,0	7,342	0	ND	ng/ml
Aldrine	293,0	11,365	0	ND	ng/ml
Chlorpyrifos	314,0	11,553	0	ND	ng/ml
Endosulfan II	339,0	13,774	0	ND	ng/ml
4,4-DDE	318,0	16,211	7	1,6910	ng/ml
4,4-DDD	237,0	18,248	13	3,7659	ng/ml
4,4-DDT	237,0			ND	ng/ml

Lindane

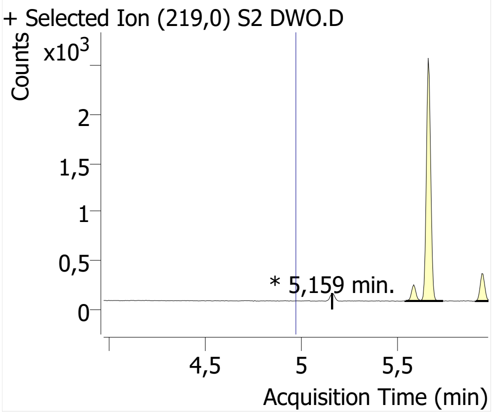

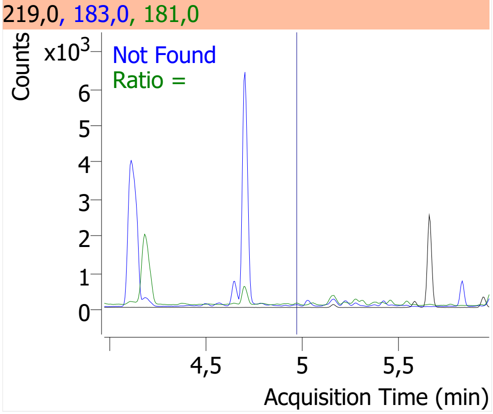

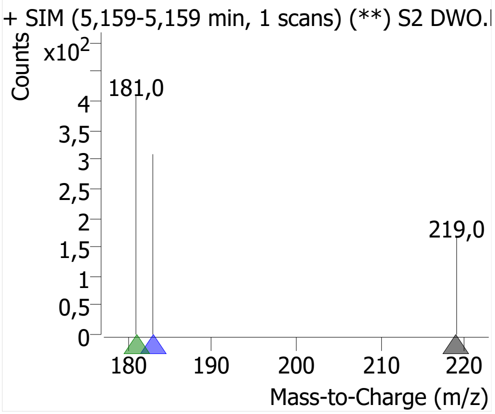

Hexachlorobenzene

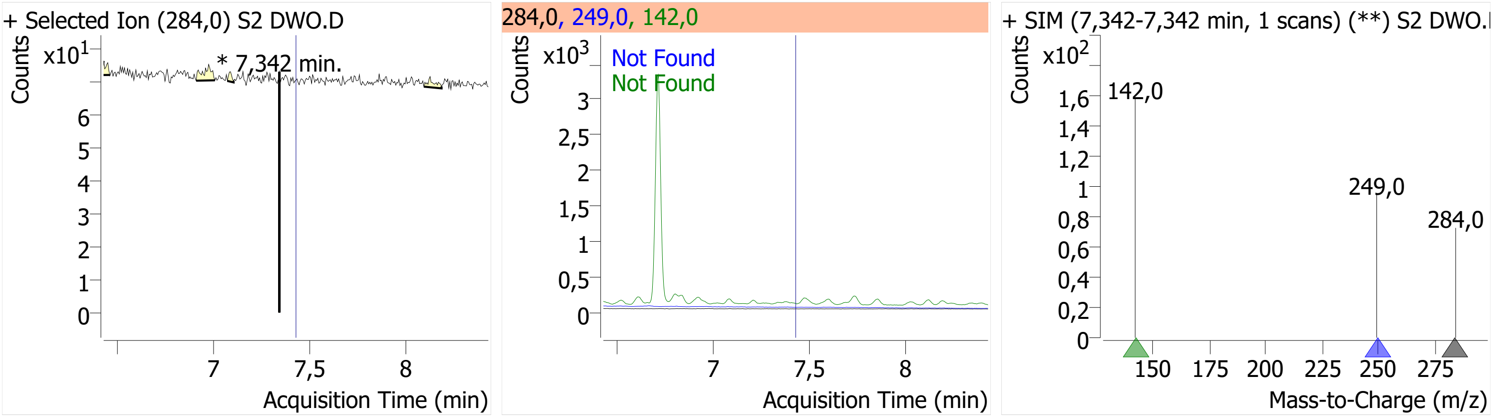

Aldrine

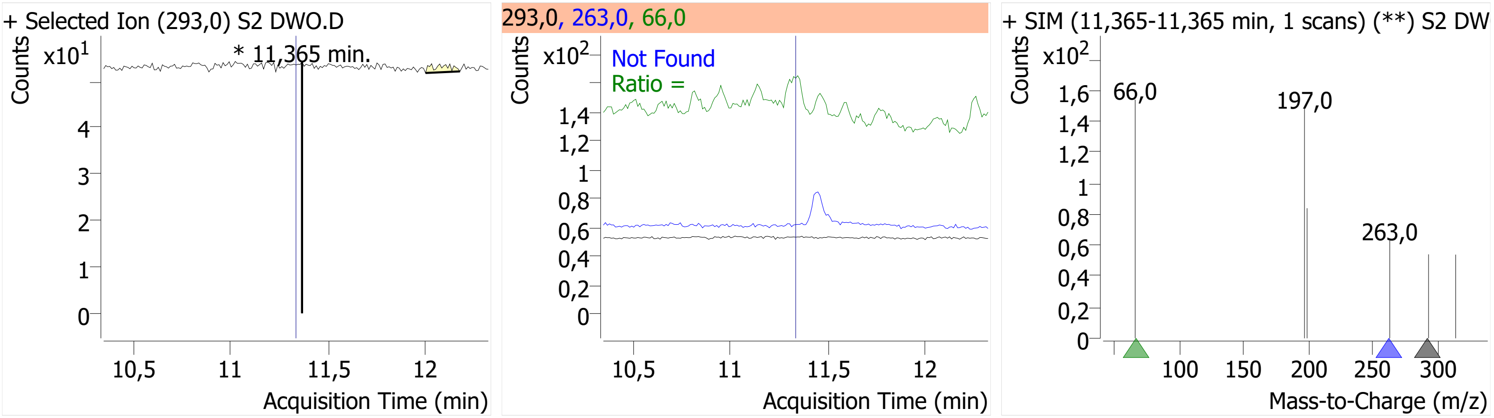

Chlorpyrifos

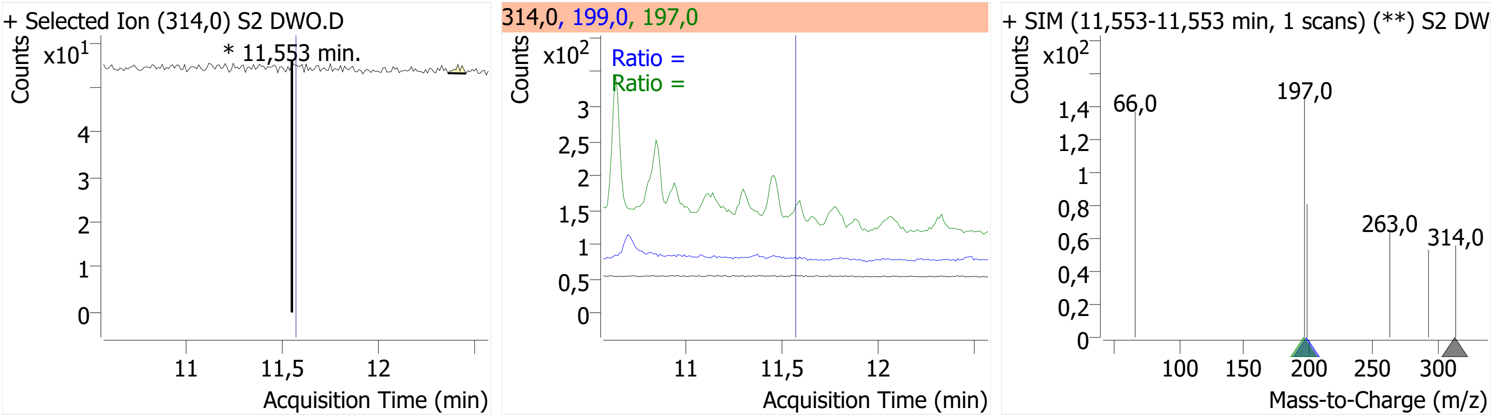

Endosulfan II

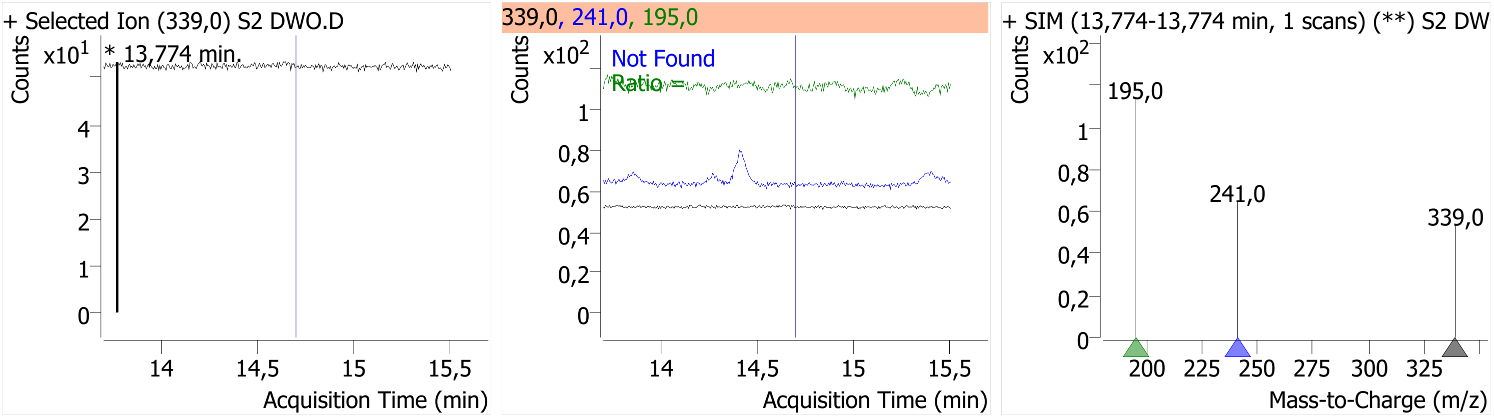

4,4-DDE

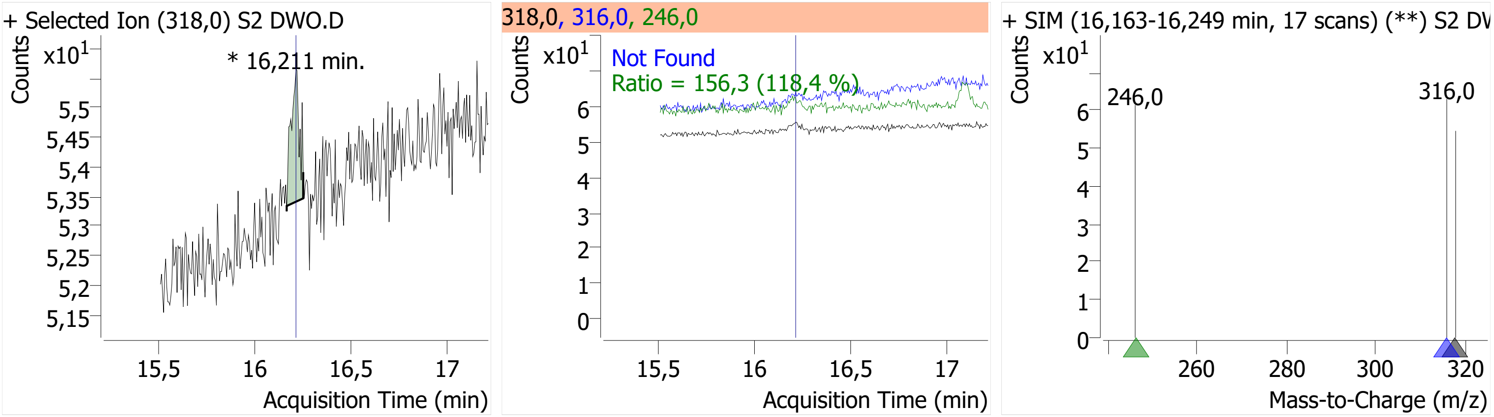

4,4-DDD

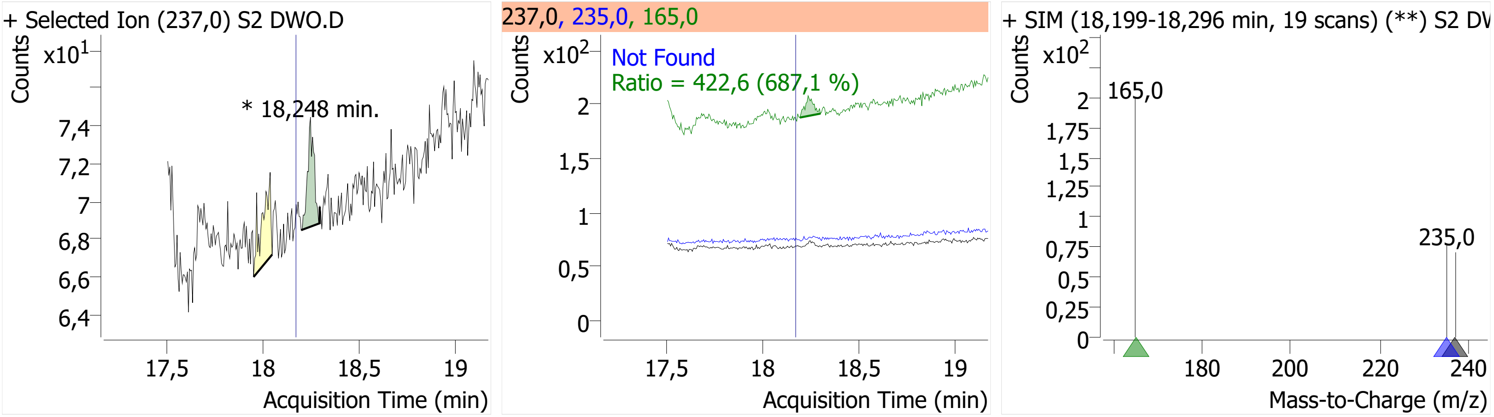

4,4-DDT

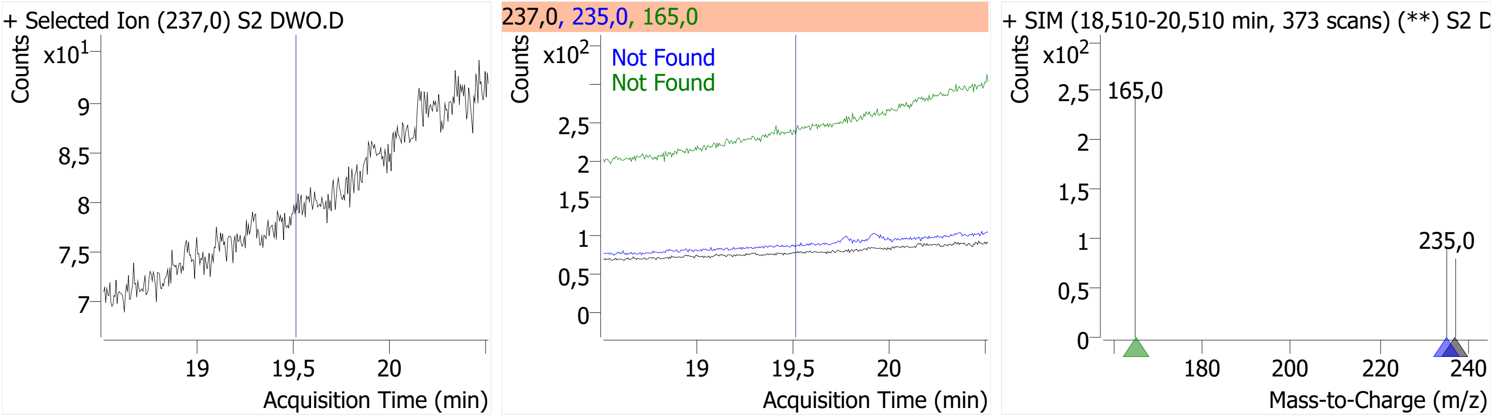

**Batch Path**	C:\Users\USER\OneDrive\Desktop\JU_Pesticide\MA\MA\QuantResults\Mohammed_Pest.batch.bin		
**Analysis Time**	13 Dec 2024 11:34	**Analyst Name**	DESKTOP-MRRPPC4\USER
**Report Time**	13 Dec 2024 11:37:29	**Reporter Name**	DESKTOP-MRRPPC4\USER
**Last Calib Update**	13 Dec 2024 11:34	**Batch State**	Processed
**Quant Batch Version**	10.0	**Quant Report Version**	10.0

**Acq. Time**	12 Nov 2024 22:43	**Data File**	S1 GGDO.D
**Sample Type**	Sample	**Sample Name**	Pest
**Dilution**	1	**Acq. Method**	pesticide std 12.11.2024

Sample Chromatogram

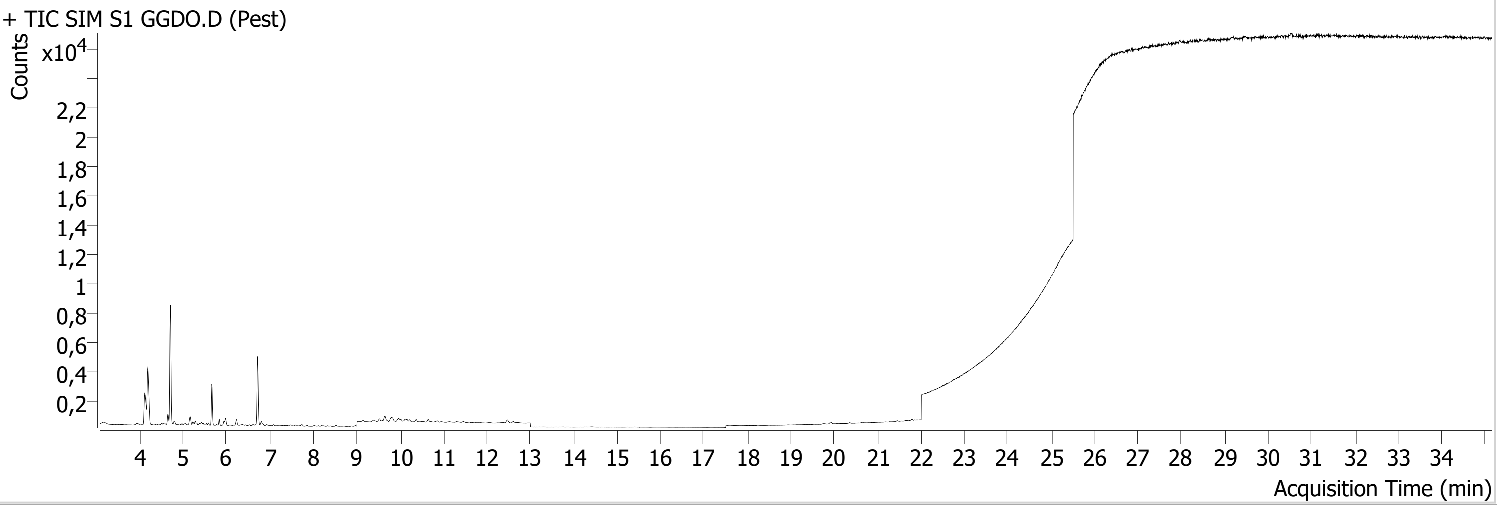

Compound	Transition	RT	Resp.	Final Conc	Units
Lindane	219,0	5,164	0	ND	ng/ml
Hexachlorobenzene	284,0	7,003	0	ND	ng/ml
Aldrine	293,0	11,490	0	ND	ng/ml
Chlorpyrifos	314,0	11,334	0	ND	ng/ml
Endosulfan II	339,0	14,689	0	ND	ng/ml
4,4-DDE	318,0	16,222	5	1,5690	ng/ml
4,4-DDD	237,0	18,253	16	4,2064	ng/ml
4,4-DDT	237,0	19,763	0	ND	ng/ml

Lindane

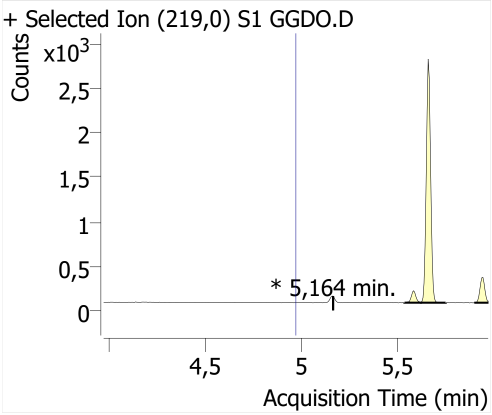

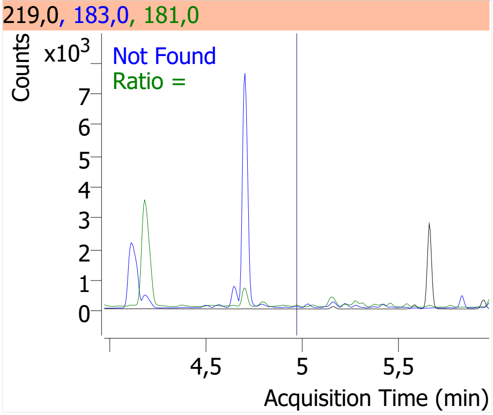

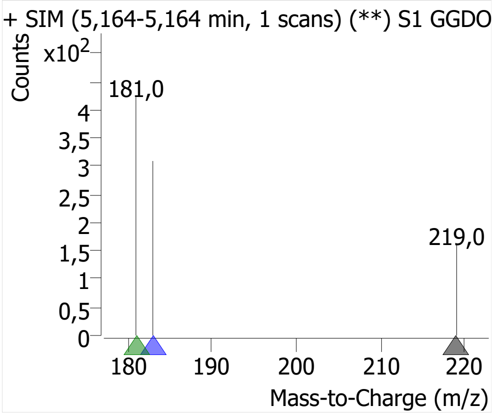

Agilent Trusted Answers

Hexachlorobenzene

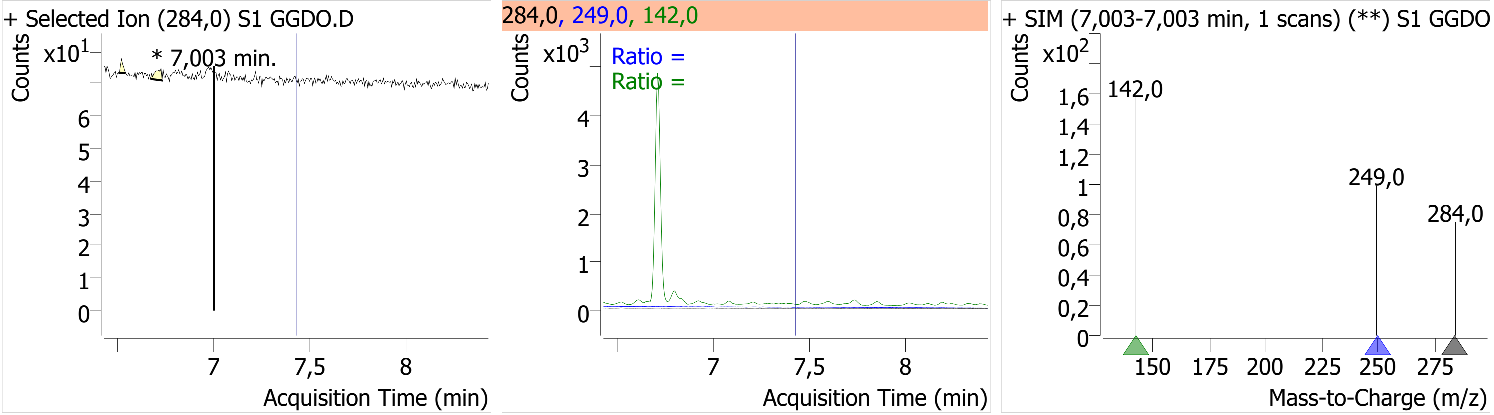

Aldrine

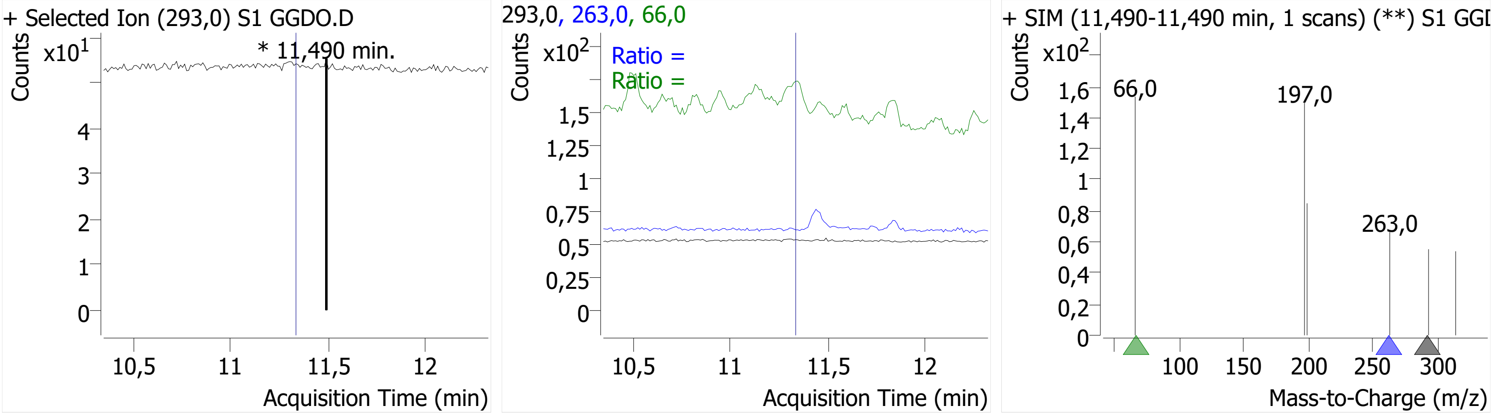

Chlorpyrifos

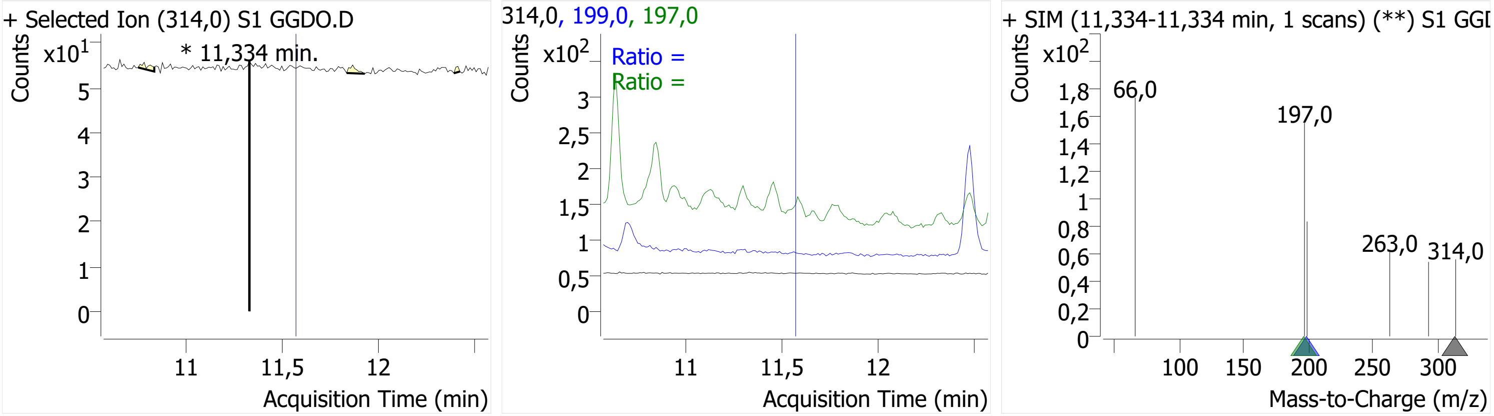

Endosulfan II

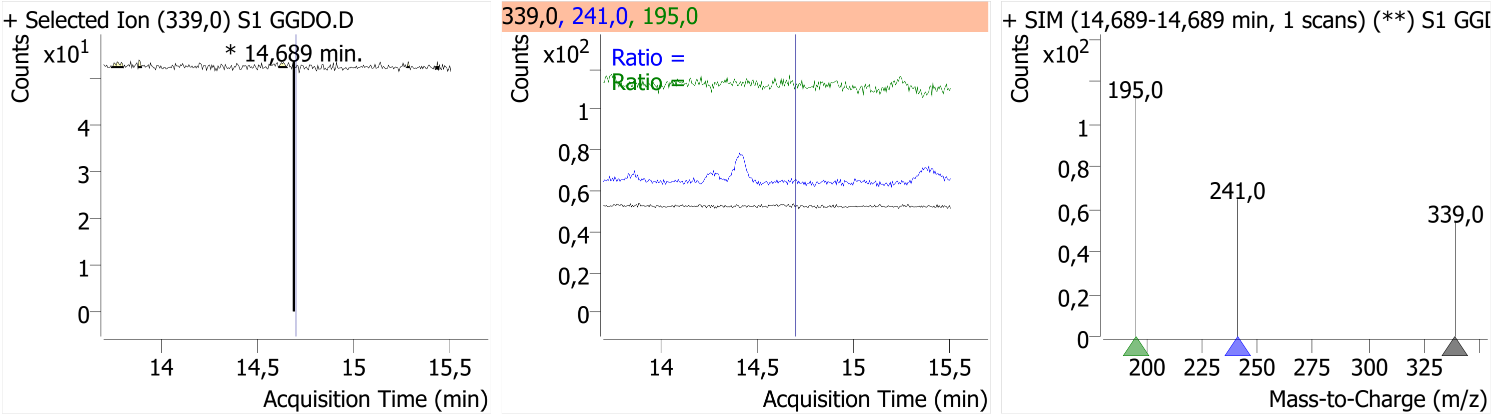

4,4-DDE

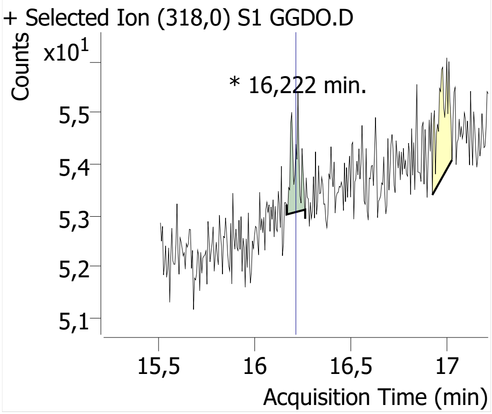

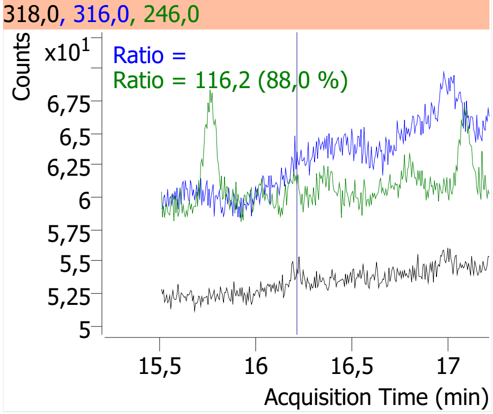

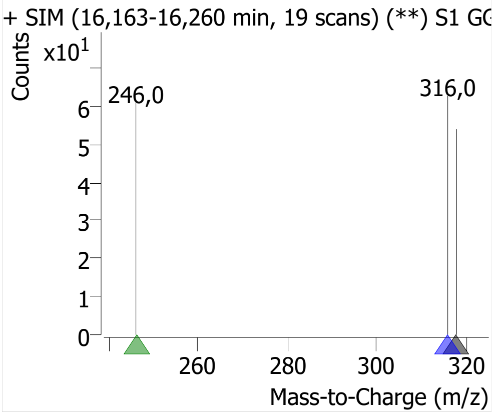

4,4-DDD

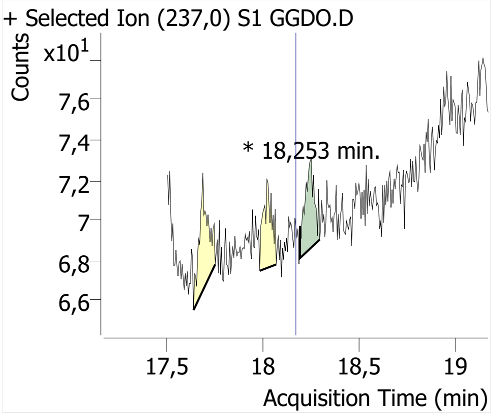

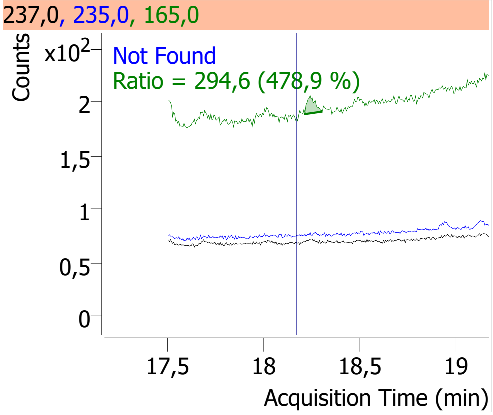

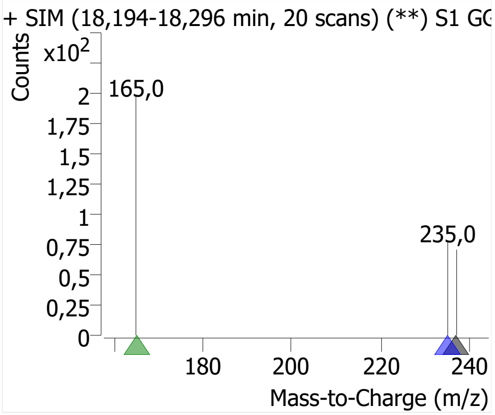

4,4-DDT

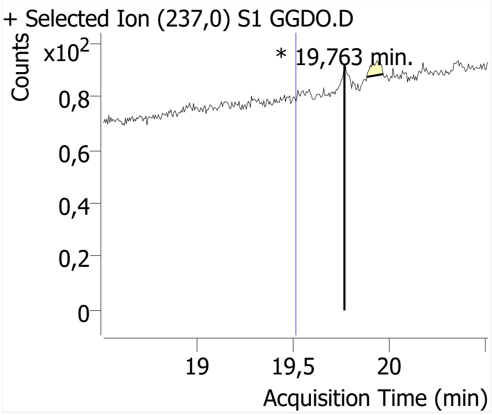

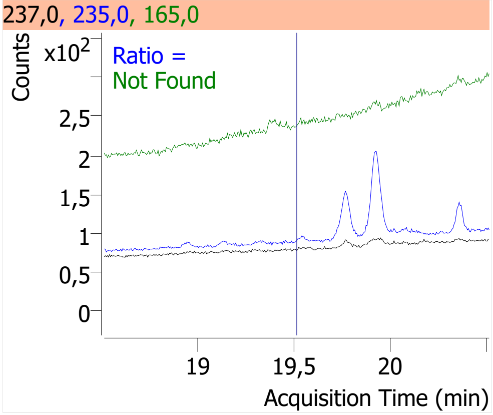

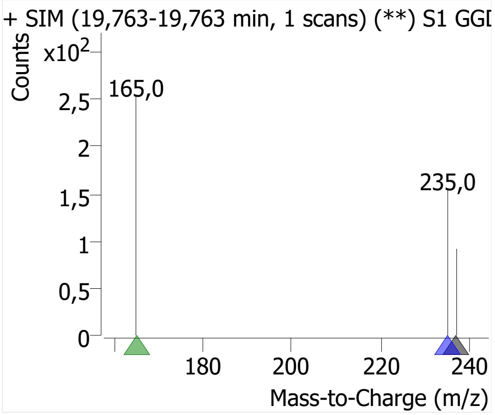

**Batch Path**	C:\Users\USER\OneDrive\Desktop\JU_Pesticide\MA\MA\QuantResults\Mohammed_Pest.batch.bin		
**Analysis Time**	13 Dec 2024 11:34	**Analyst Name**	DESKTOP-MRRPPC4\USER
**Report Time**	13 Dec 2024 11:37:30	**Reporter Name**	DESKTOP-MRRPPC4\USER
**Last Calib Update**	13 Dec 2024 11:34	**Batch State**	Processed
**Quant Batch Version**	10.0	**Quant Report Version**	10.0

**Acq. Time**	12 Nov 2024 23:23	**Data File**	S2 SUO.D
**Sample Type**	Sample	**Sample Name**	Pest
**Dilution**	1	**Acq. Method**	pesticide std 12.11.2024

Sample Chromatogram

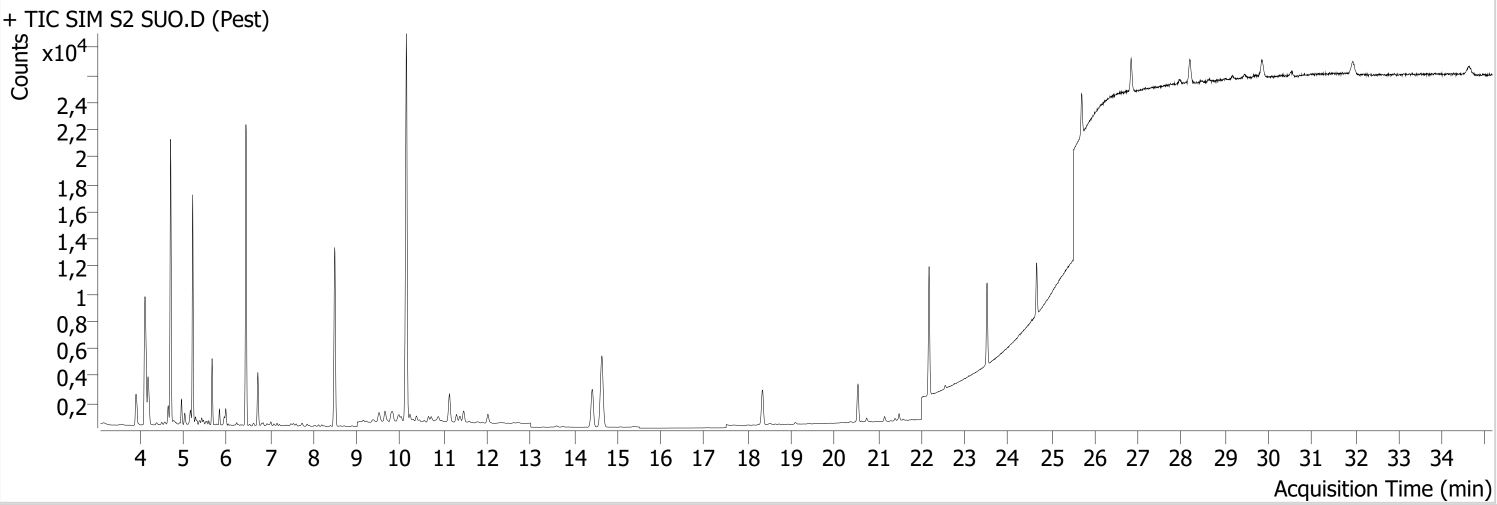

Compound	Transition	RT	Resp.	Final Conc	Units
Lindane	219,0	4,955	1241	1831,9407	ng/ml
Hexachlorobenzene	284,0	6,443	0	ND	ng/ml
Aldrine	293,0	11,125	0	ND	ng/ml
Chlorpyrifos	314,0	11,574	0	ND	ng/ml
Endosulfan II	339,0	14,641	0	ND	ng/ml
4,4-DDE	318,0	16,206	7	1,6783	ng/ml
4,4-DDD	237,0	18,344	1727	317,9851	ng/ml
4,4-DDT	237,0	19,639	53	63,5603	ng/ml

Lindane

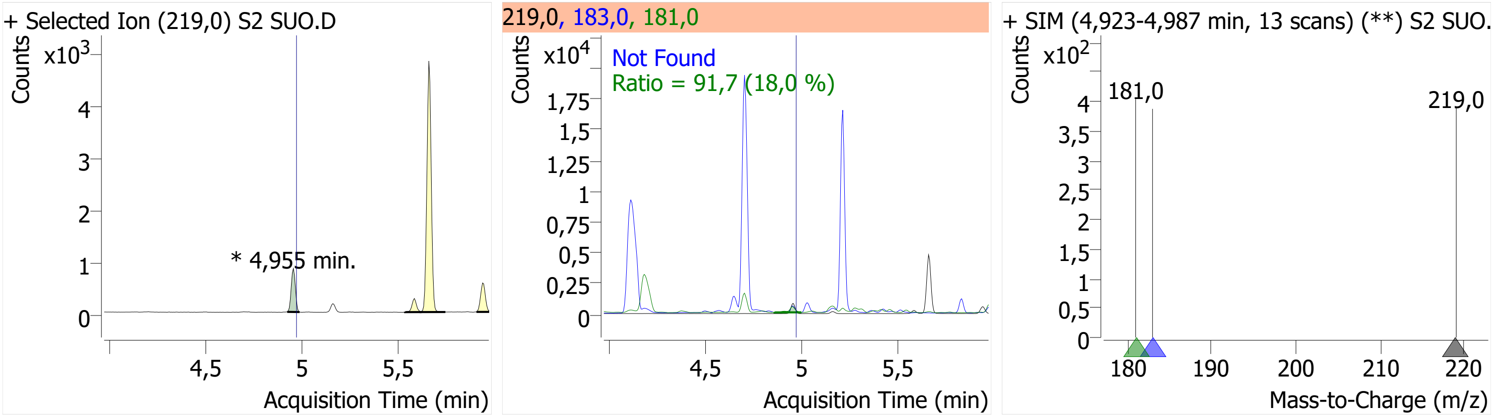

Hexachlorobenzene

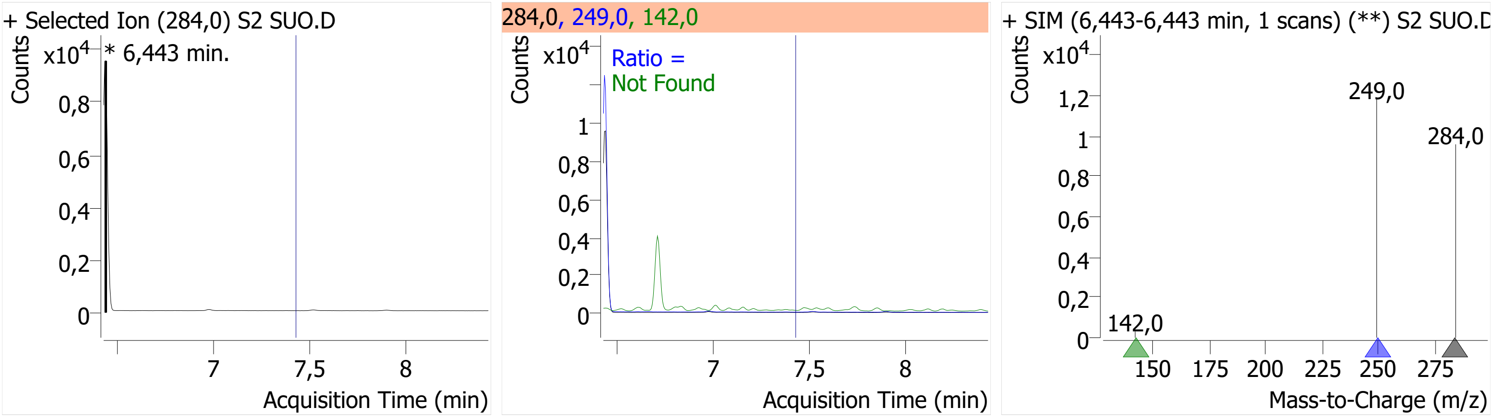

Aldrine

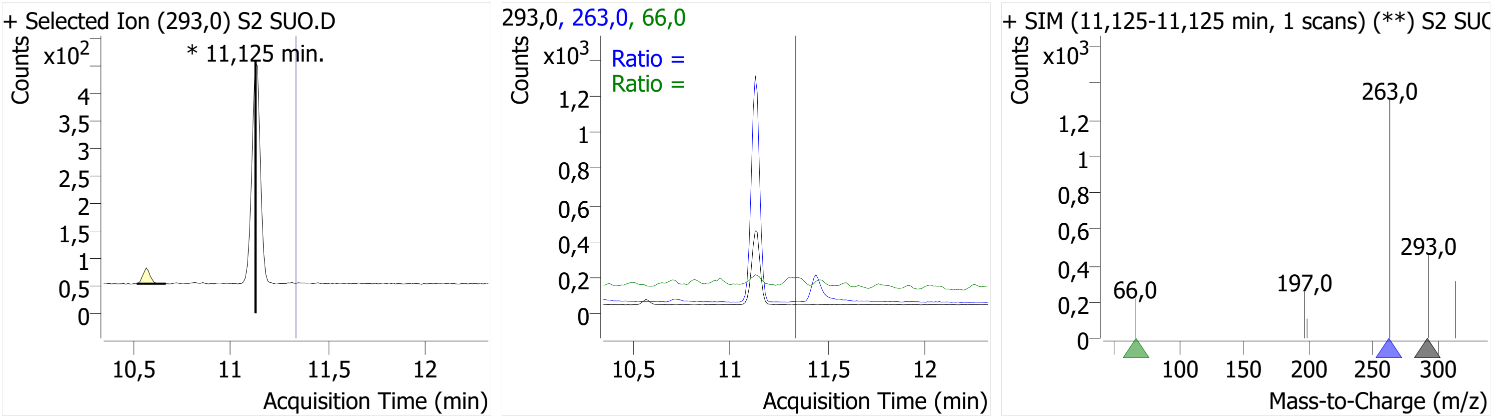

Chlorpyrifos

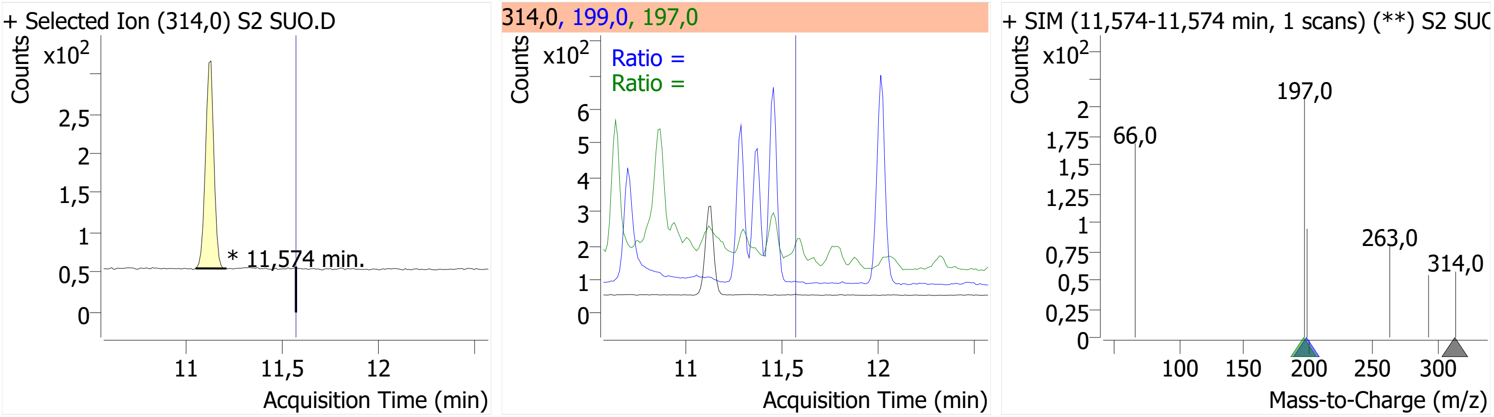

Endosulfan II

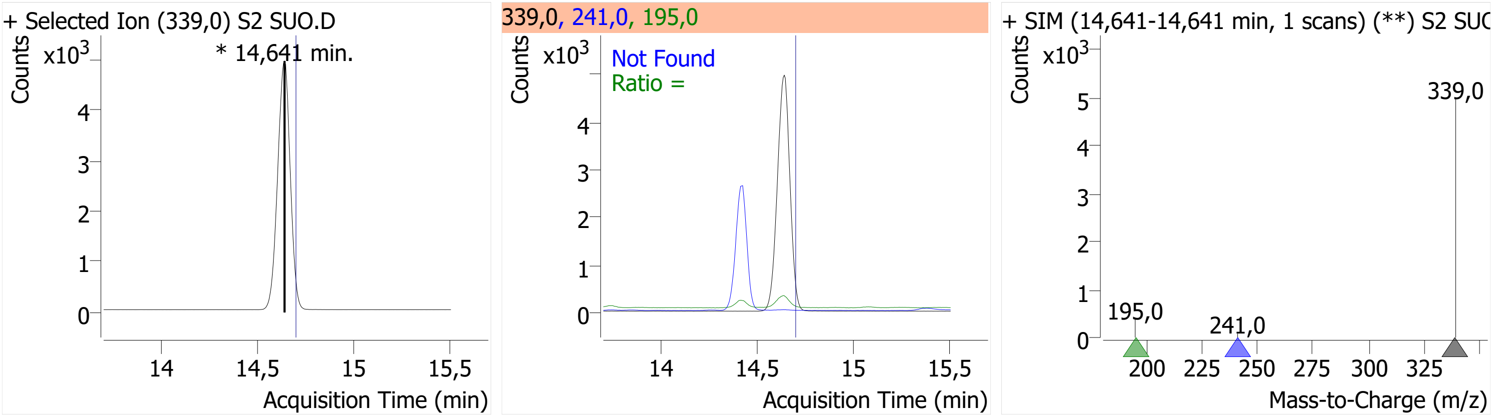

4,4-DDE

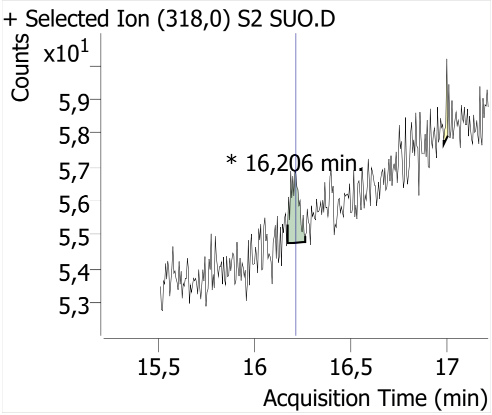

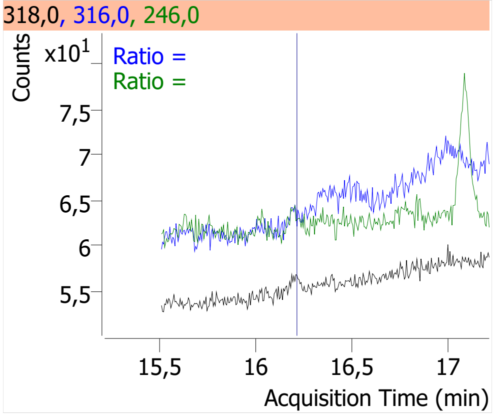

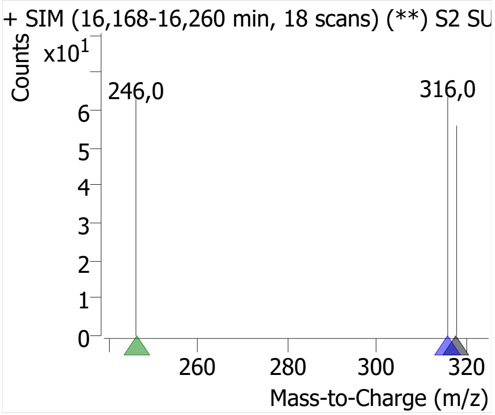

4,4-DDD

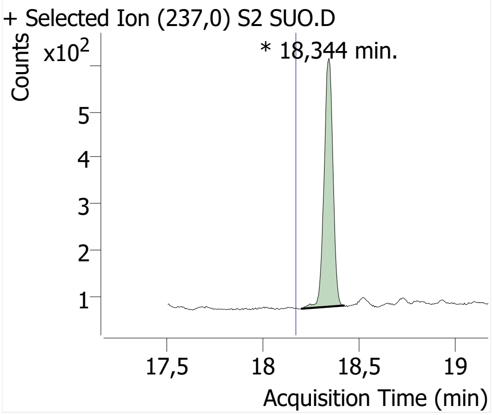

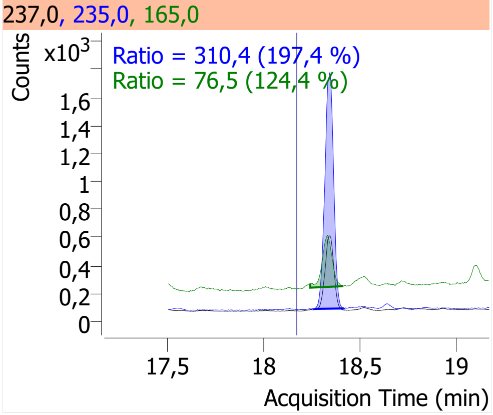

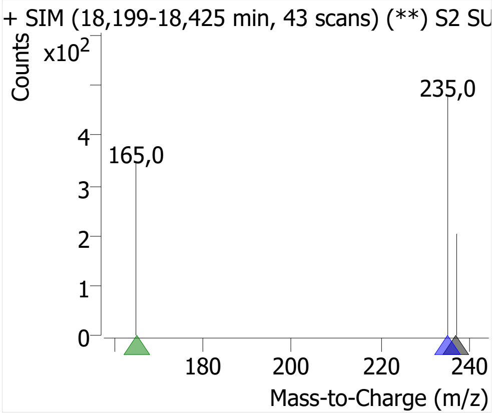

4,4-DDT

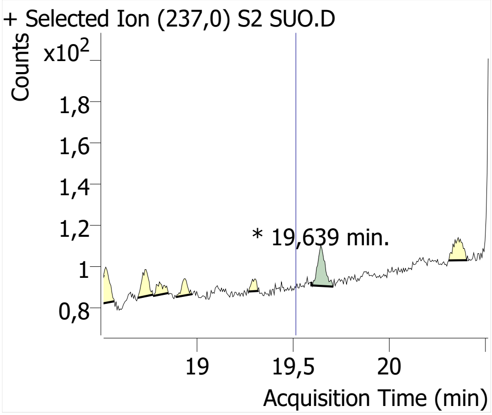

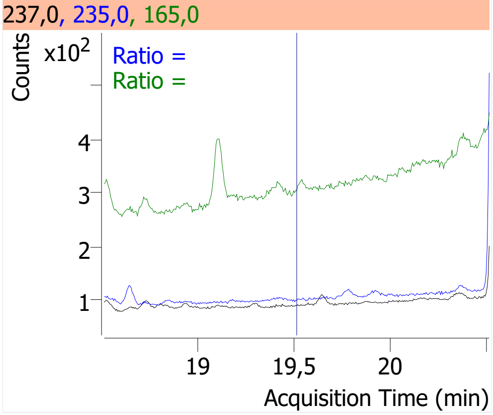

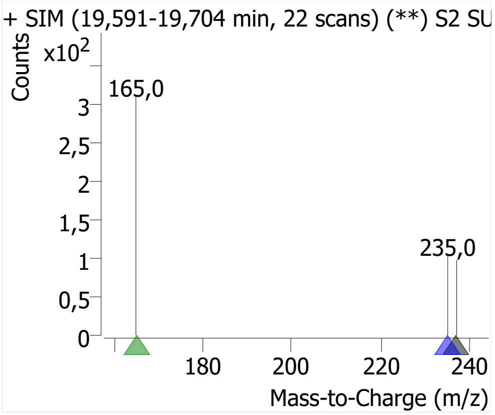

Quantitative Analysis Complete Report

**Batch Path**	C:\Users\USER\OneDrive\Desktop\JU_Pesticide\MA\MA\QuantResults\Mohammed_Pest.batch.bin		
**Analysis Time**	13 Dec 2024 11:34	**Analyst Name**	DESKTOP-MRRPPC4\USER
**Report Time**	13 Dec 2024 11:37:30	**Reporter Name**	DESKTOP-MRRPPC4\USER
**Last Calib Update**	13 Dec 2024 11:34	**Batch State**	Processed
**Quant Batch Version**	10.0	**Quant Report Version**	10.0

**Acq. Time**	13 Nov 2024 00:04	**Data File**	S1 DOO.D
**Sample Type**	Sample	**Sample Name**	Pest
**Dilution**	1	**Acq. Method**	pesticide std 12.11.2024

Sample Chromatogram

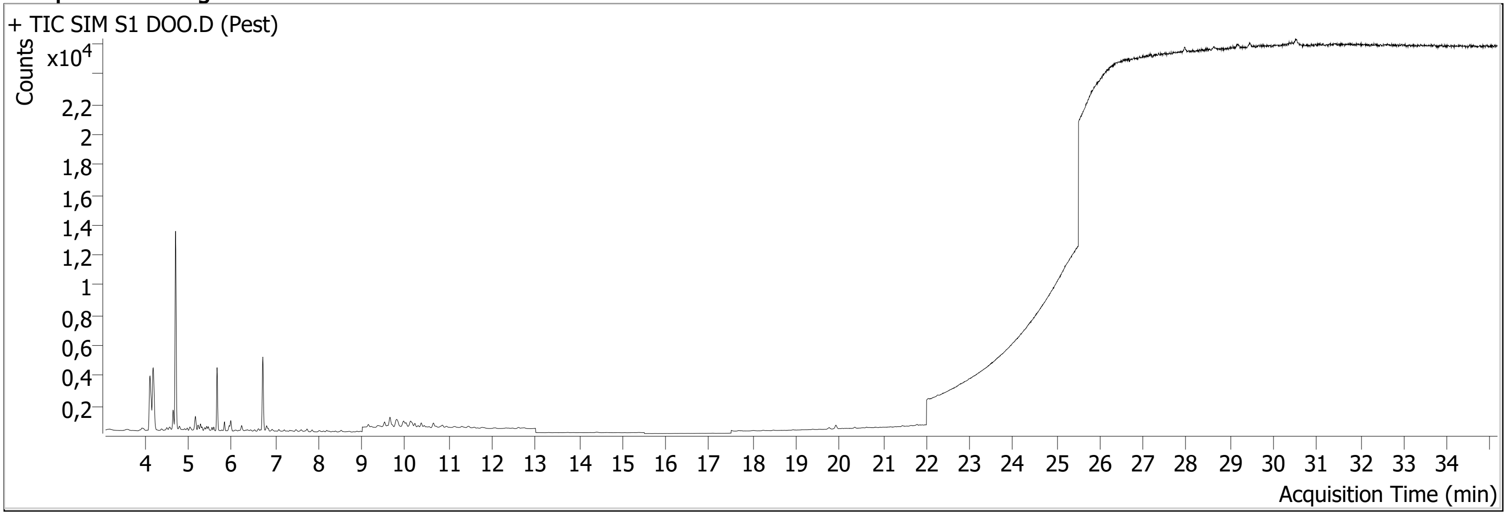

Compound	Transition	RT	Resp.	Final Conc	Units
Lindane	219,0	5,159	0	ND	ng/ml
Hexachlorobenzene	284,0	6,976	0	ND	ng/ml
Aldrine	293,0	11,303	0	ND	ng/ml
Chlorpyrifos	314,0			ND	ng/ml
Endosulfan II	339,0	14,625	35	29,2100	ng/ml
4,4-DDE	318,0			ND	ng/ml
4,4-DDD	237,0	18,237	17	4,4821	ng/ml
4,4-DDT	237,0	19,763	0	ND	ng/ml

Lindane

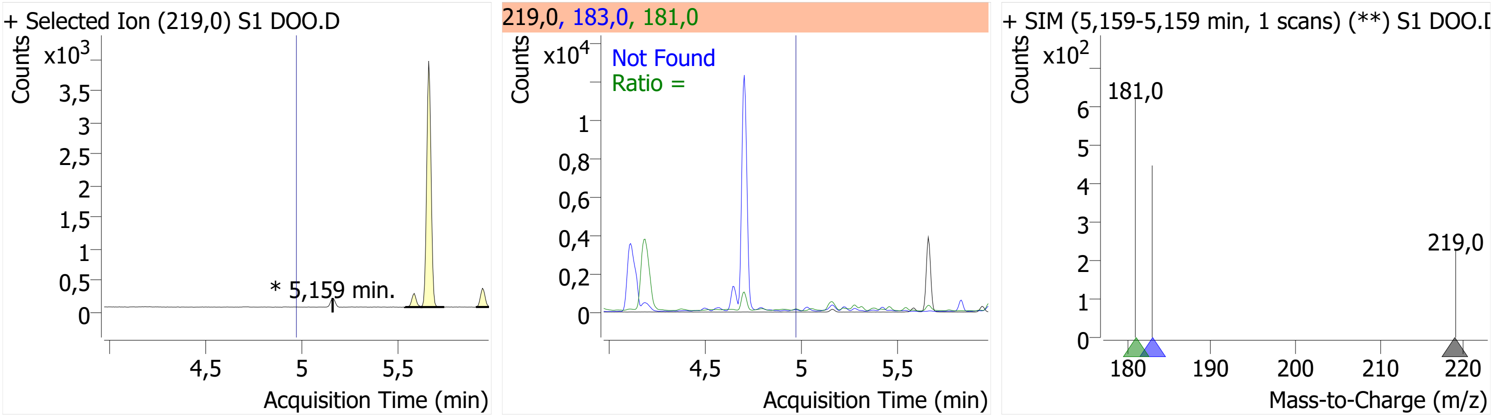

Hexachlorobenzene

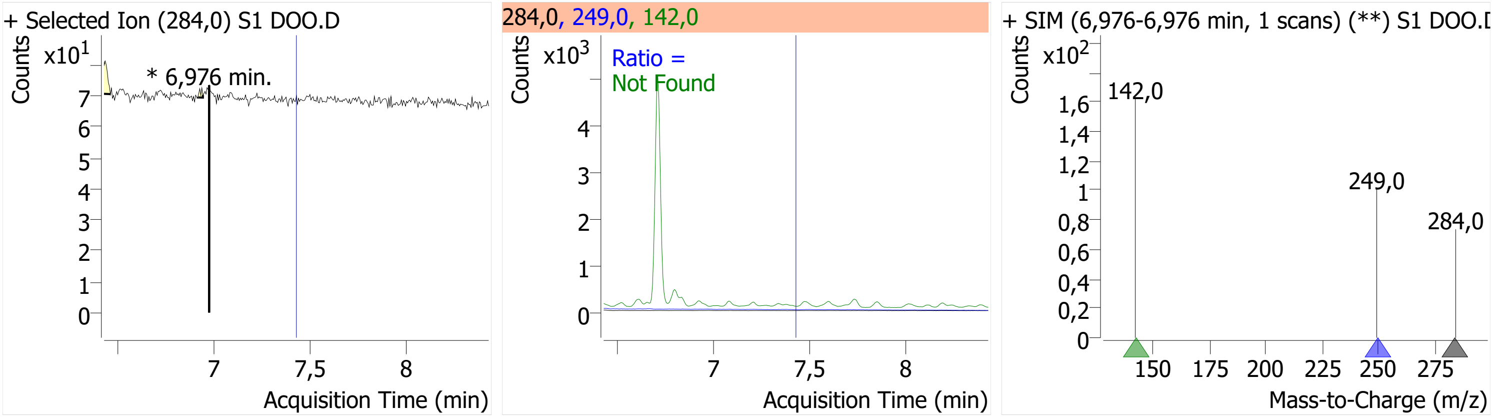

Aldrine

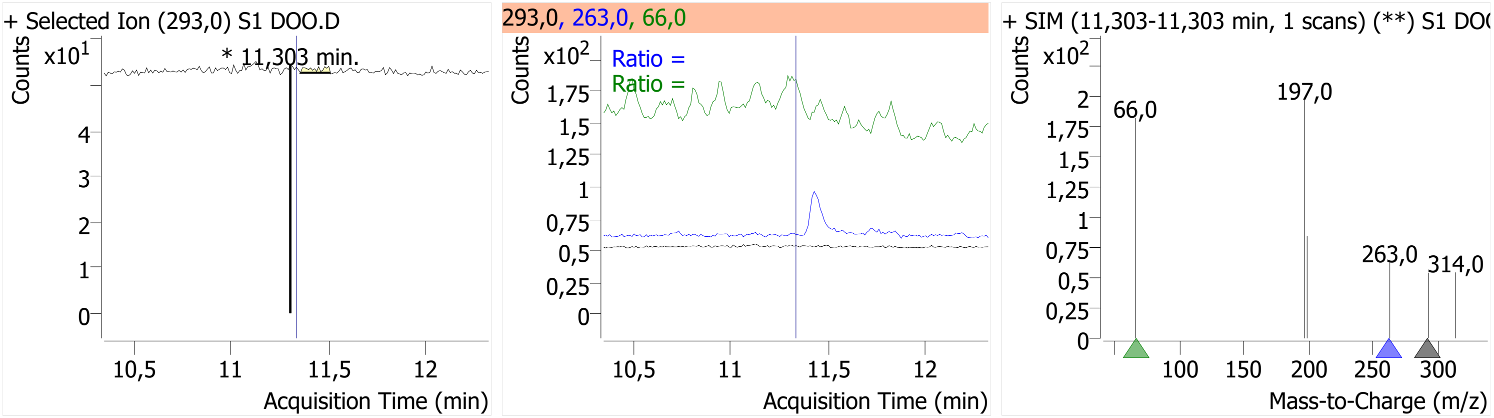

Chlorpyrifos

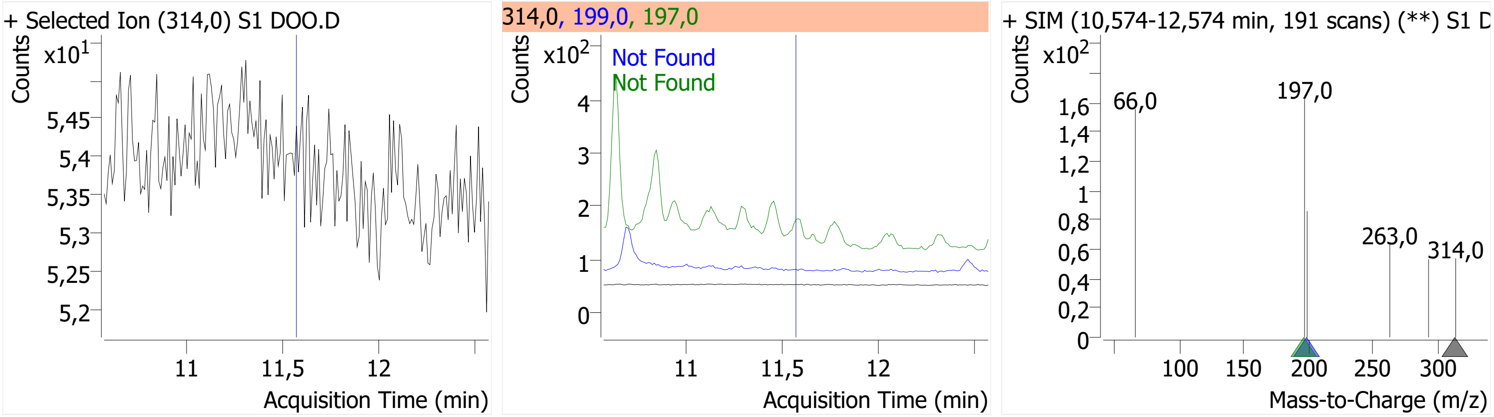

Endosulfan II

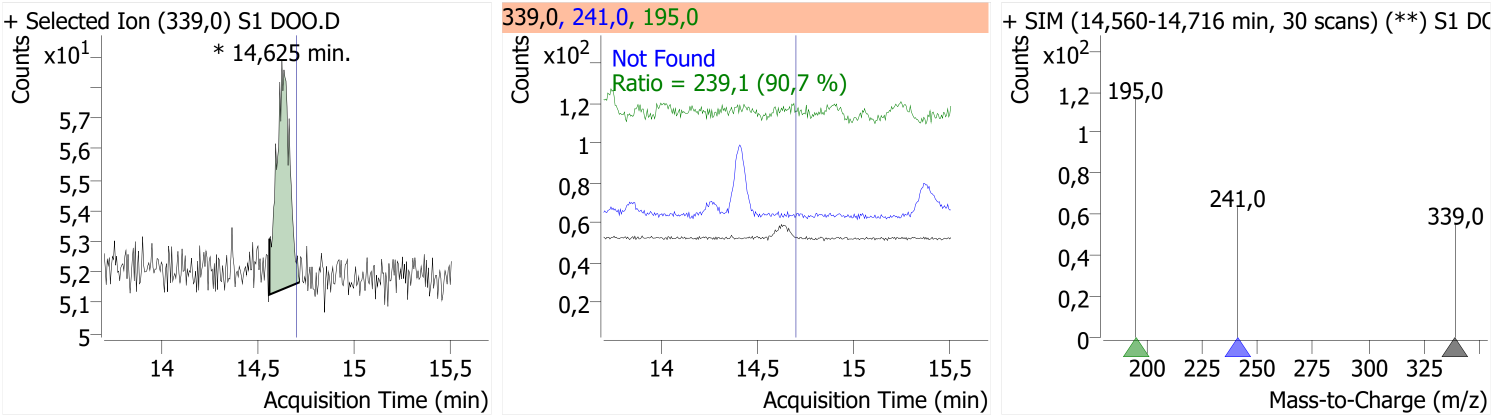

4,4-DDE

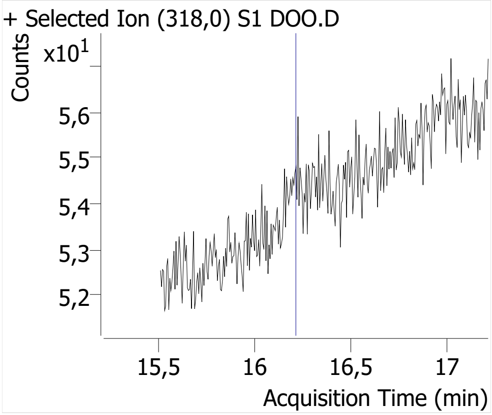

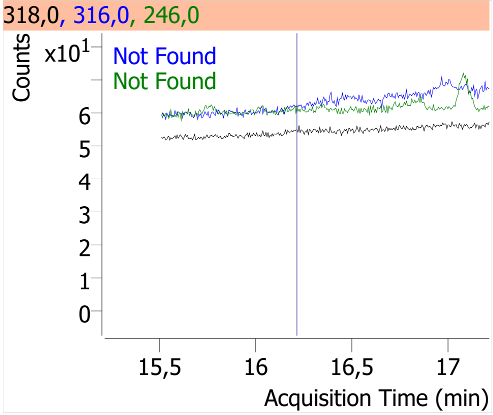

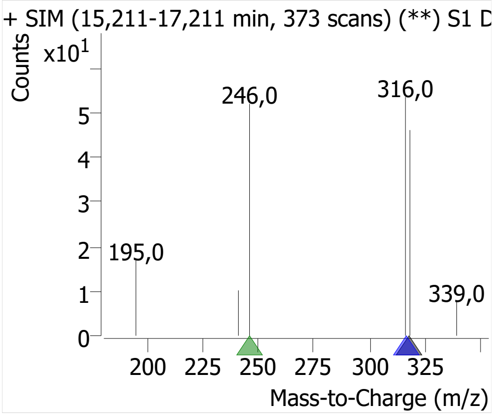

4,4-DDD

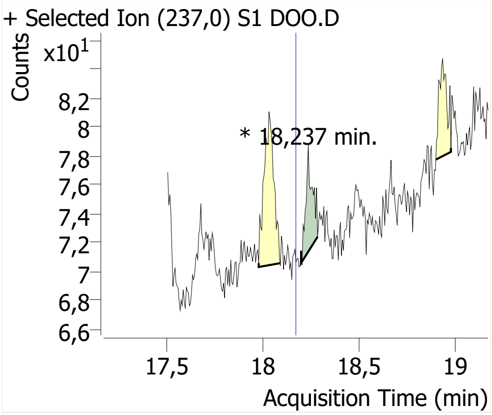

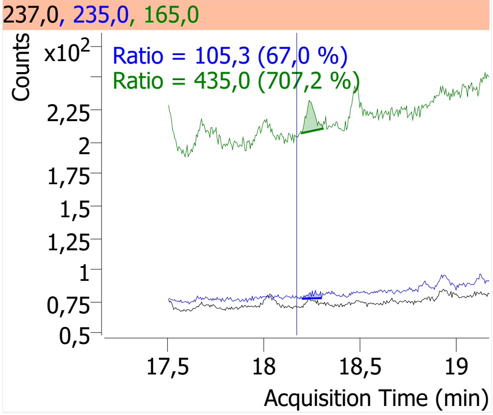

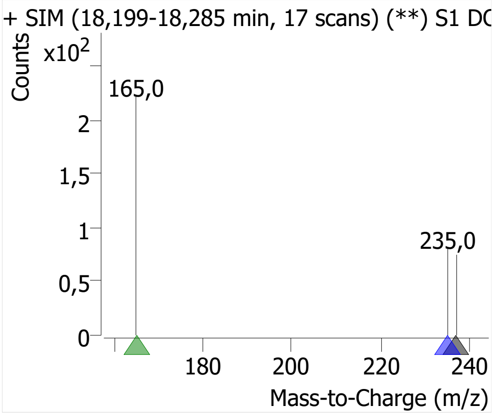

4,4-DDT

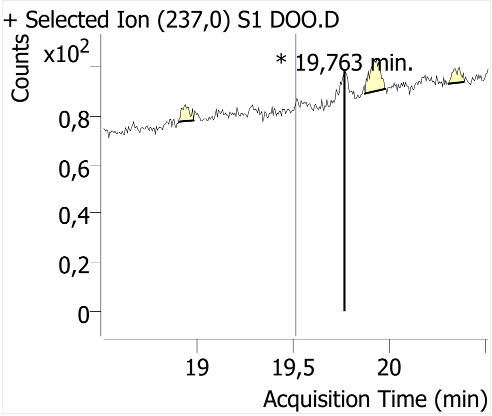

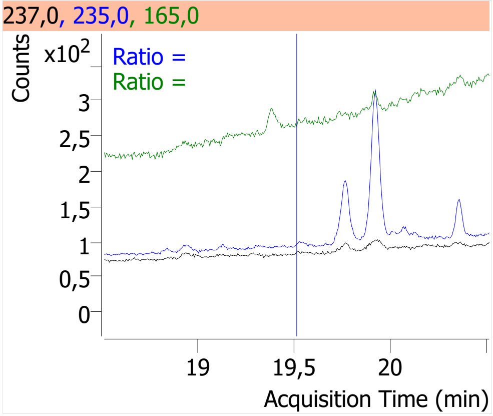

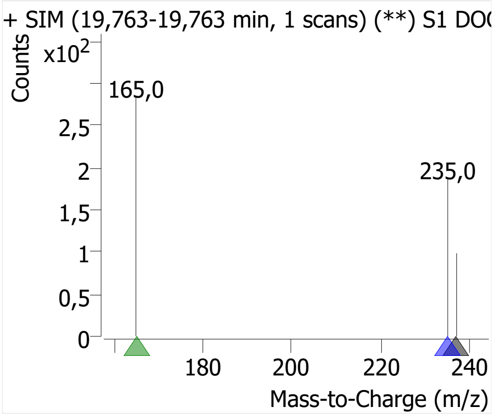

Quantitative Analysis Complete Report

**Batch Path**	C:\Users\USER\OneDrive\Desktop\JU_Pesticide\MA\MA\QuantResults\Mohammed_Pest.batch.bin		
**Analysis Time**	13 Dec 2024 11:34	**Analyst Name**	DESKTOP-MRRPPC4\USER
**Report Time**	13 Dec 2024 11:37:31	**Reporter Name**	DESKTOP-MRRPPC4\USER
**Last Calib Update**	13 Dec 2024 11:34	**Batch State**	Processed
**Quant Batch Version**	10.0	**Quant Report Version**	10.0

**Acq. Time**	13 Nov 2024 00:45	**Data File**	S2 GJJ.D
**Sample Type**	Sample	**Sample Name**	Pest
**Dilution**	1	**Acq. Method**	pesticide std 12.11.2024

Sample Chromatogram

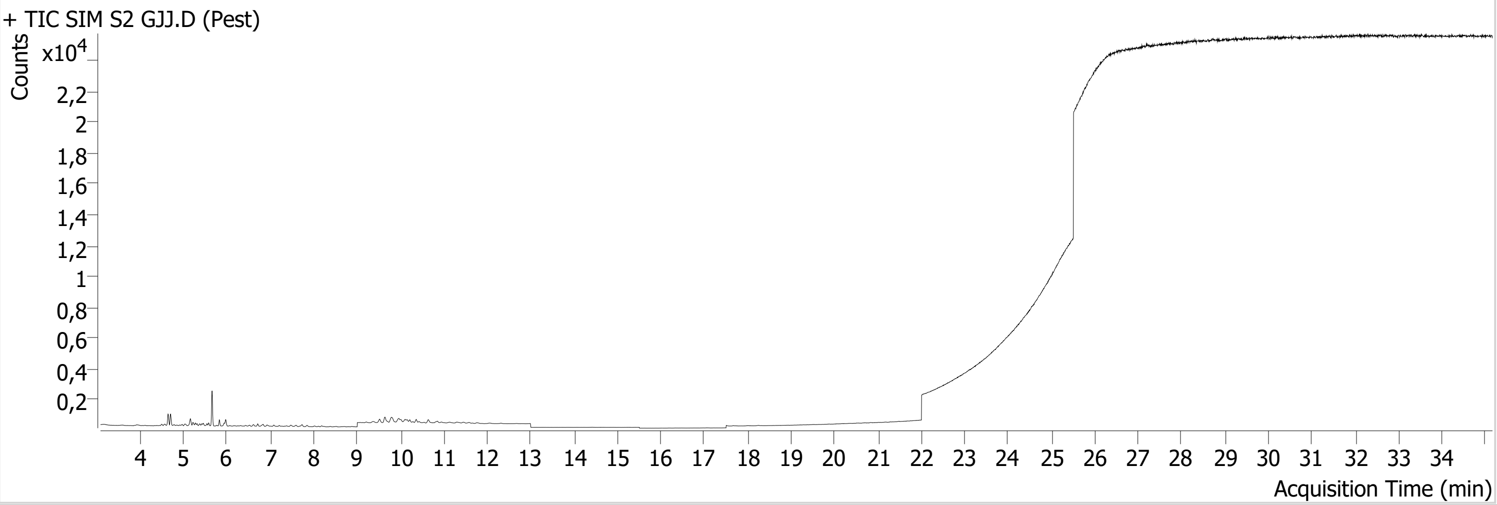

Compound	Transition	RT	Resp.	Final Conc	Units
Lindane	219,0	5,578	0	ND	ng/ml
Hexachlorobenzene	284,0	7,740	0	ND	ng/ml
Aldrine	293,0	11,281	0	ND	ng/ml
Chlorpyrifos	314,0	11,563	15	8,9950	ng/ml
Endosulfan II	339,0	14,608	12	6,4961	ng/ml
4,4-DDE	318,0	16,985	0	ND	ng/ml
4,4-DDD	237,0	18,237	15	4,0885	ng/ml
4,4-DDT	237,0			ND	ng/ml

Lindane

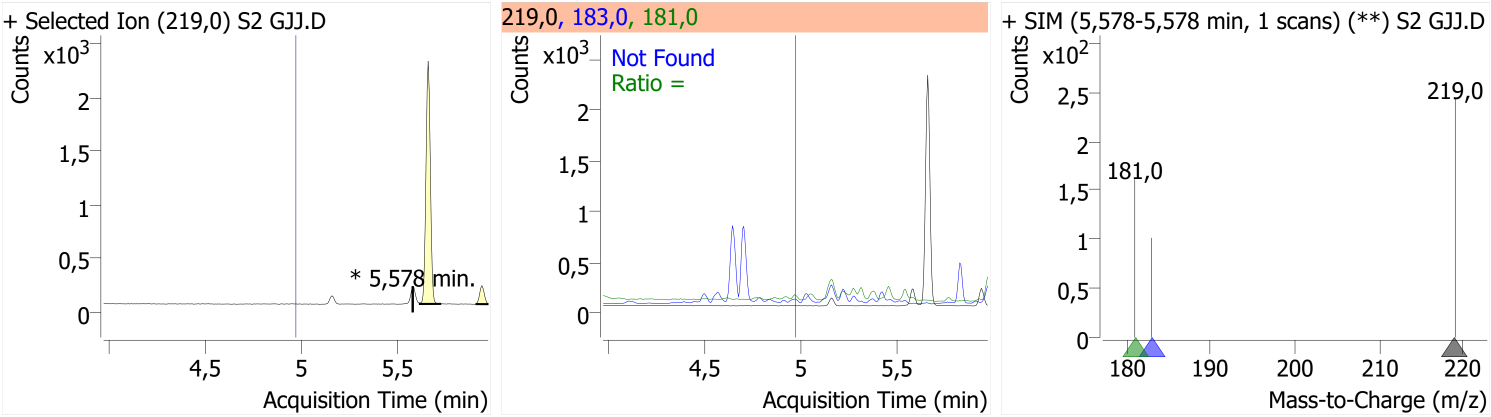

Agilent Trusted Answers

Hexachlorobenzene

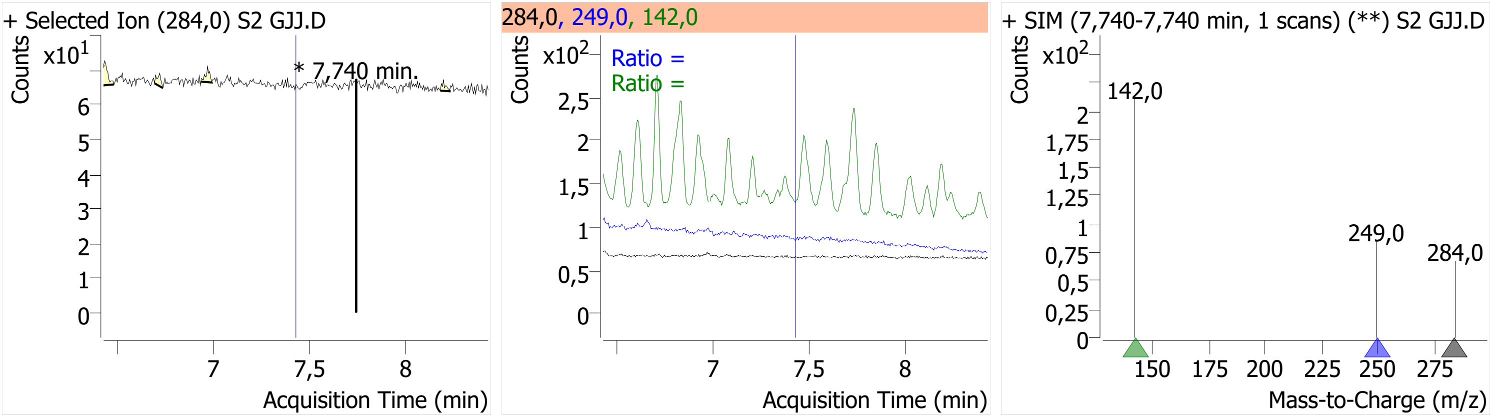

Aldrine

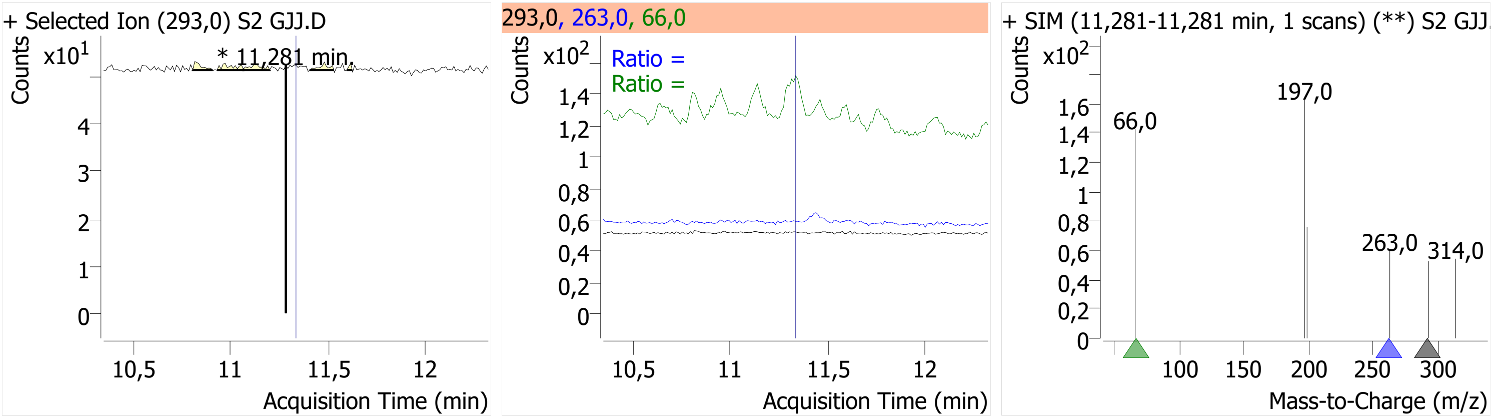

Chlorpyrifos

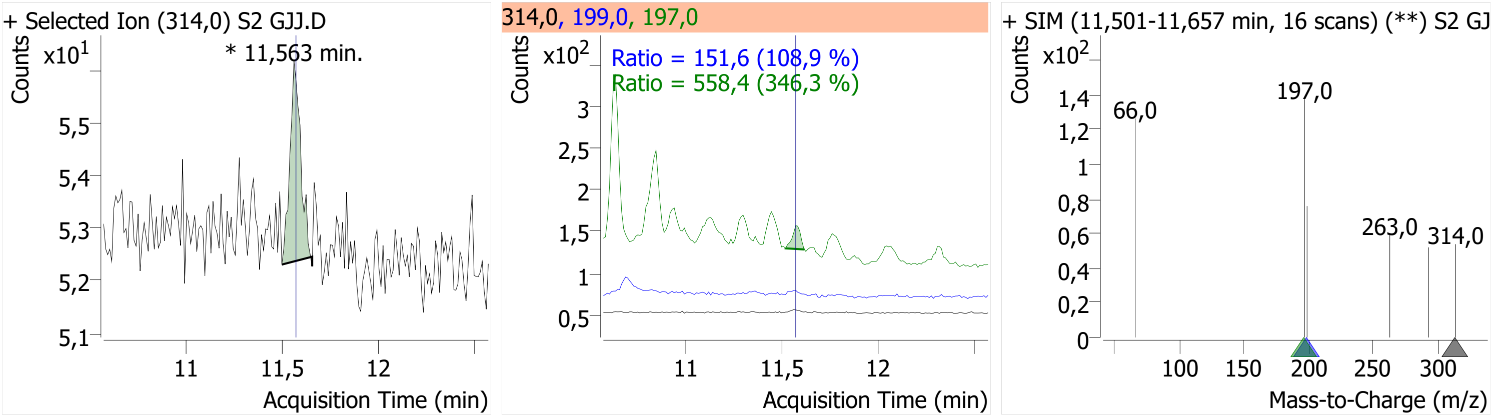

Endosulfan II

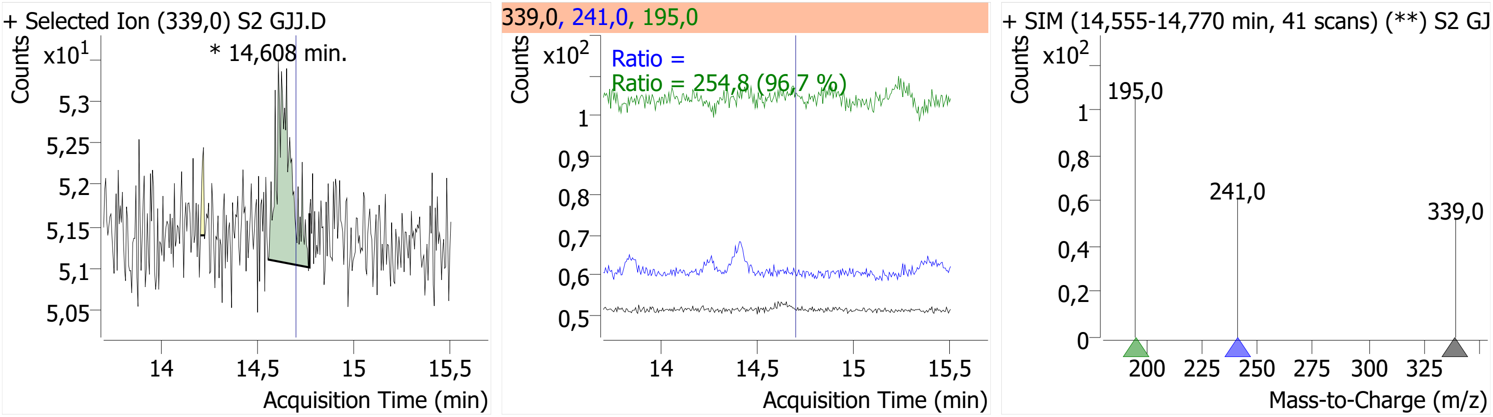

4,4-DDE

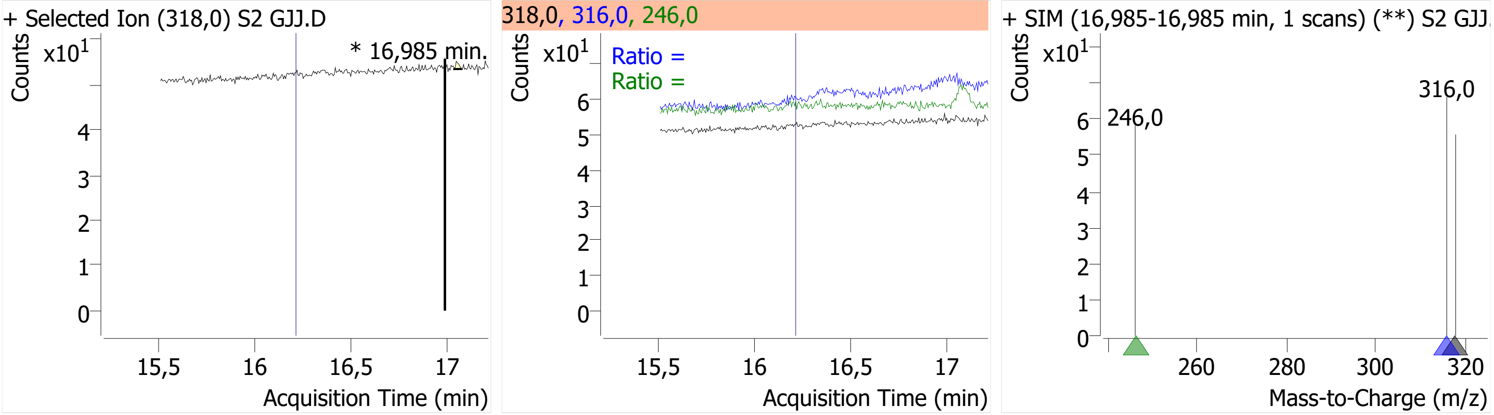

4,4-DDD

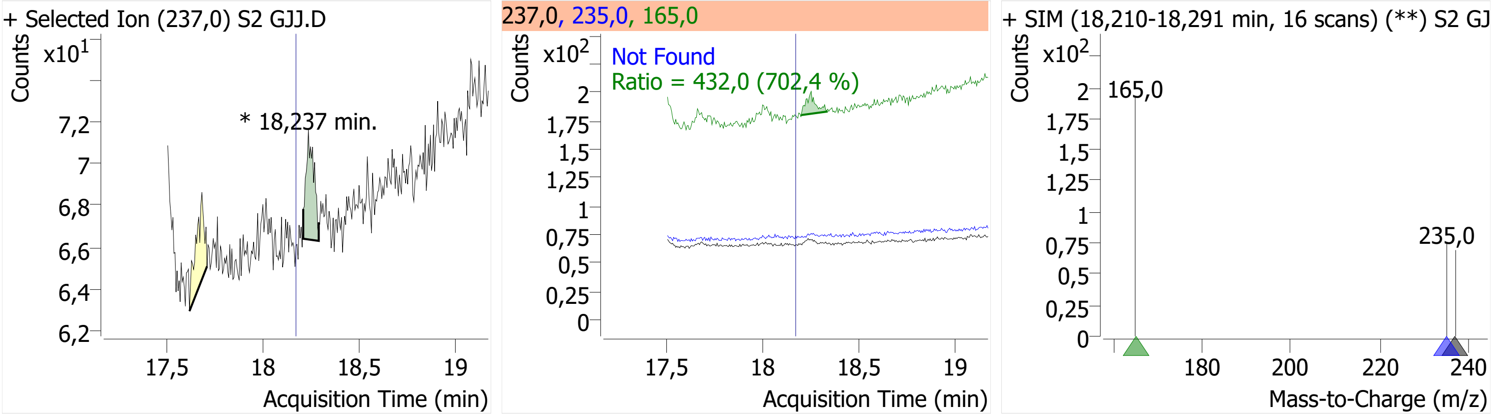

4,4-DDT

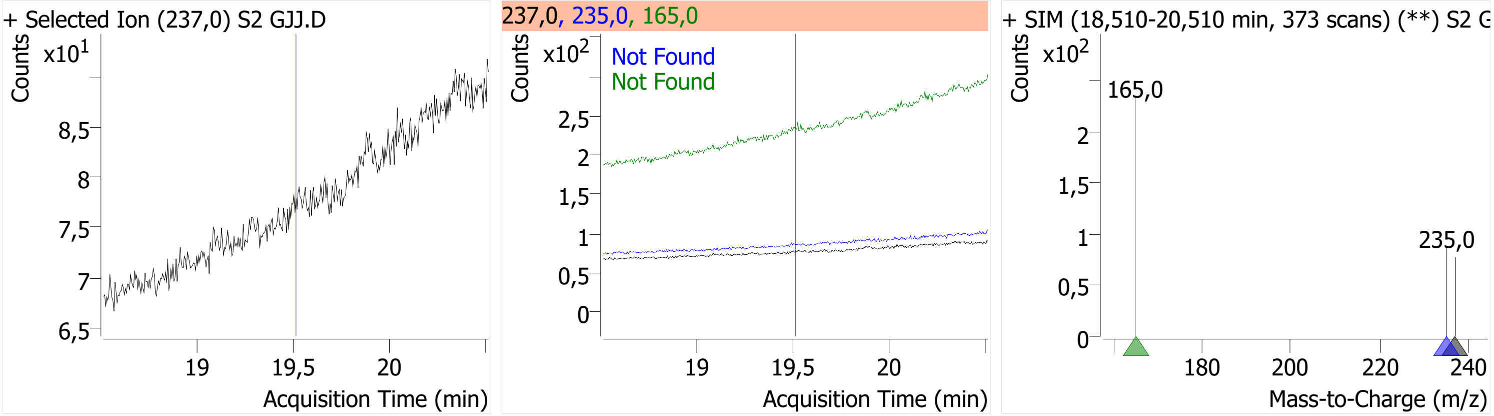

Quantitative Analysis Complete Report

**Batch Path**	C:\Users\USER\OneDrive\Desktop\JU_Pesticide\MA\MA\QuantResults\Mohammed_Pest.batch.bin		
**Analysis Time**	13 Dec 2024 11:34	**Analyst Name**	DESKTOP-MRRPPC4\USER
**Report Time**	13 Dec 2024 11:37:32	**Reporter Name**	DESKTOP-MRRPPC4\USER
**Last Calib Update**	13 Dec 2024 11:34	**Batch State**	Processed
**Quant Batch Version**	10.0	**Quant Report Version**	10.0

**Acq. Time**	13 Nov 2024 01:25	**Data File**	S1 SDO.D
**Sample Type**	Sample	**Sample Name**	Pest
**Dilution**	1	**Acq. Method**	pesticide std 12.11.2024

Sample Chromatogram

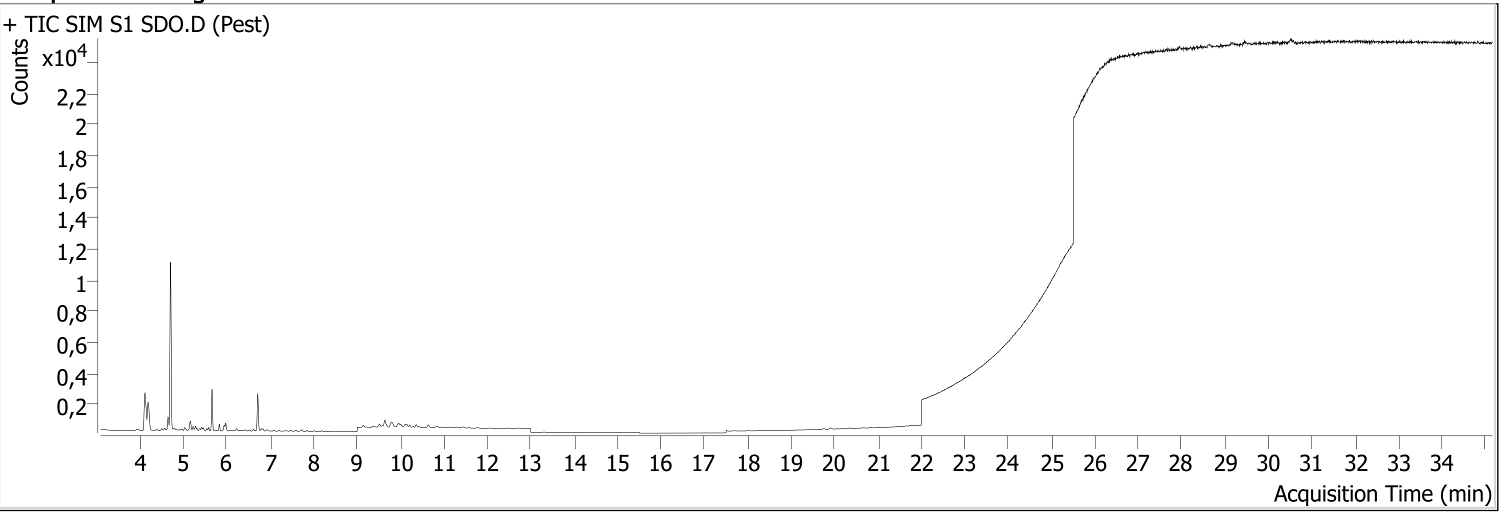

Compound	Transition	RT	Resp.	Final Conc	Units
Lindane	219,0	5,159	0	ND	ng/ml
Hexachlorobenzene	284,0	7,078	0	ND	ng/ml
Aldrine	293,0	11,114	0	ND	ng/ml
Chlorpyrifos	314,0	11,323	0	ND	ng/ml
Endosulfan II	339,0	14,619	46	40,8853	ng/ml
4,4-DDE	318,0	16,759	0	ND	ng/ml
4,4-DDD	237,0	18,242	15	4,1848	ng/ml
4,4-DDT	237,0	19,763	0	ND	ng/ml

Lindane

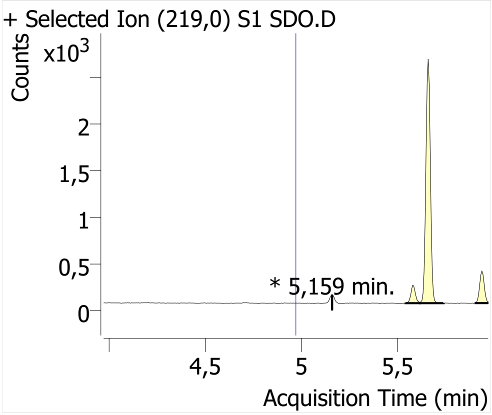

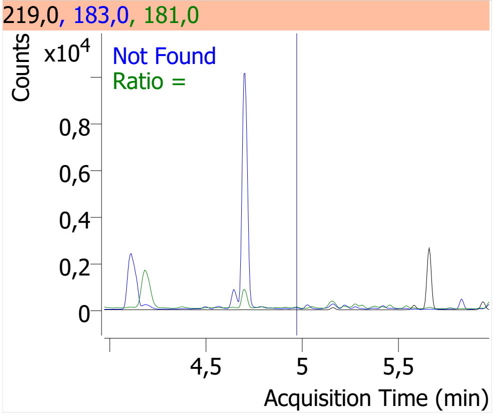

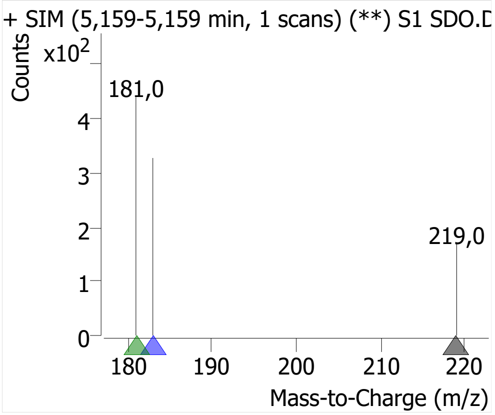

Agilent Trusted Answers

Hexachlorobenzene

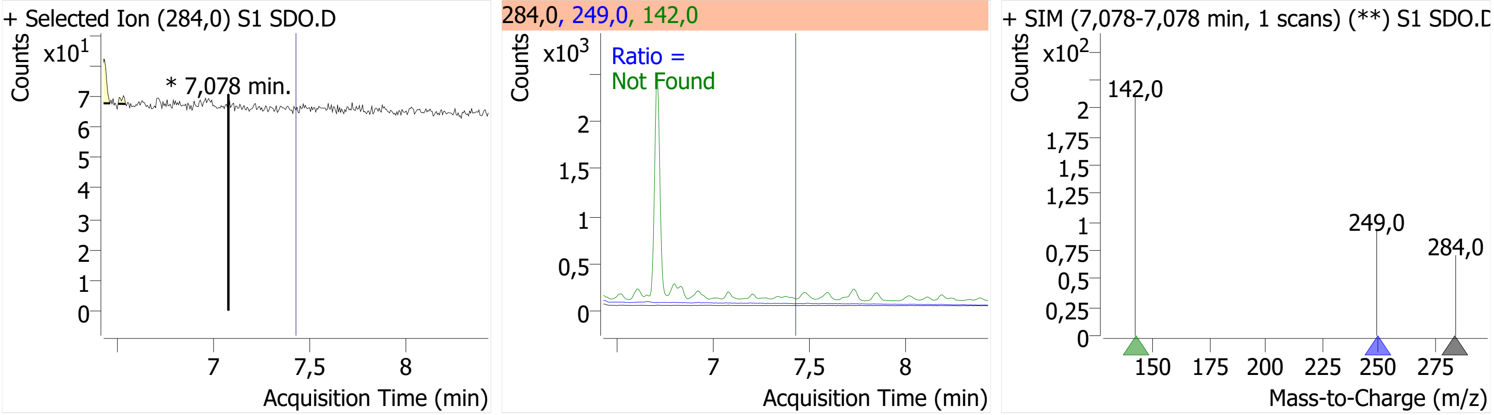

Aldrine

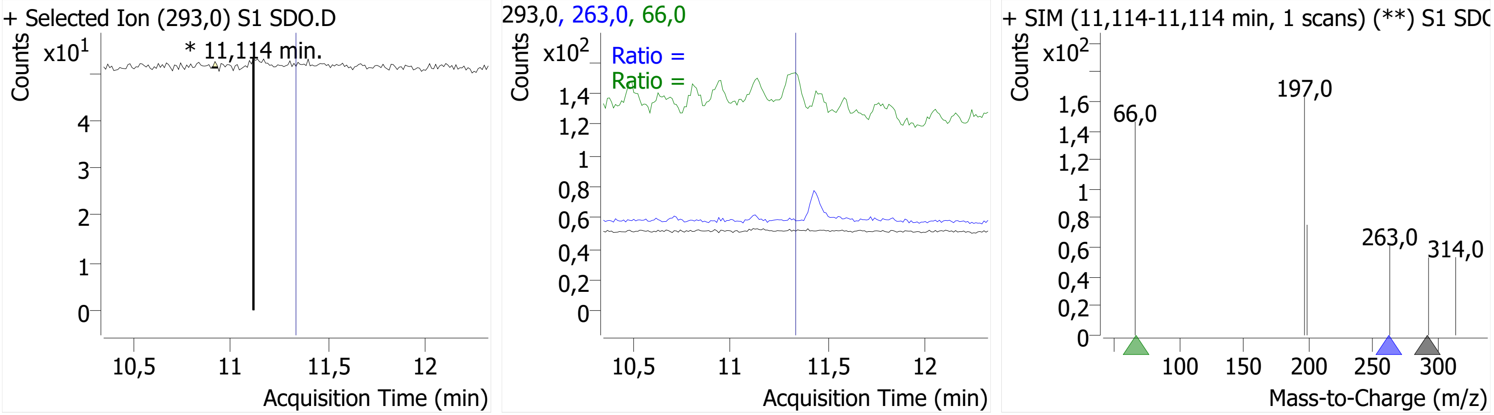

Chlorpyrifos

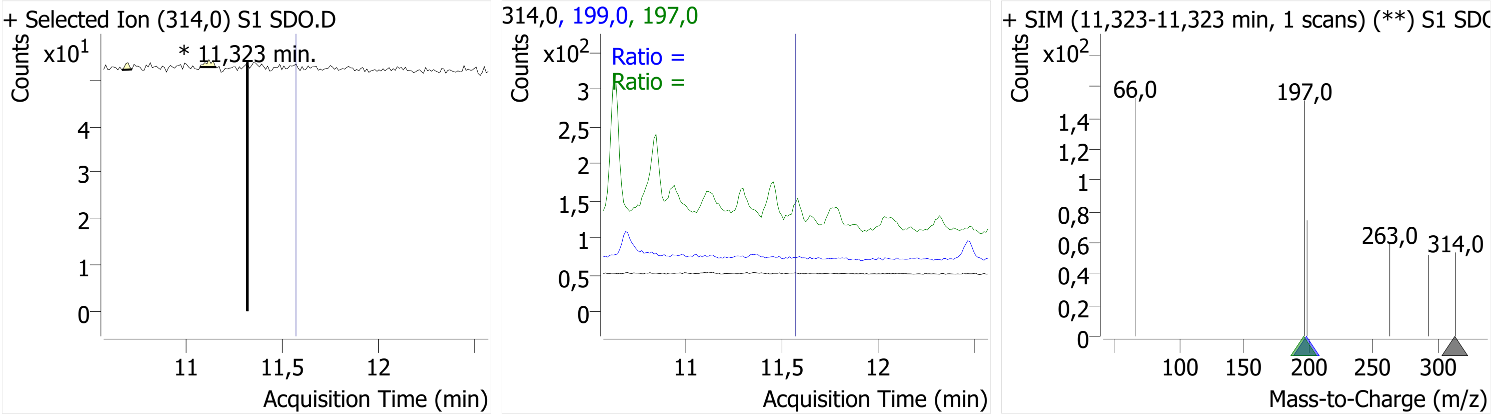

Endosulfan II

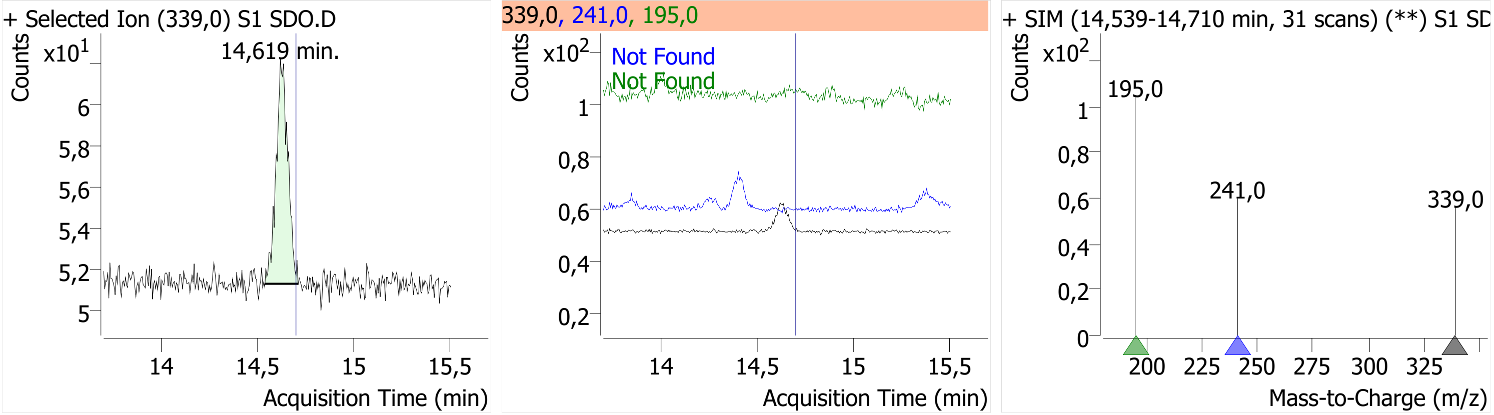

Agilent Trusted Answers

4,4-DDE

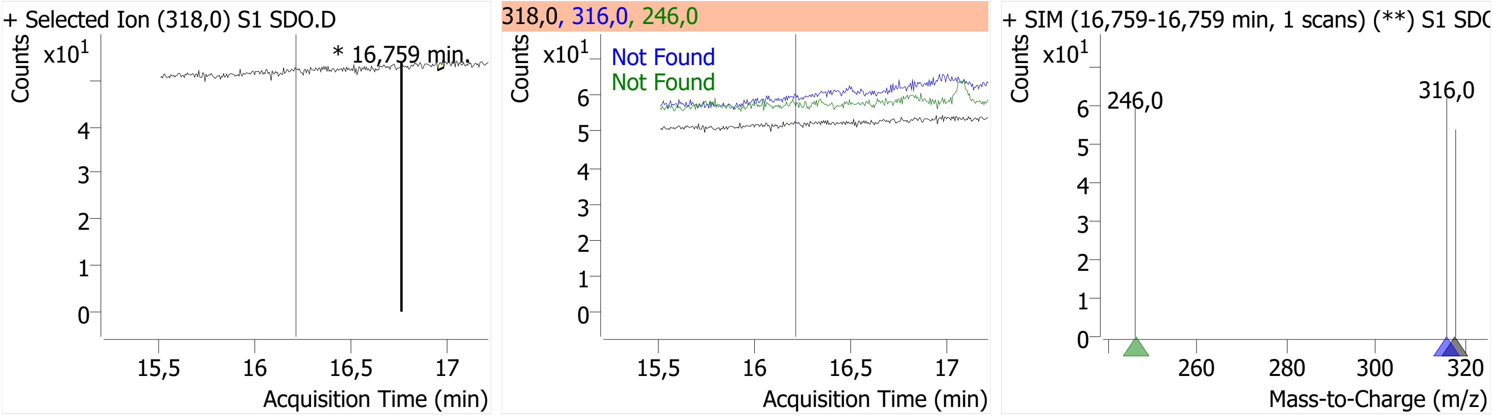

4,4-DDD

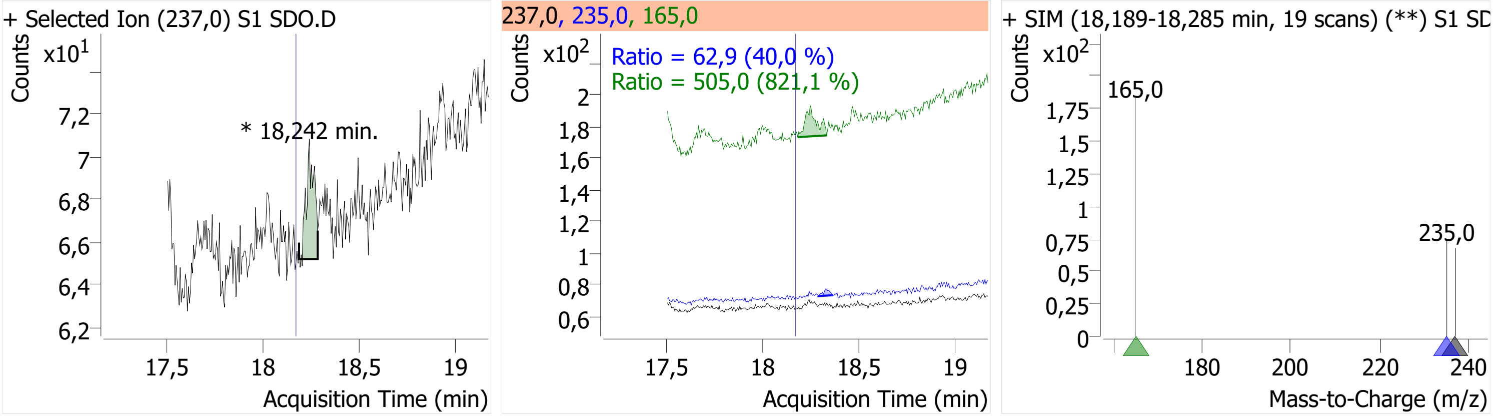

4,4-DDT

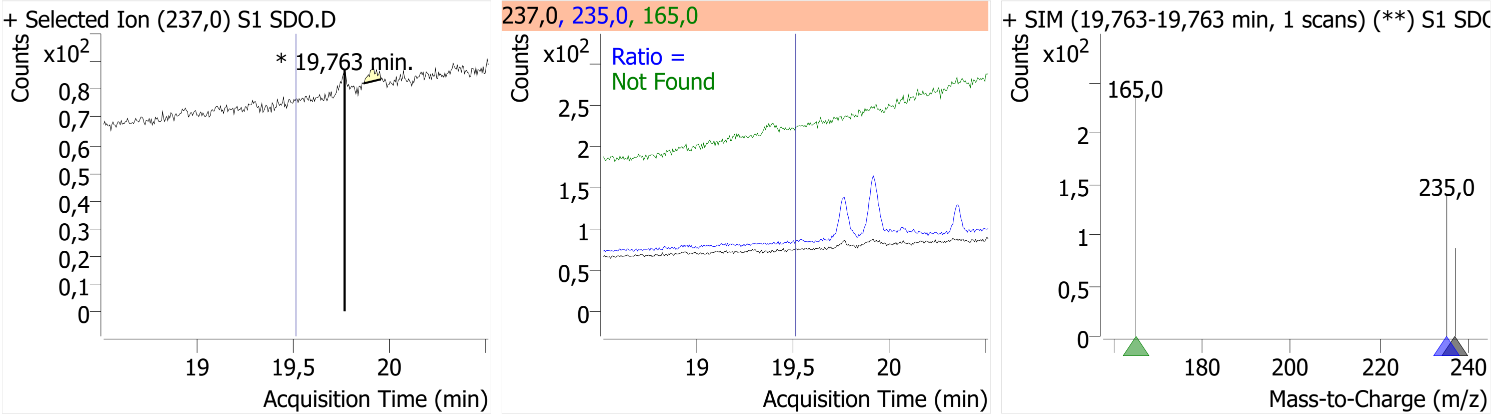

 Generated at 11:37 on 13 Dec 2024

Quantitative Analysis Complete Report

**Batch Path**	C:\Users\USER\OneDrive\Desktop\JU_Pesticide\MA\MA\QuantResults\Mohammed_Pest.batch.bin		
**Analysis Time**	13 Dec 2024 11:34	**Analyst Name**	DESKTOP-MRRPPC4\USER
**Report Time**	13 Dec 2024 11:37:33	**Reporter Name**	DESKTOP-MRRPPC4\USER
**Last Calib Update**	13 Dec 2024 11:34	**Batch State**	Processed
**Quant Batch Version**	10.0	**Quant Report Version**	10.0

**Acq. Time**	13 Nov 2024 02:06	**Data File**	S1 SDP.D
**Sample Type**	Sample	**Sample Name**	Pest
**Dilution**	1	**Acq. Method**	pesticide std 12.11.2024

Sample Chromatogram

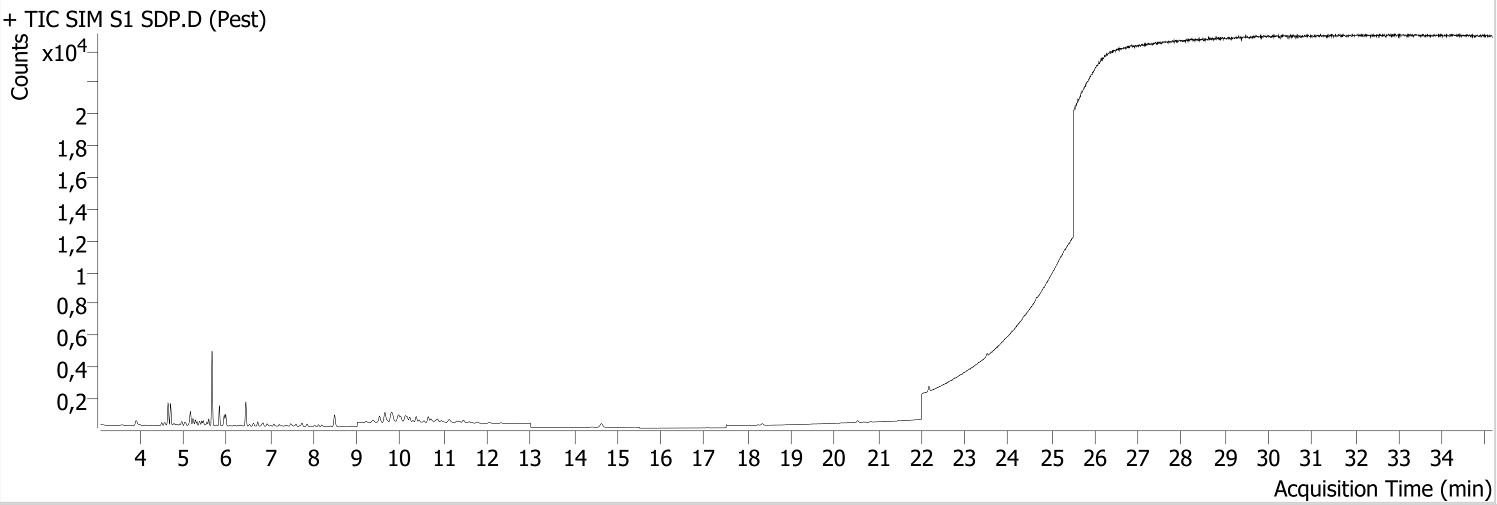

Compound	Transition	RT	Resp.	Final Conc	Units
Lindane	219,0	4,955	109	162,0754	ng/ml
Hexachlorobenzene	284,0	6,438	0	ND	ng/ml
Aldrine	293,0	11,125	0	ND	ng/ml
Chlorpyrifos	314,0	11,125	0	ND	ng/ml
Endosulfan II	339,0	14,630	0	ND	ng/ml
4,4-DDE	318,0			ND	ng/ml
4,4-DDD	237,0	18,226	19	4,8826	ng/ml
4,4-DDT	237,0	19,269	0	ND	ng/ml

Lindane

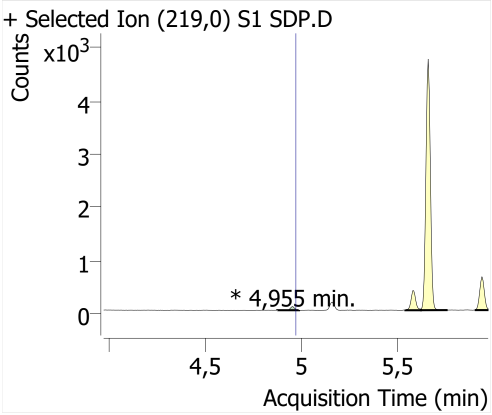

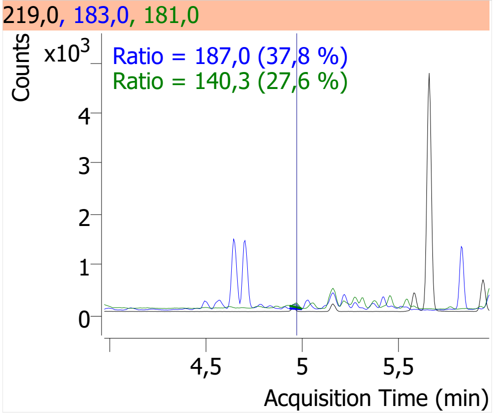

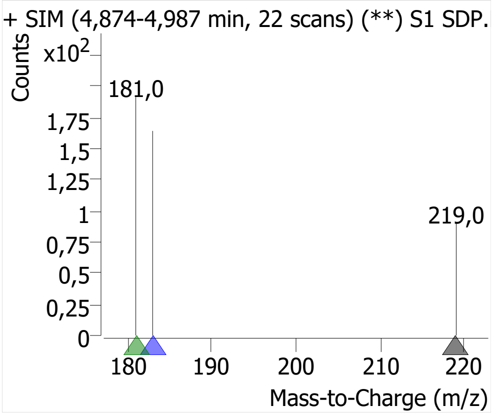

Hexachlorobenzene

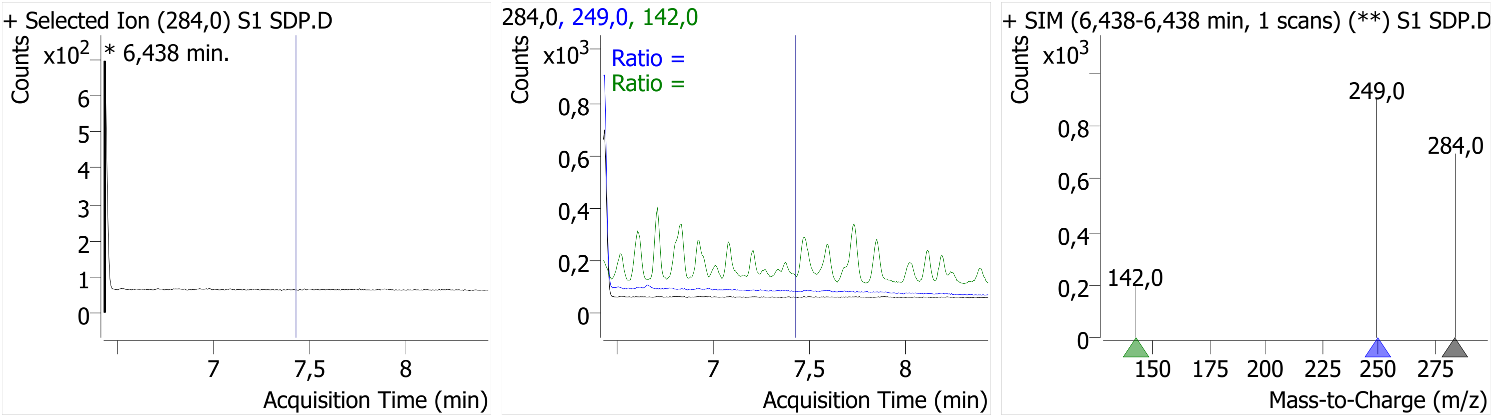

Aldrine

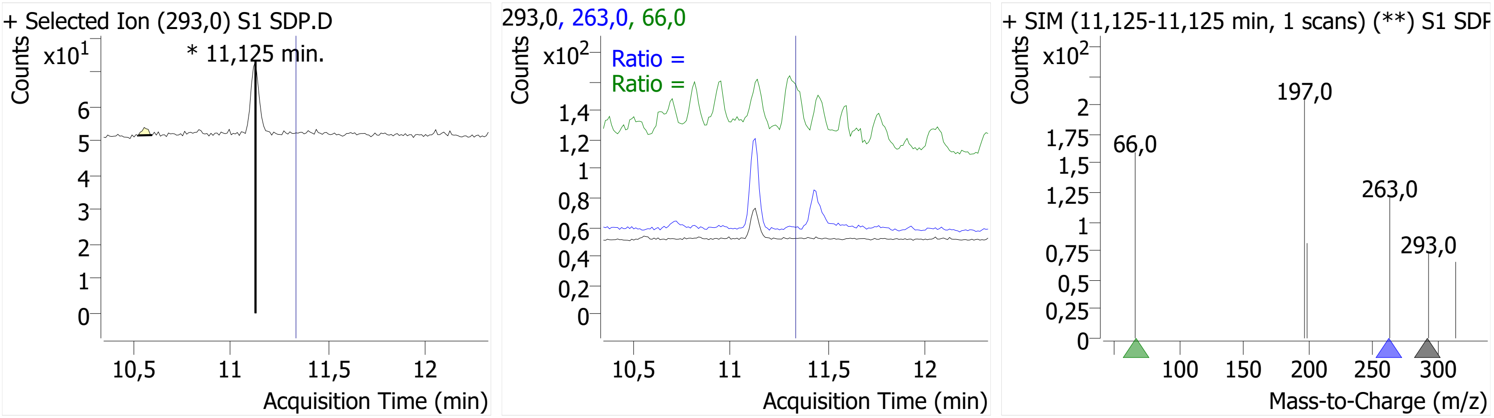

Chlorpyrifos

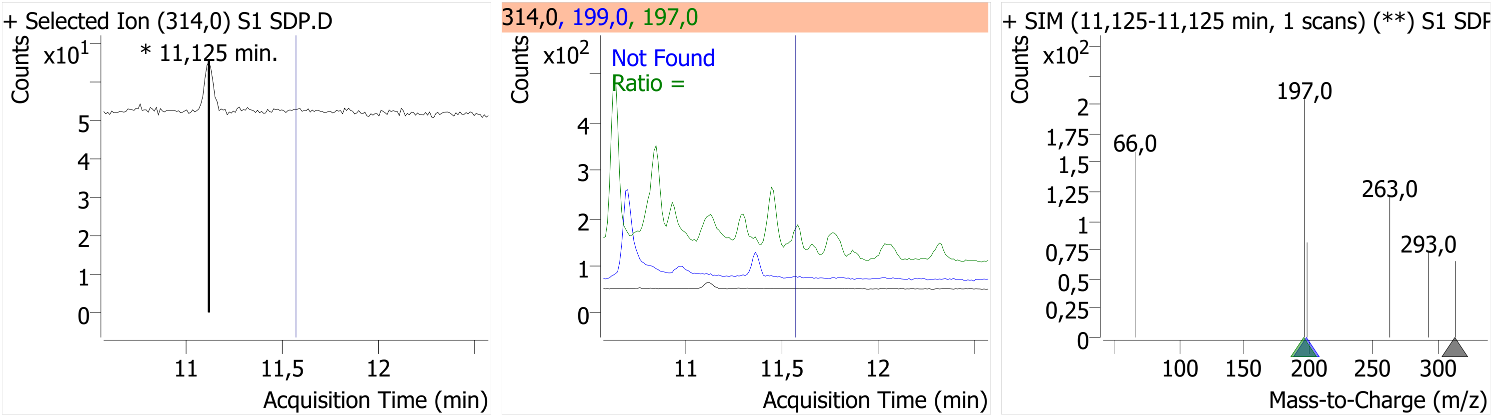

Endosulfan II

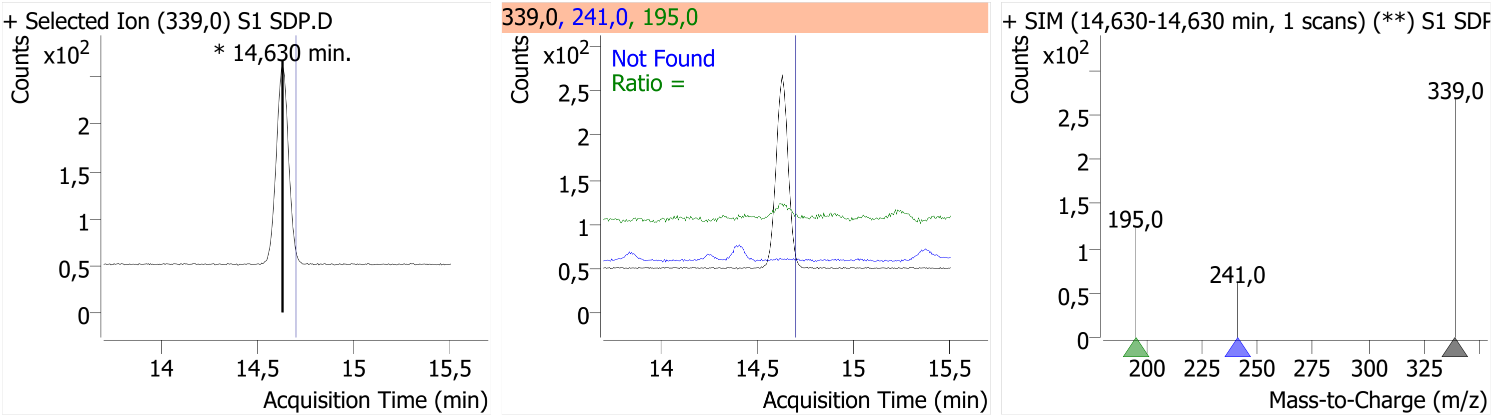

4,4-DDE

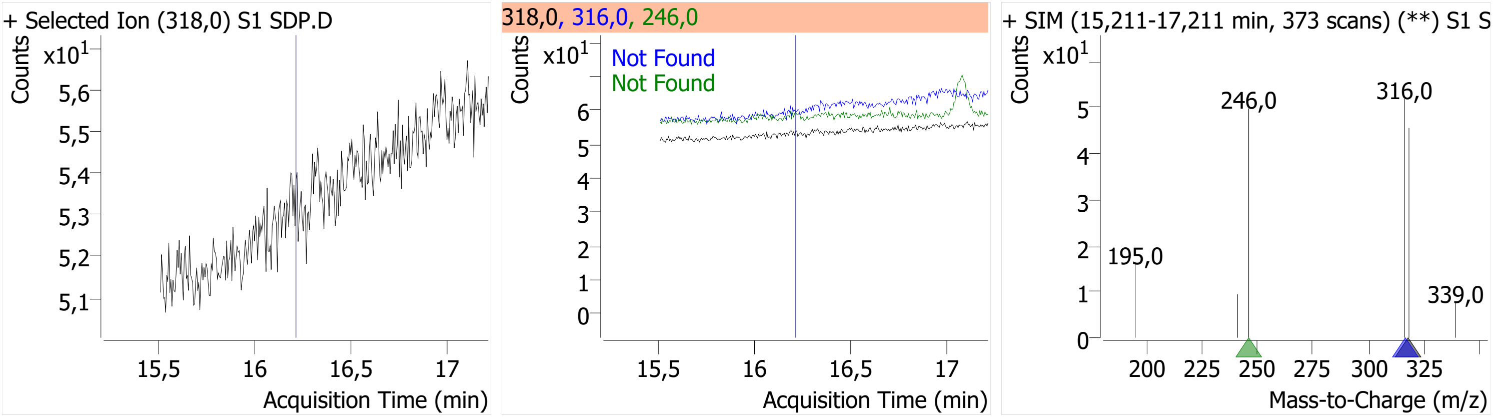

4,4-DDD

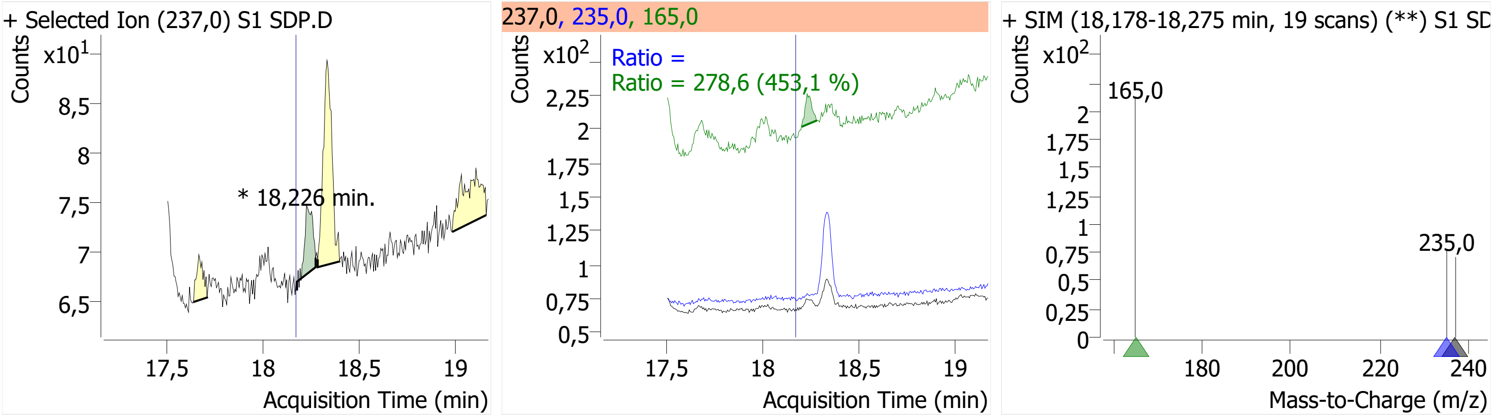

4,4-DDT

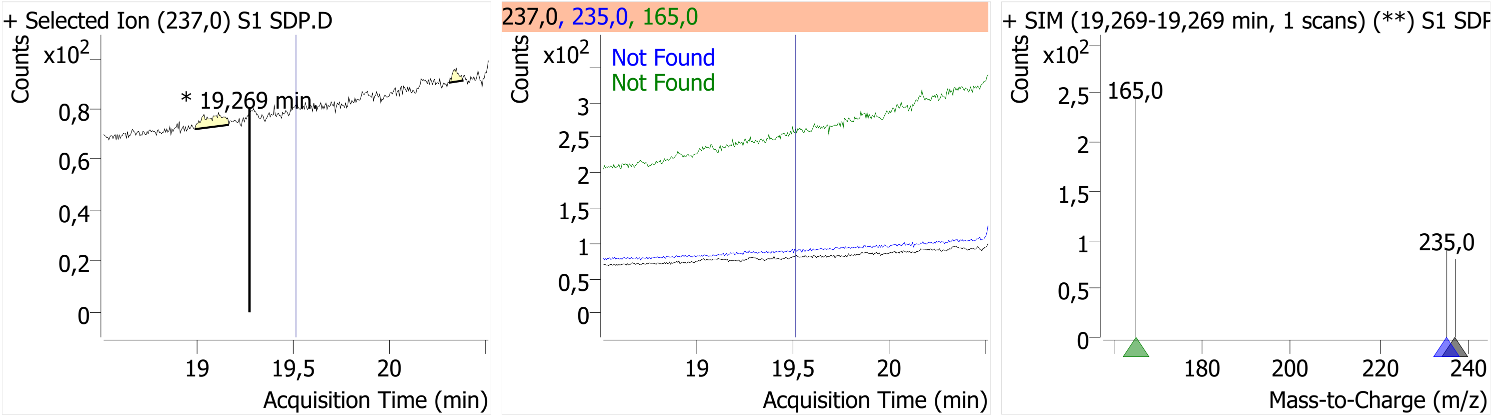

Quantitative Analysis Complete Report

**Batch Path**	C:\Users\USER\OneDrive\Desktop\JU_Pesticide\MA\MA\QuantResults\Mohammed_Pest.batch.bin		
**Analysis Time**	13 Dec 2024 11:34	**Analyst Name**	DESKTOP-MRRPPC4\USER
**Report Time**	13 Dec 2024 11:37:34	**Reporter Name**	DESKTOP-MRRPPC4\USER
**Last Calib Update**	13 Dec 2024 11:34	**Batch State**	Processed
**Quant Batch Version**	10.0	**Quant Report Version**	10.0

**Acq. Time**	13 Nov 2024 02:47	**Data File**	S2 DWP.D
**Sample Type**	Sample	**Sample Name**	Pest
**Dilution**	1	**Acq. Method**	pesticide std 12.11.2024

Sample Chromatogram

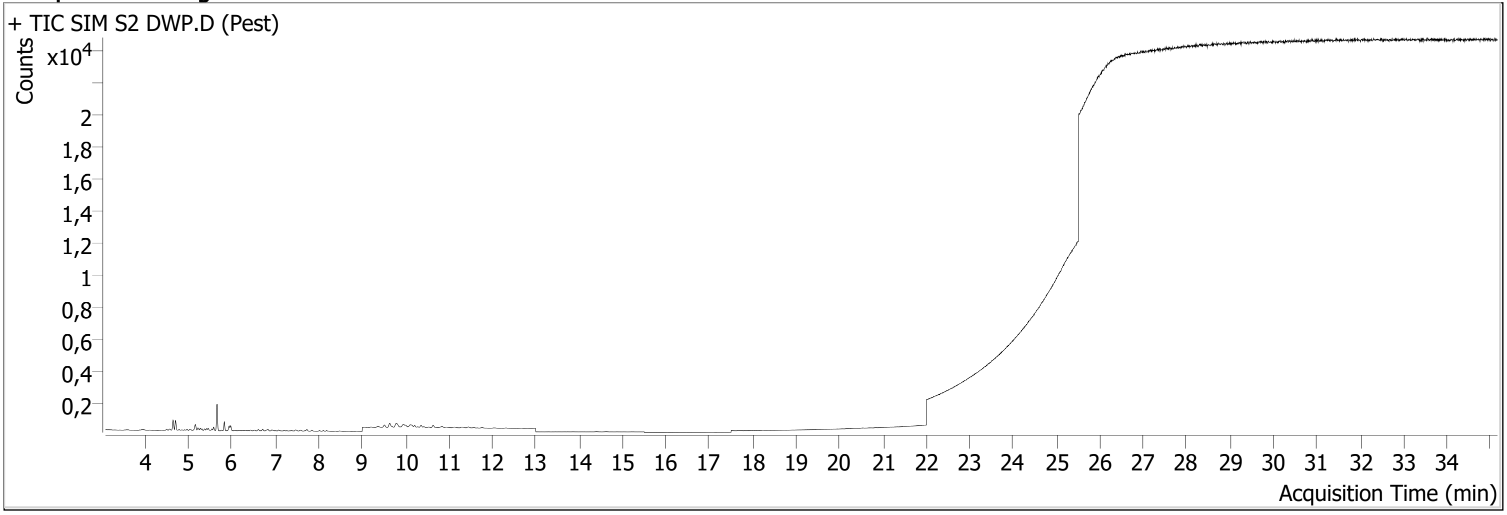

Compound	Transition	RT	Resp.	Final Conc	Units
Lindane	219,0	5,159	0	ND	ng/ml
Hexachlorobenzene	284,0	7,746	0	ND	ng/ml
Aldrine	293,0	11,469	0	ND	ng/ml
Chlorpyrifos	314,0	11,522	0	ND	ng/ml
Endosulfan II	339,0	14,625	40	34,5661	ng/ml
4,4-DDE	318,0	16,201	0	ND	ng/ml
4,4-DDD	237,0	18,237	8	2,8902	ng/ml
4,4-DDT	237,0			ND	ng/ml

Lindane

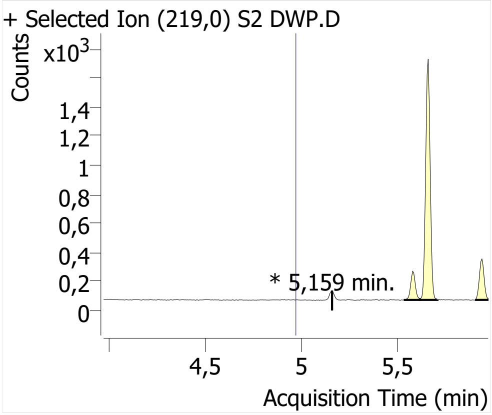

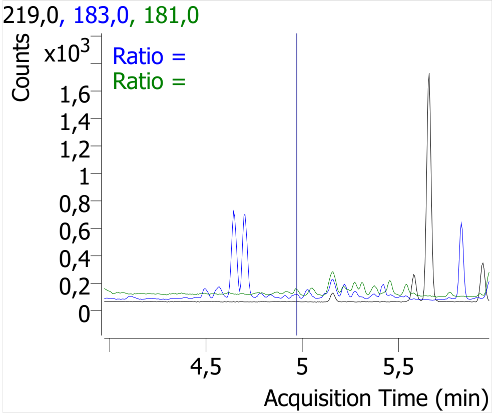

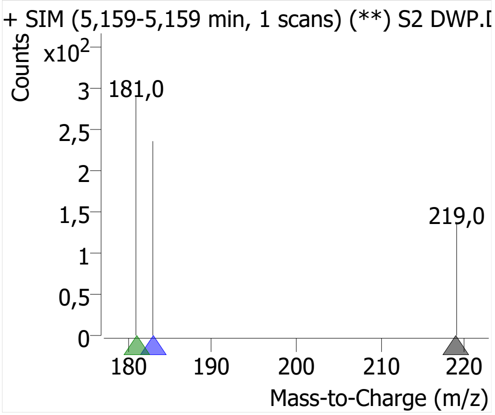

Hexachlorobenzene

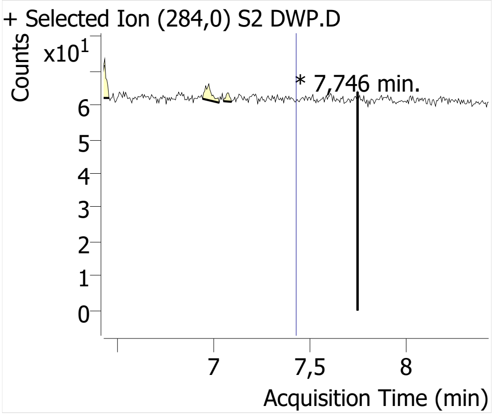

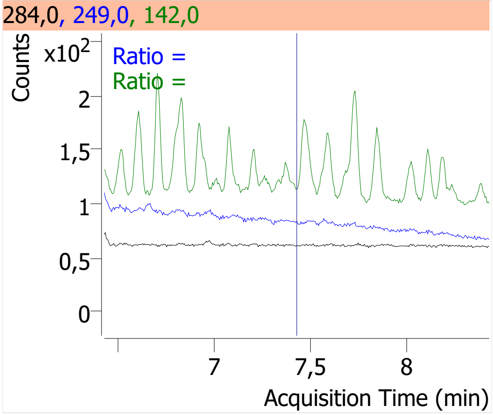

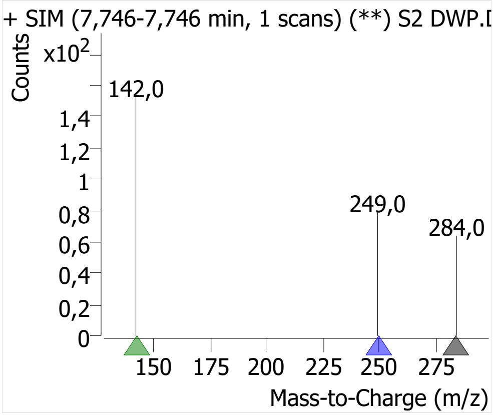

Aldrine

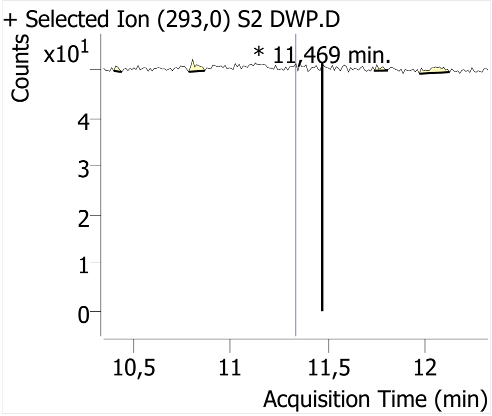

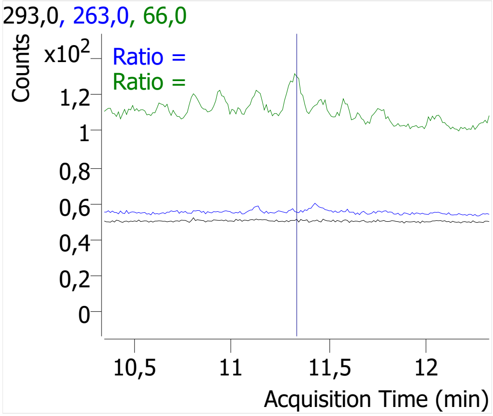

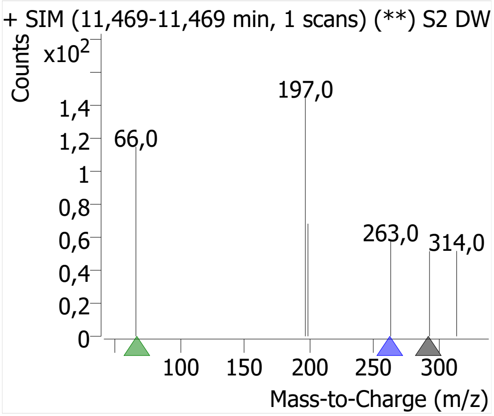

Chlorpyrifos

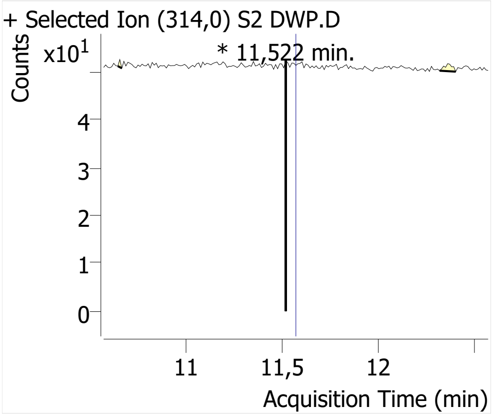

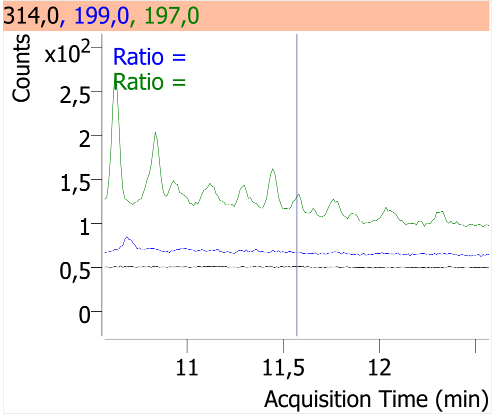

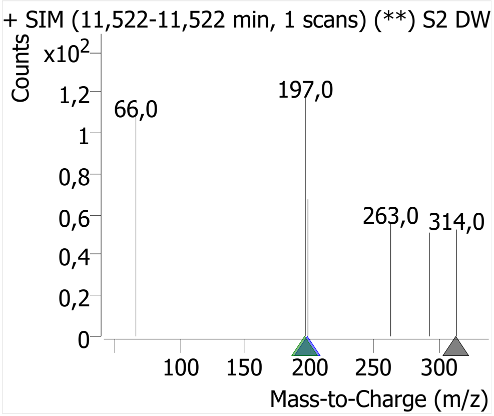

Endosulfan II

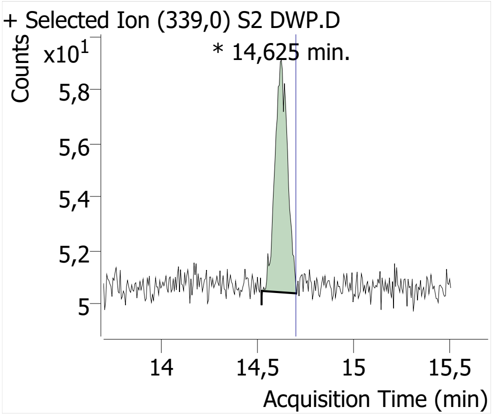

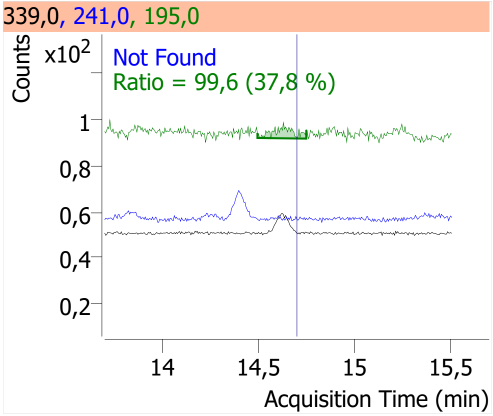

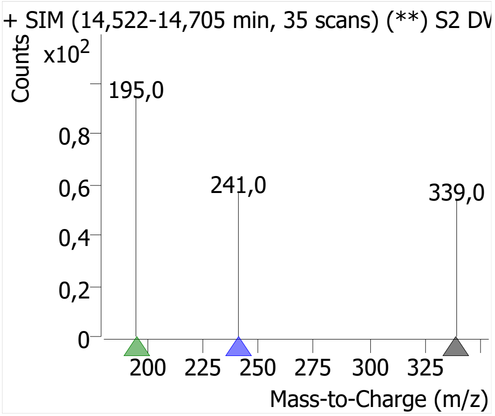

4,4-DDE

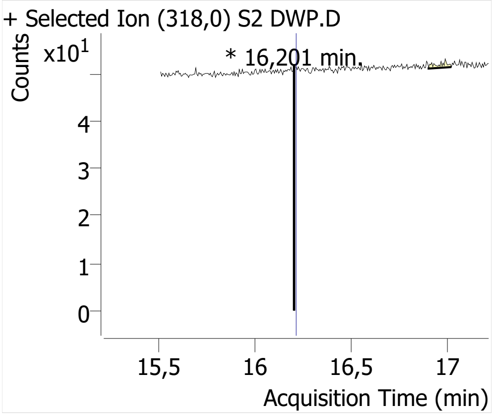

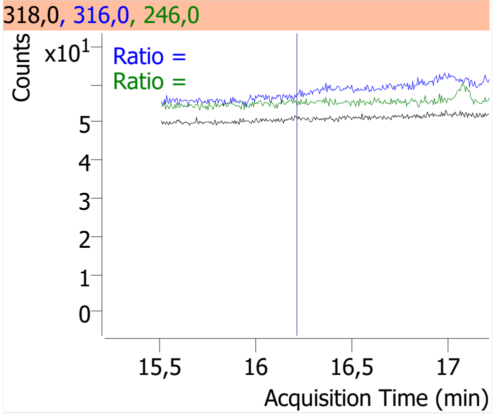

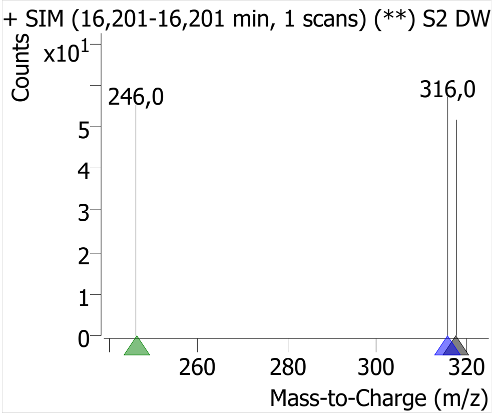

4,4-DDD

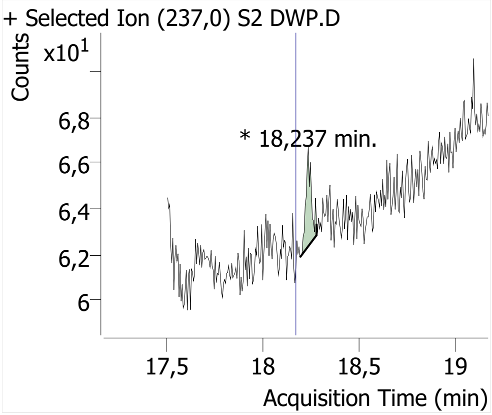

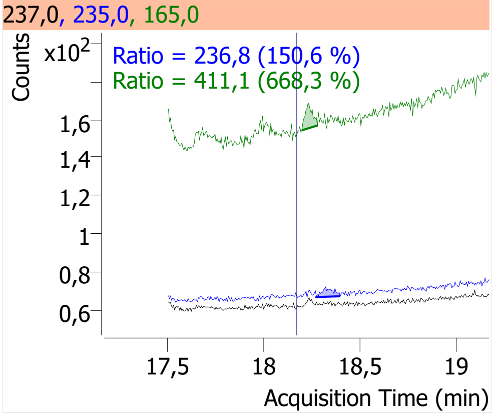

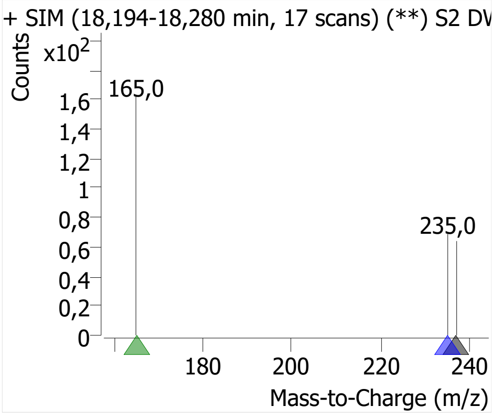

4,4-DDT

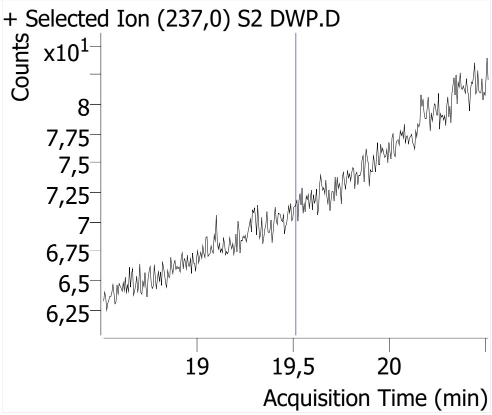

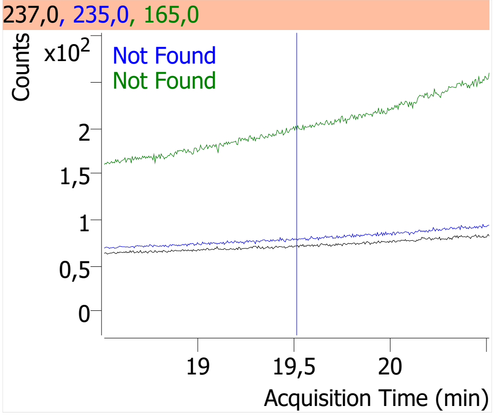

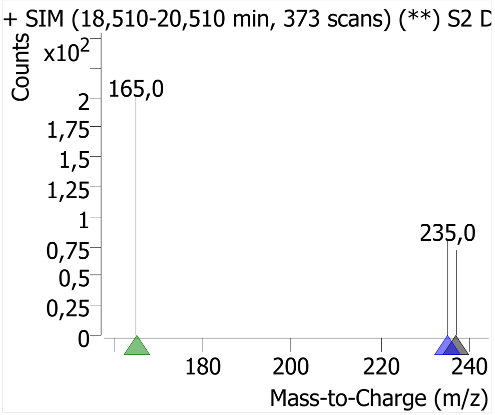

Quantitative Analysis Complete Report

**Batch Path**	C:\Users\USER\OneDrive\Desktop\JU_Pesticide\MA\MA\QuantResults\Mohammed_Pest.batch.bin		
**Analysis Time**	13 Dec 2024 11:34	**Analyst Name**	DESKTOP-MRRPPC4\USER
**Report Time**	13 Dec 2024 11:37:35	**Reporter Name**	DESKTOP-MRRPPC4\USER
**Last Calib Update**	13 Dec 2024 11:34	**Batch State**	Processed
**Quant Batch Version**	10.0	**Quant Report Version**	10.0

**Acq. Time**	13 Nov 2024 03:27	**Data File**	S1 DOP.D
**Sample Type**	Sample	**Sample Name**	Pest
**Dilution**	1	**Acq. Method**	pesticide std 12.11.2024

Sample Chromatogram

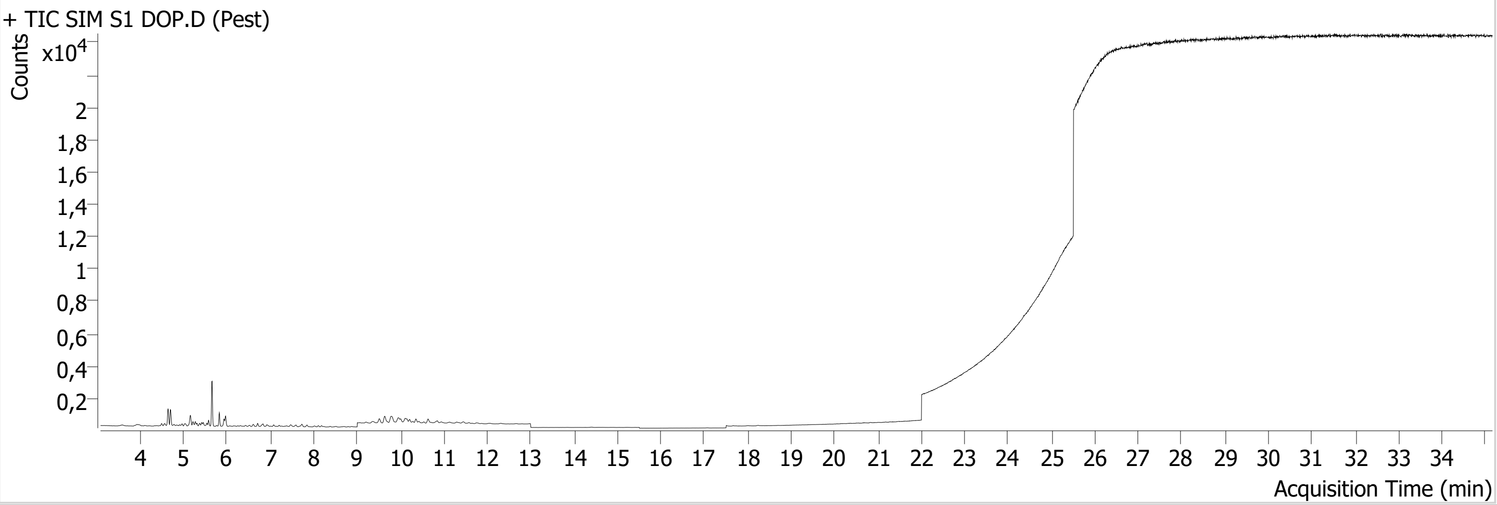

Compound	Transition	RT	Resp.	Final Conc	Units
Lindane	219,0	5,159	0	ND	ng/ml
Hexachlorobenzene	284,0	6,965	0	ND	ng/ml
Aldrine	293,0	11,323	0	ND	ng/ml
Chlorpyrifos	314,0	10,916	0	ND	ng/ml
Endosulfan II	339,0	14,625	8	2,0207	ng/ml
4,4-DDE	318,0	16,985	0	ND	ng/ml
4,4-DDD	237,0	18,237	26	6,1581	ng/ml
4,4-DDT	237,0			ND	ng/ml

Lindane

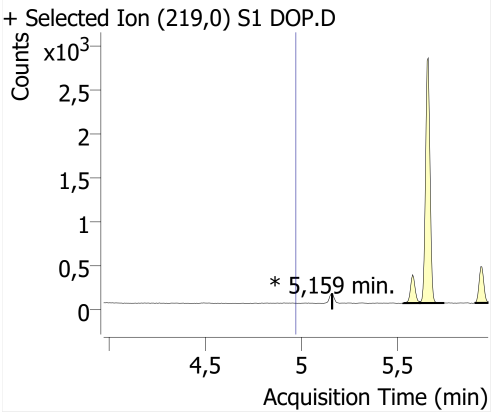

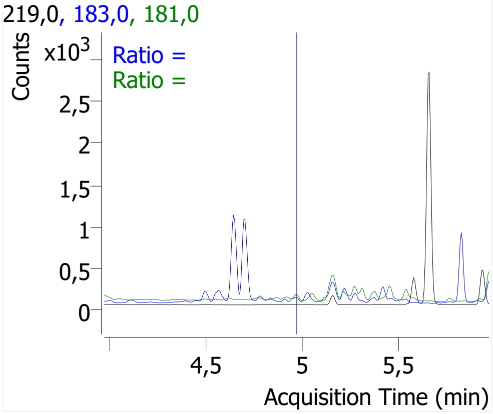

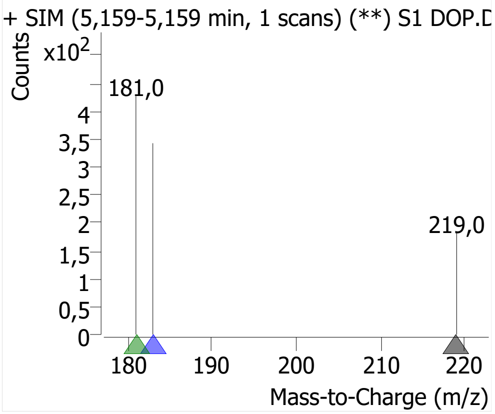

Hexachlorobenzene

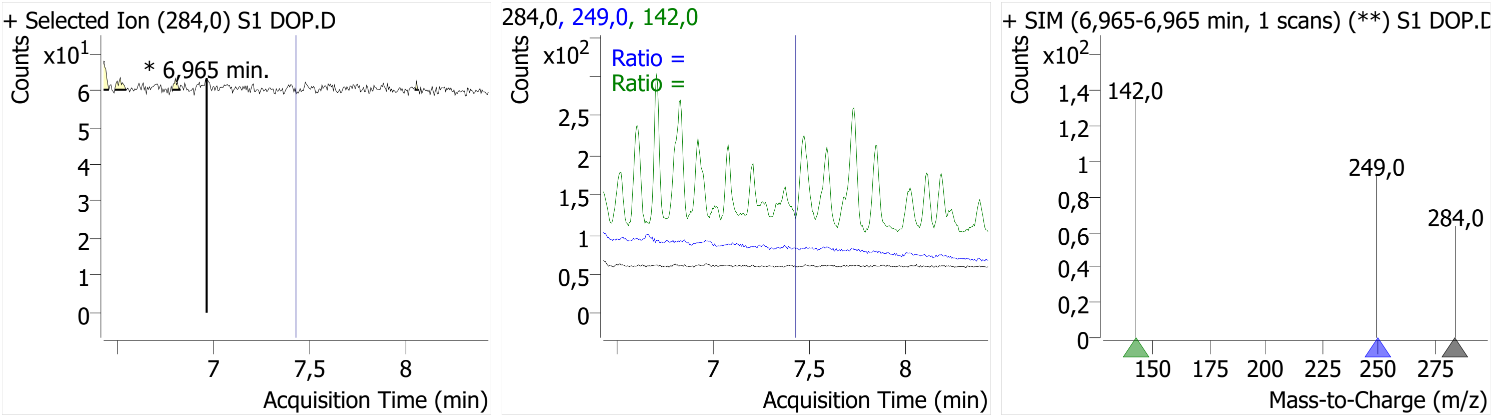

Aldrine

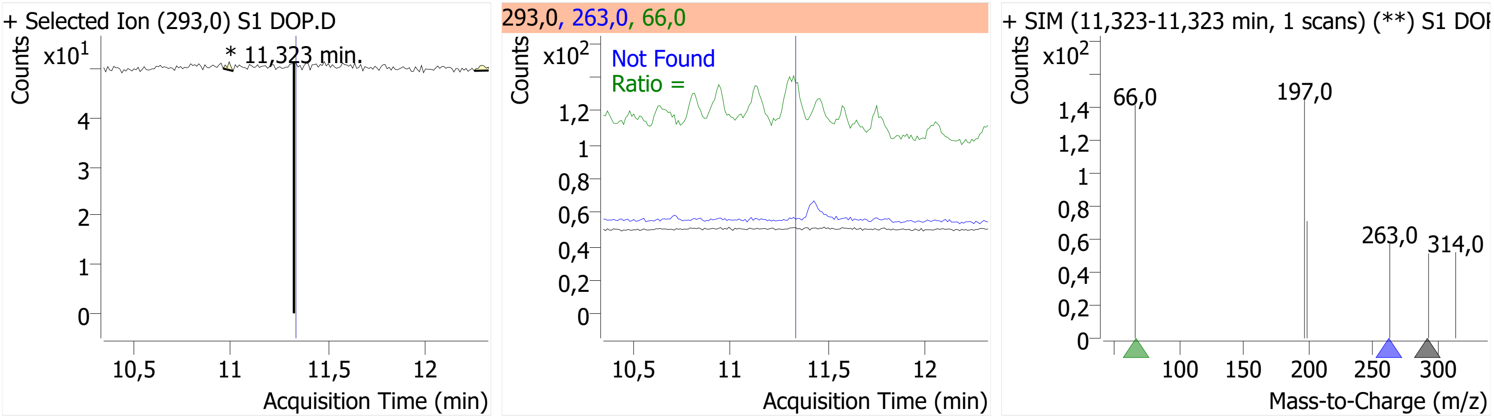

Chlorpyrifos

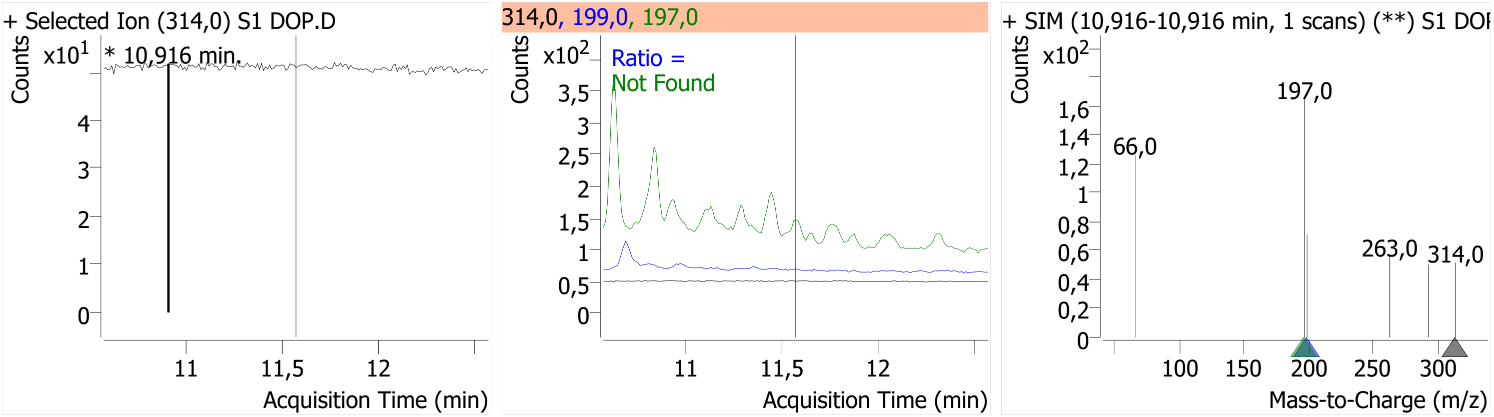

Endosulfan II

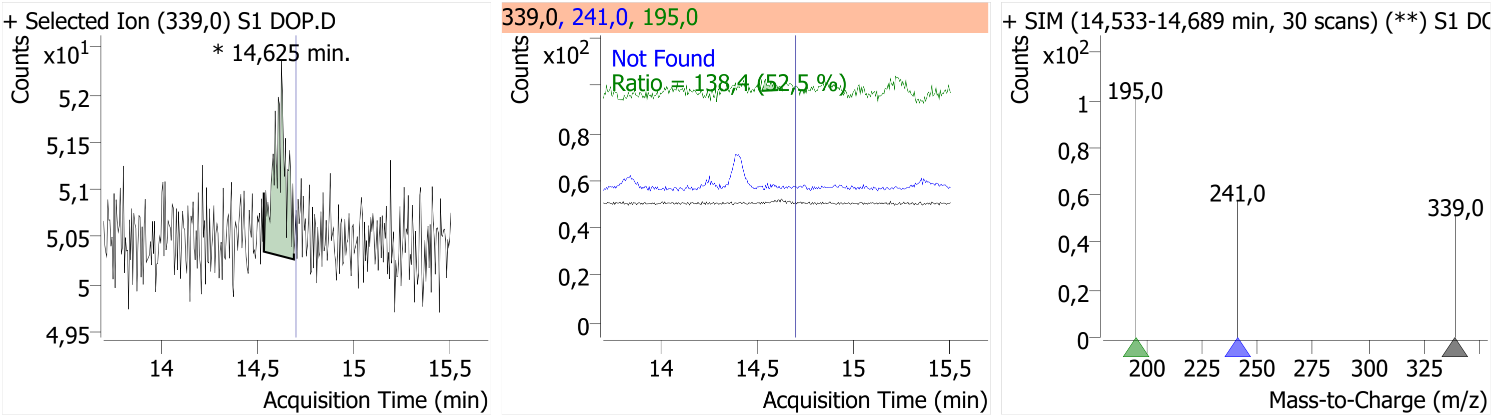

Agilent Trusted Answers

4,4-DDE

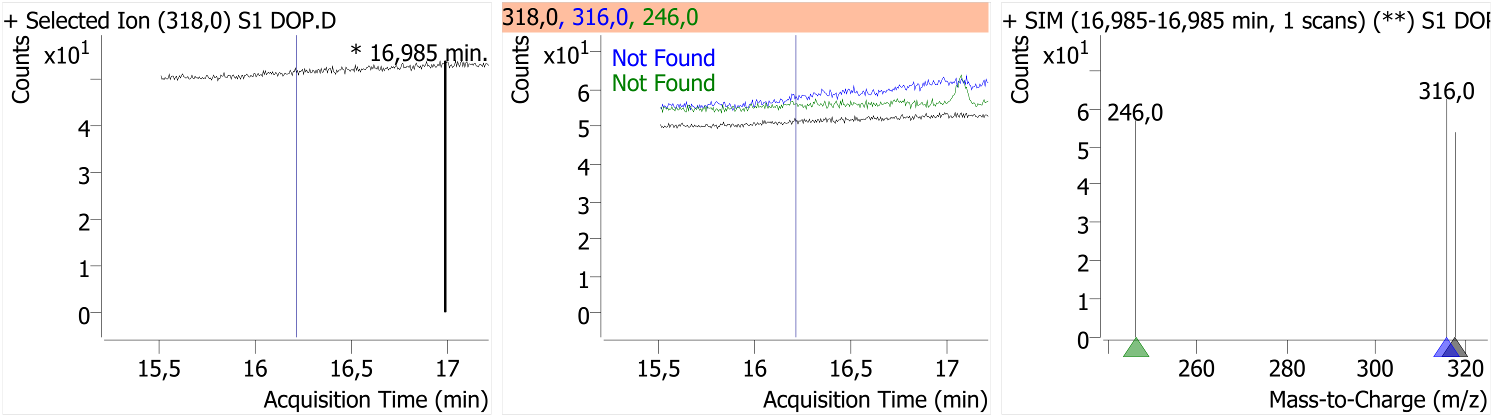

4,4-DDD

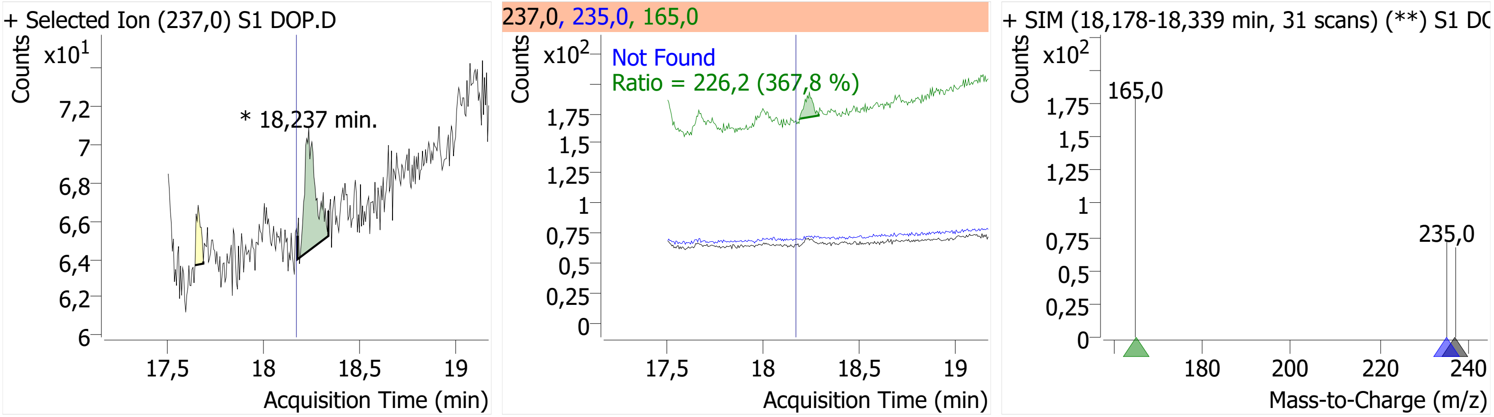

4,4-DDT

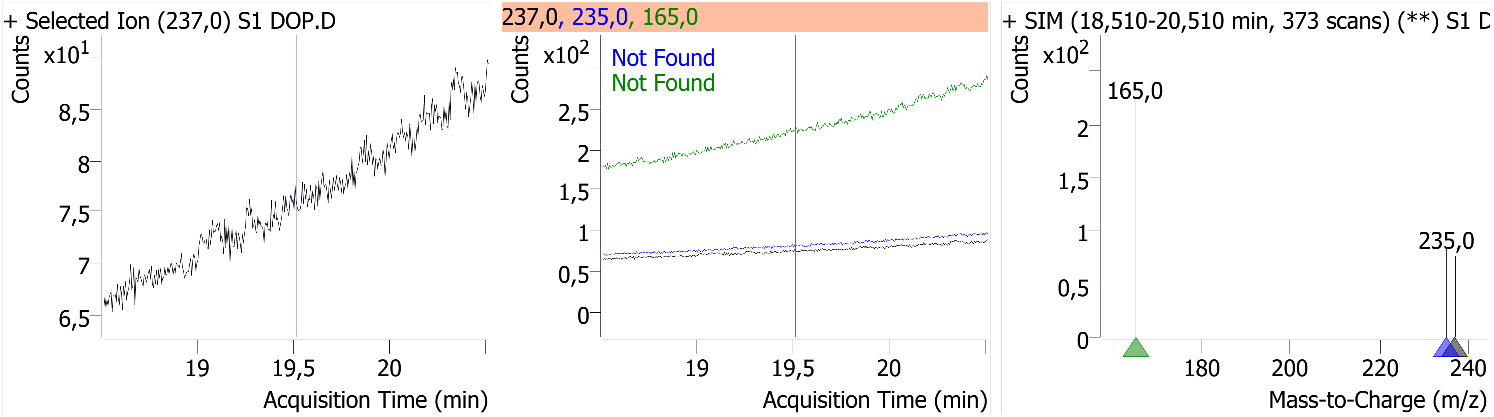

Quantitative Analysis Complete Report

**Batch Path**	C:\Users\USER\OneDrive\Desktop\JU_Pesticide\MA\MA\QuantResults\Mohammed_Pest.batch.bin		
**Analysis Time**	13 Dec 2024 11:34	**Analyst Name**	DESKTOP-MRRPPC4\USER
**Report Time**	13 Dec 2024 11:37:36	**Reporter Name**	DESKTOP-MRRPPC4\USER
**Last Calib Update**	13 Dec 2024 11:34	**Batch State**	Processed
**Quant Batch Version**	10.0	**Quant Report Version**	10.0

**Acq. Time**	13 Nov 2024 04:08	**Data File**	S2 SUP.D
**Sample Type**	Sample	**Sample Name**	Pest
**Dilution**	1	**Acq. Method**	pesticide std 12.11.2024

Sample Chromatogram

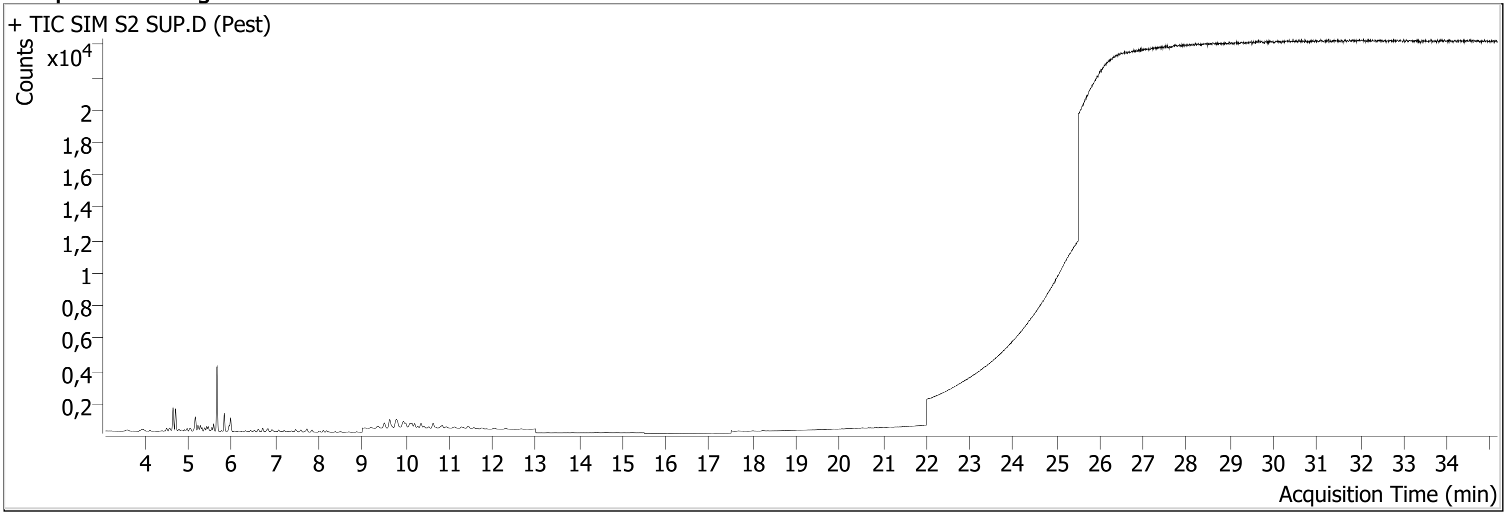

Compound	Transition	RT	Resp.	Final Conc	Units
Lindane	219,0	5,159	0	ND	ng/ml
Hexachlorobenzene	284,0	7,078	0	ND	ng/ml
Aldrine	293,0	11,334	0	ND	ng/ml
Chlorpyrifos	314,0	11,135	0	ND	ng/ml
Endosulfan II	339,0	14,624	37	32,0586	ng/ml
4,4-DDE	318,0			ND	ng/ml
4,4-DDD	237,0	18,248	22	5,3525	ng/ml
4,4-DDT	237,0	19,854	0	ND	ng/ml

Lindane

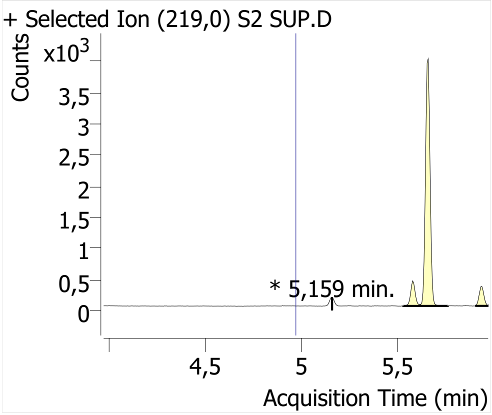

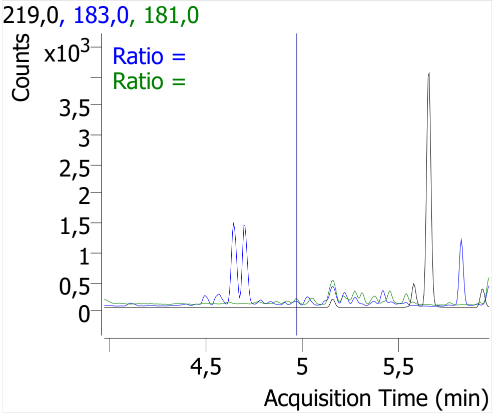

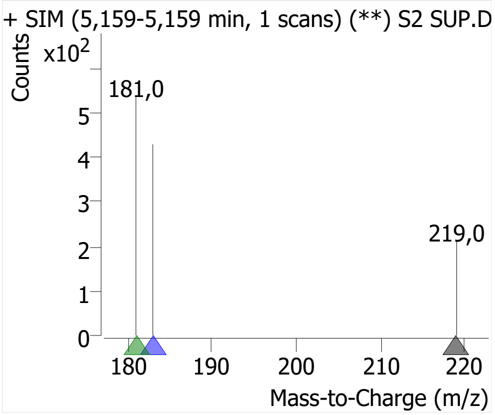

Hexachlorobenzene

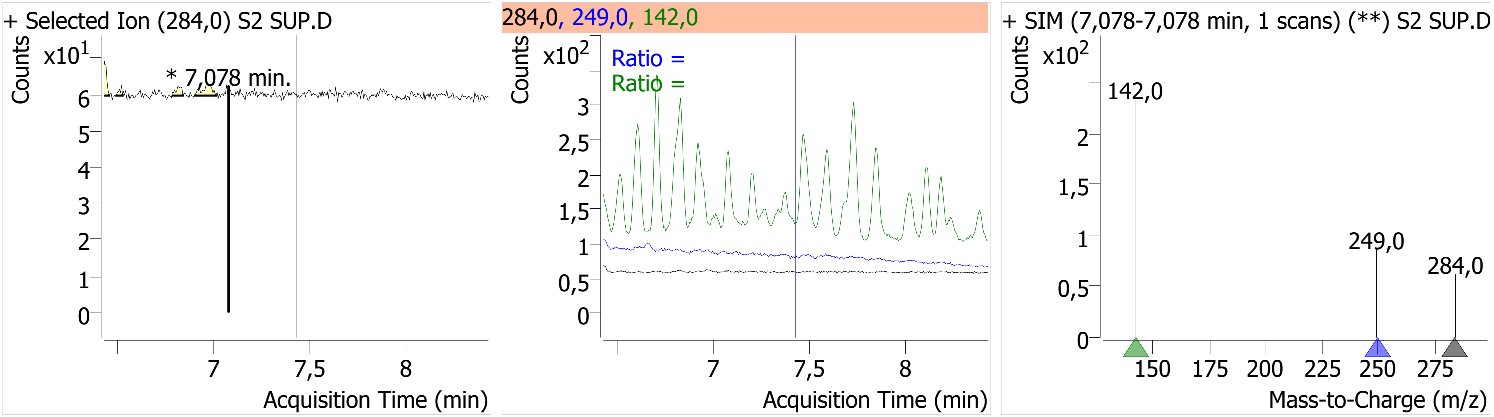

Aldrine

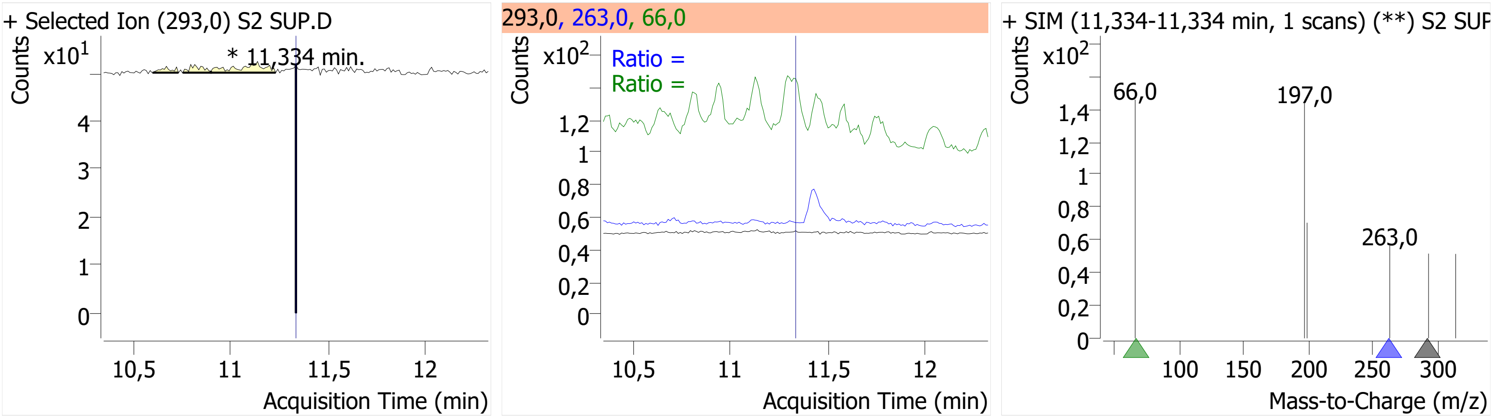

Chlorpyrifos

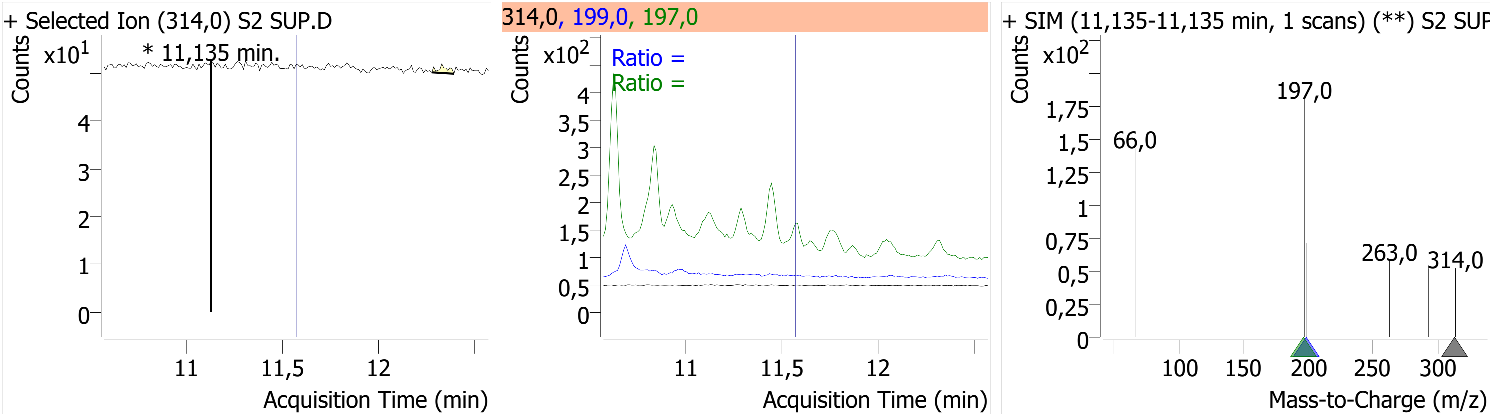

Endosulfan II

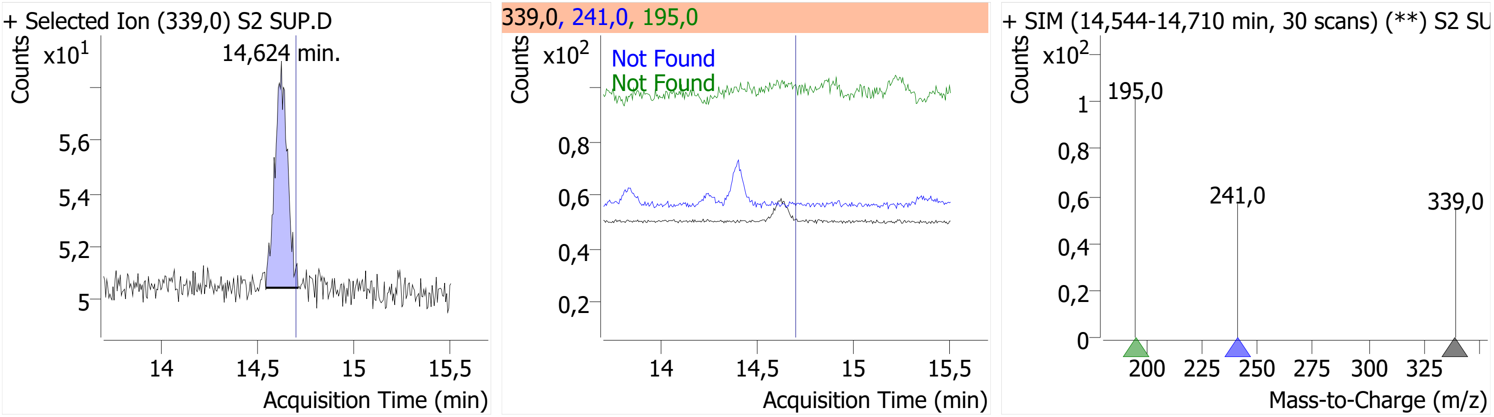

4,4-DDE

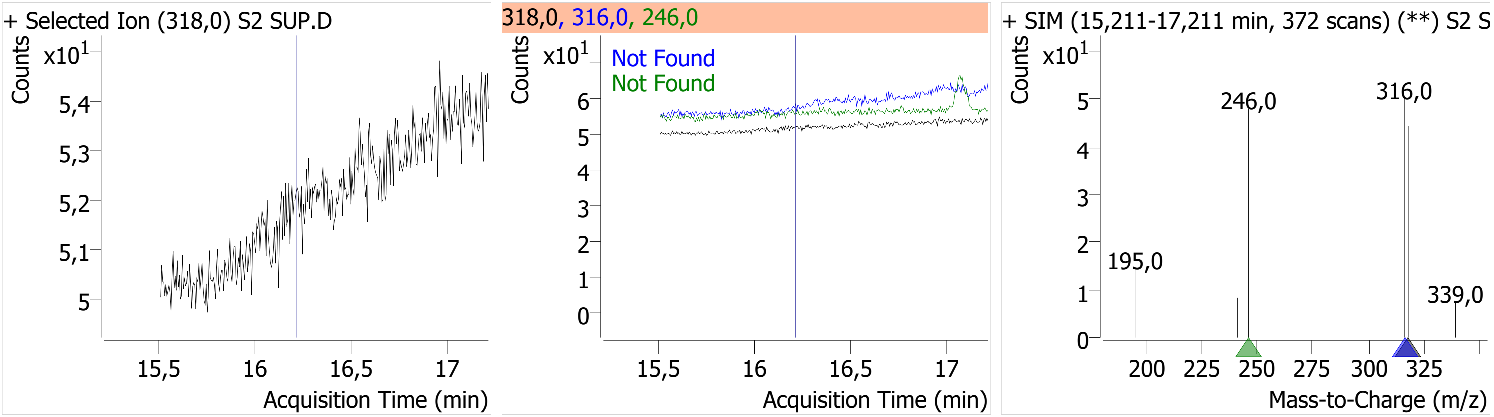

4,4-DDD

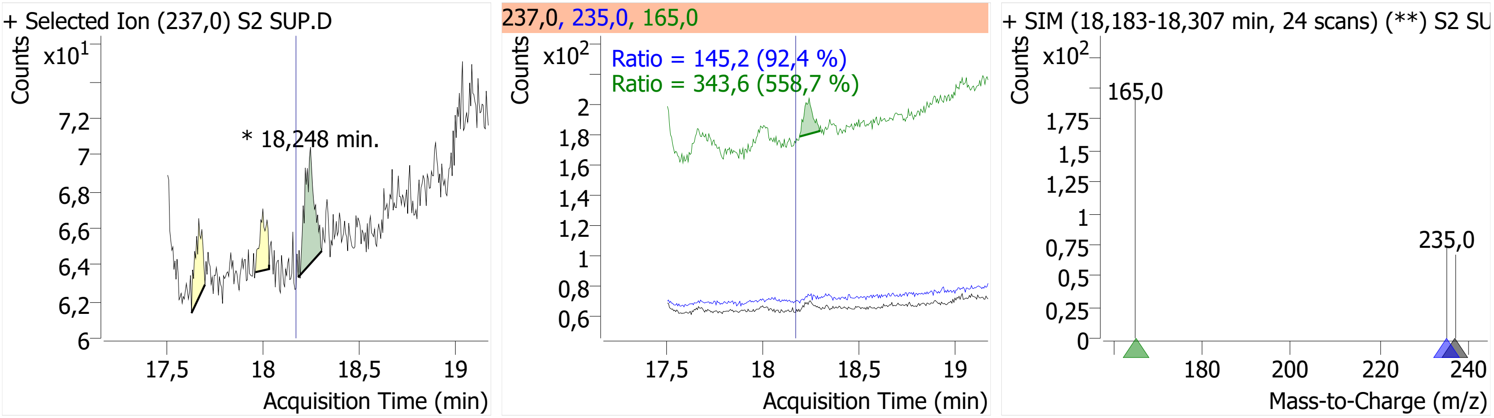

4,4-DDT

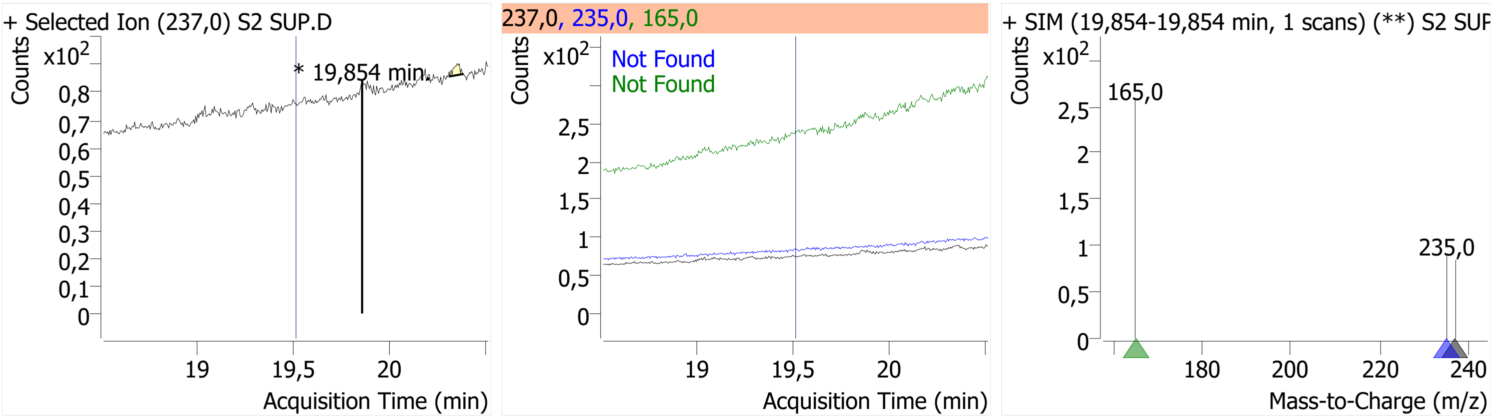

Quantitative Analysis Complete Report

**Batch Path**	C:\Users\USER\OneDrive\Desktop\JU_Pesticide\MA\MA\QuantResults\Mohammed_Pest.batch.bin		
**Analysis Time**	13 Dec 2024 11:34	**Analyst Name**	DESKTOP-MRRPPC4\USER
**Report Time**	13 Dec 2024 11:37:37	**Reporter Name**	DESKTOP-MRRPPC4\USER
**Last Calib Update**	13 Dec 2024 11:34	**Batch State**	Processed
**Quant Batch Version**	10.0	**Quant Report Version**	10.0

**Acq. Time**	13 Nov 2024 04:49	**Data File**	S1 DOP-1.D
**Sample Type**	Sample	**Sample Name**	Pest
**Dilution**	1	**Acq. Method**	pesticide std 12.11.2024

Sample Chromatogram

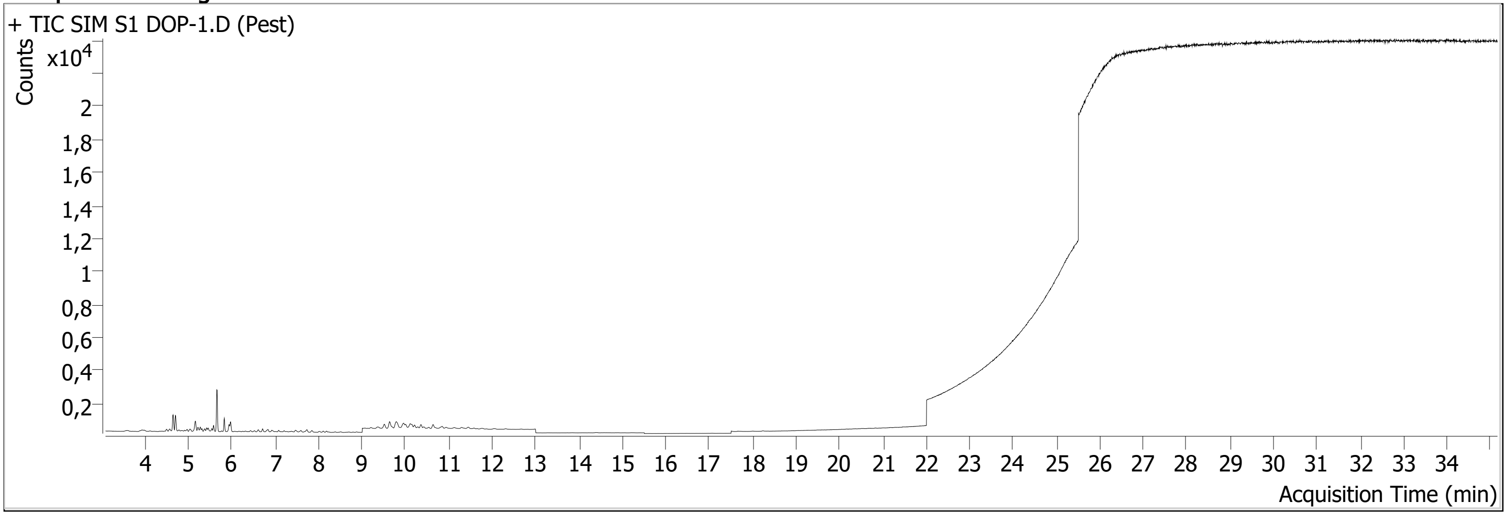

Compound	Transition	RT	Resp.	Final Conc	Units
Lindane	219,0	5,154	0	ND	ng/ml
Hexachlorobenzene	284,0	7,073	0	ND	ng/ml
Aldrine	293,0	11,292	0	ND	ng/ml
Chlorpyrifos	314,0			ND	ng/ml
Endosulfan II	339,0	14,630	8	2,2285	ng/ml
4,4-DDE	318,0	16,195	0	ND	ng/ml
4,4-DDD	237,0	18,237	15	4,1526	ng/ml
4,4-DDT	237,0			ND	ng/ml

Lindane

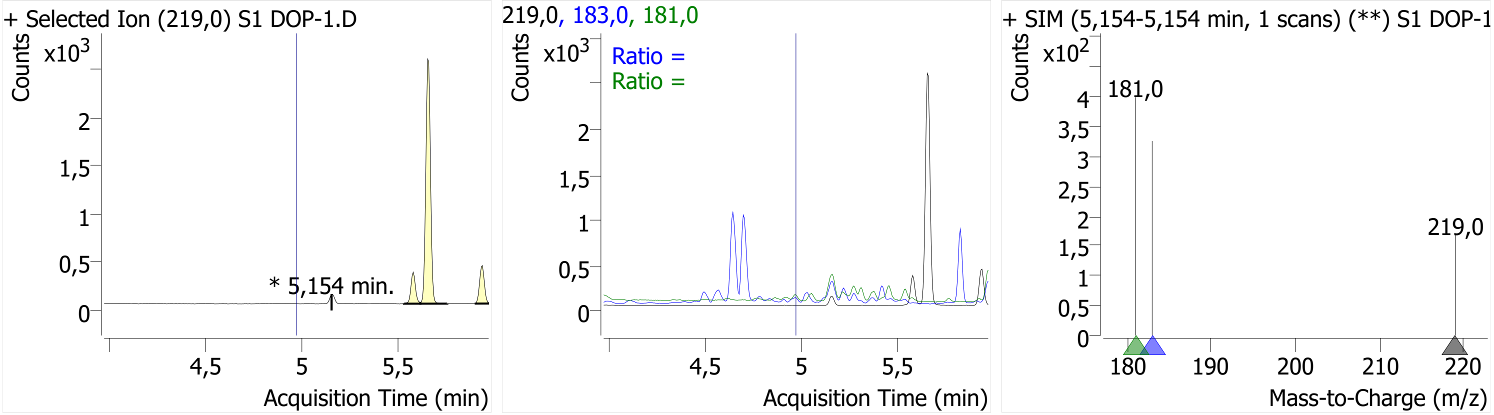

Hexachlorobenzene

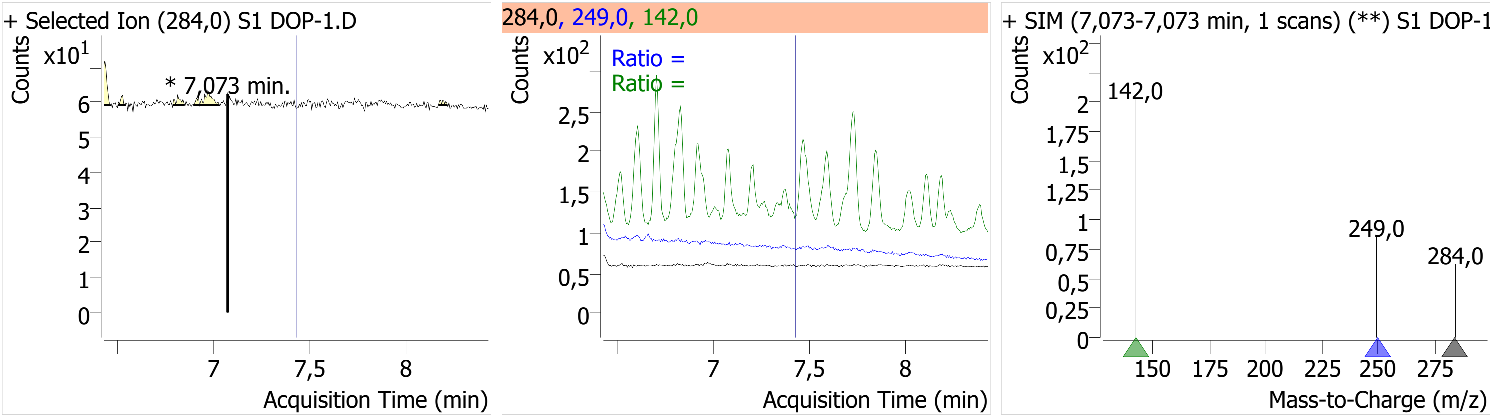

Aldrine

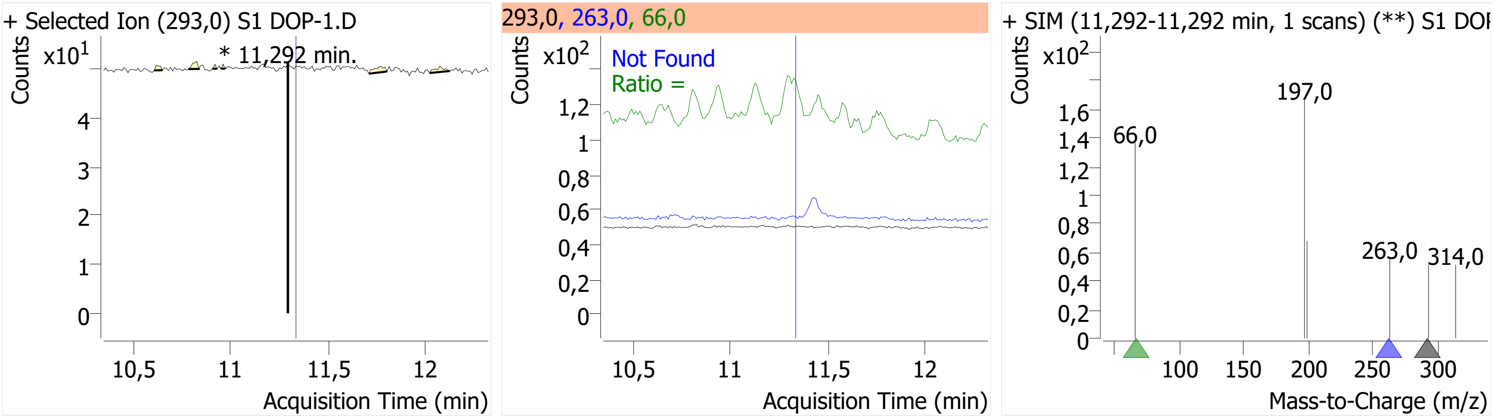

Chlorpyrifos

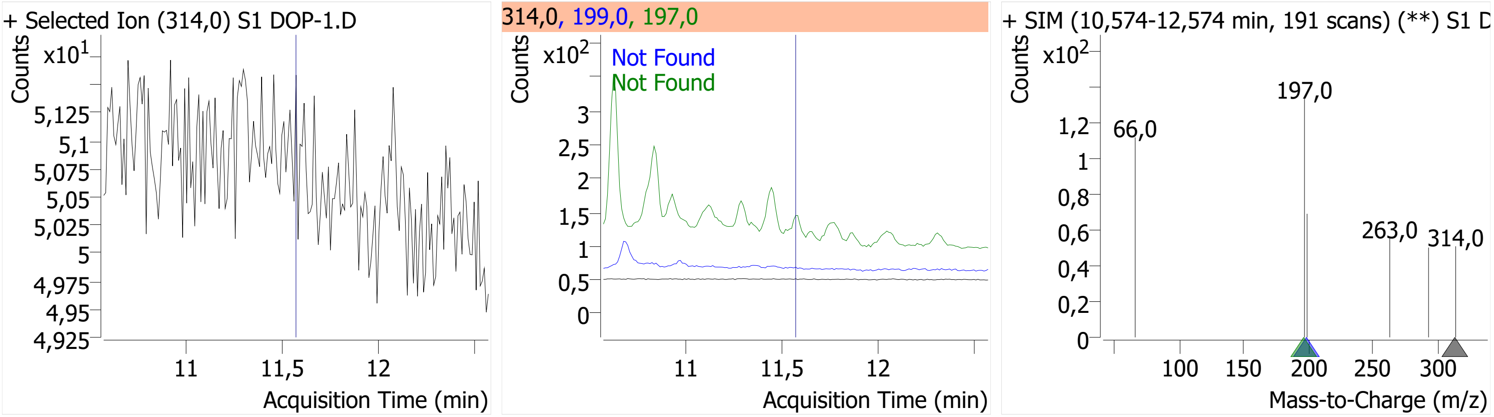

Endosulfan II

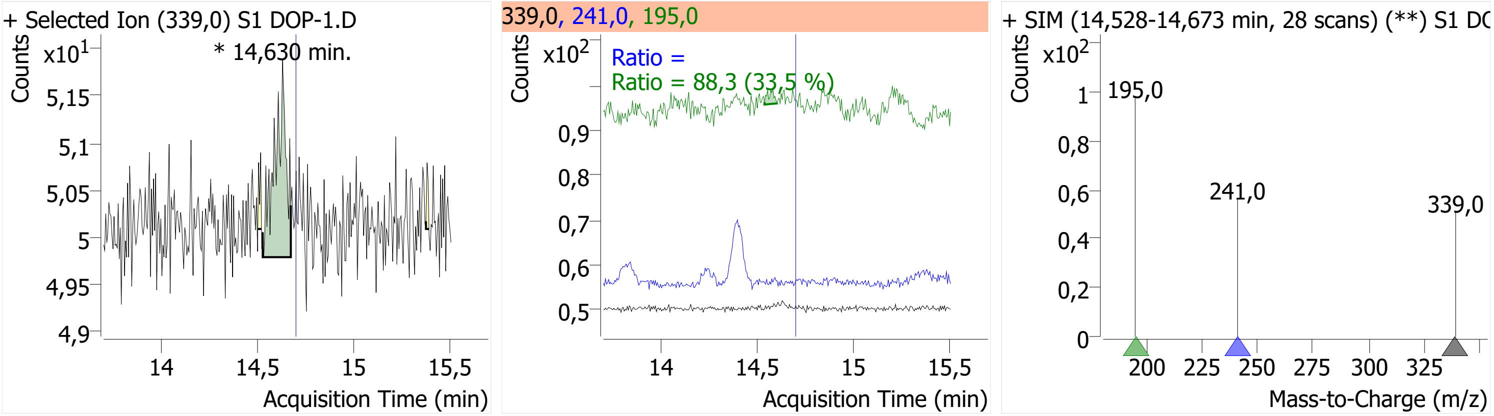

4,4-DDE

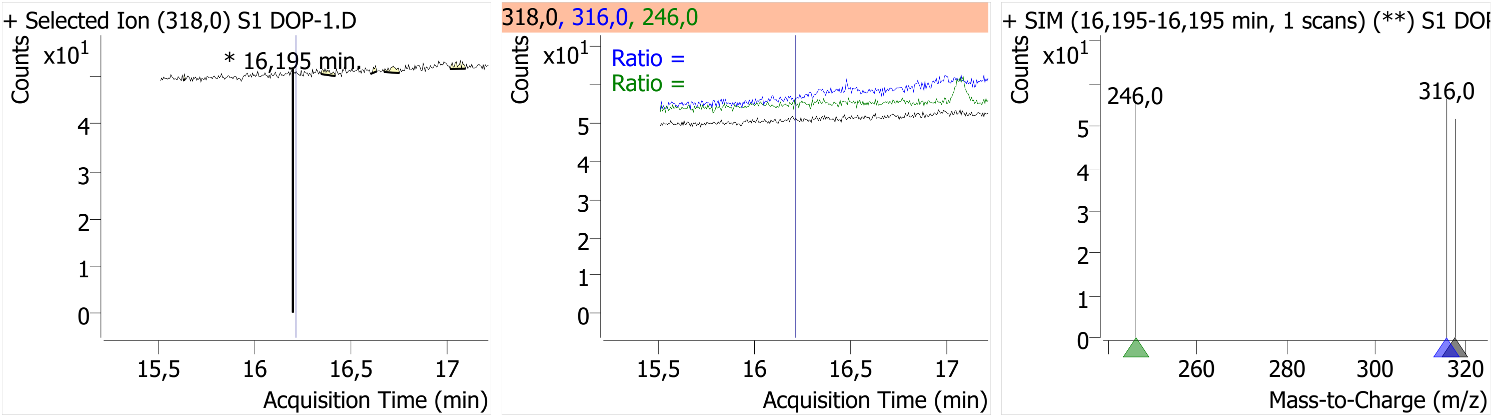

4,4-DDD

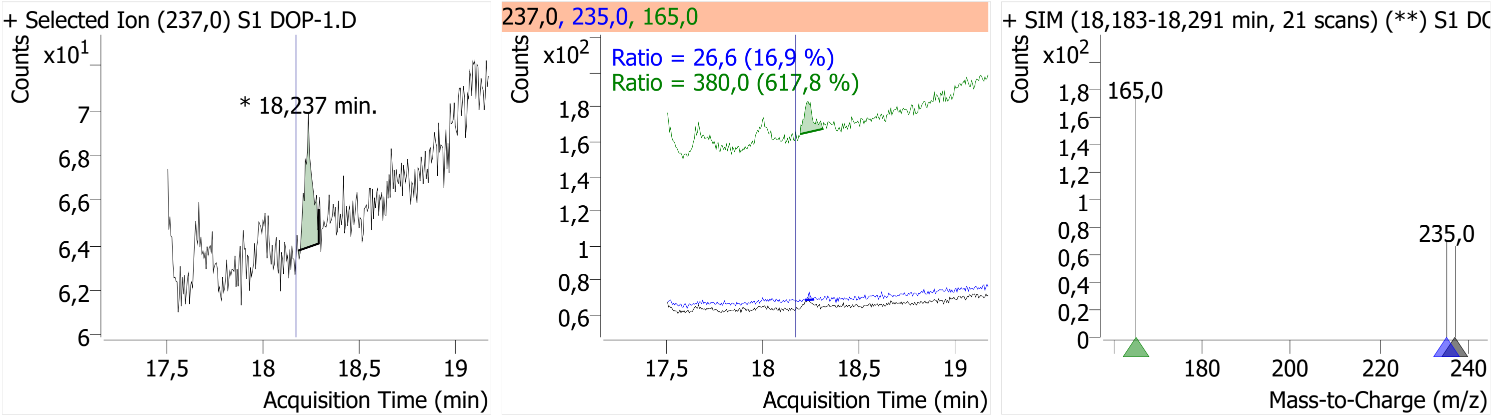

4,4-DDT

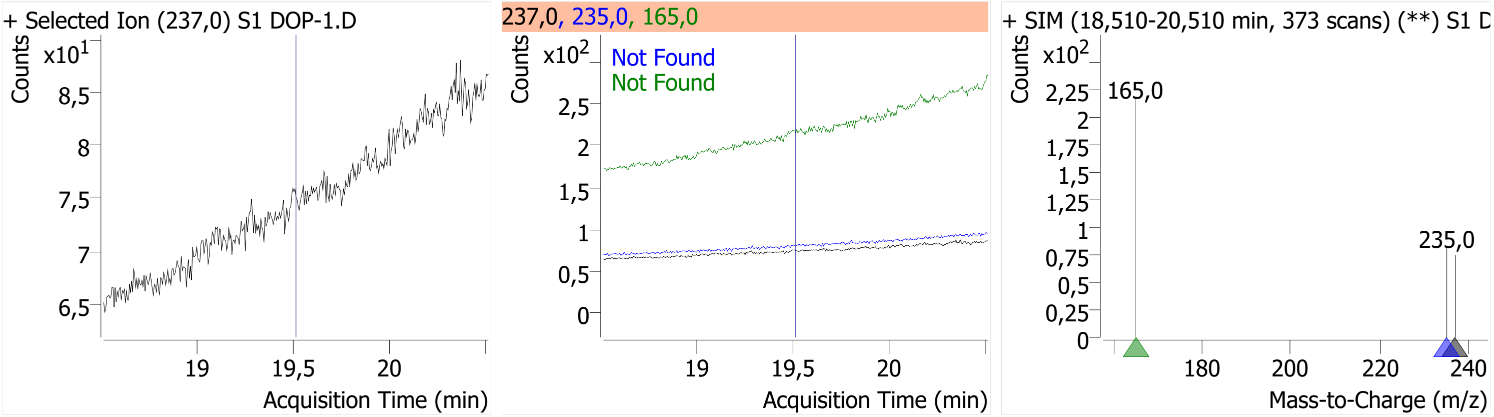

Quantitative Analysis Complete Report

**Batch Path**	C:\Users\USER\OneDrive\Desktop\JU_Pesticide\MA\MA\QuantResults\Mohammed_Pest.batch.bin		
**Analysis Time**	13 Dec 2024 11:34	**Analyst Name**	DESKTOP-MRRPPC4\USER
**Report Time**	13 Dec 2024 11:37:37	**Reporter Name**	DESKTOP-MRRPPC4\USER
**Last Calib Update**	13 Dec 2024 11:34	**Batch State**	Processed
**Quant Batch Version**	10.0	**Quant Report Version**	10.0

**Acq. Time**	13 Nov 2024 05:29	**Data File**	S1 GGC.D
**Sample Type**	Sample	**Sample Name**	Pest
**Dilution**	1	**Acq. Method**	pesticide std 12.11.2024

Sample Chromatogram

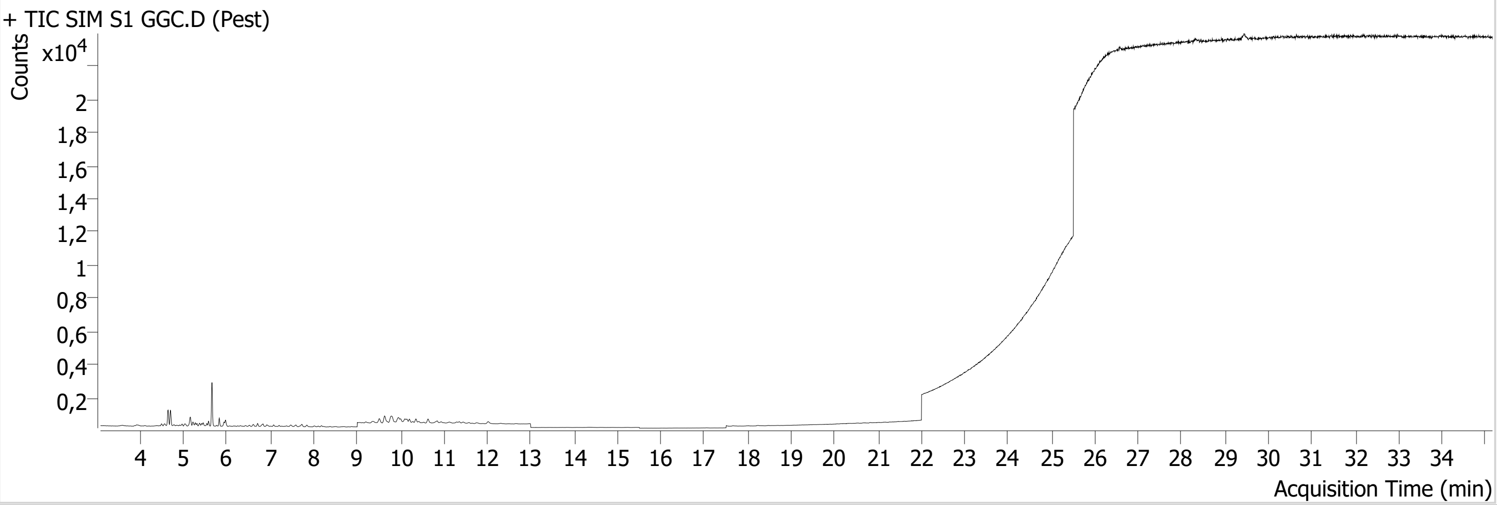

Compound	Transition	RT	Resp.	Final Conc	Units
Lindane	219,0	5,159	0	ND	ng/ml
Hexachlorobenzene	284,0	7,563	0	ND	ng/ml
Aldrine	293,0	11,125	0	ND	ng/ml
Chlorpyrifos	314,0	11,281	0	ND	ng/ml
Endosulfan II	339,0	14,619	15	8,7871	ng/ml
4,4-DDE	318,0			ND	ng/ml
4,4-DDD	237,0	18,258	23	5,5163	ng/ml
4,4-DDT	237,0			ND	ng/ml

Lindane

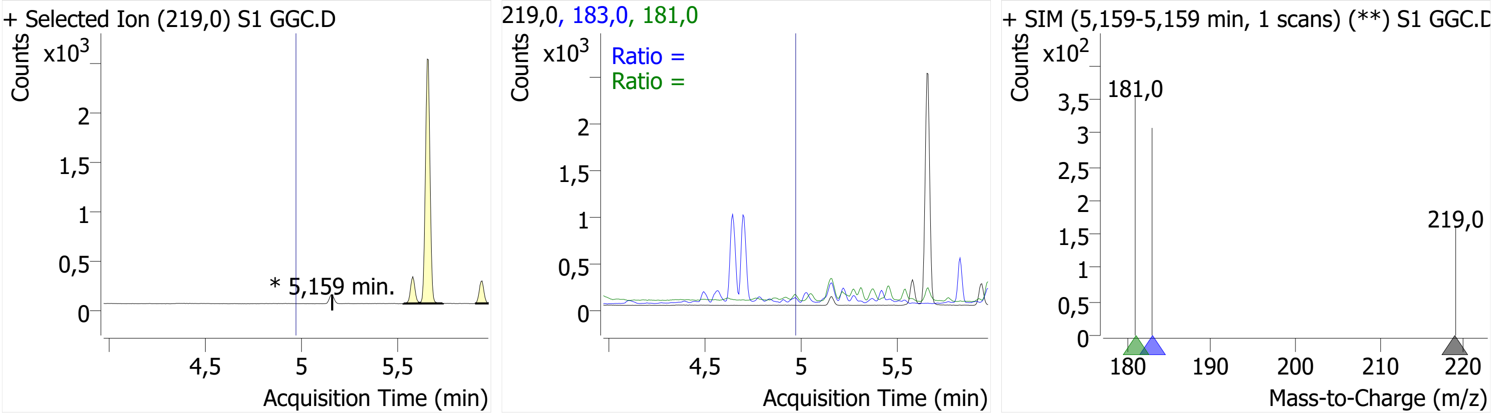

Hexachlorobenzene

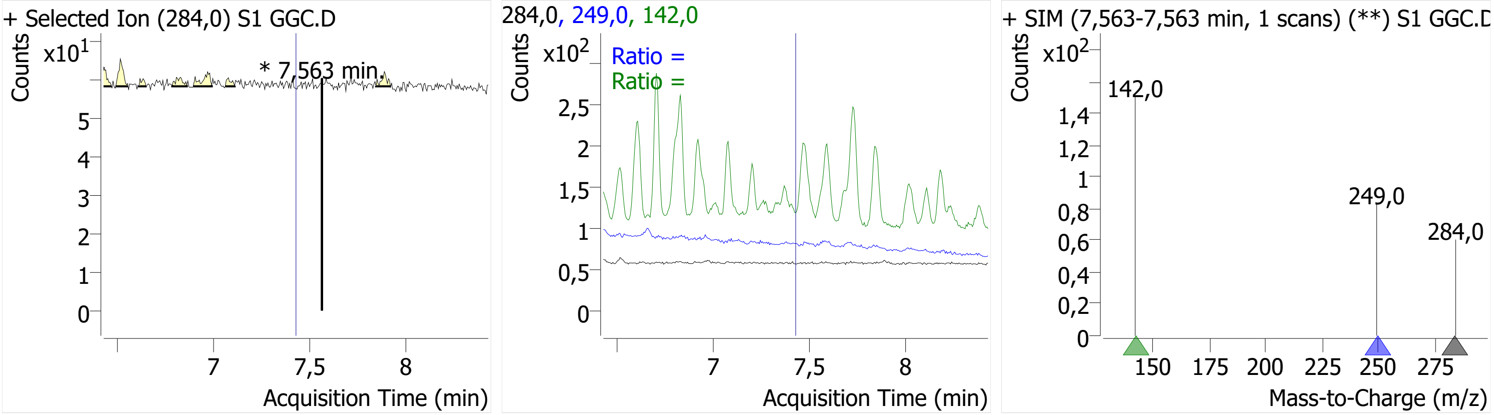

Aldrine

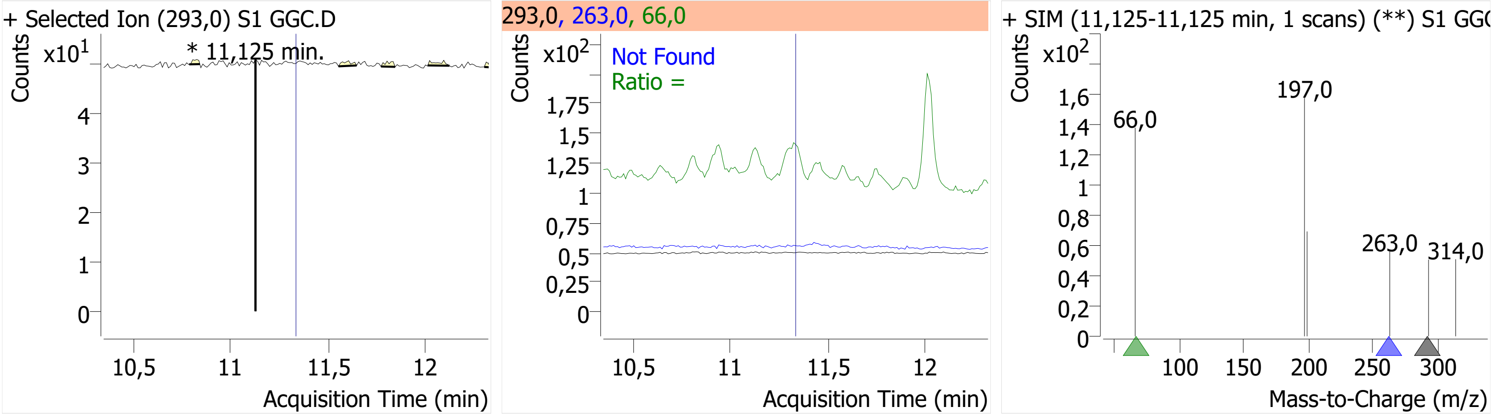

Chlorpyrifos

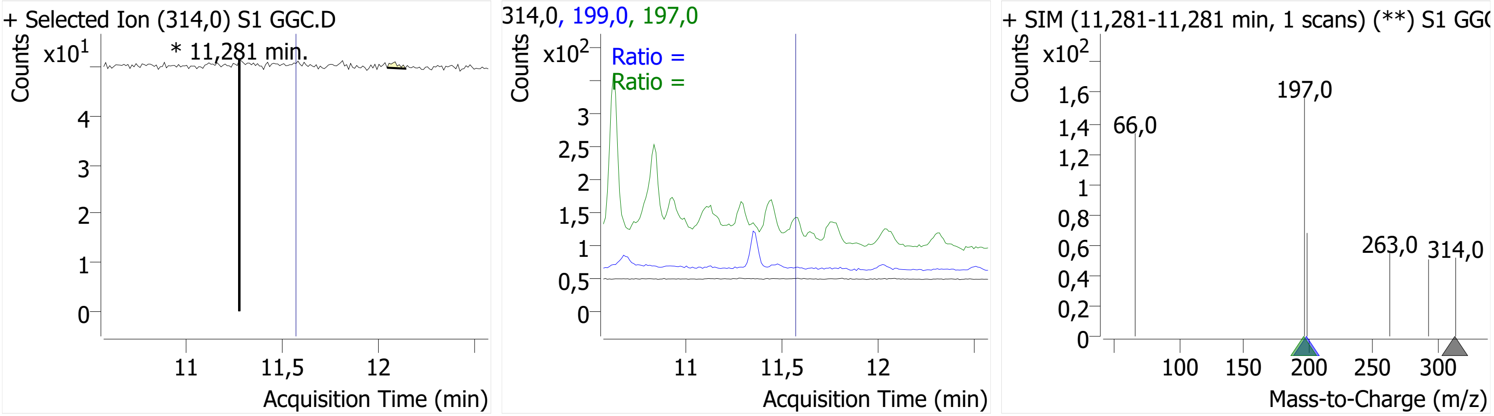

Endosulfan II

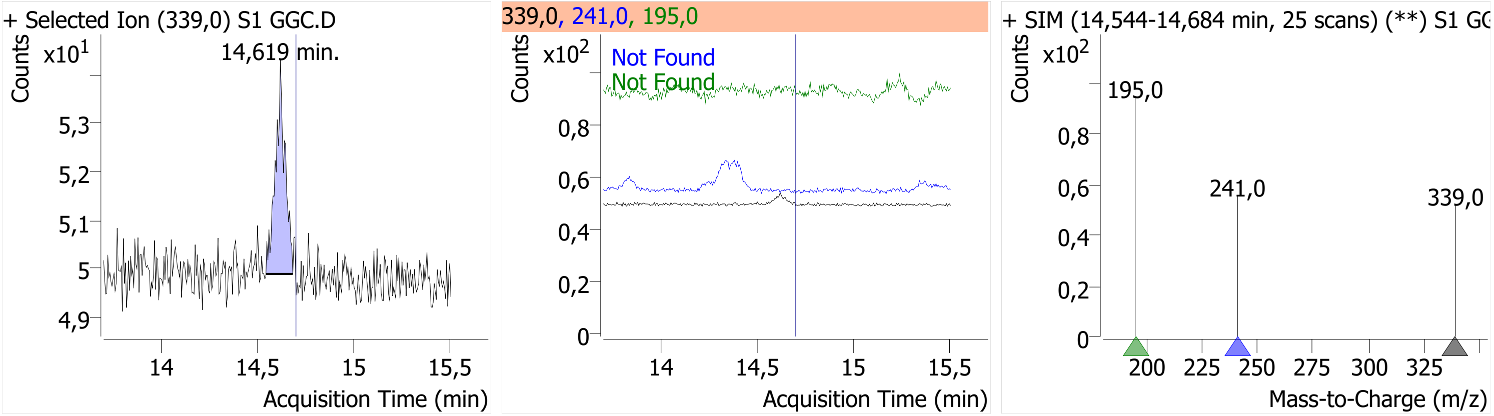

4,4-DDE

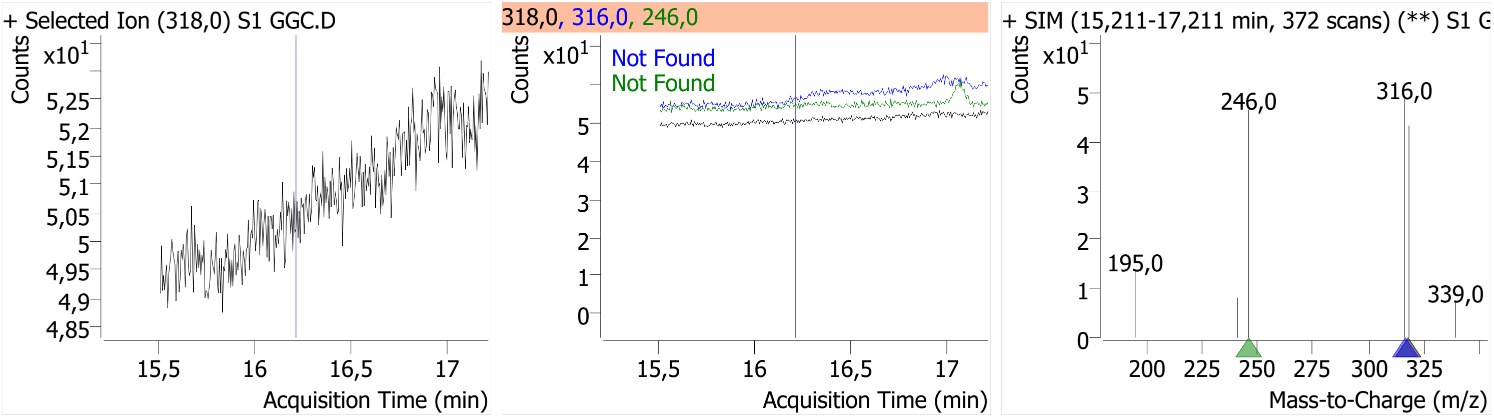

4,4-DDD

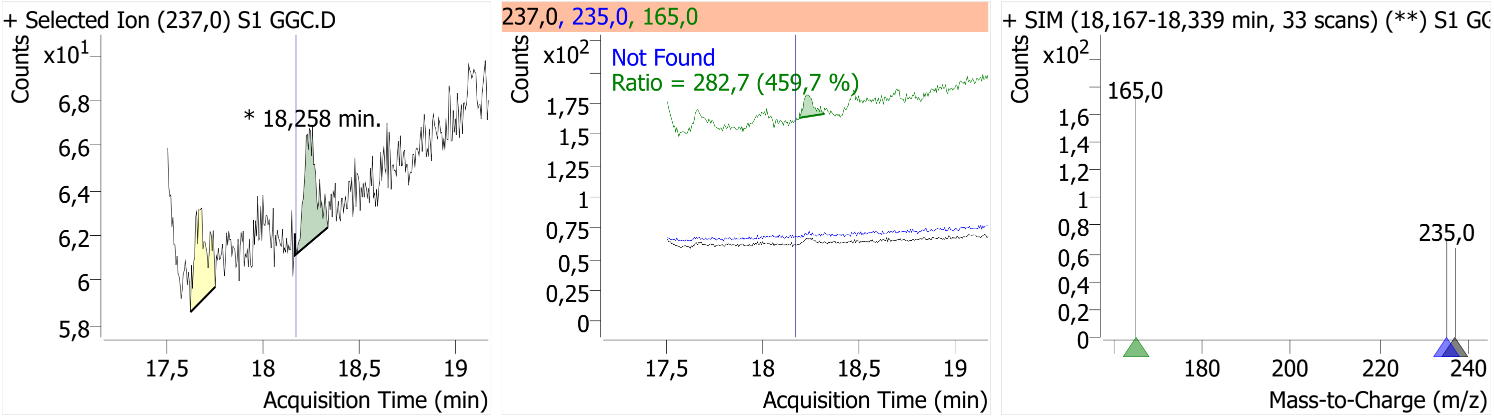

4,4-DDT

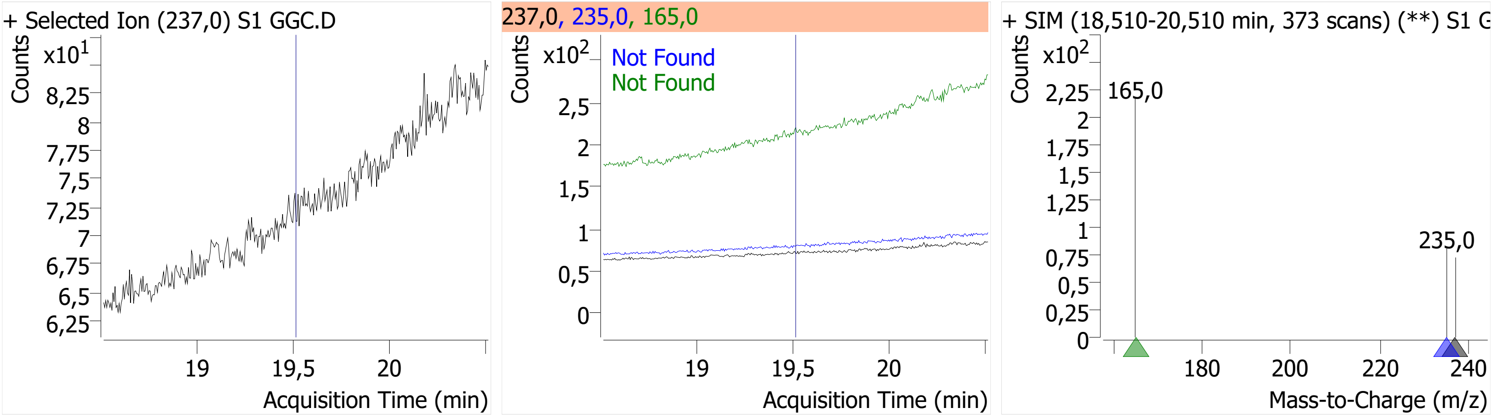

Quantitative Analysis Complete Report

**Batch Path**	C:\Users\USER\OneDrive\Desktop\JU_Pesticide\MA\MA\QuantResults\Mohammed_Pest.batch.bin		
**Analysis Time**	13 Dec 2024 11:34	**Analyst Name**	DESKTOP-MRRPPC4\USER
**Report Time**	13 Dec 2024 11:37:38	**Reporter Name**	DESKTOP-MRRPPC4\USER
**Last Calib Update**	13 Dec 2024 11:34	**Batch State**	Processed
**Quant Batch Version**	10.0	**Quant Report Version**	10.0

**Acq. Time**	13 Nov 2024 06:10	**Data File**	S1 GGP.D
**Sample Type**	Sample	**Sample Name**	Pest
**Dilution**	1	**Acq. Method**	pesticide std 12.11.2024

Sample Chromatogram

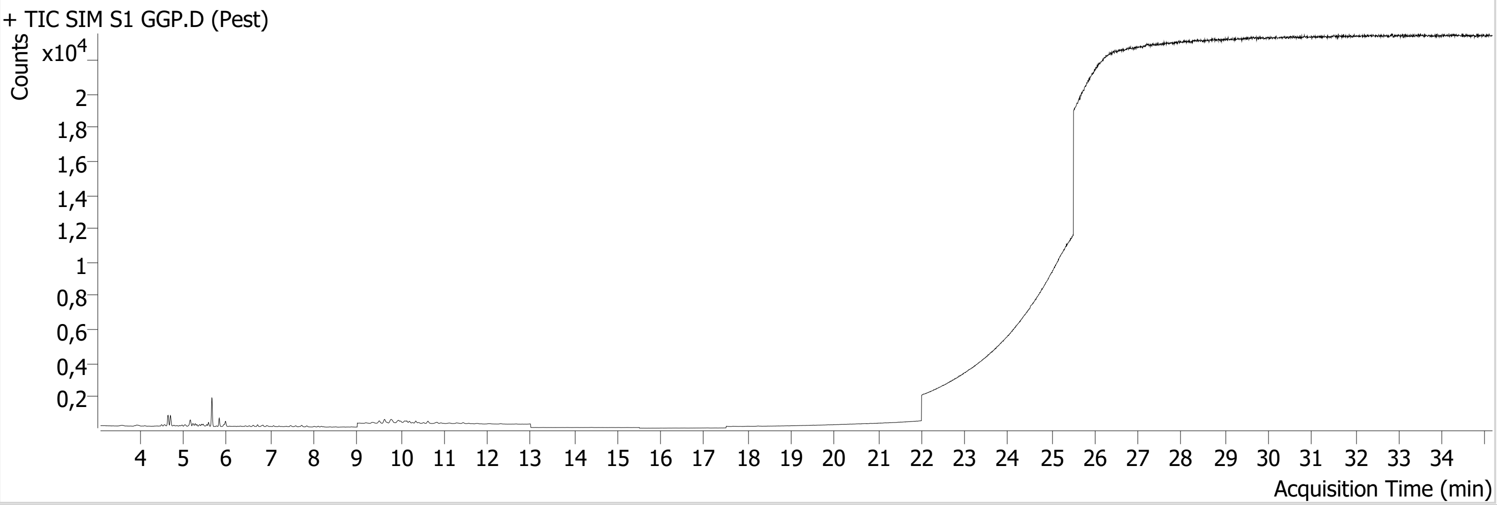

Compound	Transition	RT	Resp.	Final Conc	Units
Lindane	219,0	5,154	0	ND	ng/ml
Hexachlorobenzene	284,0	7,466	0	ND	ng/ml
Aldrine	293,0	11,459	0	ND	ng/ml
Chlorpyrifos	314,0	12,065	0	ND	ng/ml
Endosulfan II	339,0	14,635	0	ND	ng/ml
4,4-DDE	318,0			ND	ng/ml
4,4-DDD	237,0	18,226	15	4,0388	ng/ml
4,4-DDT	237,0			ND	ng/ml

Lindane

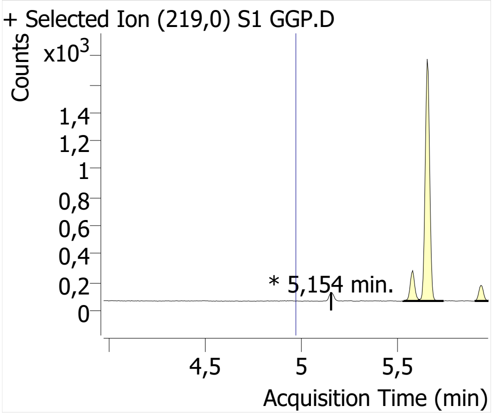

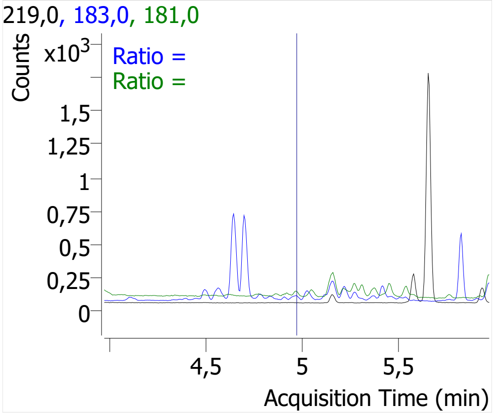

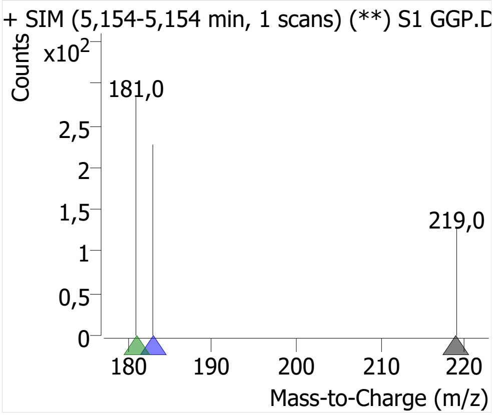

Agilent Trusted Answers

Hexachlorobenzene

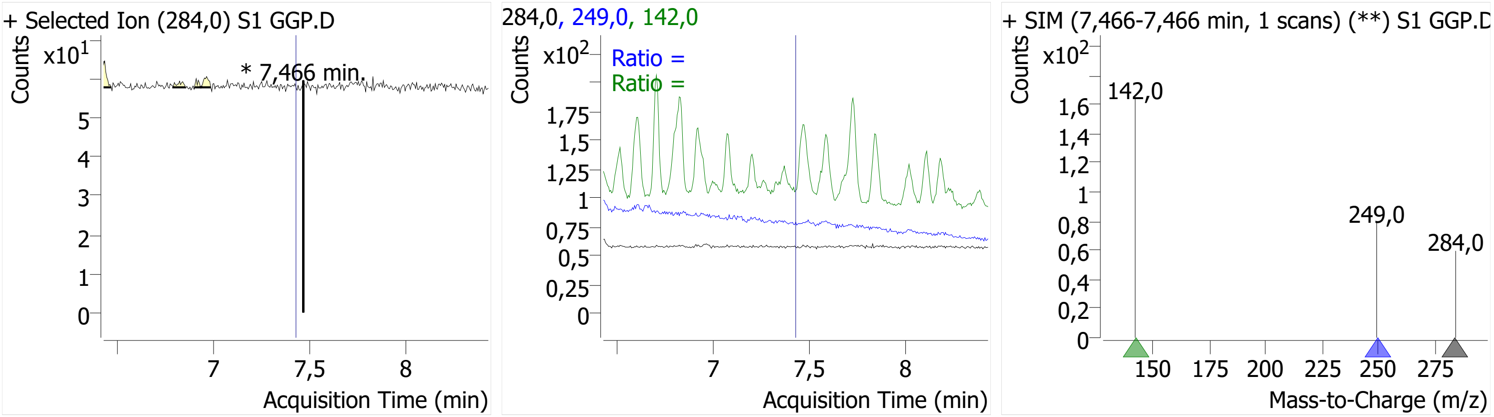

Aldrine

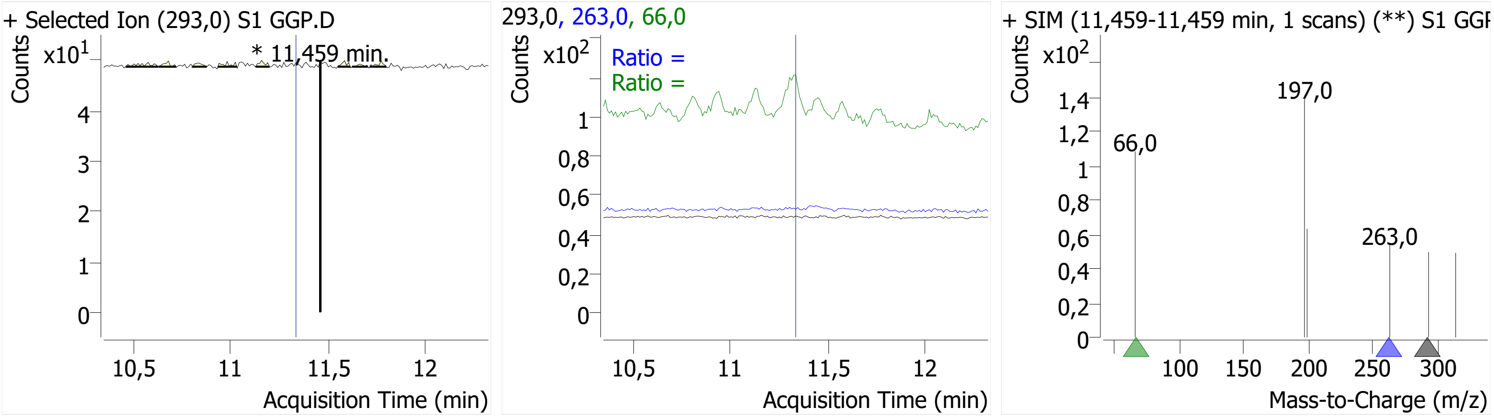

Chlorpyrifos

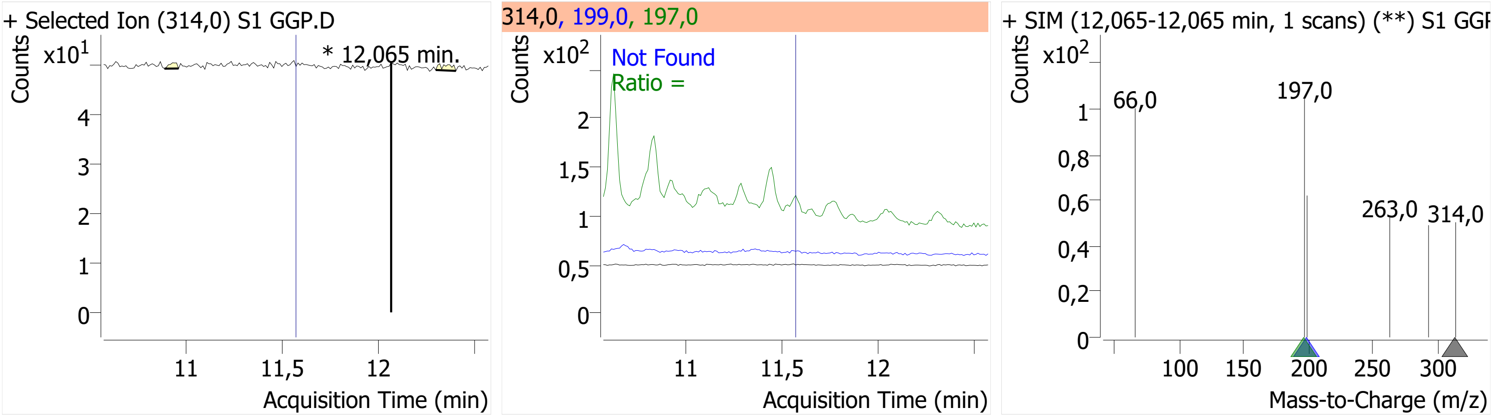

Endosulfan II

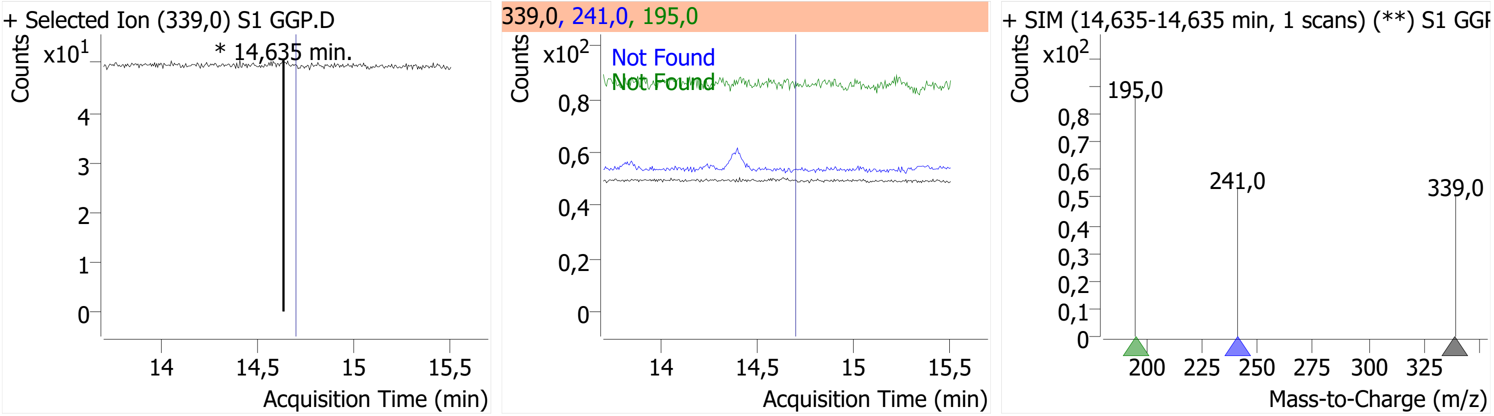

4,4-DDE

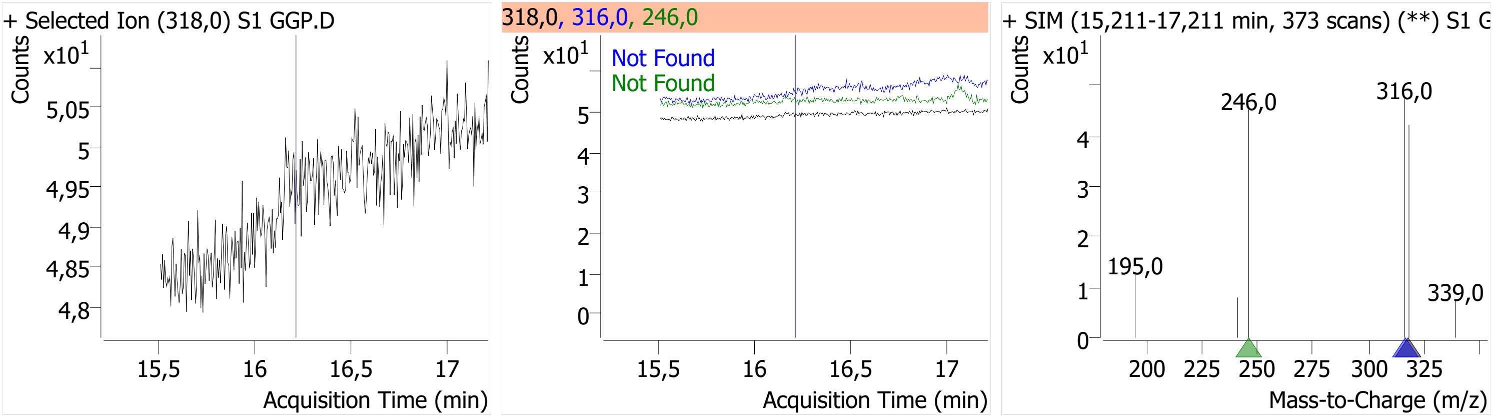

4,4-DDD

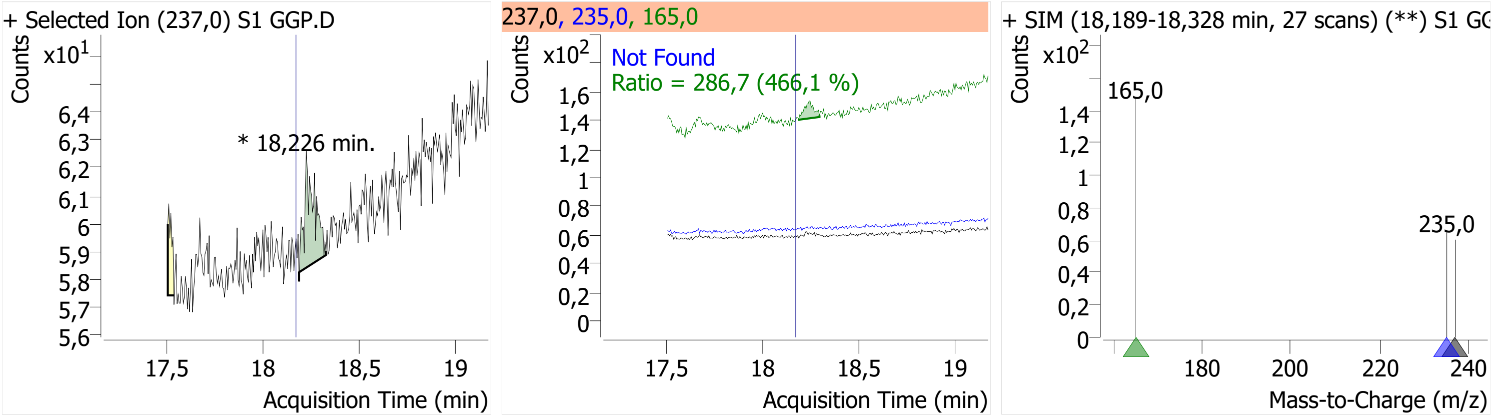

4,4-DDT

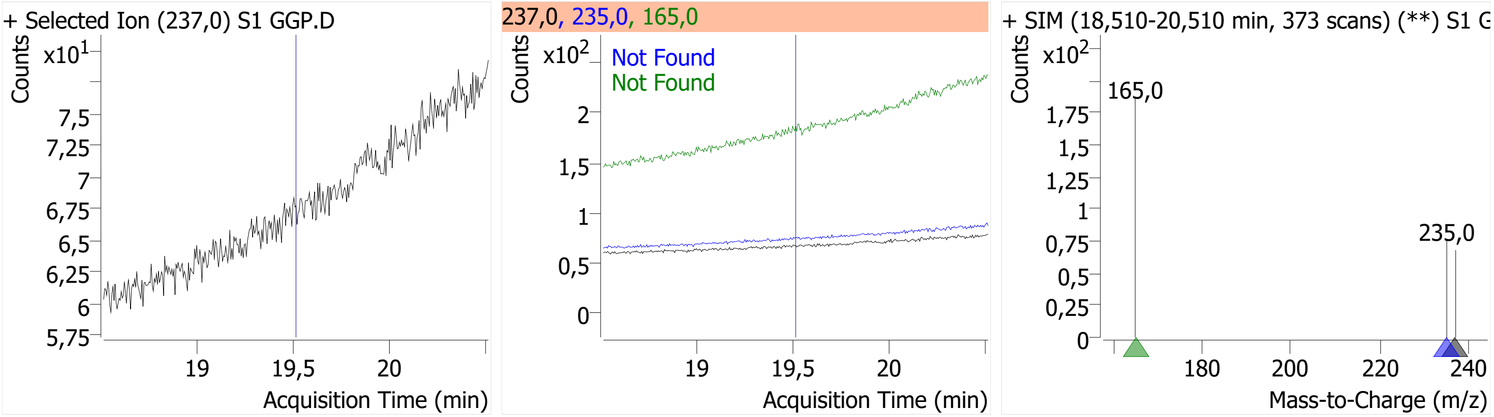

**Batch Path**	C:\Users\USER\OneDrive\Desktop\JU_Pesticide\MA\MA\QuantResults\Mohammed_Pest.batch.bin		
**Analysis Time**	13 Dec 2024 11:34	**Analyst Name**	DESKTOP-MRRPPC4\USER
**Report Time**	13 Dec 2024 11:37:39	**Reporter Name**	DESKTOP-MRRPPC4\USER
**Last Calib Update**	13 Dec 2024 11:34	**Batch State**	Processed
**Quant Batch Version**	10.0	**Quant Report Version**	10.0

**Acq. Time**	13 Nov 2024 06:51	**Data File**	S1 DWT.D
**Sample Type**	Sample	**Sample Name**	Pest
**Dilution**	1	**Acq. Method**	pesticide std 12.11.2024

Sample Chromatogram

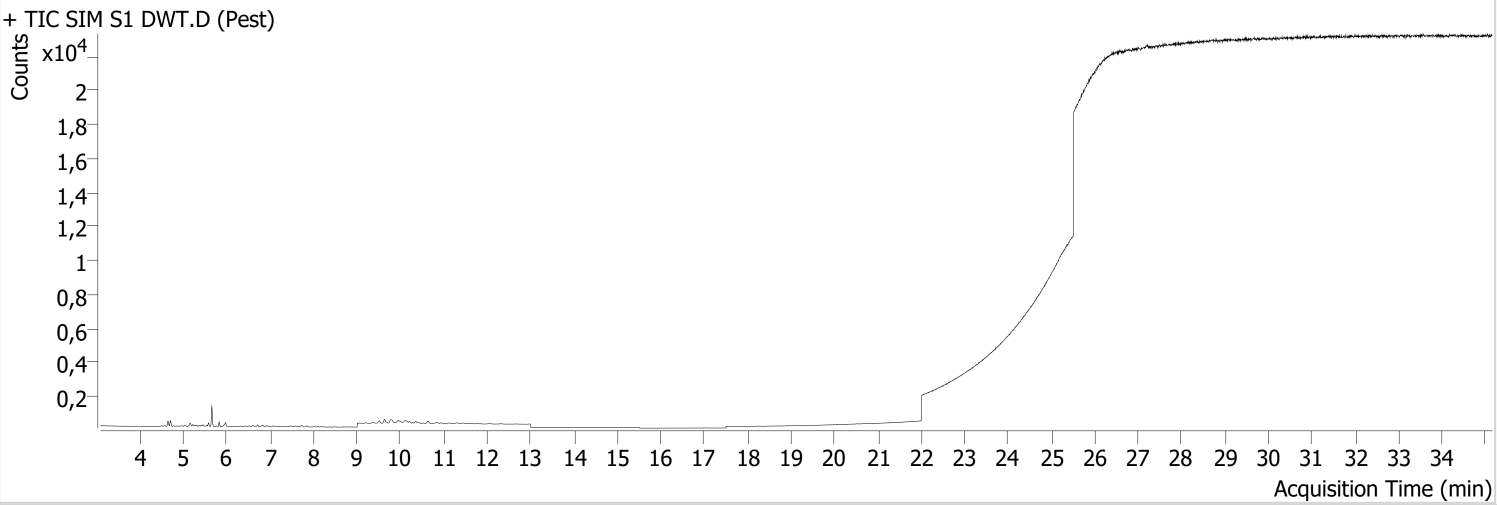

Compound	Transition	RT	Resp.	Final Conc	Units
Lindane	219,0	5,154	0	ND	ng/ml
Hexachlorobenzene	284,0	6,971	0	ND	ng/ml
Aldrine	293,0	11,177	0	ND	ng/ml
Chlorpyrifos	314,0			ND	ng/ml
Endosulfan II	339,0	14,635	18	11,6826	ng/ml
4,4-DDE	318,0	16,324	0	ND	ng/ml
4,4-DDD	237,0	18,232	12	3,4701	ng/ml
4,4-DDT	237,0			ND	ng/ml

Lindane

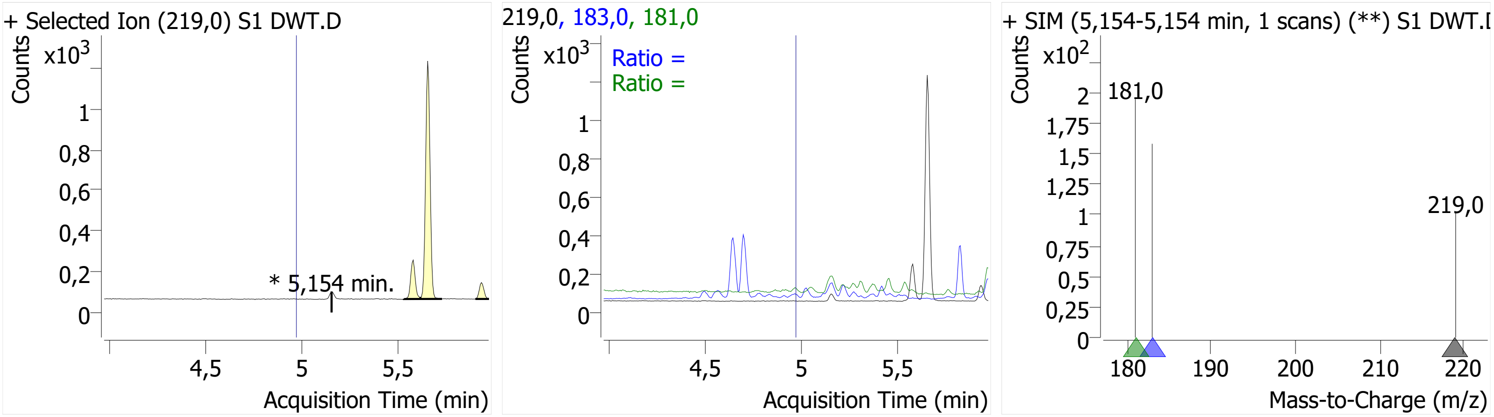

Hexachlorobenzene

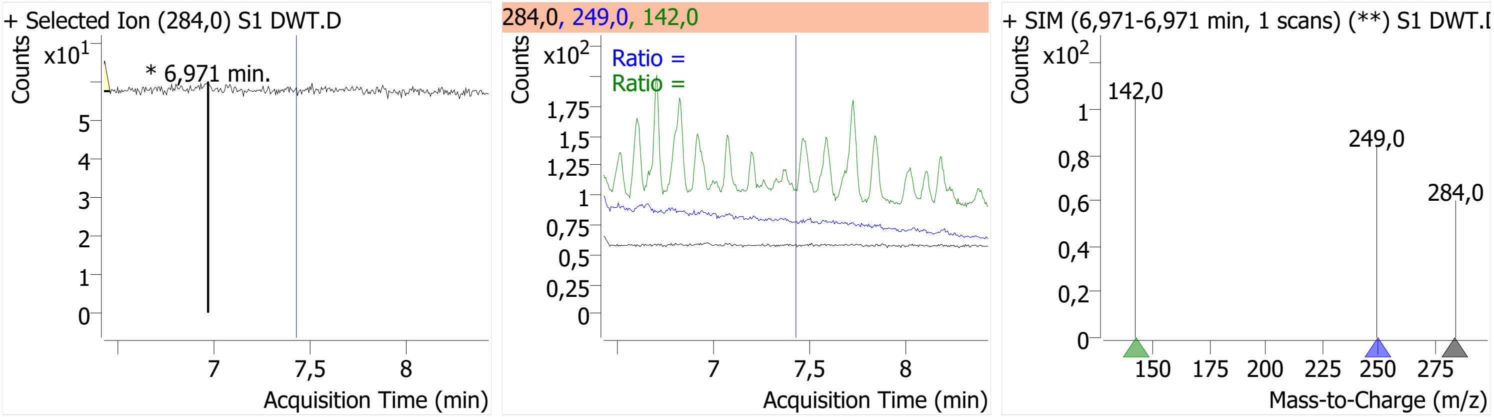

Aldrine

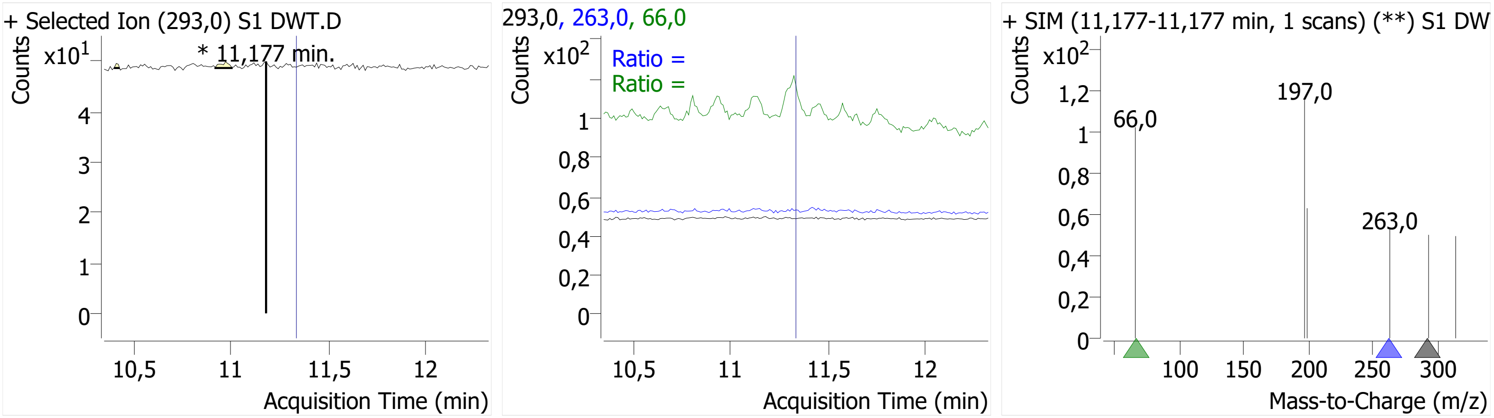

Chlorpyrifos

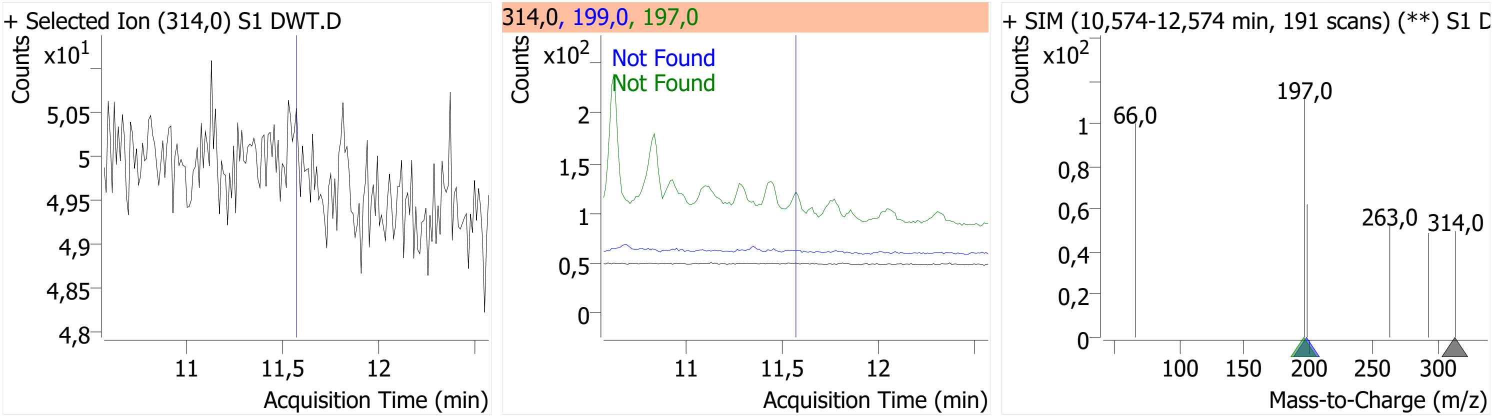

Endosulfan II

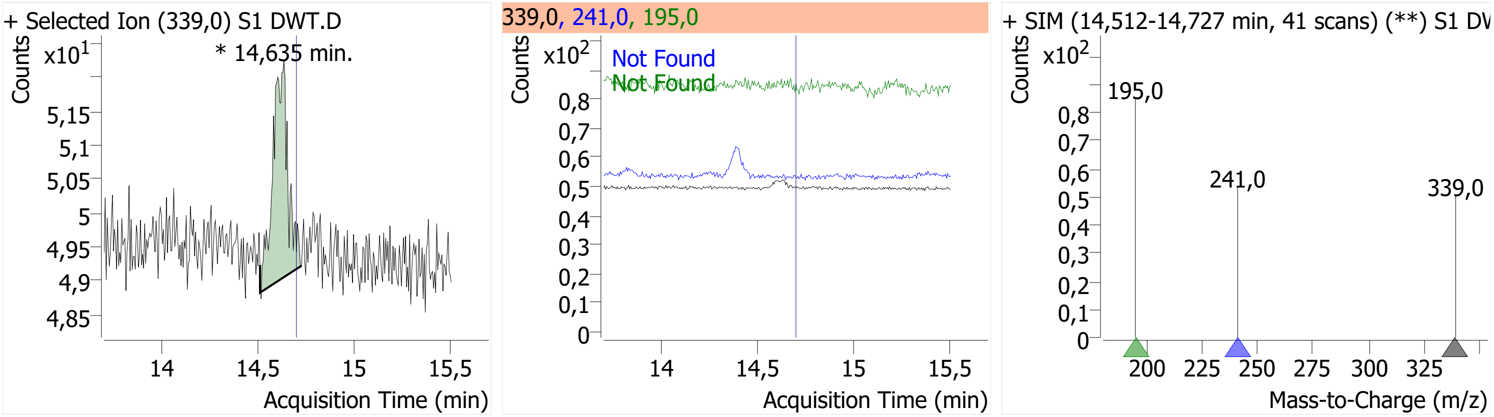

4,4-DDE

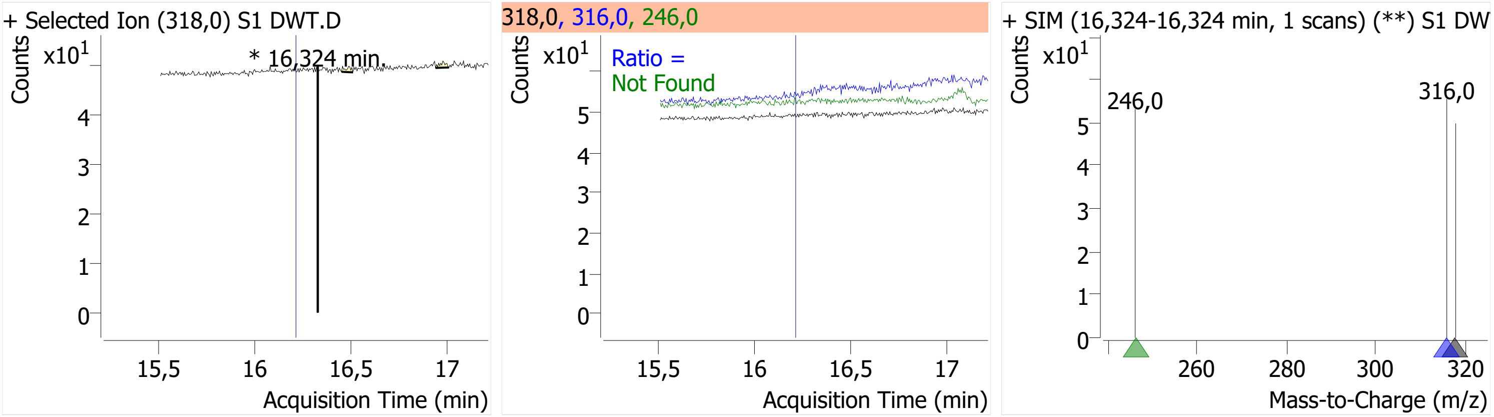

4,4-DDD

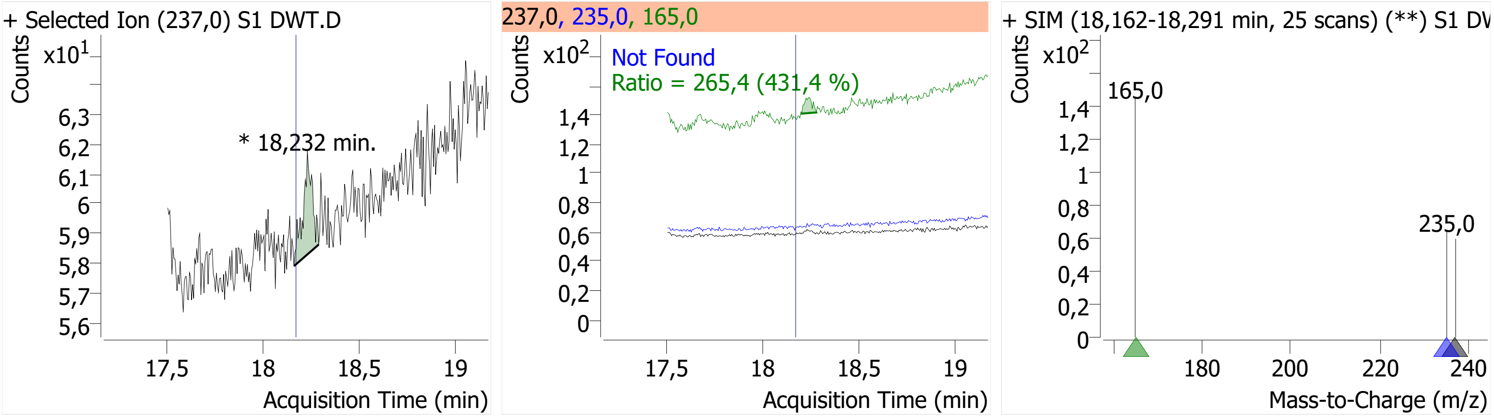

4,4-DDT

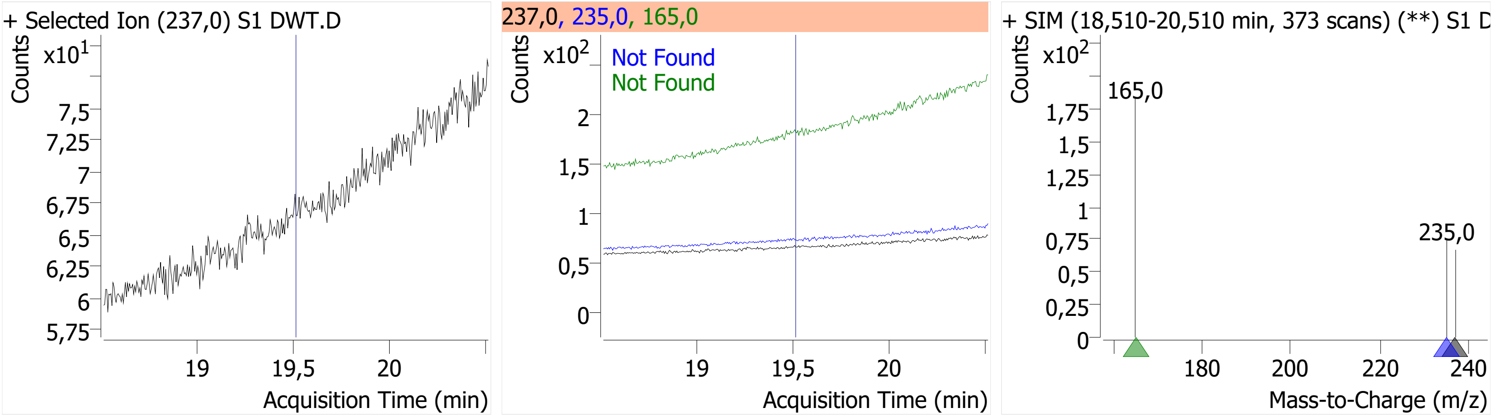

Quantitative Analysis Complete Report

**Batch Path**	C:\Users\USER\OneDrive\Desktop\JU_Pesticide\MA\MA\QuantResults\Mohammed_Pest.batch.bin		
**Analysis Time**	13 Dec 2024 11:34	**Analyst Name**	DESKTOP-MRRPPC4\USER
**Report Time**	13 Dec 2024 11:37:40	**Reporter Name**	DESKTOP-MRRPPC4\USER
**Last Calib Update**	13 Dec 2024 11:34	**Batch State**	Processed
**Quant Batch Version**	10.0	**Quant Report Version**	10.0

**Acq. Time**	13 Nov 2024 07:31	**Data File**	S1 DOT.D
**Sample Type**	Sample	**Sample Name**	Pest
**Dilution**	1	**Acq. Method**	pesticide std 12.11.2024

Sample Chromatogram

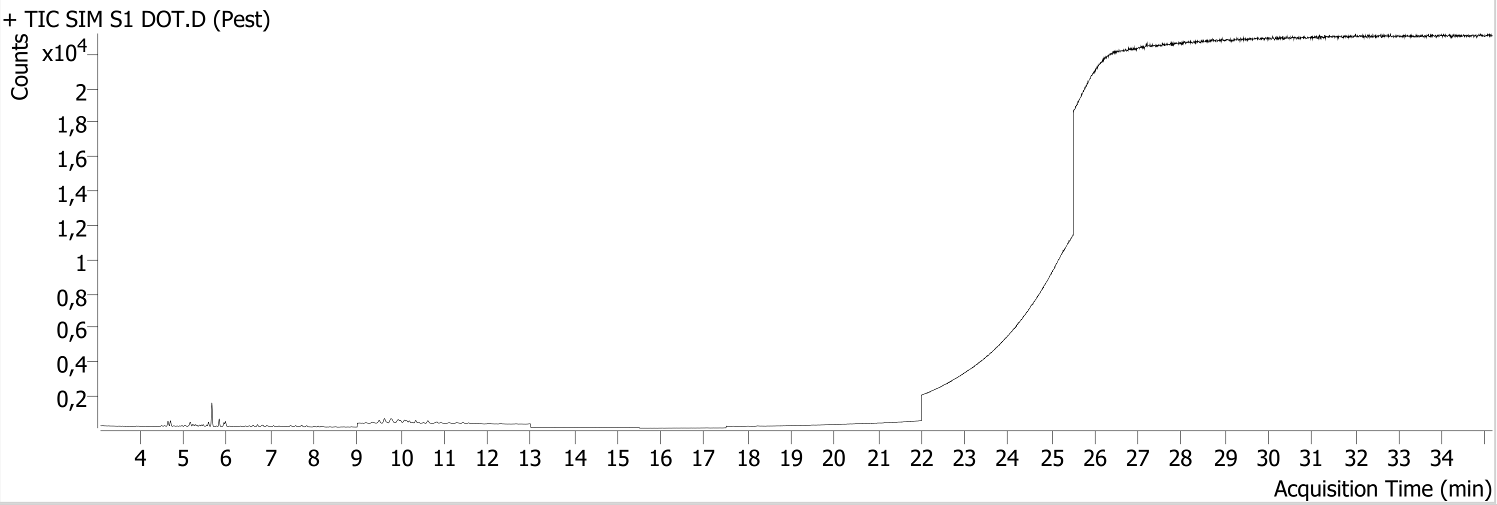

Compound	Transition	RT	Resp.	Final Conc	Units
Lindane	219,0	5,153	0	ND	ng/ml
Hexachlorobenzene	284,0	7,756	0	ND	ng/ml
Aldrine	293,0	11,125	0	ND	ng/ml
Chlorpyrifos	314,0	11,553	10	6,9763	ng/ml
Endosulfan II	339,0	14,619	15	9,1162	ng/ml
4,4-DDE	318,0	17,205	0	ND	ng/ml
4,4-DDD	237,0	18,221	15	4,1917	ng/ml
4,4-DDT	237,0			ND	ng/ml

Lindane

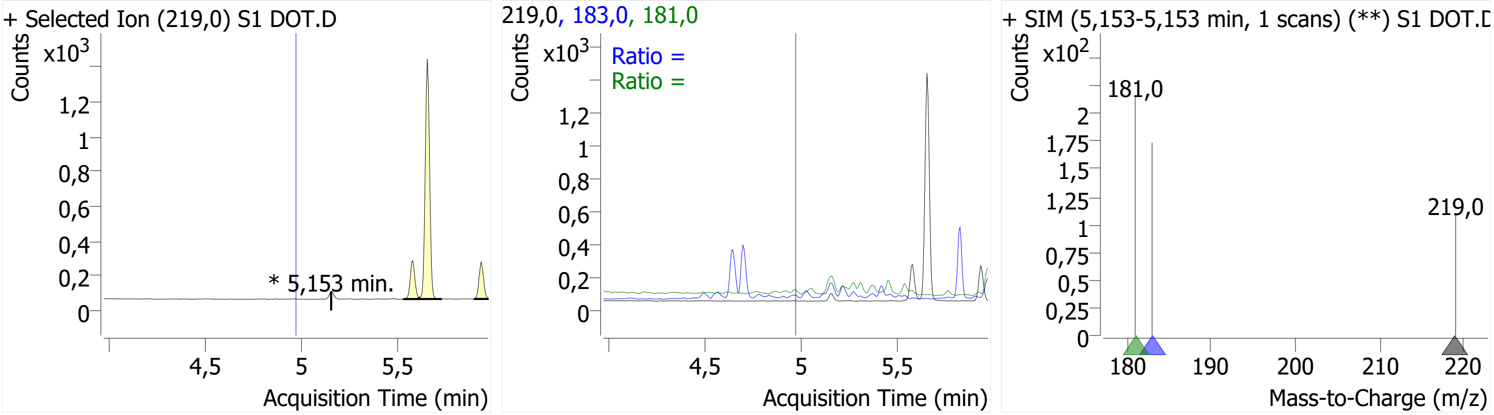

Hexachlorobenzene

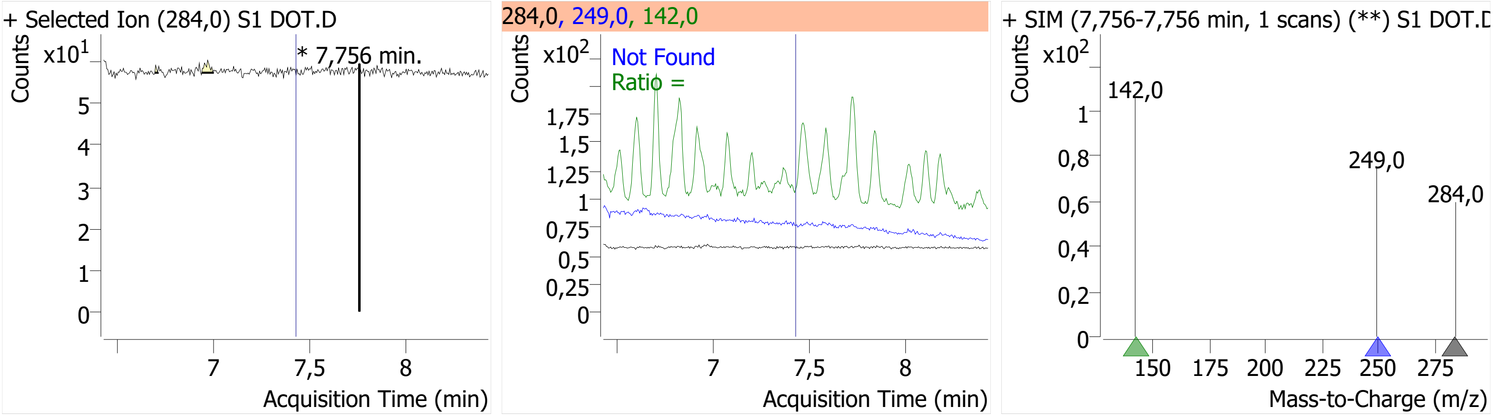

Aldrine

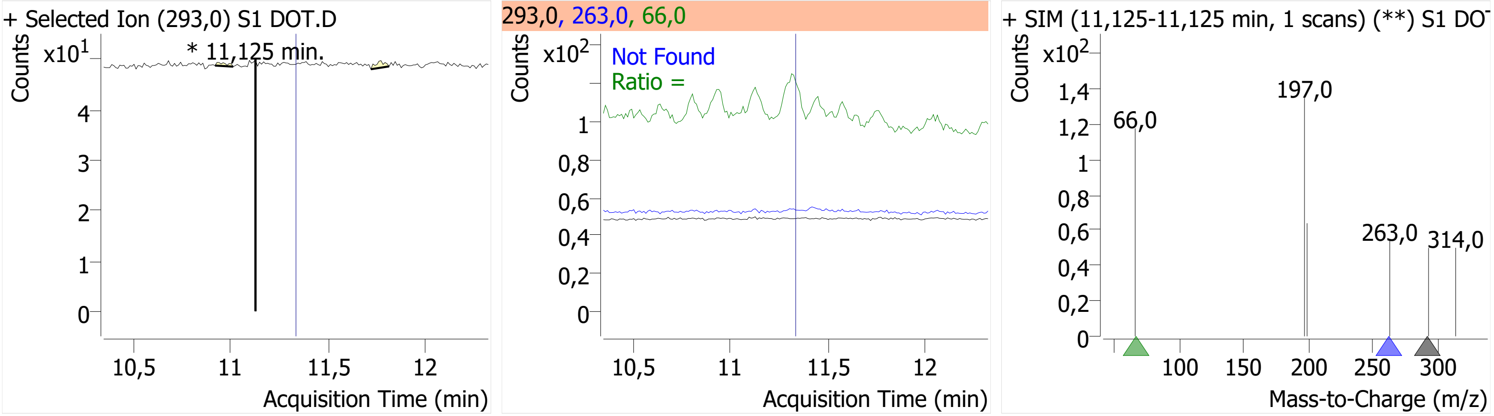

Chlorpyrifos

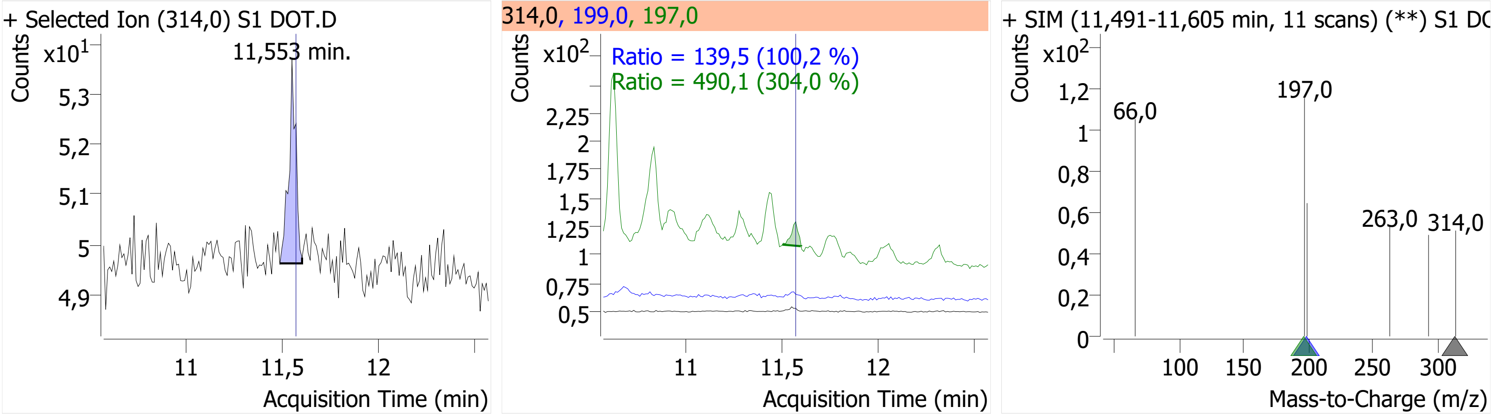

Endosulfan II

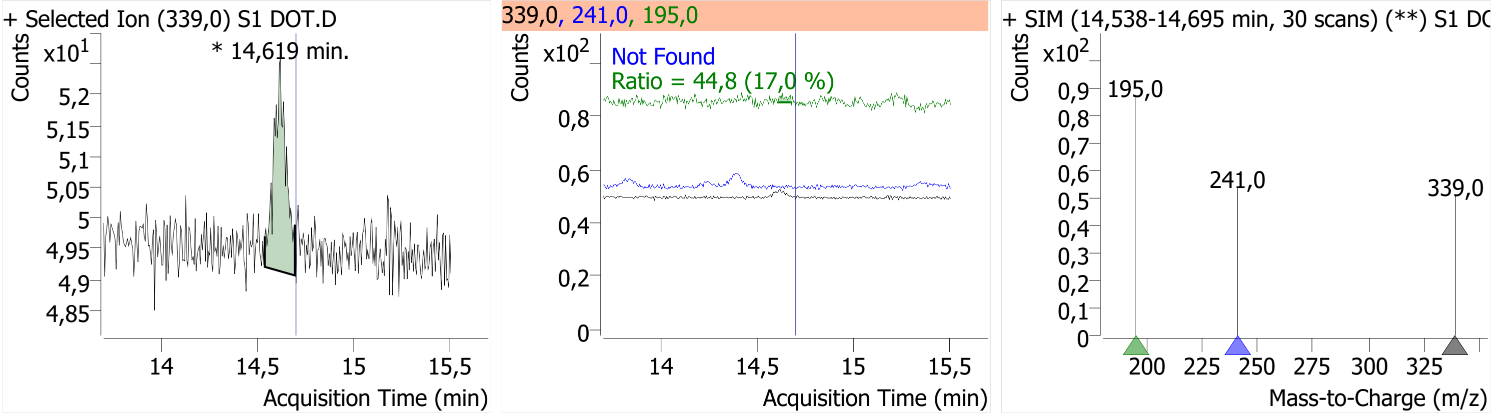

4,4-DDE

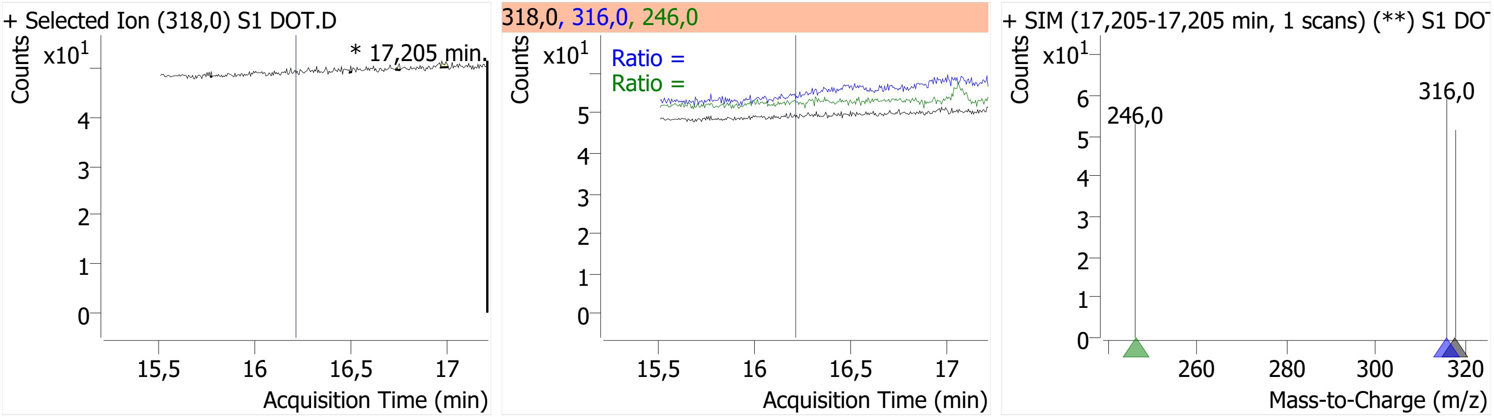

4,4-DDD

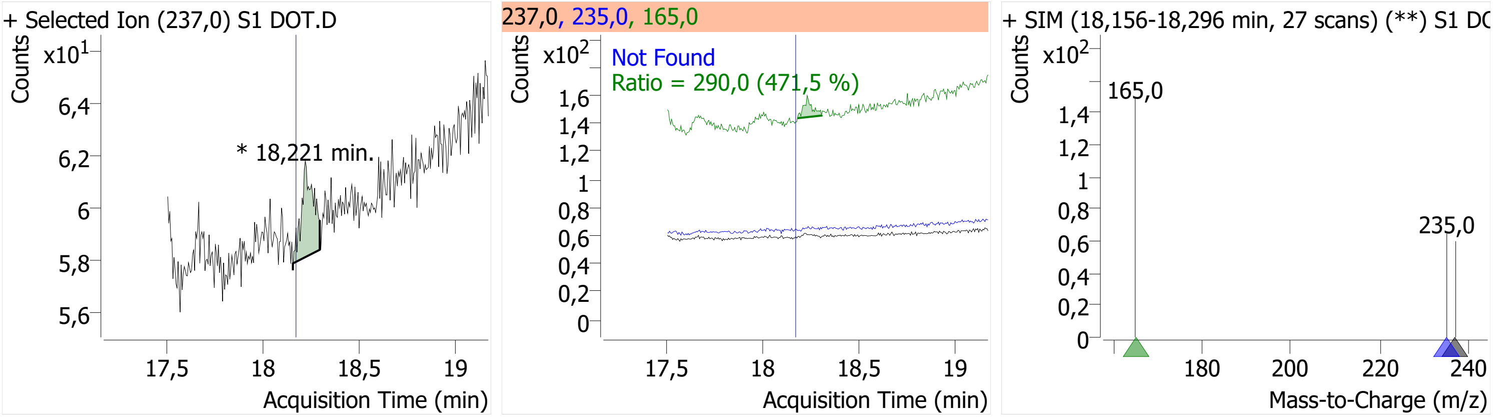

4,4-DDT

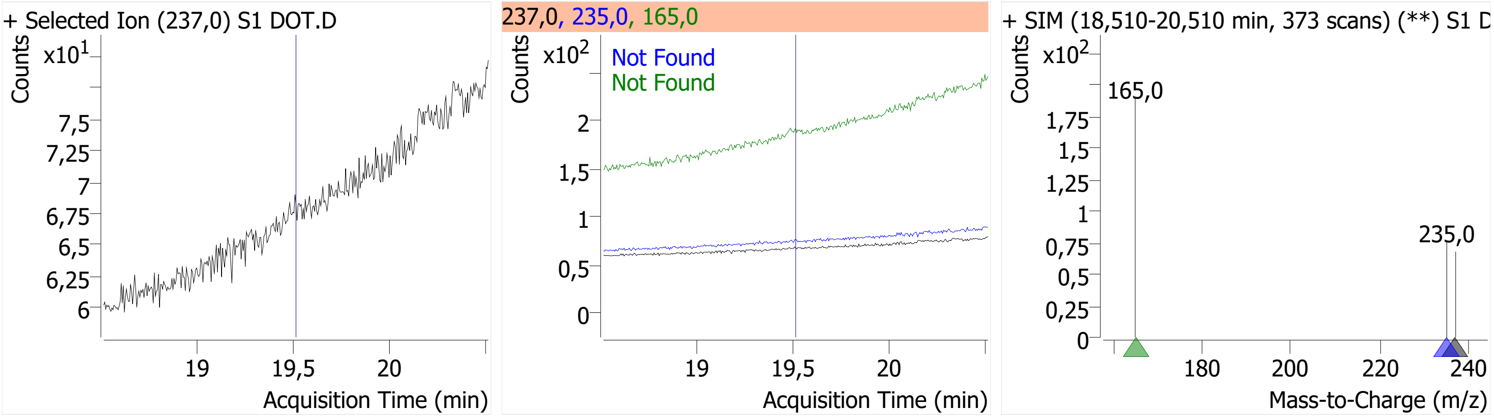

Quantitative Analysis Complete Report

**Batch Path**	C:\Users\USER\OneDrive\Desktop\JU_Pesticide\MA\MA\QuantResults\Mohammed_Pest.batch.bin		
**Analysis Time**	13 Dec 2024 11:34	**Analyst Name**	DESKTOP-MRRPPC4\USER
**Report Time**	13 Dec 2024 11:37:41	**Reporter Name**	DESKTOP-MRRPPC4\USER
**Last Calib Update**	13 Dec 2024 11:34	**Batch State**	Processed
**Quant Batch Version**	10.0	**Quant Report Version**	10.0

**Acq. Time**	13 Nov 2024 08:12	**Data File**	S2 GJC.D
**Sample Type**	Sample	**Sample Name**	Pest
**Dilution**	1	**Acq. Method**	pesticide std 12.11.2024

Sample Chromatogram

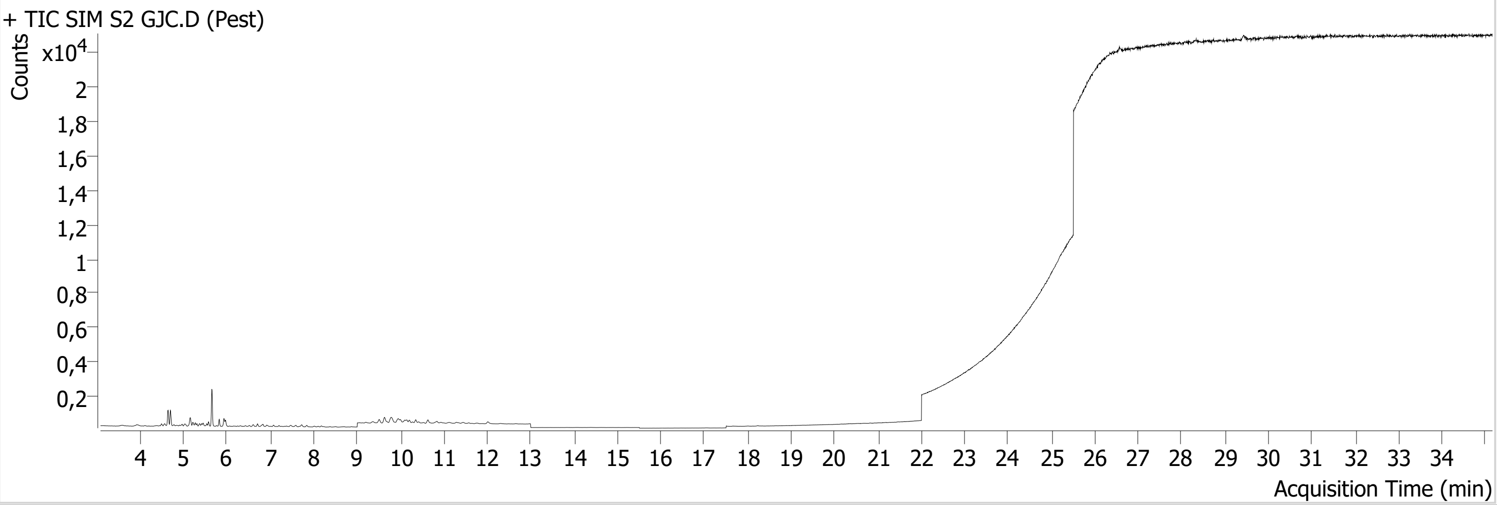

Compound	Transition	RT	Resp.	Final Conc	Units
Lindane	219,0	5,153	0	ND	ng/ml
Hexachlorobenzene	284,0	7,590	0	ND	ng/ml
Aldrine	293,0	11,156	0	ND	ng/ml
Chlorpyrifos	314,0	12,096	0	ND	ng/ml
Endosulfan II	339,0	14,635	12	6,2162	ng/ml
4,4-DDE	318,0	16,963	0	ND	ng/ml
4,4-DDD	237,0	18,231	14	3,8790	ng/ml
4,4-DDT	237,0	19,258	0	ND	ng/ml

Lindane

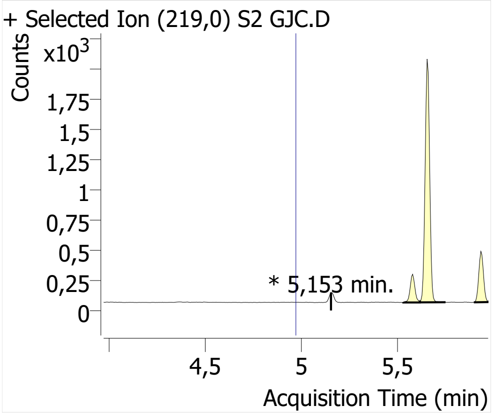

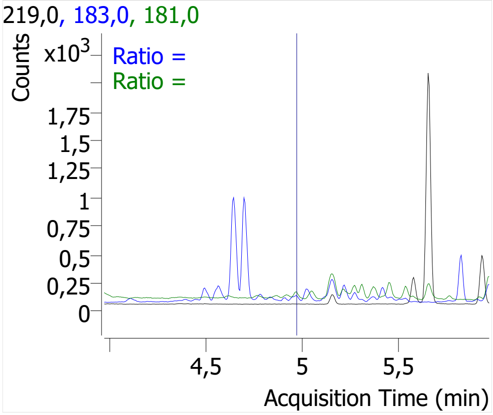

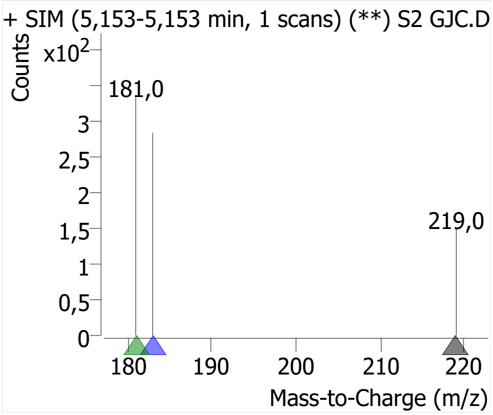

Hexachlorobenzene

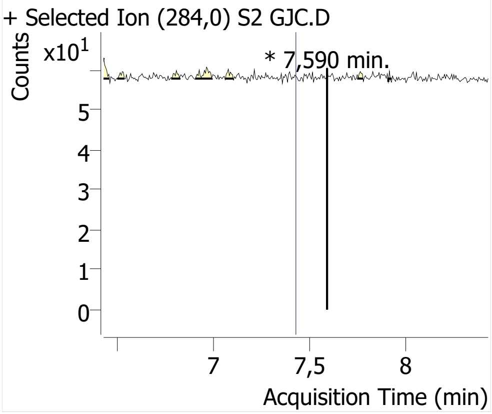

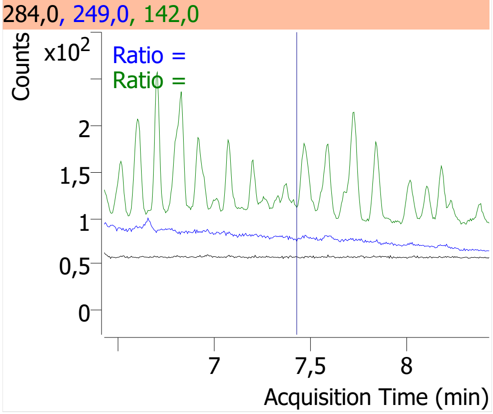

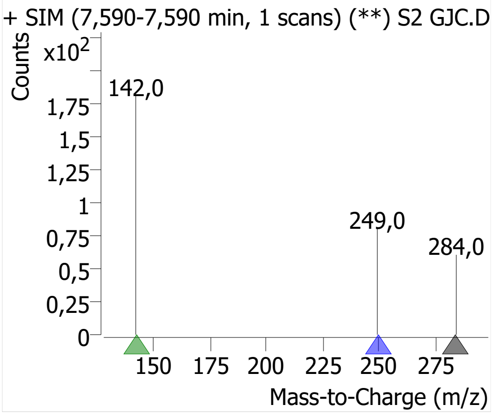

Aldrine

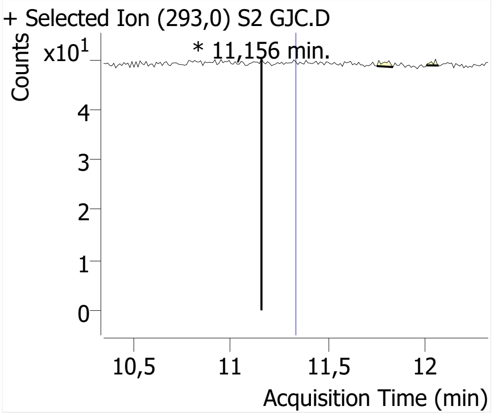

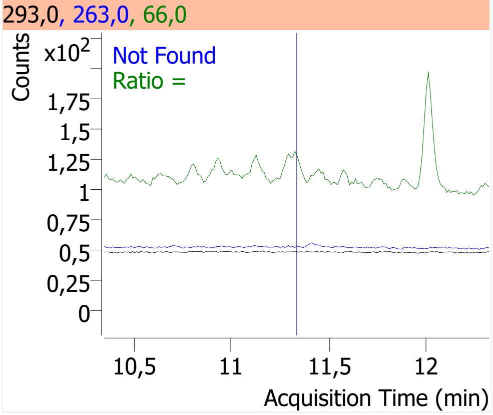

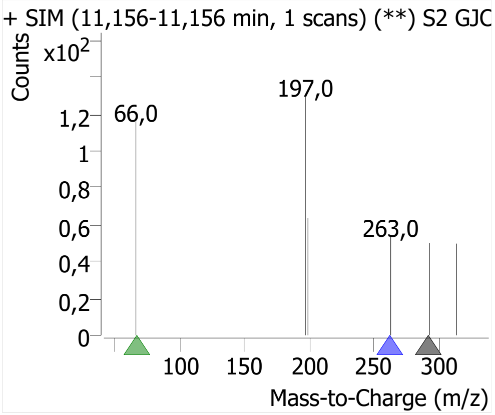

Chlorpyrifos

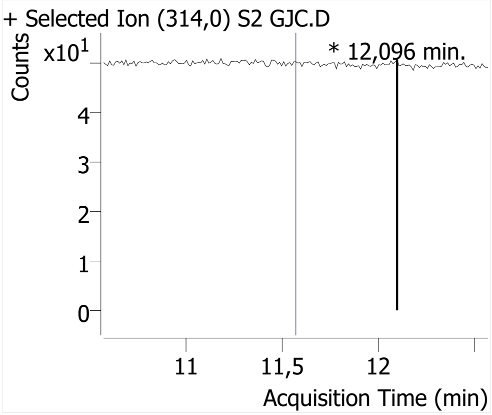

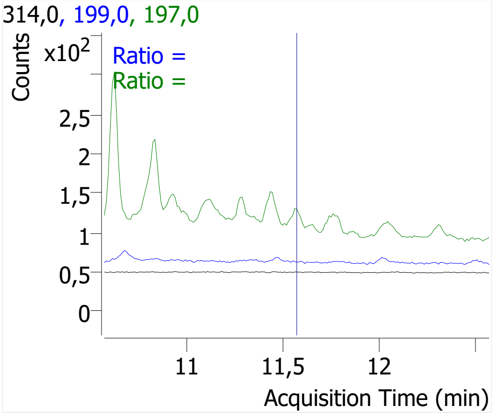

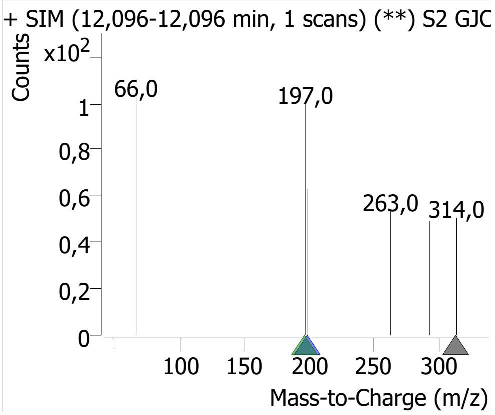

Endosulfan II

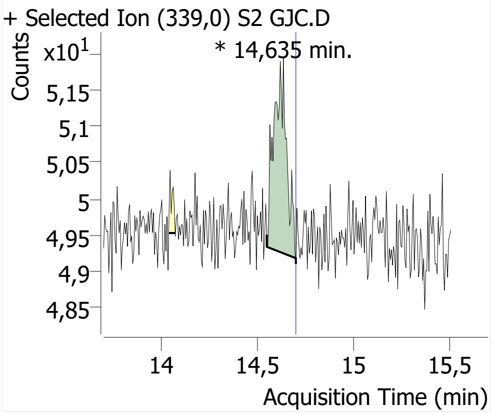

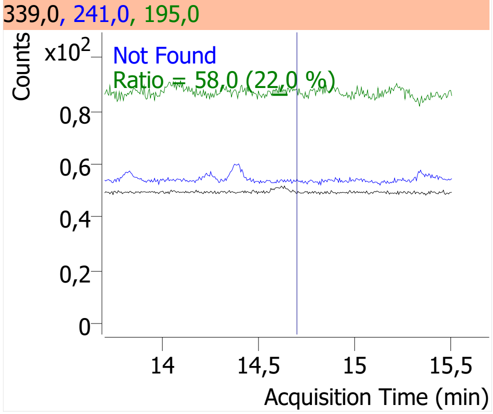

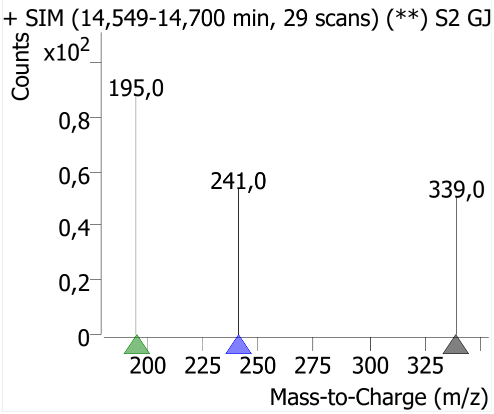

4,4-DDE

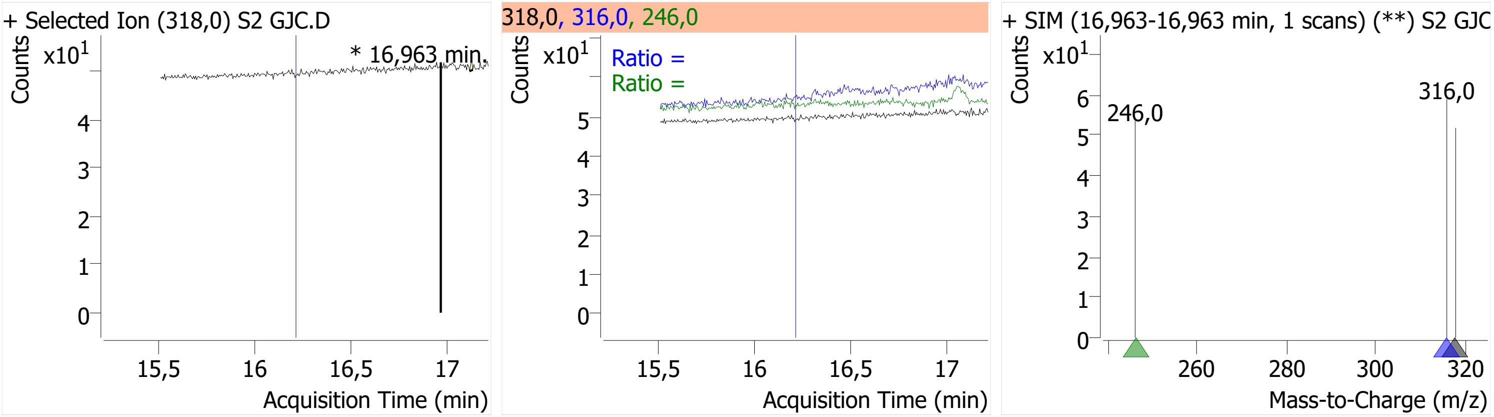

4,4-DDD

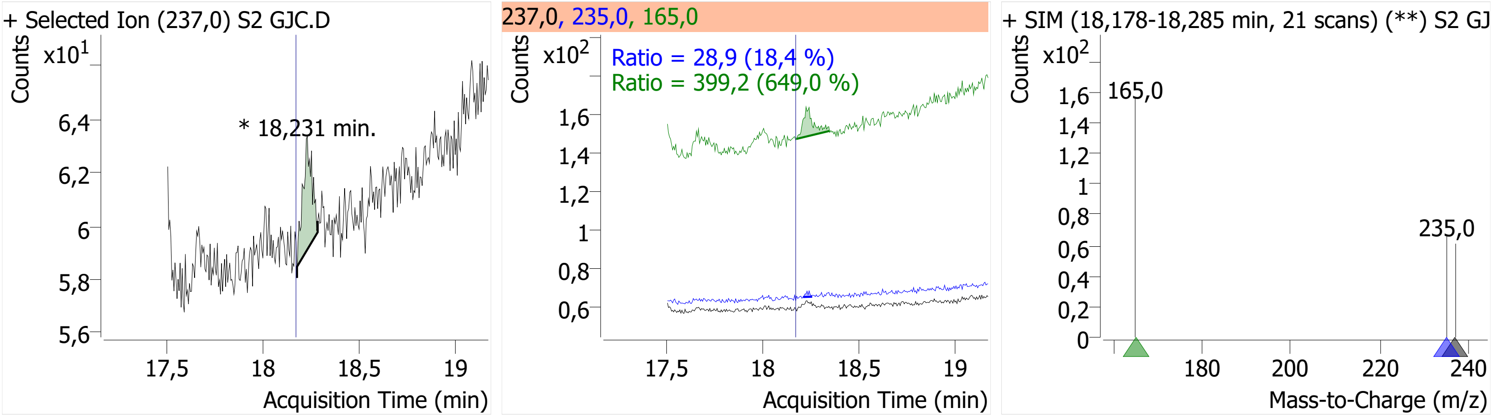

4,4-DDT

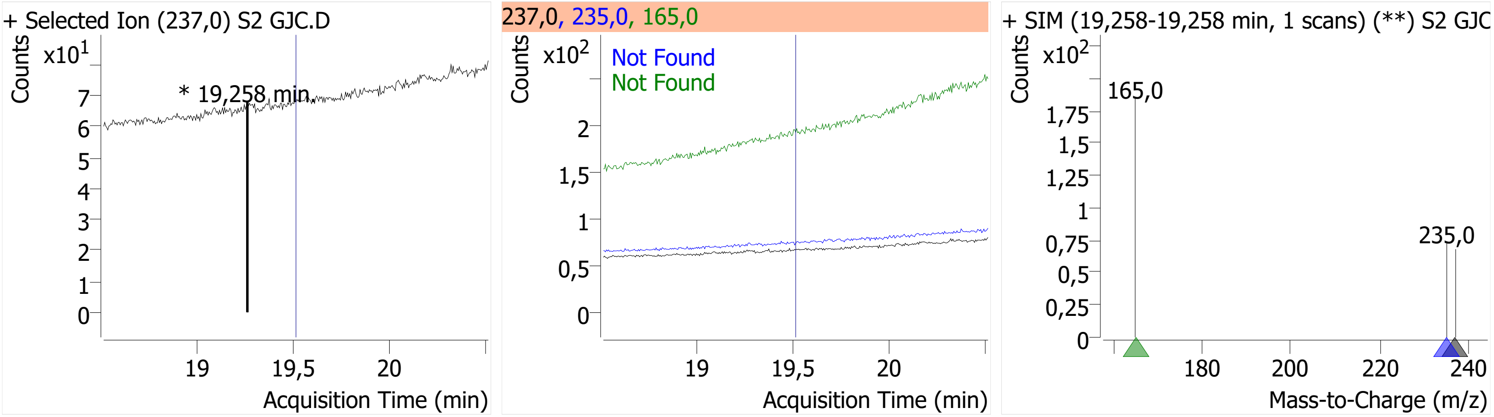

Quantitative Analysis Complete Report

Agilent Trusted Answers

**Batch Path**	C:\Users\USER\OneDrive\Desktop\JU_Pesticide\MA\MA\QuantResults\Mohammed_Pest.batch.bin		
**Analysis Time**	13 Dec 2024 11:34	**Analyst Name**	DESKTOP-MRRPPC4\USER
**Report Time**	13 Dec 2024 11:37:43	**Reporter Name**	DESKTOP-MRRPPC4\USER
**Last Calib Update**	13 Dec 2024 11:34	**Batch State**	Processed
**Quant Batch Version**	10.0	**Quant Report Version**	10.0

**Acq. Time**	13 Nov 2024 08:53	**Data File**	S1 SDT.D
**Sample Type**	Sample	**Sample Name**	Pest
**Dilution**	1	**Acq. Method**	pesticide std 12.11.2024

Sample Chromatogram

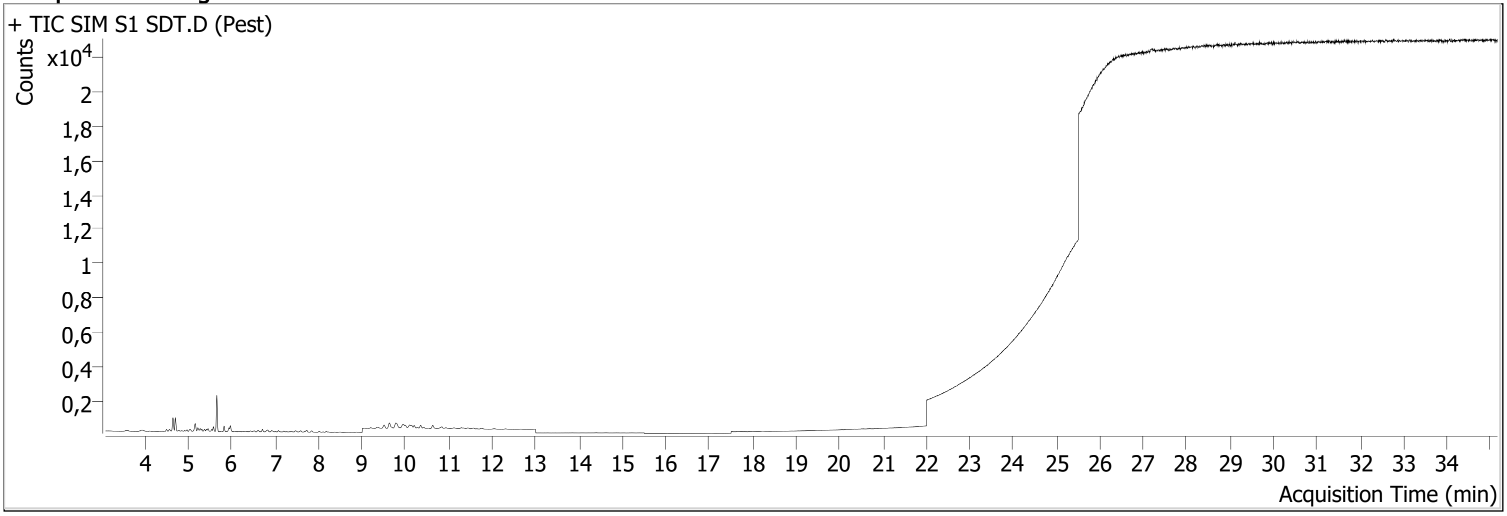

Compound	Transition	RT	Resp.	Final Conc	Units
Lindane	219,0	5,154	0	ND	ng/ml
Hexachlorobenzene	284,0	6,965	0	ND	ng/ml
Aldrine	293,0	11,282	0	ND	ng/ml
Chlorpyrifos	314,0	11,553	13	8,1930	ng/ml
Endosulfan II	339,0	14,603	21	14,7670	ng/ml
4,4-DDE	318,0	17,039	0	ND	ng/ml
4,4-DDD	237,0	18,237	13	3,7660	ng/ml
4,4-DDT	237,0			ND	ng/ml

Lindane

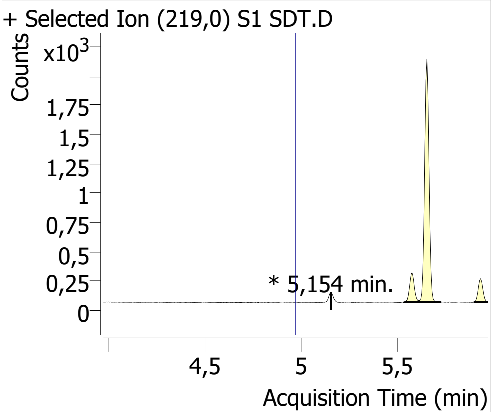

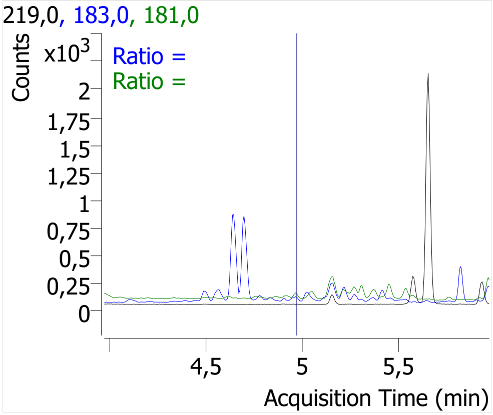

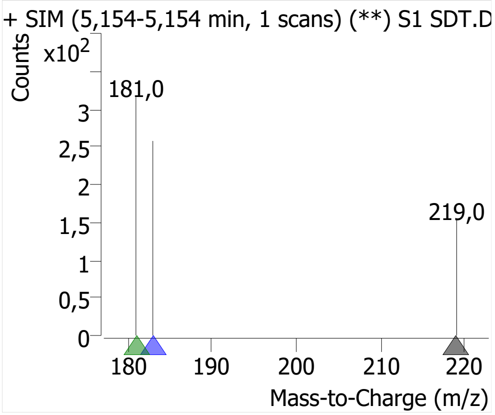

Hexachlorobenzene

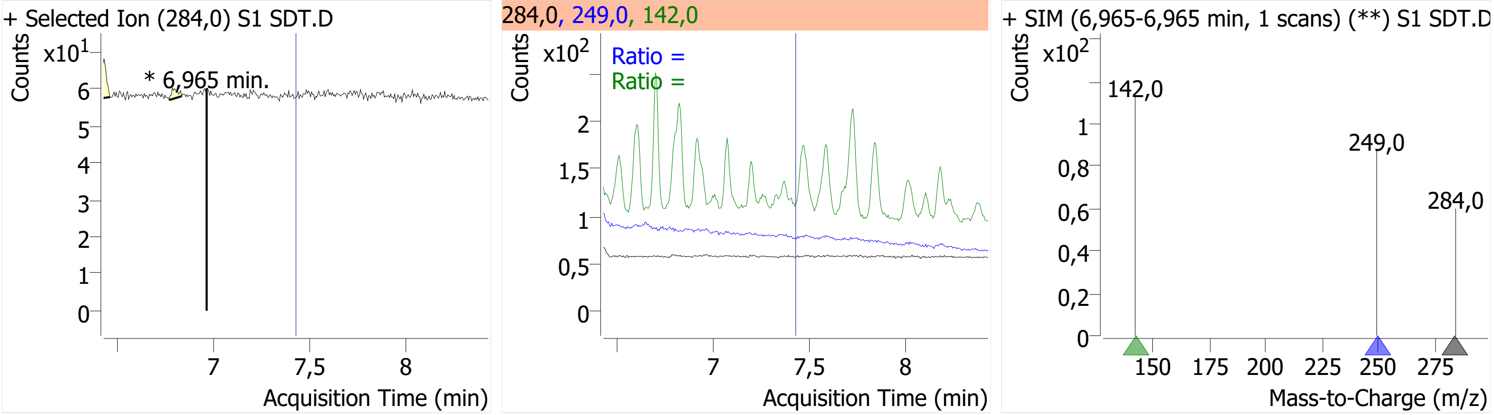

Aldrine

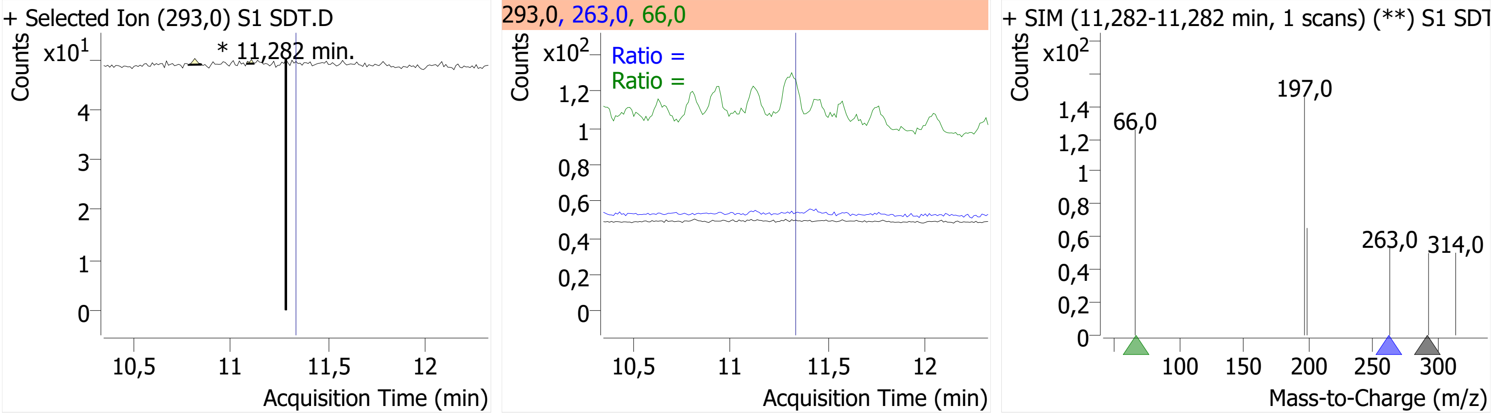

Chlorpyrifos

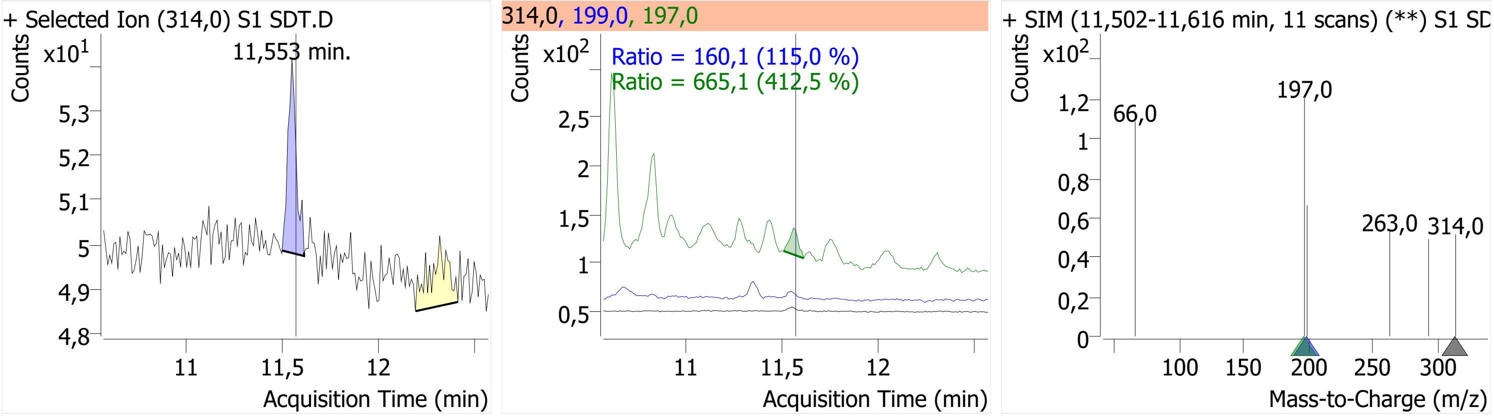

Endosulfan II

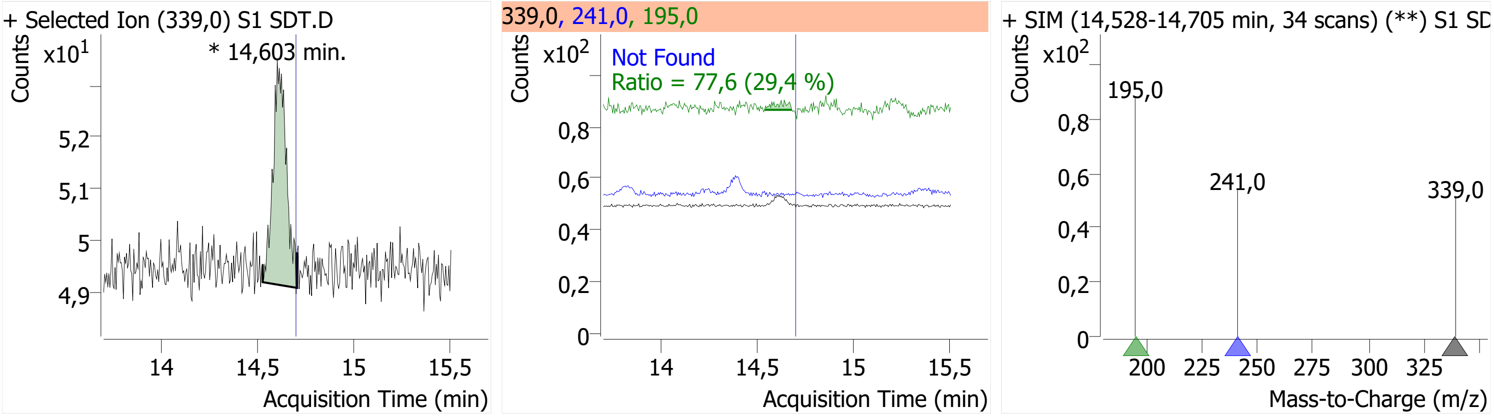

4,4-DDE

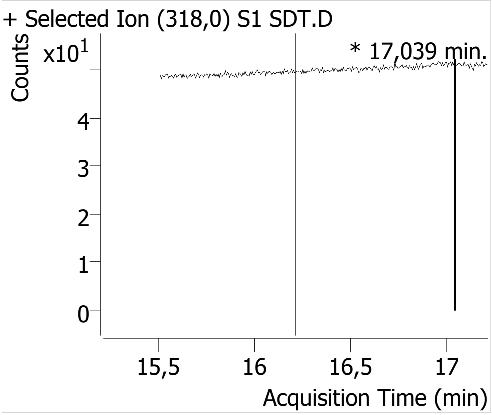

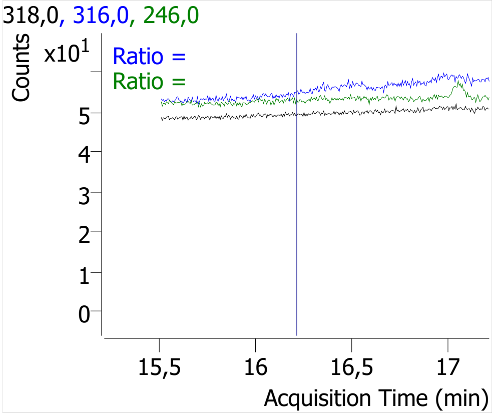

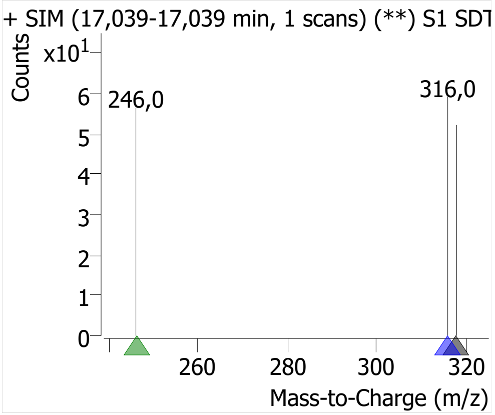

4,4-DDD

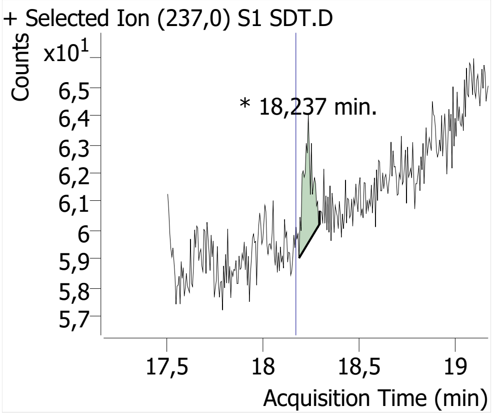

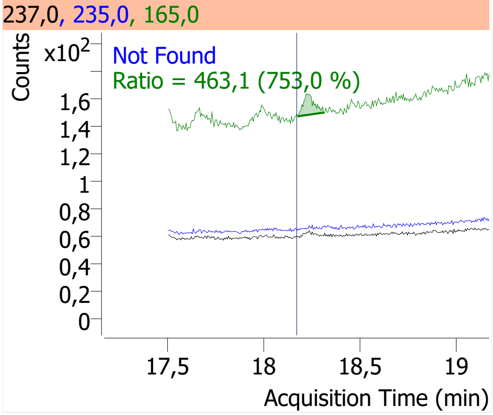

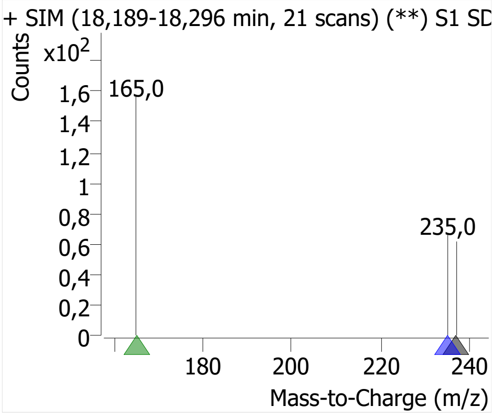

4,4-DDT

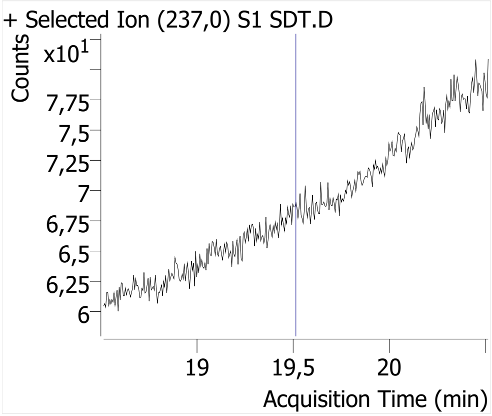

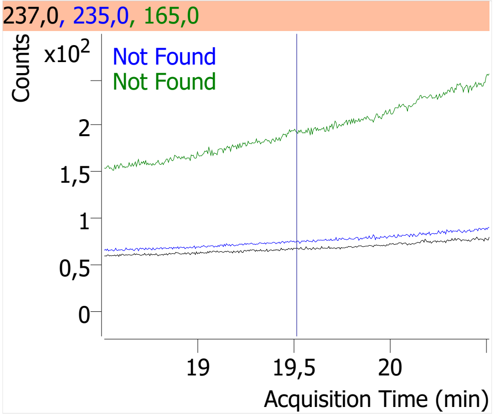

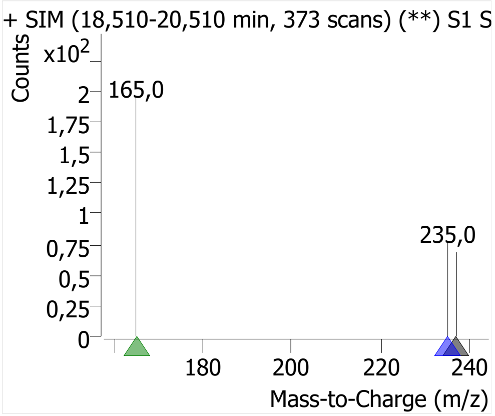

Quantitative Analysis Complete Report

**Batch Path**	C:\Users\USER\OneDrive\Desktop\JU_Pesticide\MA\MA\QuantResults\Mohammed_Pest.batch.bin		
**Analysis Time**	13 Dec 2024 11:34	**Analyst Name**	DESKTOP-MRRPPC4\USER
**Report Time**	13 Dec 2024 11:37:44	**Reporter Name**	DESKTOP-MRRPPC4\USER
**Last Calib Update**	13 Dec 2024 11:34	**Batch State**	Processed
**Quant Batch Version**	10.0	**Quant Report Version**	10.0

**Acq. Time**	13 Nov 2024 09:33	**Data File**	S1 DDC.D
**Sample Type**	Sample	**Sample Name**	Pest
**Dilution**	1	**Acq. Method**	pesticide std 12.11.2024

Sample Chromatogram

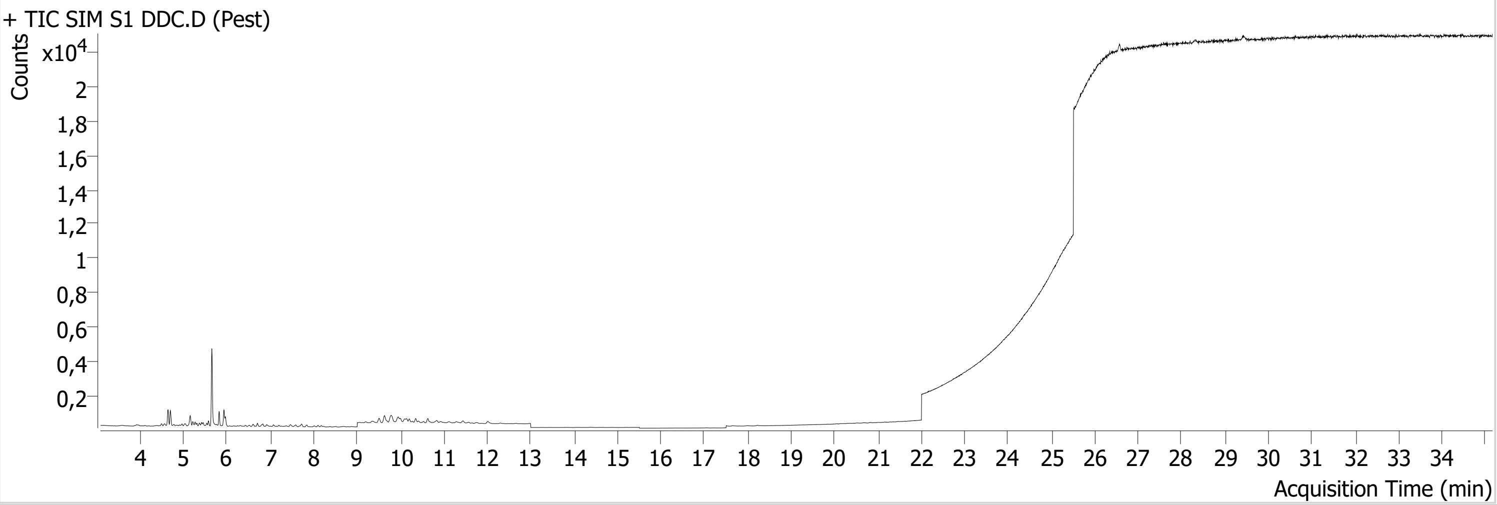

Compound	Transition	RT	Resp.	Final Conc	Units
Lindane	219,0	5,153	0	ND	ng/ml
Hexachlorobenzene	284,0	7,568	0	ND	ng/ml
Aldrine	293,0	11,407	0	ND	ng/ml
Chlorpyrifos	314,0			ND	ng/ml
Endosulfan II	339,0	14,598	0	ND	ng/ml
4,4-DDE	318,0	16,791	0	ND	ng/ml
4,4-DDD	237,0	18,226	19	4,8692	ng/ml
4,4-DDT	237,0	20,327	0	ND	ng/ml

Lindane

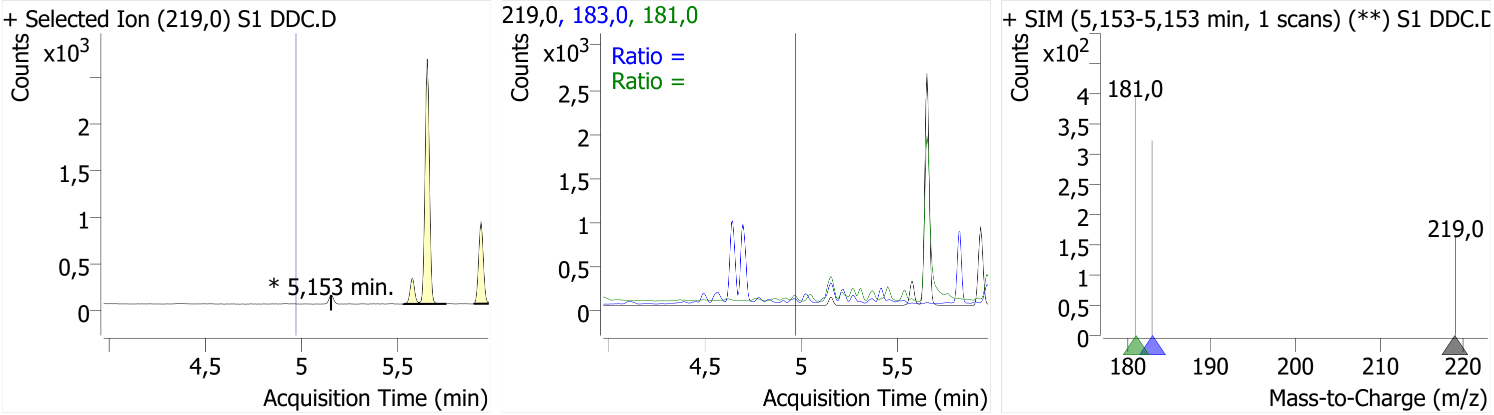

Hexachlorobenzene

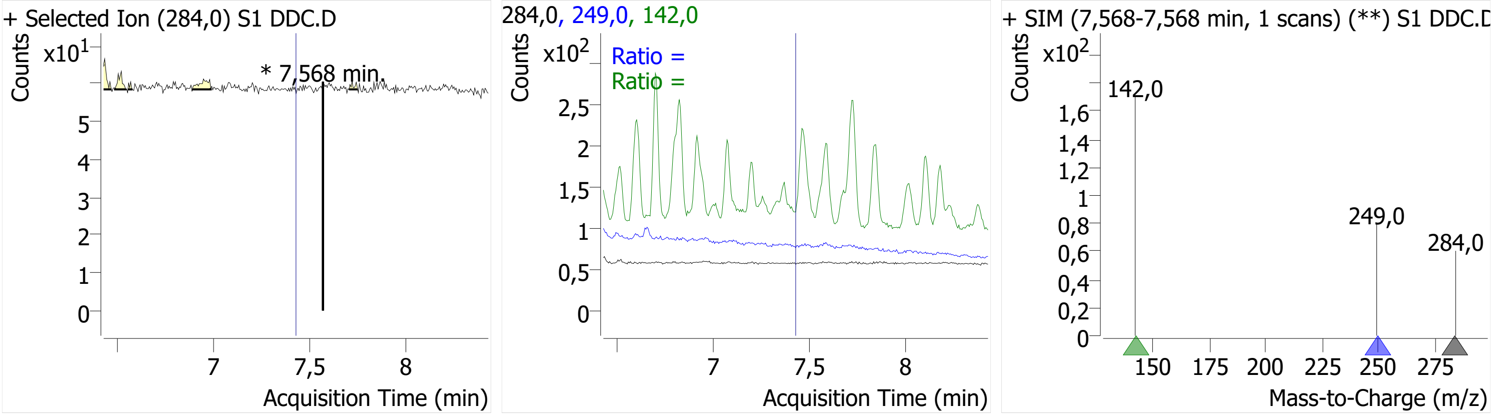

Aldrine

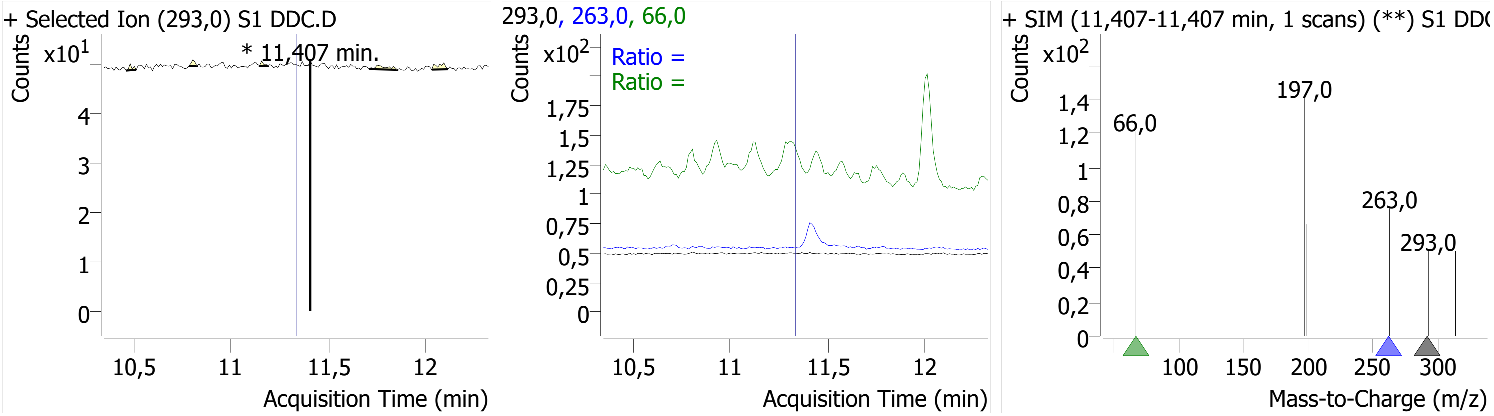

Chlorpyrifos

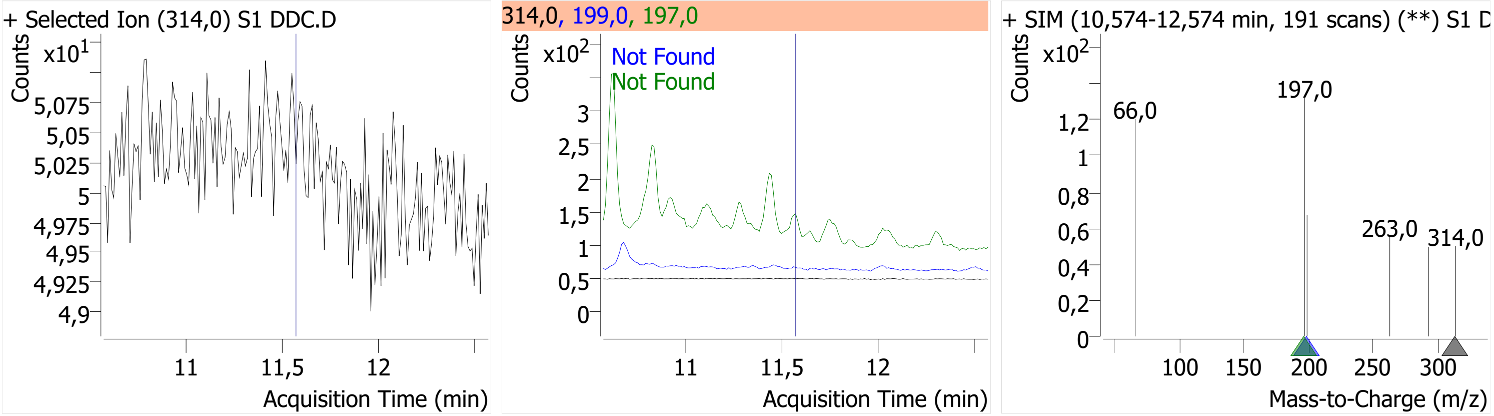

Endosulfan II

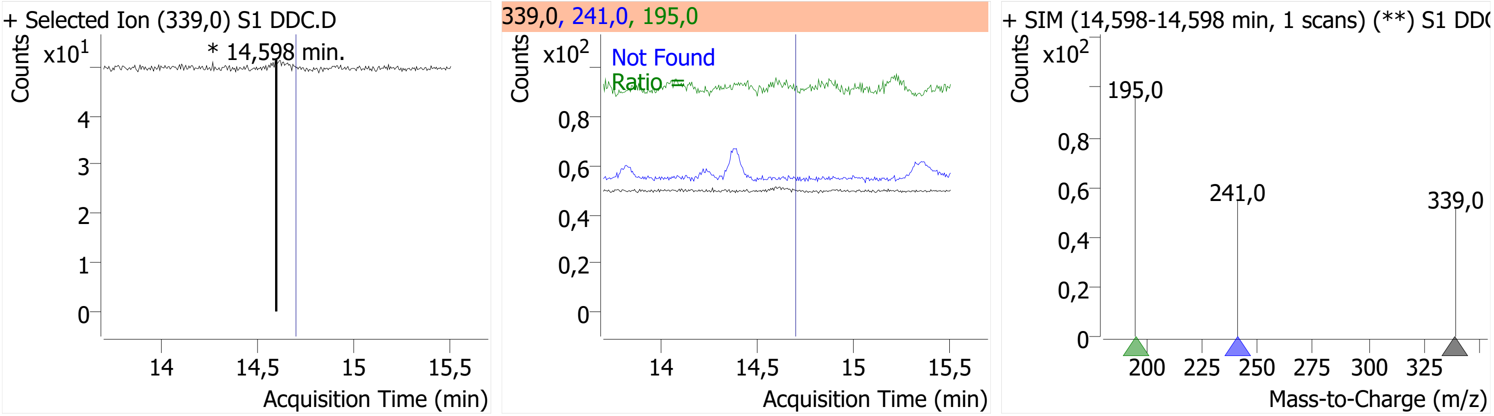

4,4-DDE

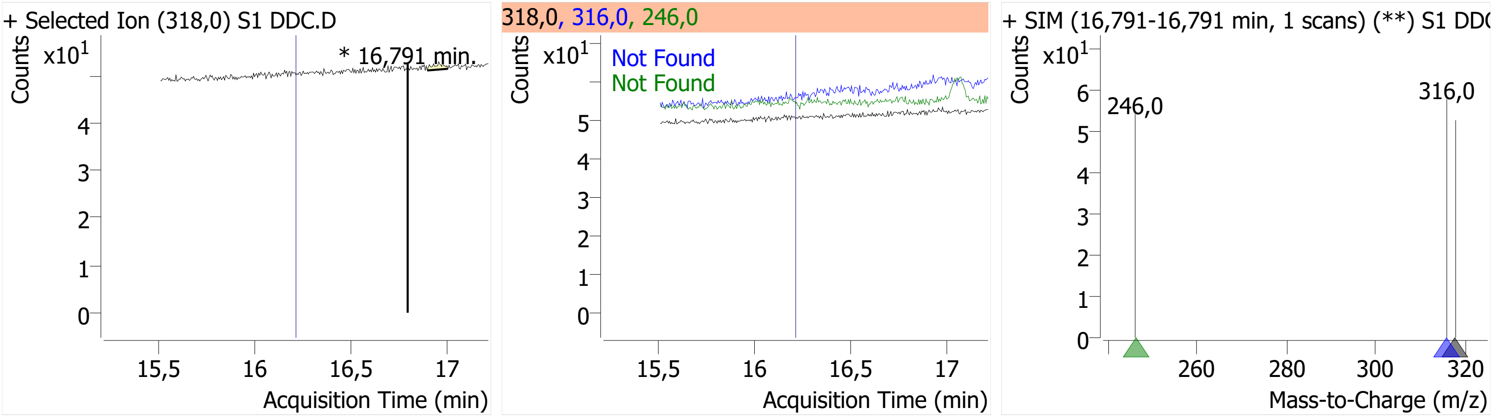

4,4-DDD

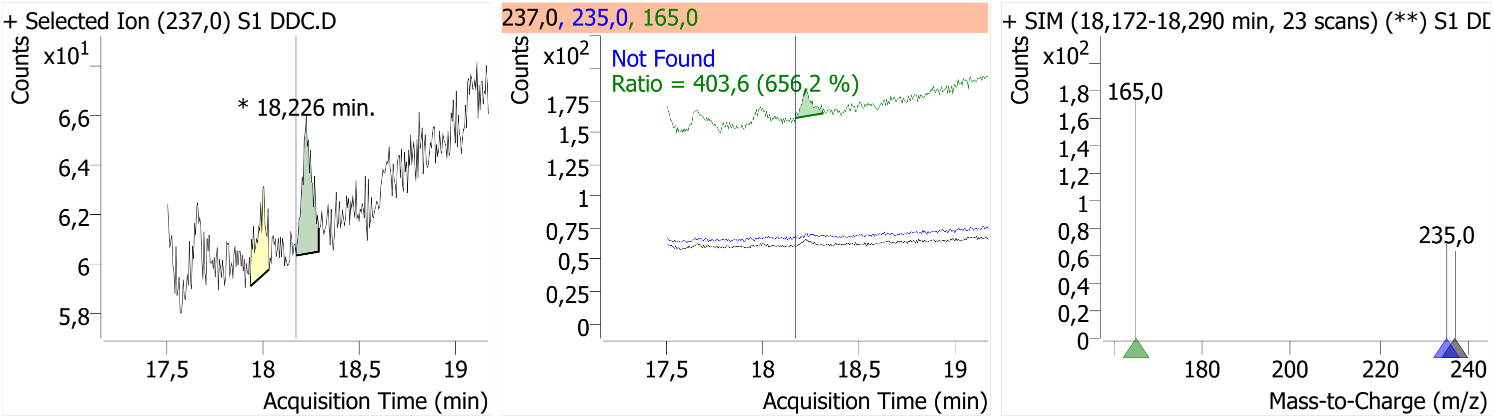

4,4-DDT

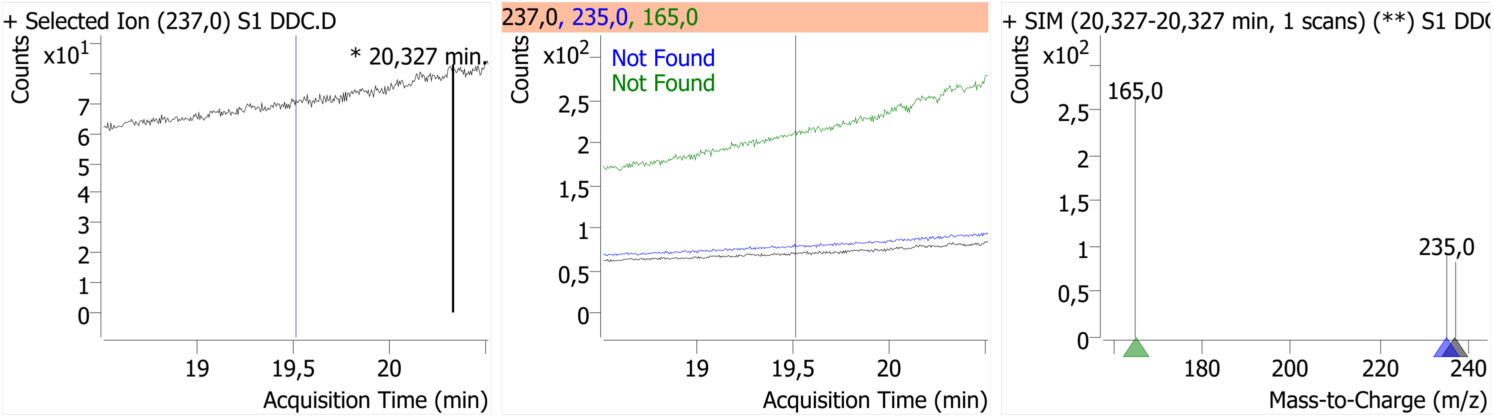

Quantitative Analysis Complete Report

**Batch Path**	C:\Users\USER\OneDrive\Desktop\JU_Pesticide\MA\MA\QuantResults\Mohammed_Pest.batch.bin		
**Analysis Time**	13 Dec 2024 11:34	**Analyst Name**	DESKTOP-MRRPPC4\USER
**Report Time**	13 Dec 2024 11:37:44	**Reporter Name**	DESKTOP-MRRPPC4\USER
**Last Calib Update**	13 Dec 2024 11:34	**Batch State**	Processed
**Quant Batch Version**	10.0	**Quant Report Version**	10.0

**Acq. Time**	13 Nov 2024 10:14	**Data File**	S1 DOC.D
**Sample Type**	Sample	**Sample Name**	Pest
**Dilution**	1	**Acq. Method**	pesticide std 12.11.2024

Sample Chromatogram

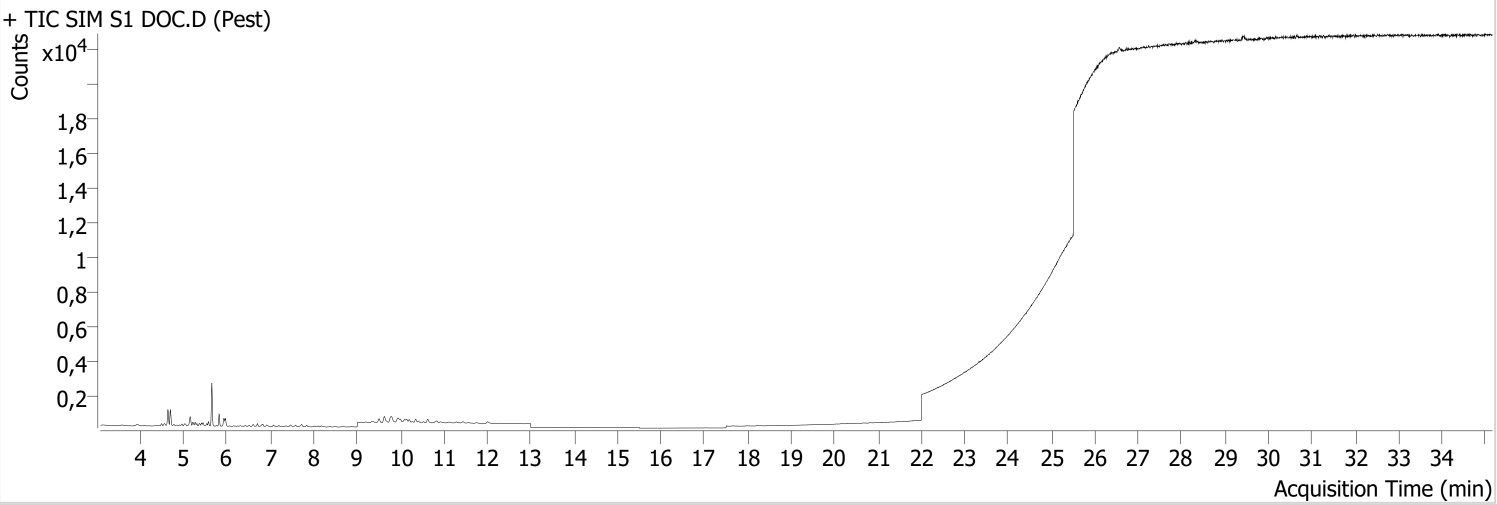

Compound	Transition	RT	Resp.	Final Conc	Units
Lindane	219,0	5,154	0	ND	ng/ml
Hexachlorobenzene	284,0	8,015	0	ND	ng/ml
Aldrine	293,0	11,334	0	ND	ng/ml
Chlorpyrifos	314,0	12,326	0	ND	ng/ml
Endosulfan II	339,0	14,614	5	ND	ng/ml
4,4-DDE	318,0	16,217	0	ND	ng/ml
4,4-DDD	237,0	18,221	15	4,0880	ng/ml
4,4-DDT	237,0	20,031	0	ND	ng/ml

Lindane

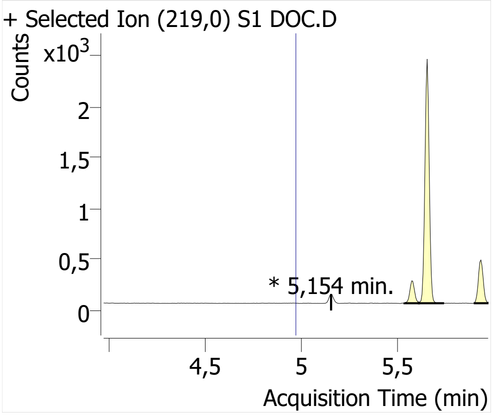

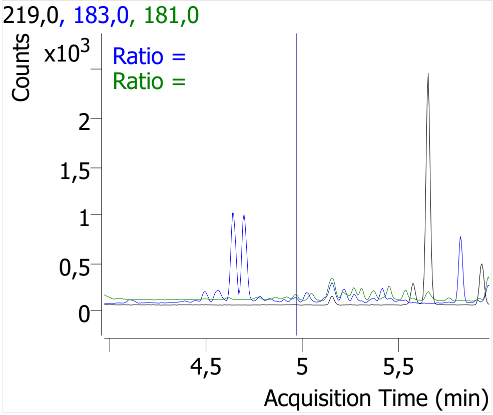

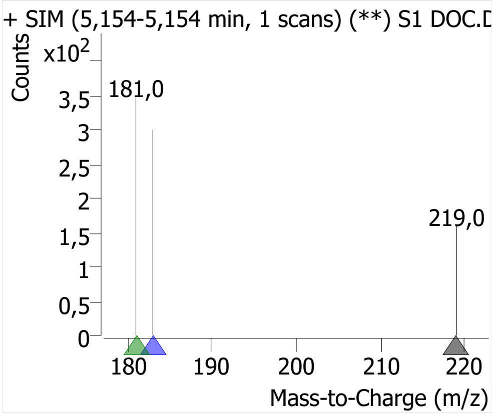

Hexachlorobenzene

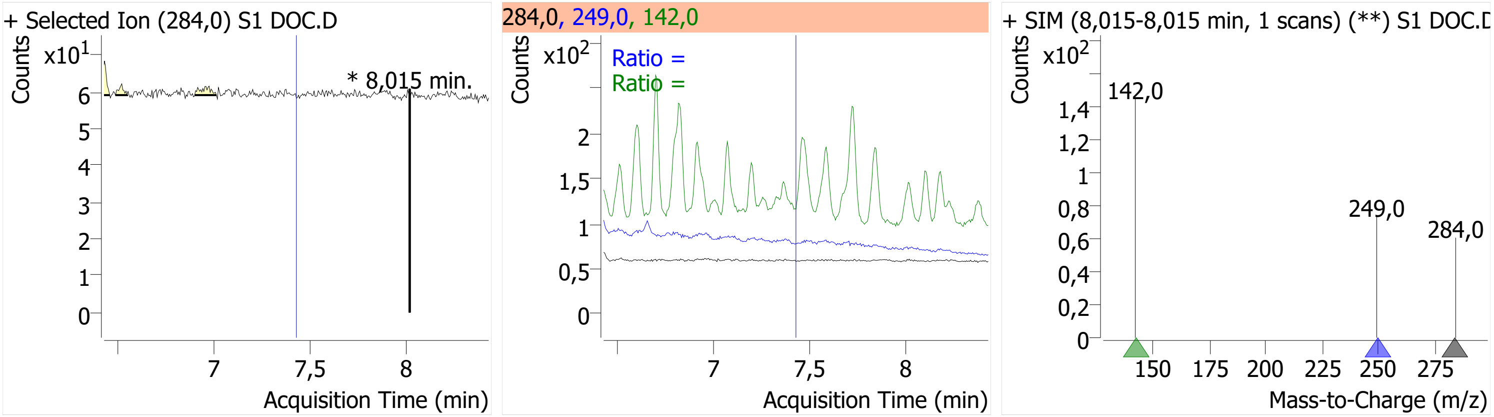

Aldrine

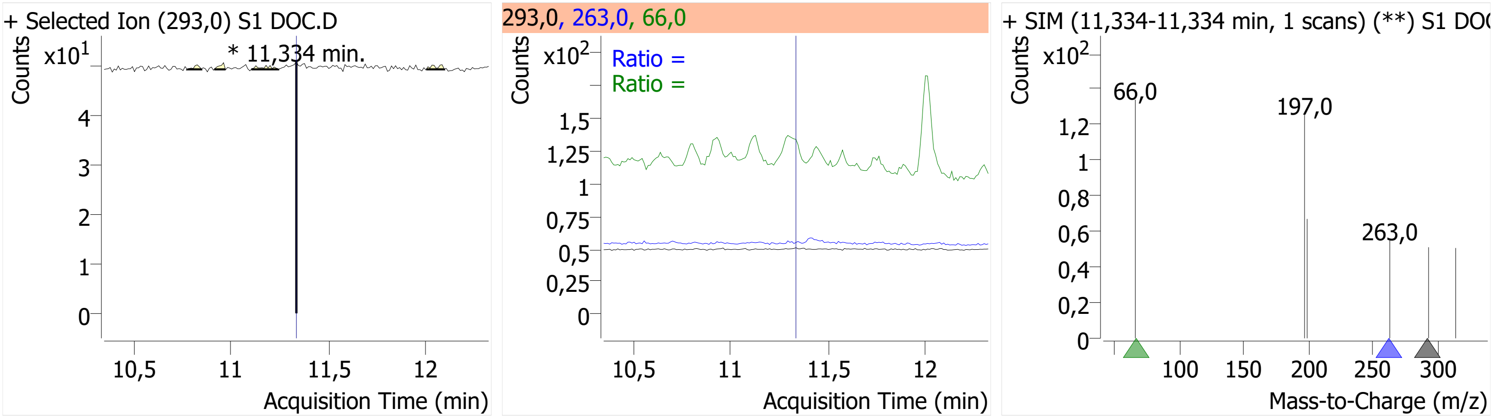

Chlorpyrifos

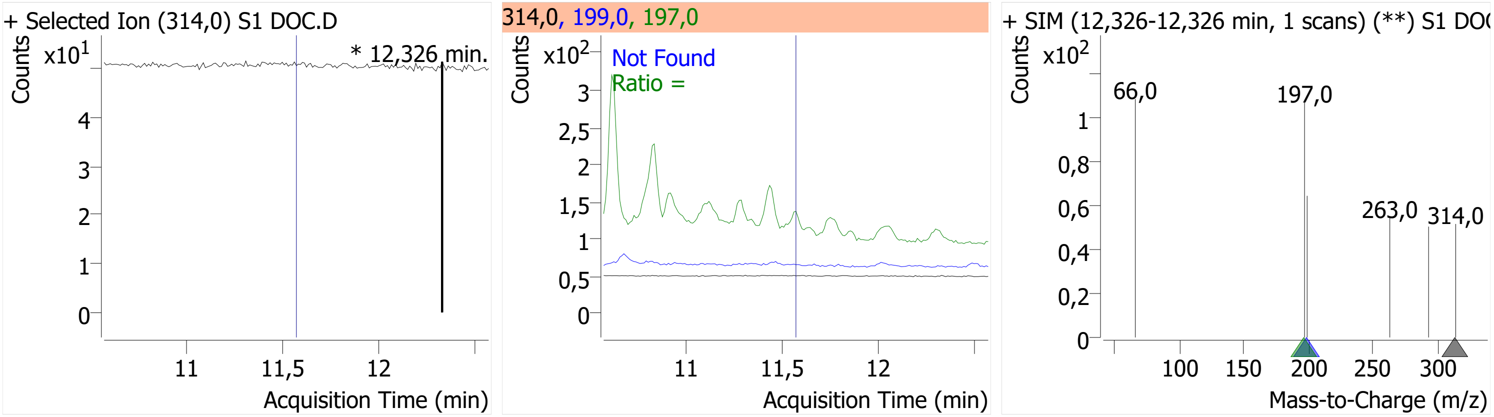

Endosulfan II

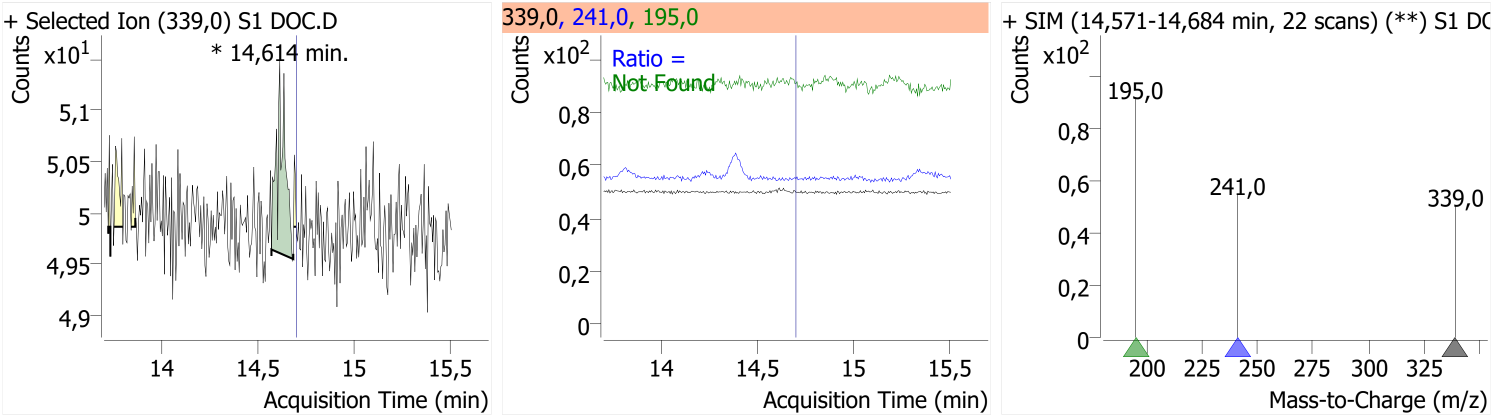

4,4-DDE

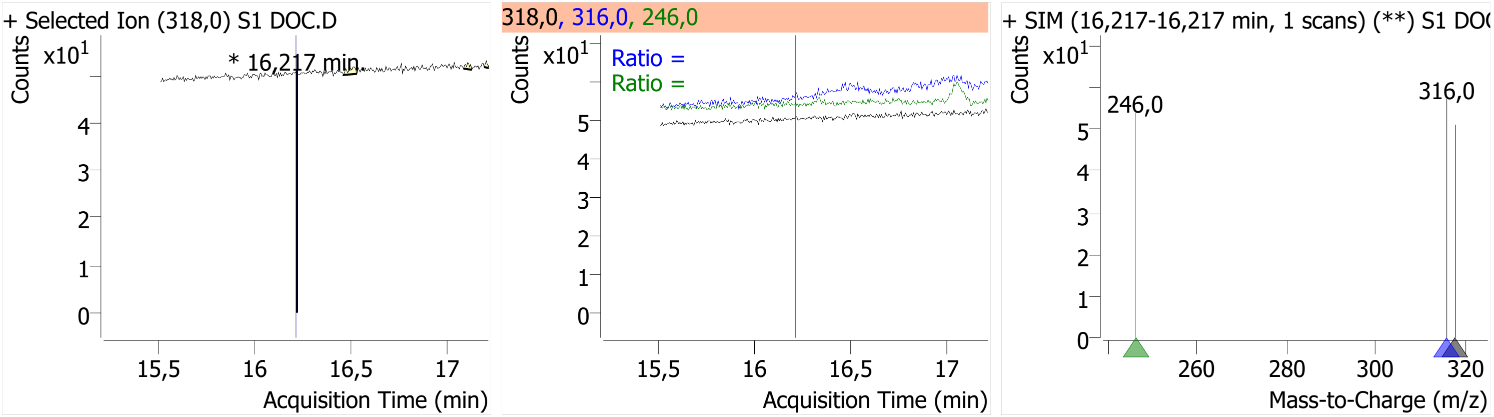

4,4-DDD

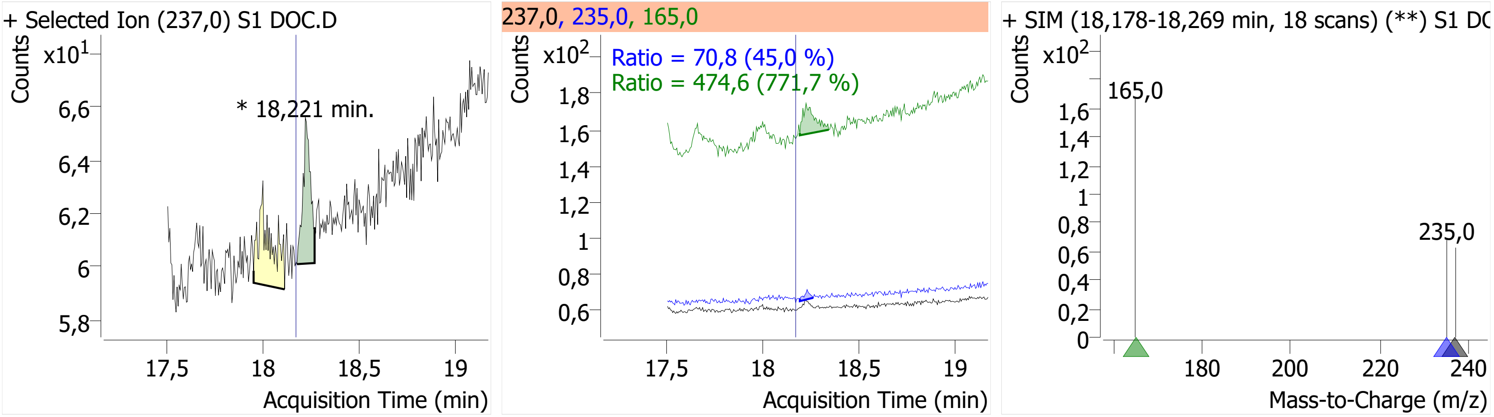

4,4-DDT

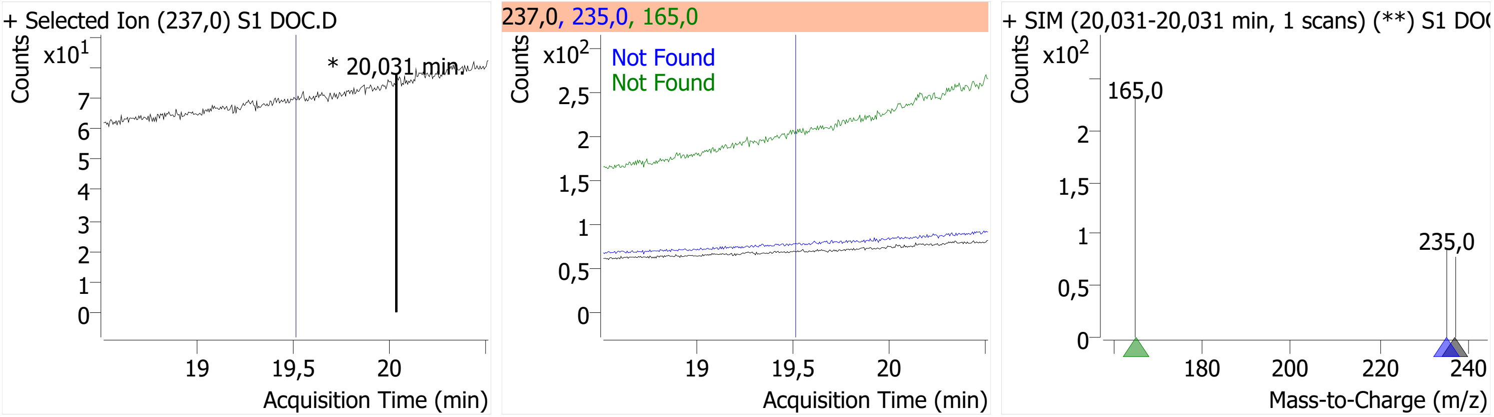

Quantitative Analysis Complete Report

**Batch Path**	C:\Users\USER\OneDrive\Desktop\JU_Pesticide\MA\MA\QuantResults\Mohammed_Pest.batch.bin		
**Analysis Time**	13 Dec 2024 11:34	**Analyst Name**	DESKTOP-MRRPPC4\USER
**Report Time**	13 Dec 2024 11:37:45	**Reporter Name**	DESKTOP-MRRPPC4\USER
**Last Calib Update**	13 Dec 2024 11:34	**Batch State**	Processed
**Quant Batch Version**	10.0	**Quant Report Version**	10.0

**Acq. Time**	13 Nov 2024 10:55	**Data File**	S2 GGP.D
**Sample Type**	Sample	**Sample Name**	Pest
**Dilution**	1	**Acq. Method**	pesticide std 12.11.2024

Sample Chromatogram

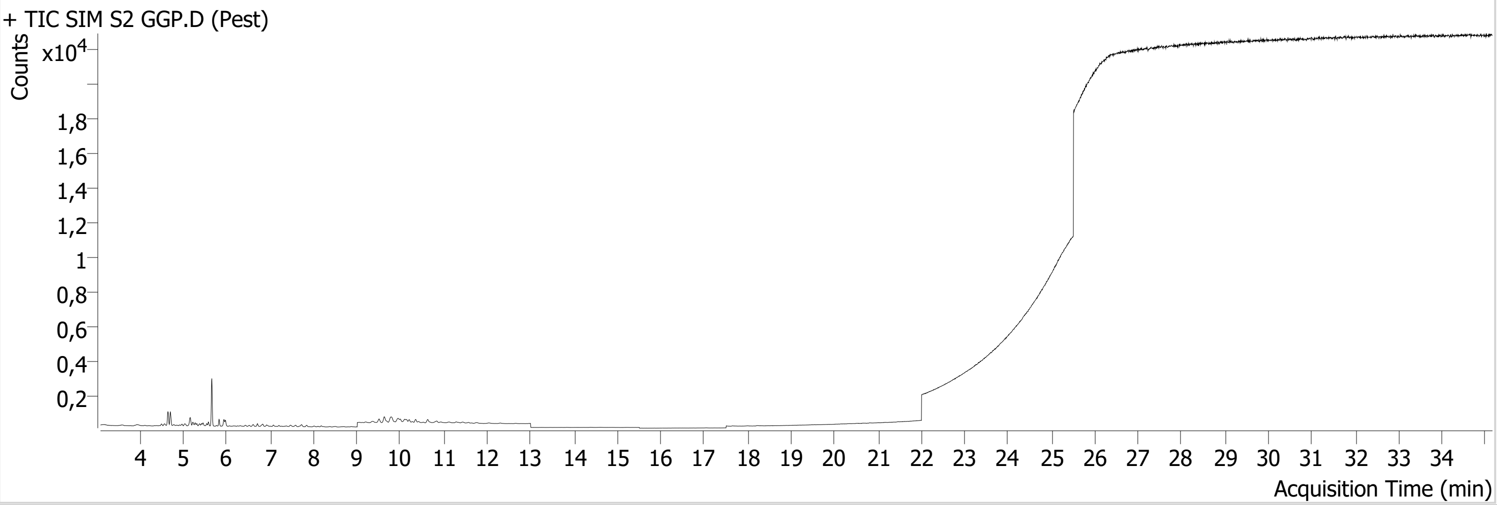

Compound	Transition	RT	Resp.	Final Conc	Units
Lindane	219,0	5,154	0	ND	ng/ml
Hexachlorobenzene	284,0	6,432	0	ND	ng/ml
Aldrine	293,0	11,146	0	ND	ng/ml
Chlorpyrifos	314,0	11,135	0	ND	ng/ml
Endosulfan II	339,0	14,625	22	16,6110	ng/ml
4,4-DDE	318,0	15,975	0	ND	ng/ml
4,4-DDD	237,0	18,226	17	4,4756	ng/ml
4,4-DDT	237,0			ND	ng/ml

Lindane

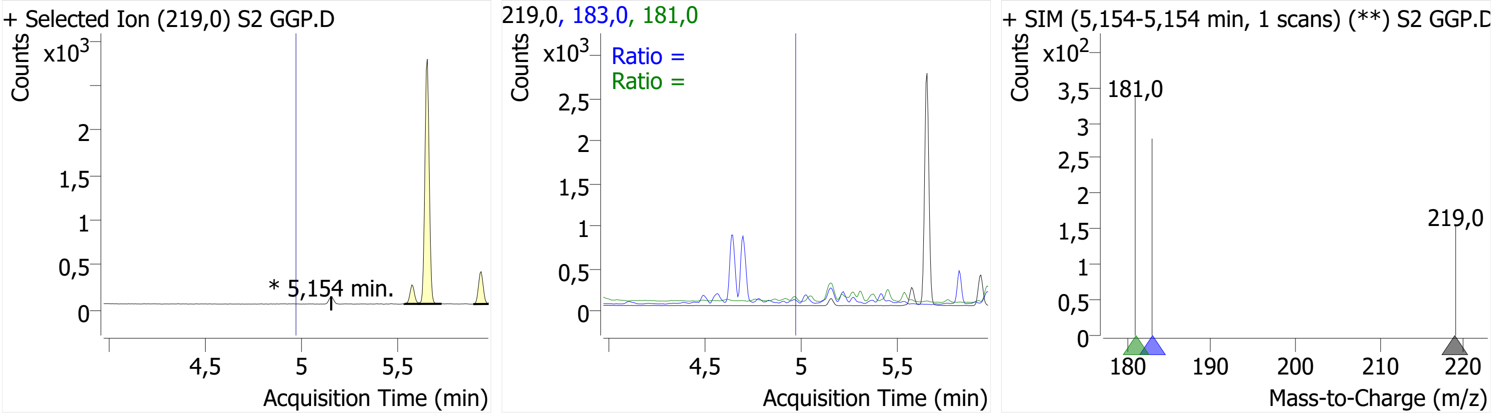

Hexachlorobenzene

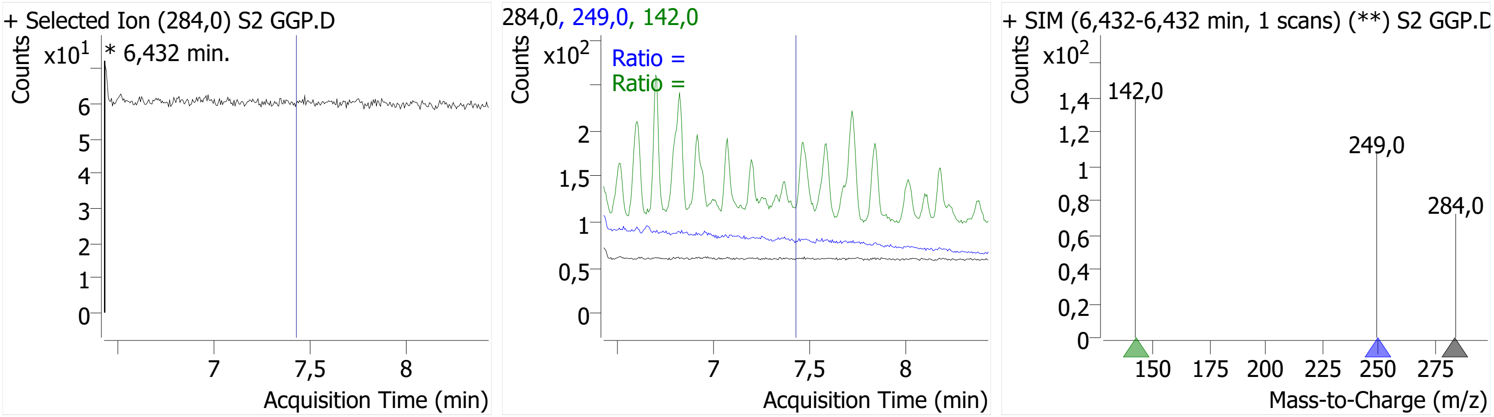

Aldrine

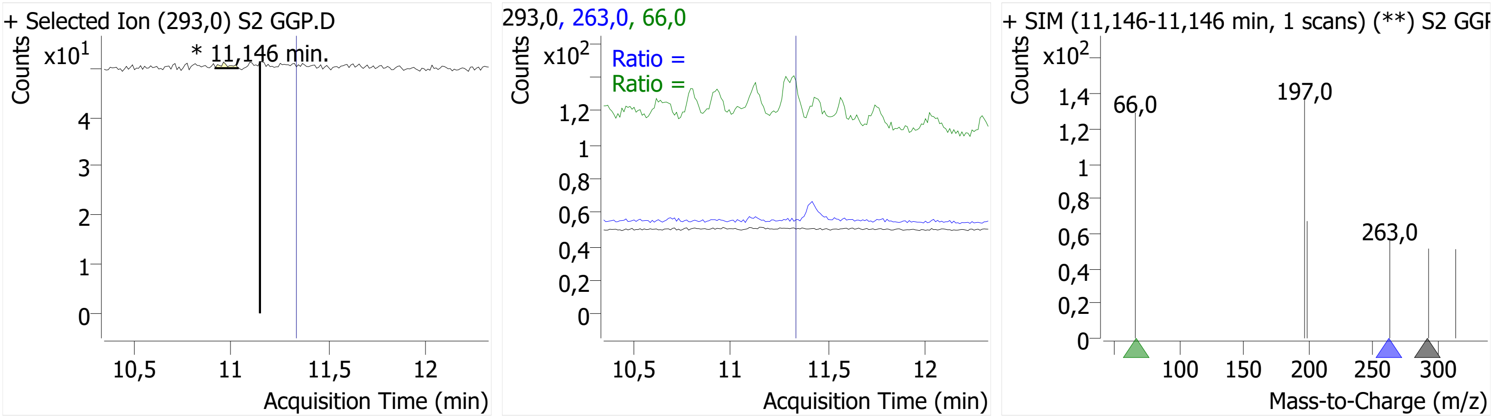

Chlorpyrifos

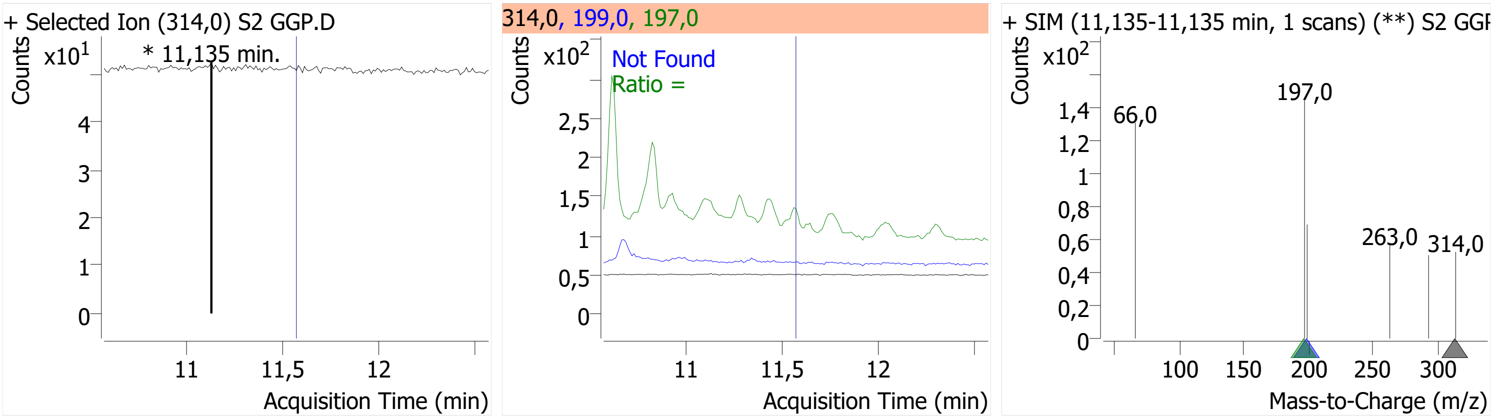

Endosulfan II

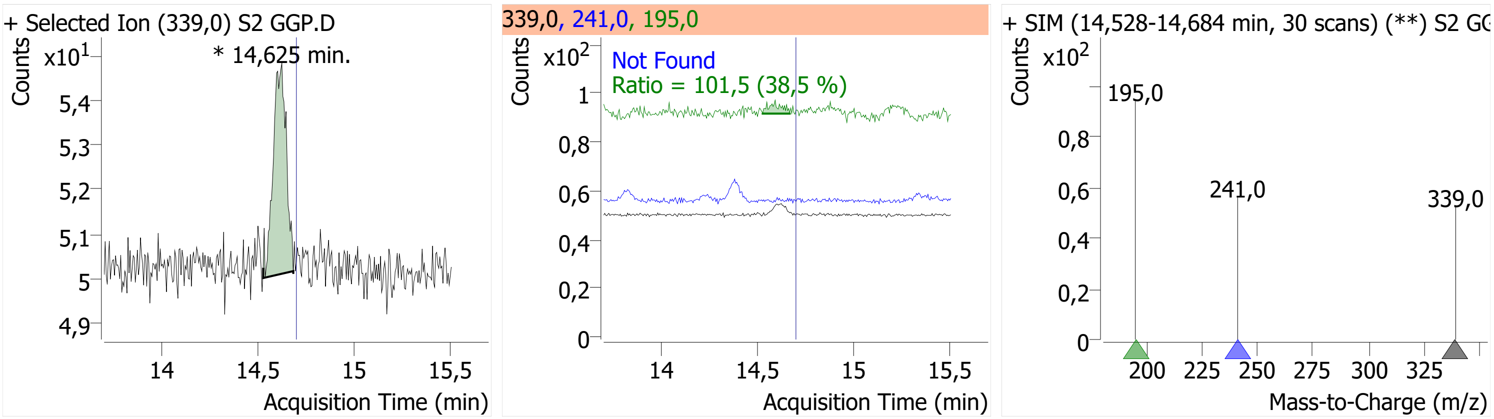

4,4-DDE

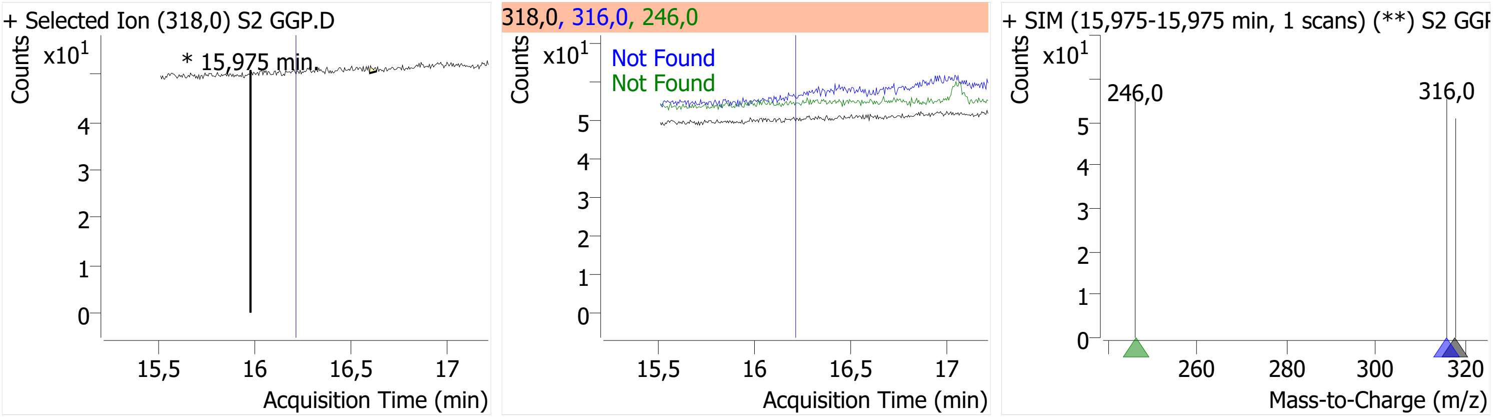

4,4-DDD

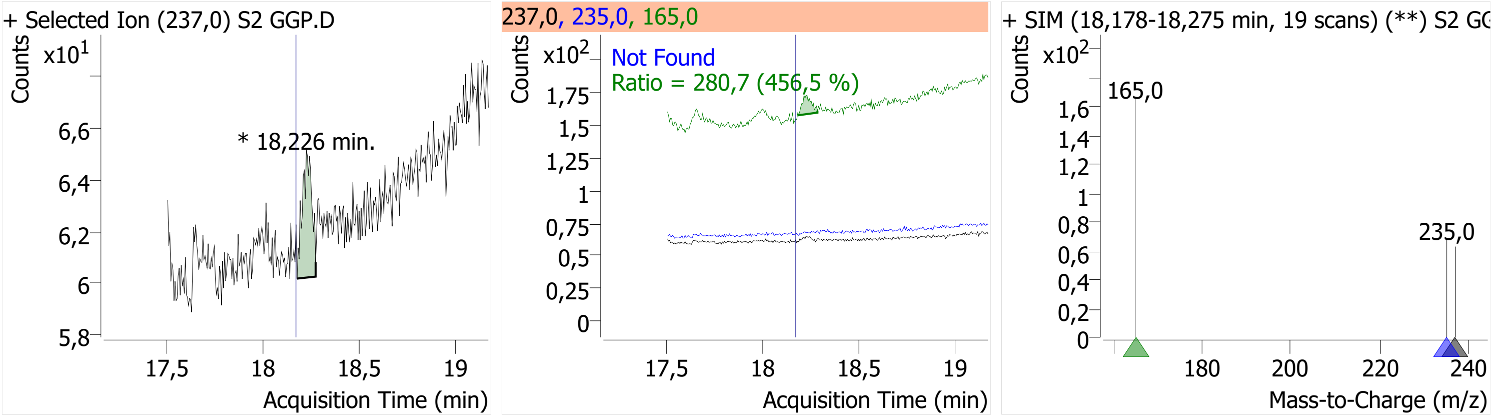

4,4-DDT

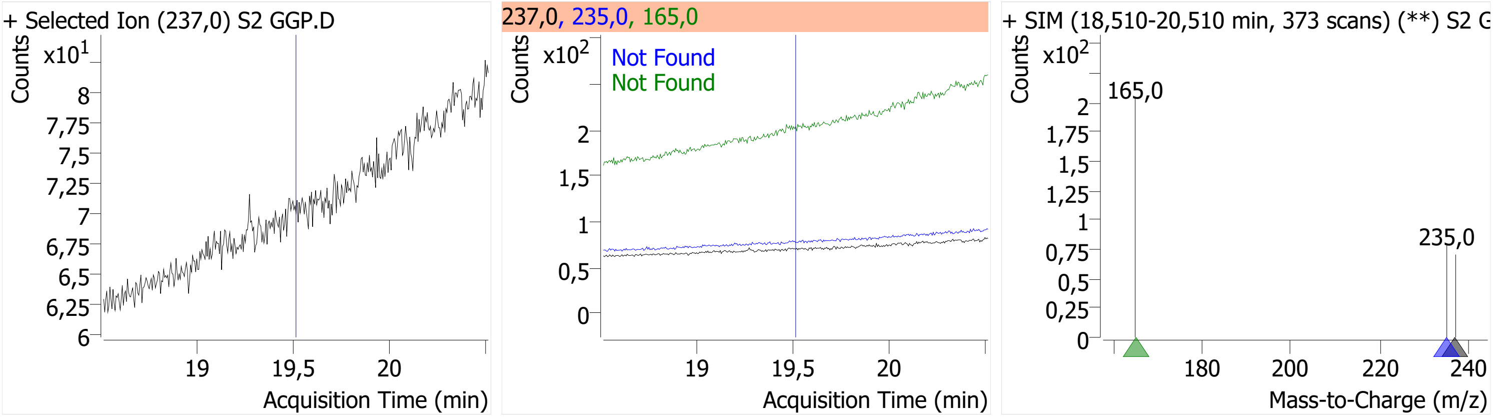

Quantitative Analysis Complete Report

**Batch Path**	C:\Users\USER\OneDrive\Desktop\JU_Pesticide\MA\MA\QuantResults\Mohammed_Pest.batch.bin		
**Analysis Time**	13 Dec 2024 11:34	**Analyst Name**	DESKTOP-MRRPPC4\USER
**Report Time**	13 Dec 2024 11:37:46	**Reporter Name**	DESKTOP-MRRPPC4\USER
**Last Calib Update**	13 Dec 2024 11:34	**Batch State**	Processed
**Quant Batch Version**	10.0	**Quant Report Version**	10.0

**Acq. Time**	13 Nov 2024 11:35	**Data File**	S1 SDC.D
**Sample Type**	Sample	**Sample Name**	Pest
**Dilution**	1	**Acq. Method**	pesticide std 12.11.2024

Sample Chromatogram

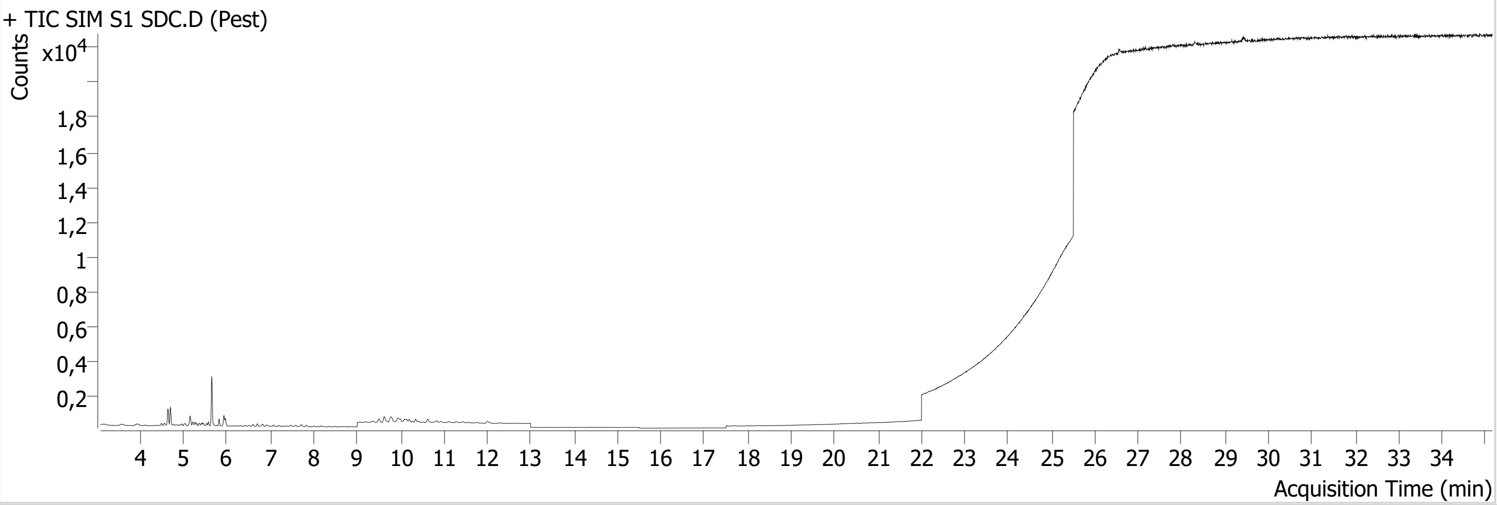

Compound	Transition	RT	Resp.	Final Conc	Units
Lindane	219,0	5,153	0	ND	ng/ml
Hexachlorobenzene	284,0	6,438	0	ND	ng/ml
Aldrine	293,0	10,801	0	ND	ng/ml
Chlorpyrifos	314,0			ND	ng/ml
Endosulfan II	339,0	14,614	35	29,4546	ng/ml
4,4-DDE	318,0			ND	ng/ml
4,4-DDD	237,0	18,226	16	4,2089	ng/ml
4,4-DDT	237,0			ND	ng/ml

Lindane

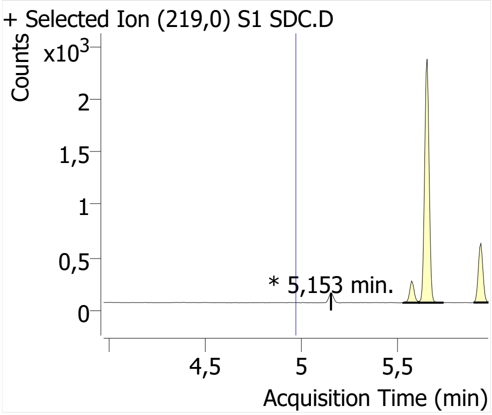

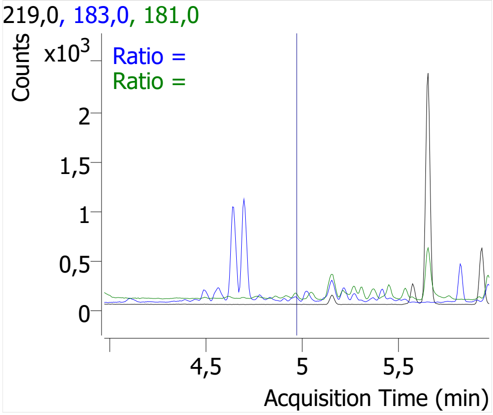

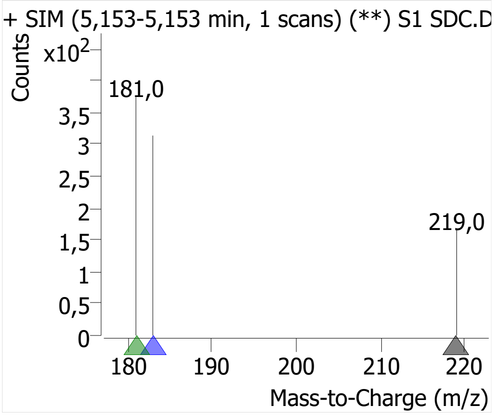

Hexachlorobenzene

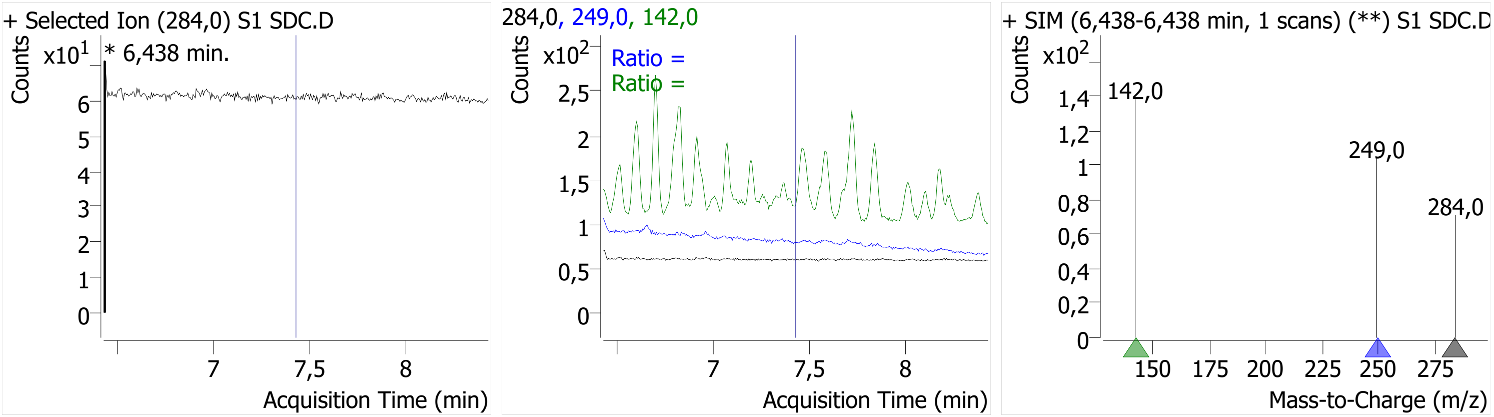

Aldrine

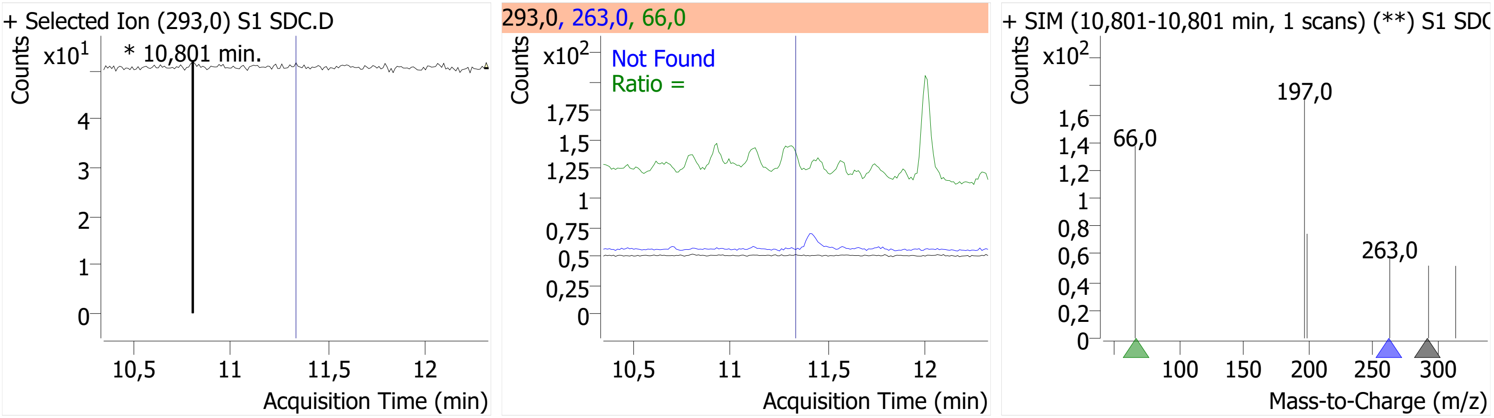

Chlorpyrifos

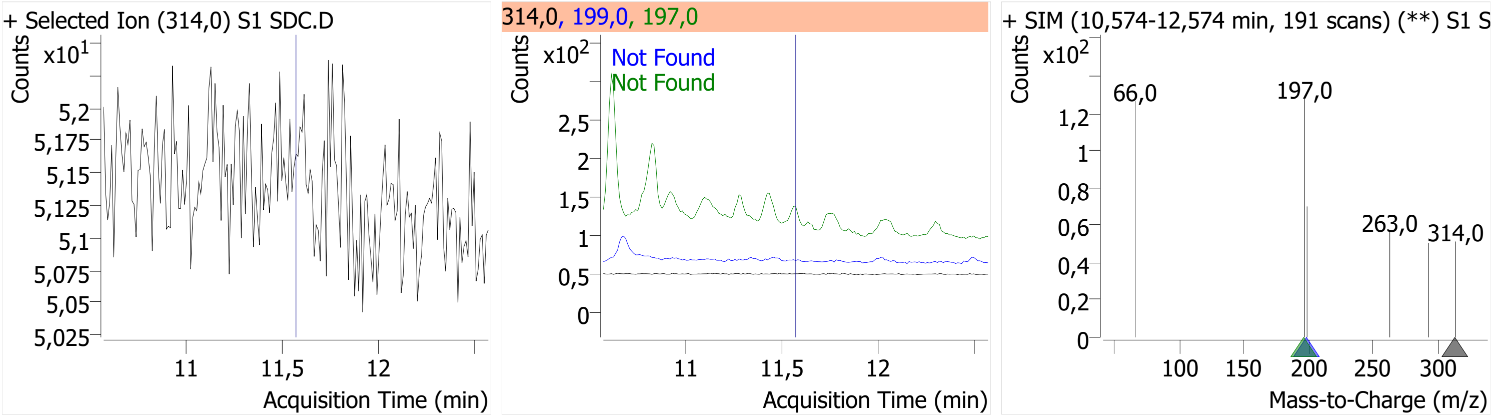

Endosulfan II

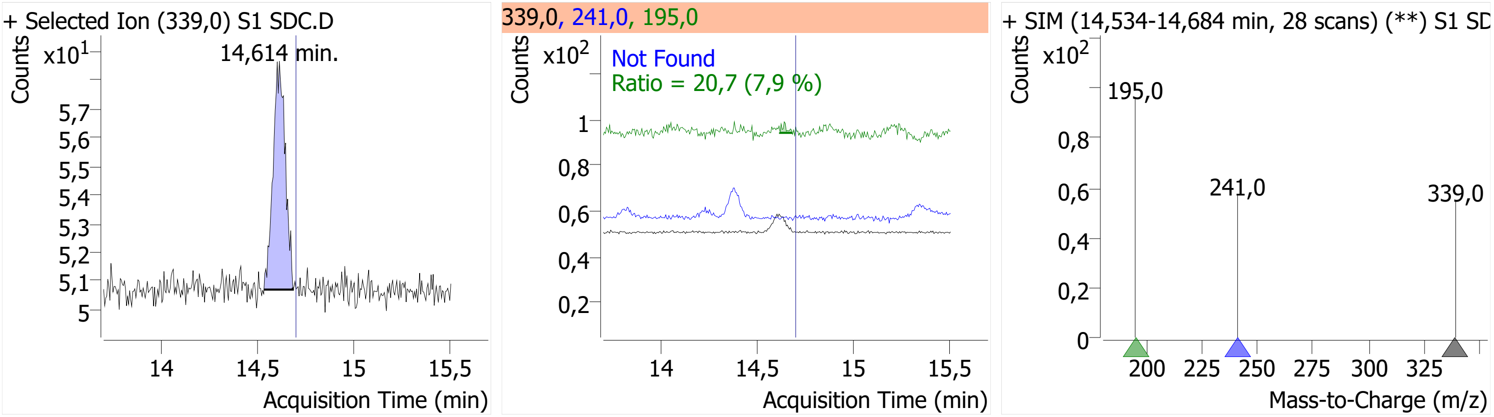

4,4-DDE

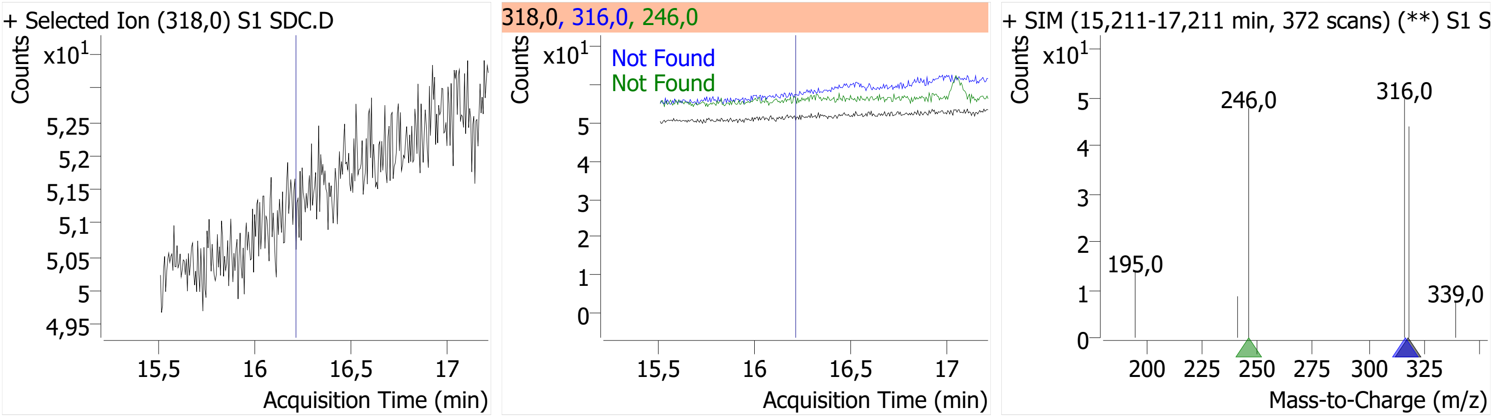

4,4-DDD

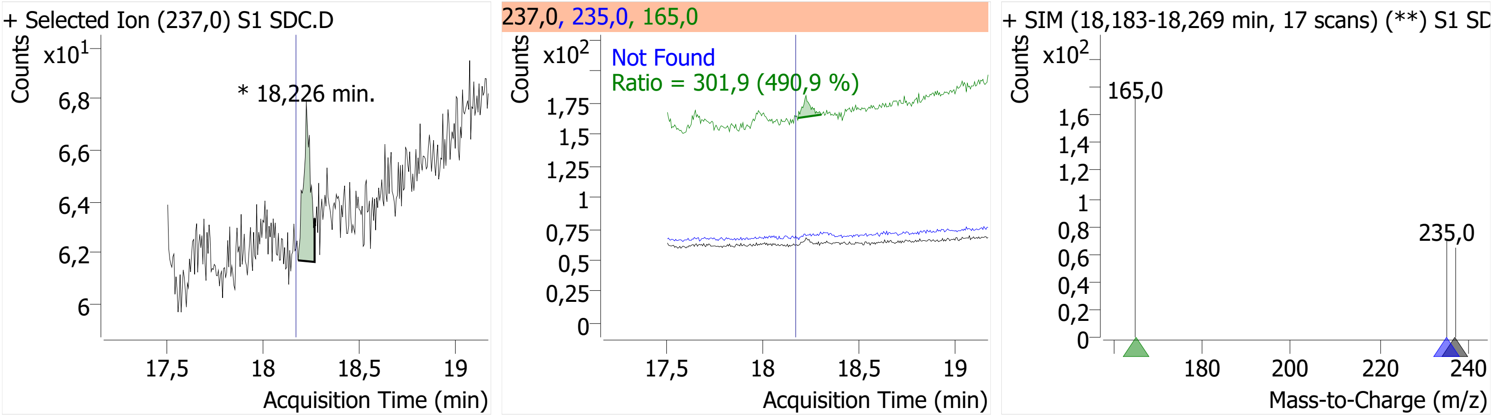

4,4-DDT

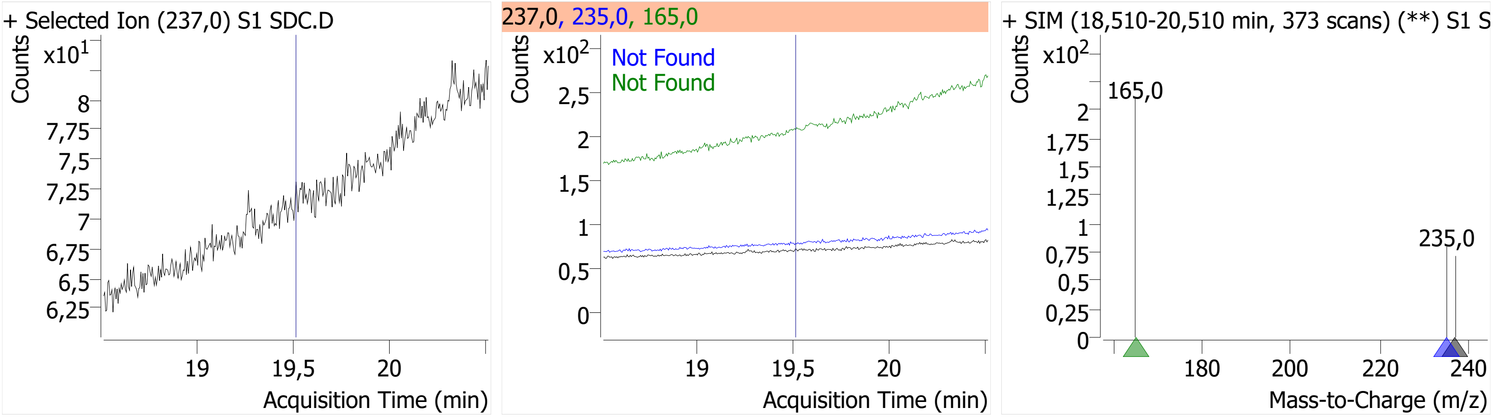

Quantitative Analysis Complete Report

**Batch Path**	C:\Users\USER\OneDrive\Desktop\JU_Pesticide\MA\MA\QuantResults\Mohammed_Pest.batch.bin		
**Analysis Time**	13 Dec 2024 11:34	**Analyst Name**	DESKTOP-MRRPPC4\USER
**Report Time**	13 Dec 2024 11:37:47	**Reporter Name**	DESKTOP-MRRPPC4\USER
**Last Calib Update**	13 Dec 2024 11:34	**Batch State**	Processed
**Quant Batch Version**	10.0	**Quant Report Version**	10.0

**Acq. Time**	13 Nov 2024 12:16	**Data File**	S1 GGC-1.D
**Sample Type**	Sample	**Sample Name**	Pest
**Dilution**	1	**Acq. Method**	pesticide std 12.11.2024

Sample Chromatogram

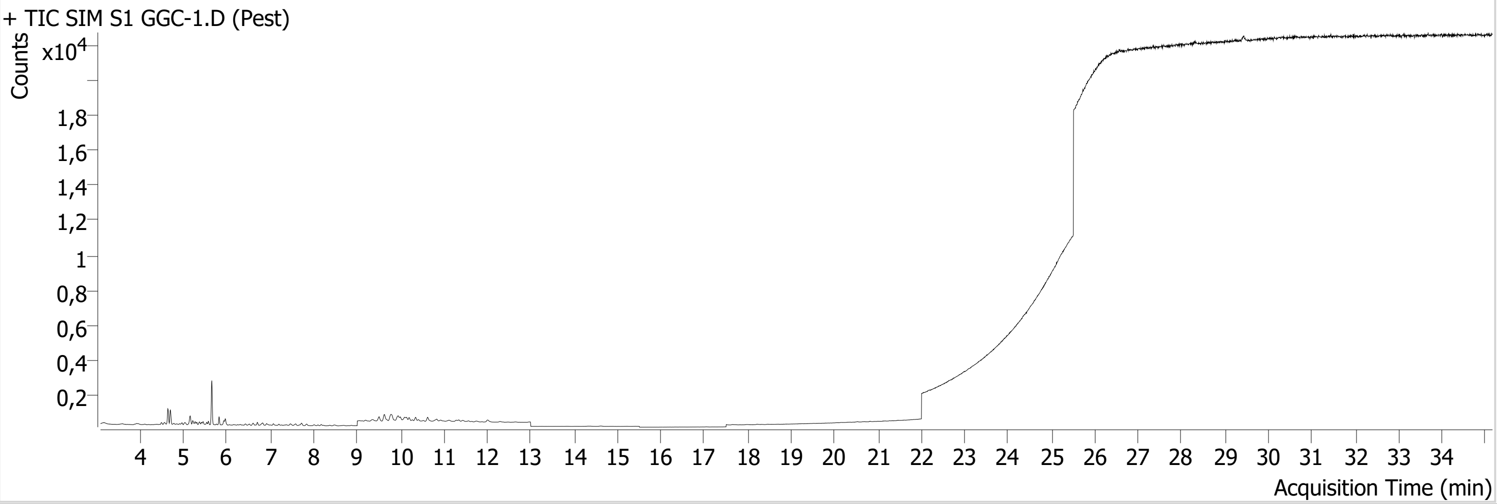

Compound	Transition	RT	Resp.	Final Conc	Units
Lindane	219,0	5,153	0	ND	ng/ml
Hexachlorobenzene	284,0	6,970	0	ND	ng/ml
Aldrine	293,0	11,104	0	ND	ng/ml
Chlorpyrifos	314,0	11,135	0	ND	ng/ml
Endosulfan II	339,0	14,608	74	69,9216	ng/ml
4,4-DDE	318,0	17,001	0	ND	ng/ml
4,4-DDD	237,0	18,221	14	4,0023	ng/ml
4,4-DDT	237,0	20,166	0	ND	ng/ml

Lindane

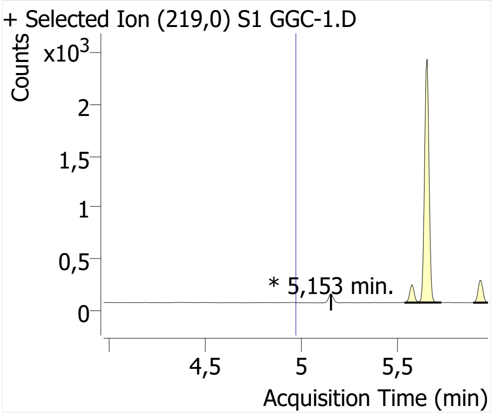

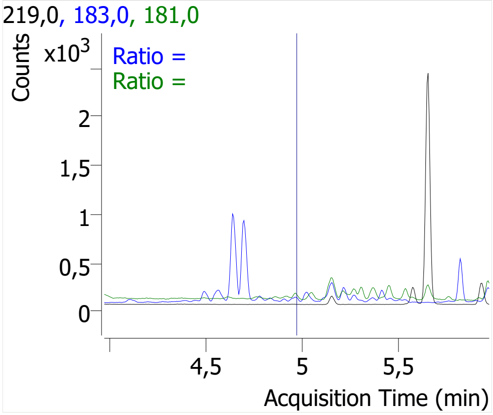

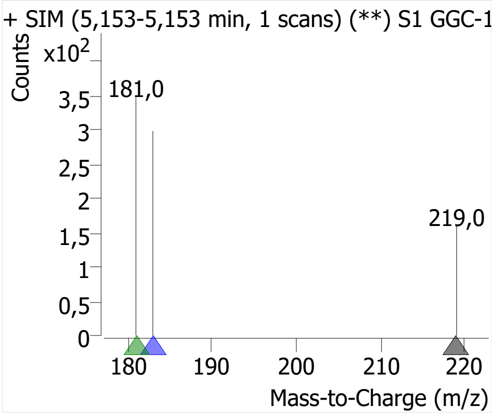

Hexachlorobenzene

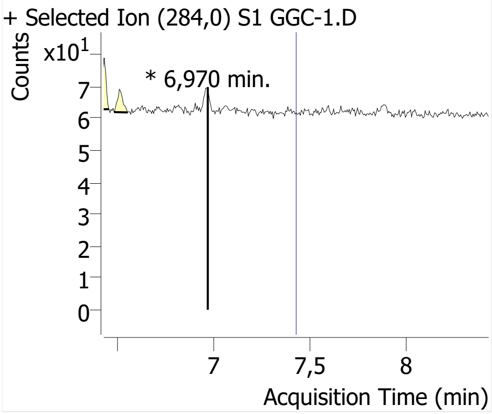

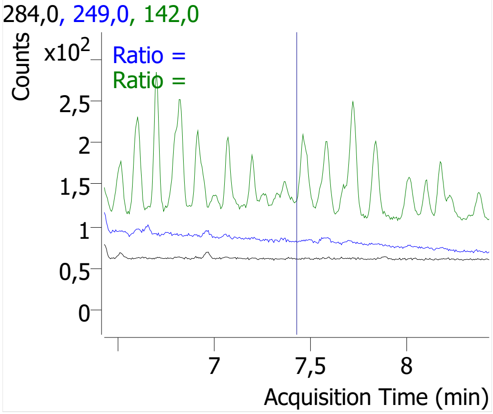

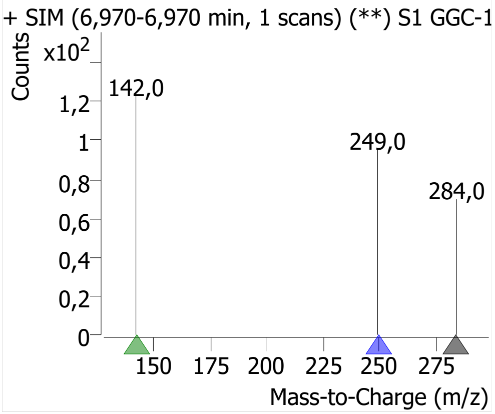

Aldrine

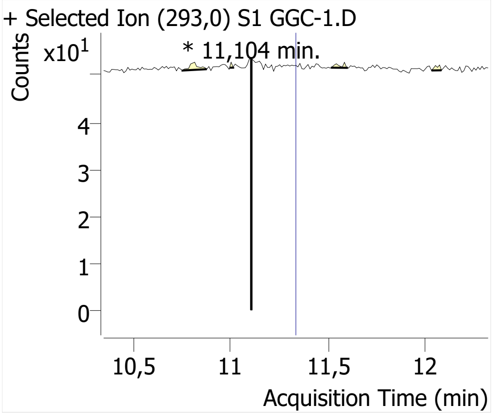

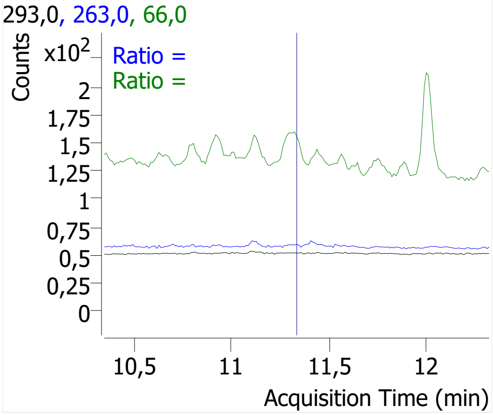

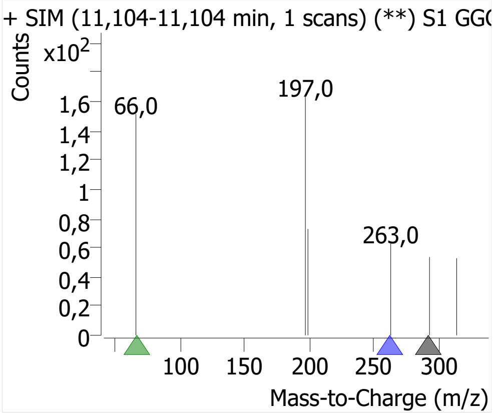

Chlorpyrifos

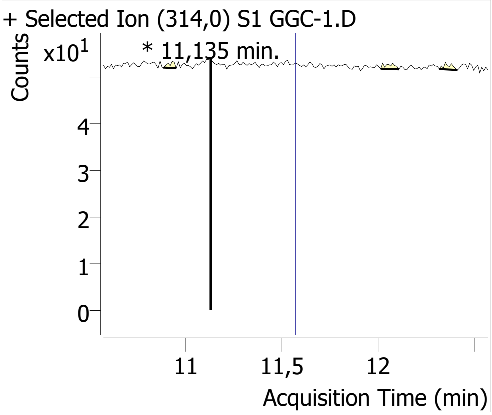

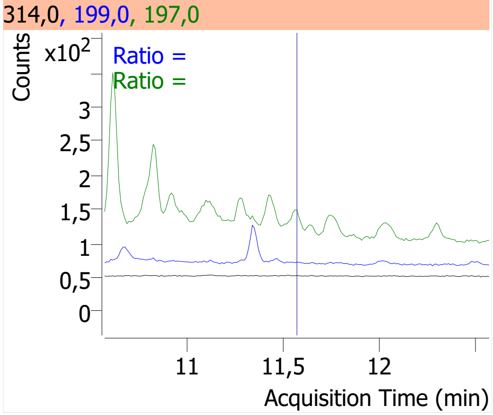

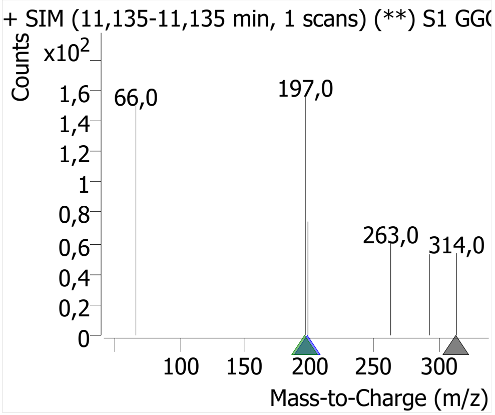

Endosulfan II

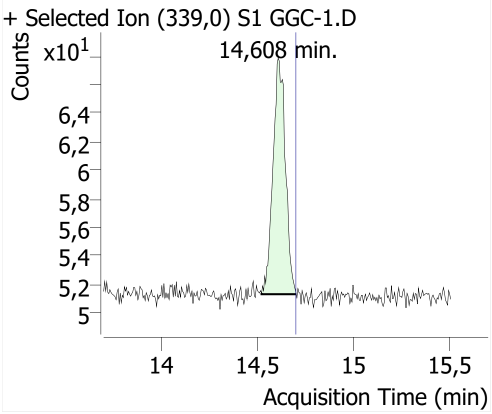

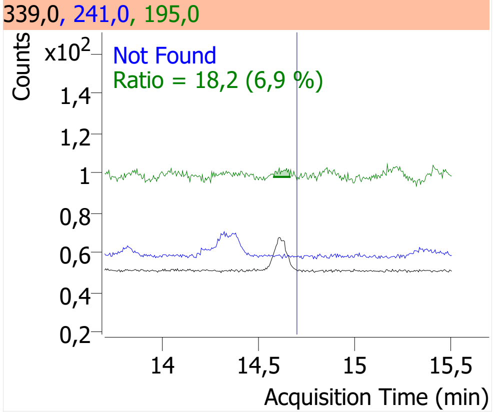

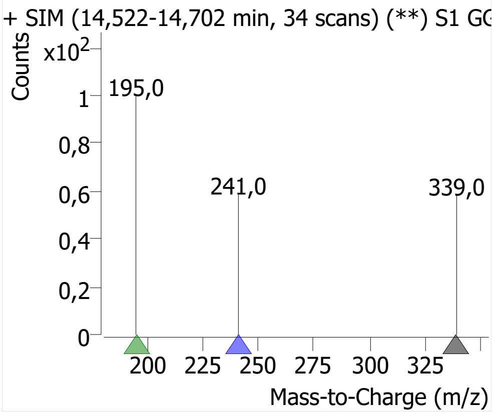

4,4-DDE

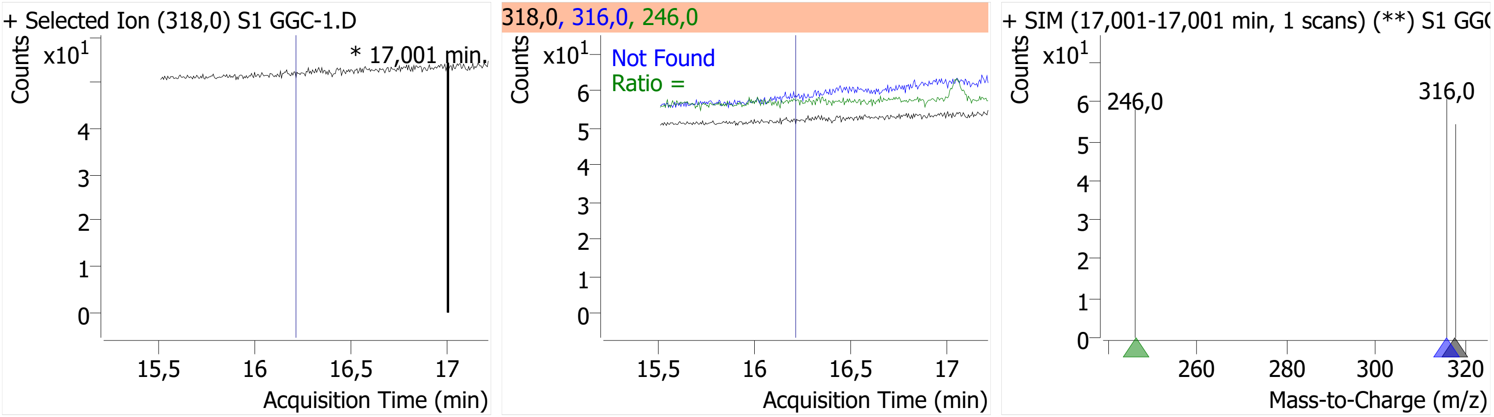

4,4-DDD

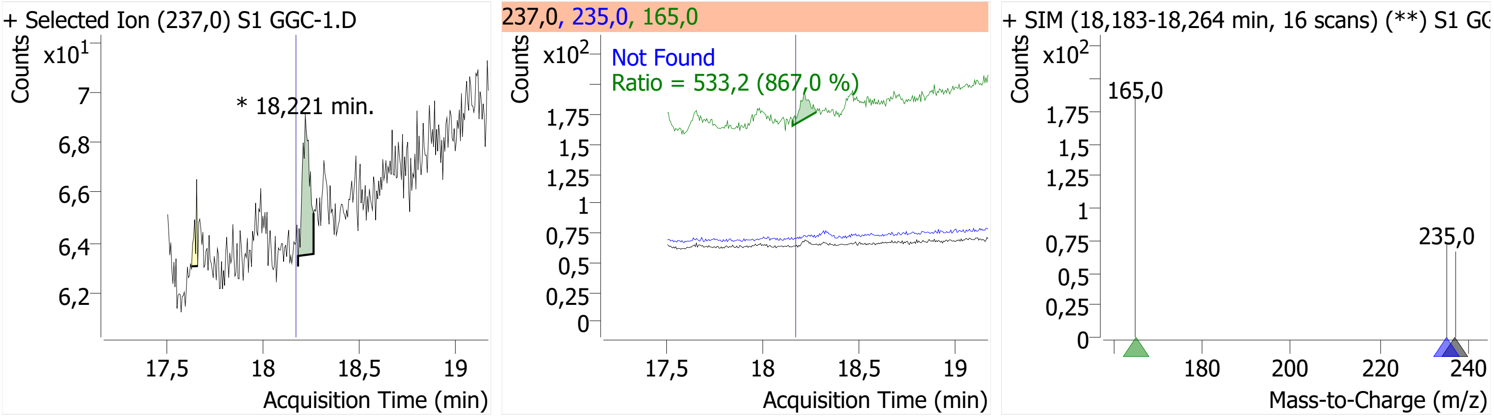

4,4-DDT

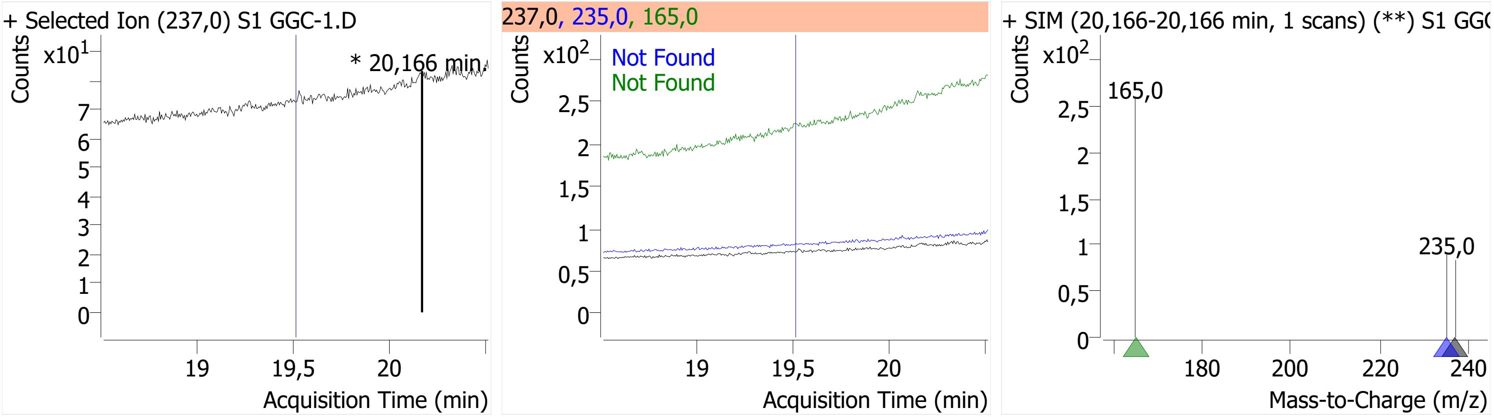

Quantitative Analysis Complete Report

**Batch Path**	C:\Users\USER\OneDrive\Desktop\JU_Pesticide\MA\MA\QuantResults\Mohammed_Pest.batch.bin		
**Analysis Time**	13 Dec 2024 11:34	**Analyst Name**	DESKTOP-MRRPPC4\USER
**Report Time**	13 Dec 2024 11:37:48	**Reporter Name**	DESKTOP-MRRPPC4\USER
**Last Calib Update**	13 Dec 2024 11:34	**Batch State**	Processed
**Quant Batch Version**	10.0	**Quant Report Version**	10.0

**Acq. Time**	13 Nov 2024 12:57	**Data File**	5 PPb.D
**Sample Type**	Cal	**Sample Name**	Pest
**Dilution**	1	**Acq. Method**	pesticide std 12.11.2024

Sample Chromatogram

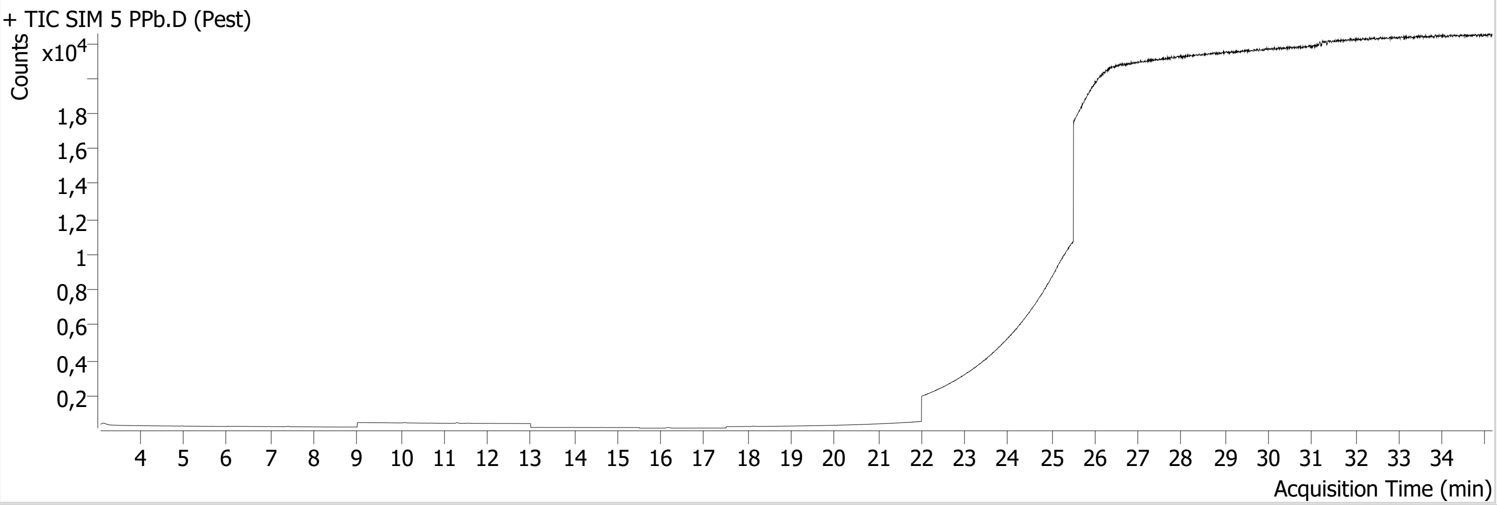

Compound	Transition	RT	Resp.	Final Conc	Units
Lindane	219,0	4,966	2	5,3511	ng/ml
Hexachlorobenzene	284,0	7,412	46	5,2431	ng/ml
Aldrine	293,0	11,302	18	6,0374	ng/ml
Chlorpyrifos	314,0	11,553	9	6,2445	ng/ml
Endosulfan II	339,0	14,609	10	3,8941	ng/ml
4,4-DDE	318,0	16,174	53	4,8457	ng/ml
4,4-DDD	237,0	18,129	23	5,6262	ng/ml
4,4-DDT	237,0	19,494	5	5,2082	ng/ml

Lindane

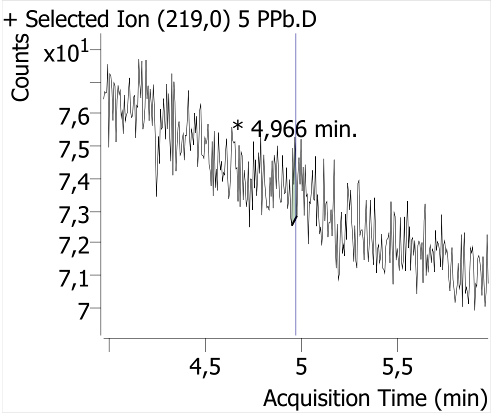

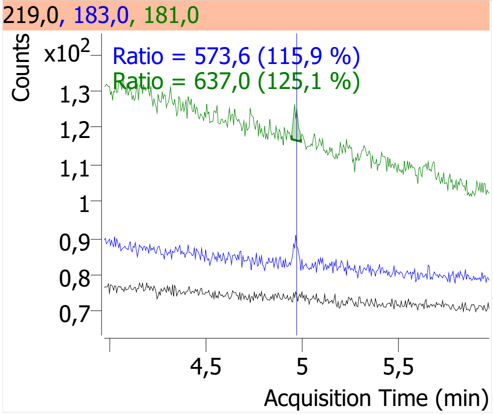

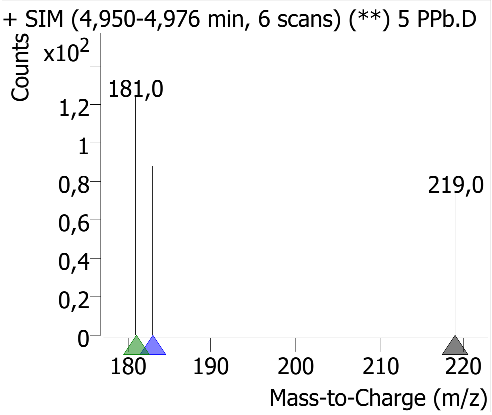

Hexachlorobenzene

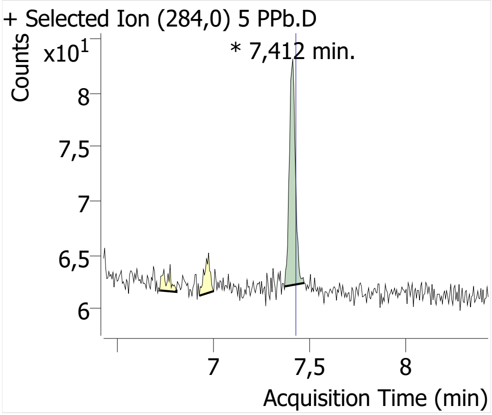

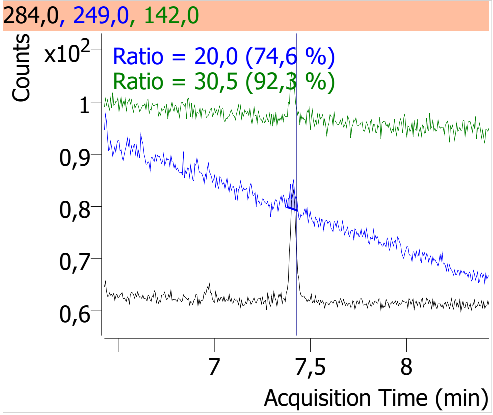

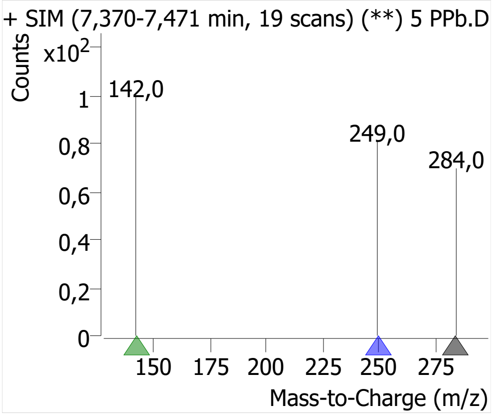

Aldrine

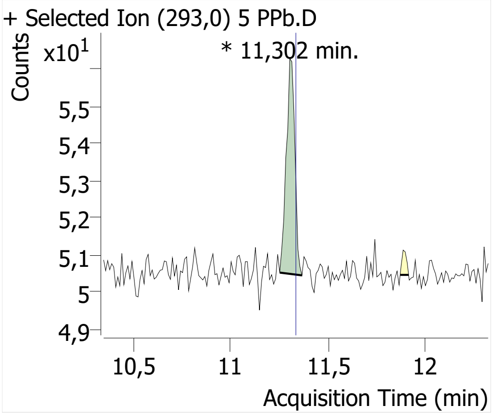

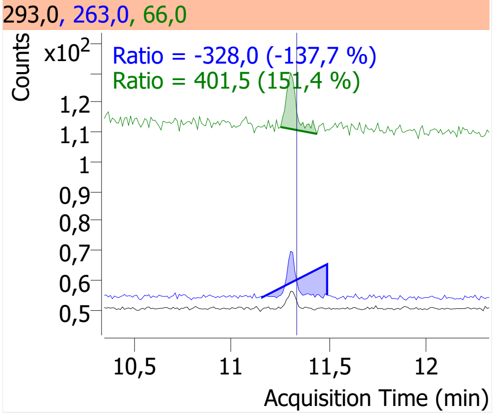

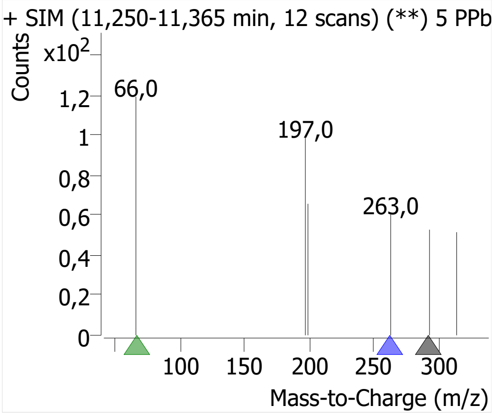

Chlorpyrifos

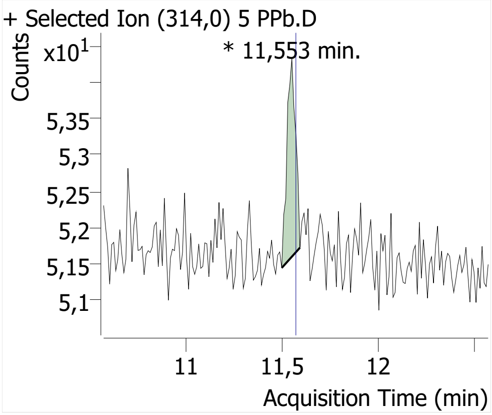

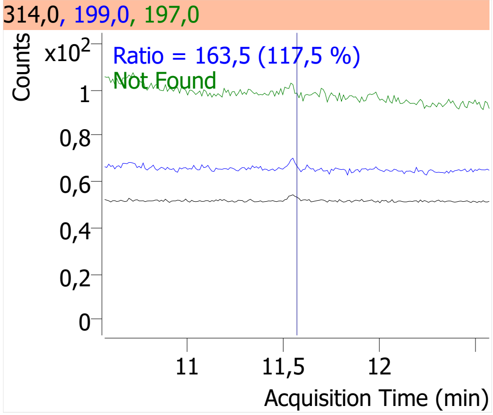

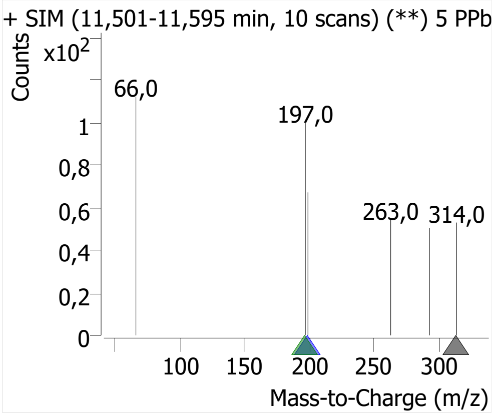

Endosulfan II

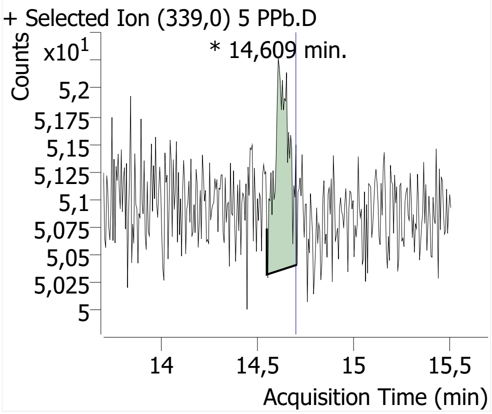

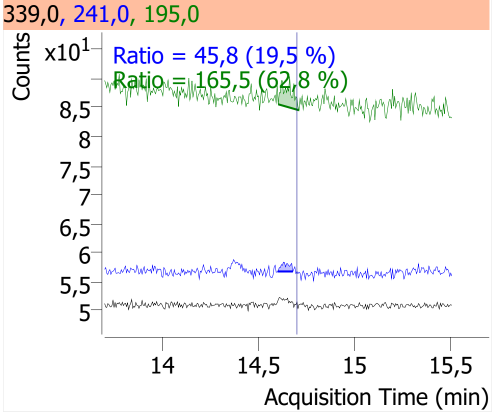

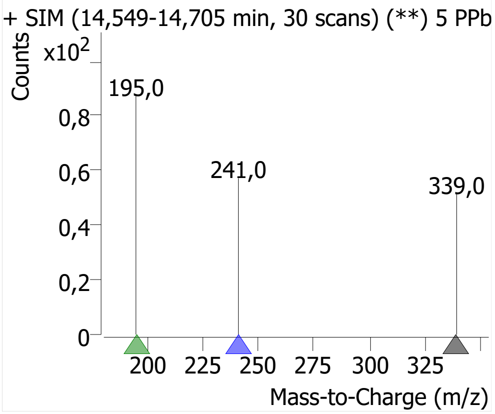

4,4-DDE

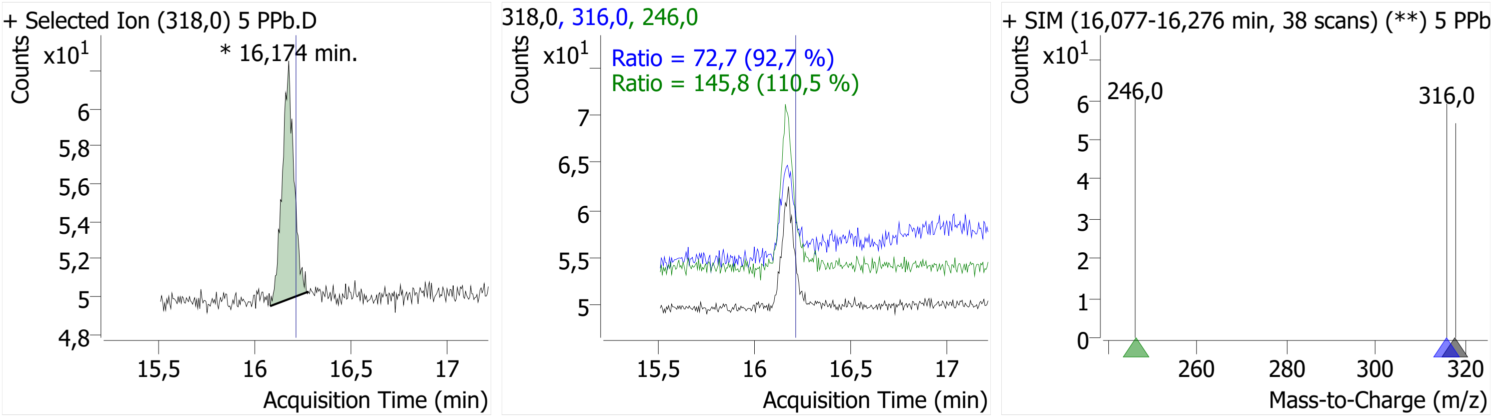

4,4-DDD

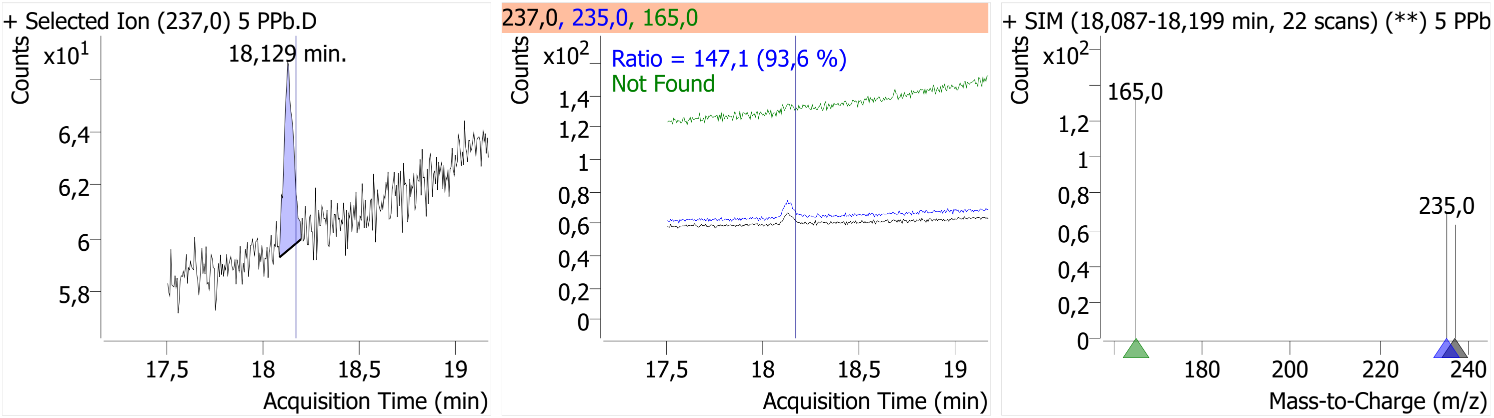

4,4-DDT

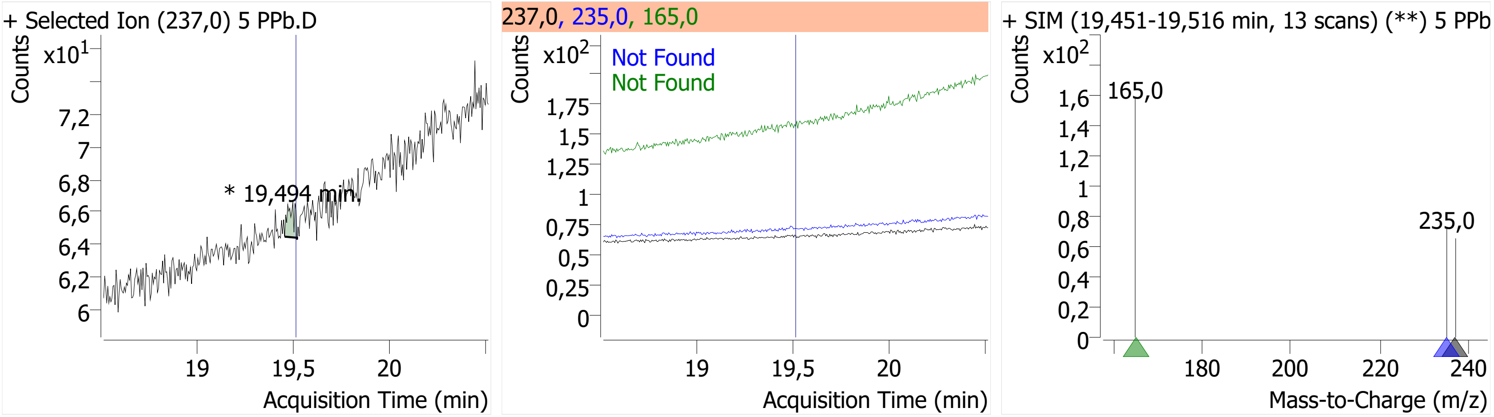

Quantitative Analysis Complete Report

**Batch Path**	C:\Users\USER\OneDrive\Desktop\JU_Pesticide\MA\MA\QuantResults\Mohammed_Pest.batch.bin		
**Analysis Time**	13 Dec 2024 11:34	**Analyst Name**	DESKTOP-MRRPPC4\USER
**Report Time**	13 Dec 2024 11:37:49	**Reporter Name**	DESKTOP-MRRPPC4\USER
**Last Calib Update**	13 Dec 2024 11:34	**Batch State**	Processed
**Quant Batch Version**	10.0	**Quant Report Version**	10.0

**Acq. Time**	13 Nov 2024 13:37	**Data File**	S1 DDO S.D
**Sample Type**	Sample	**Sample Name**	Pest
**Dilution**	1	**Acq. Method**	pesticide std 12.11.2024

Sample Chromatogram

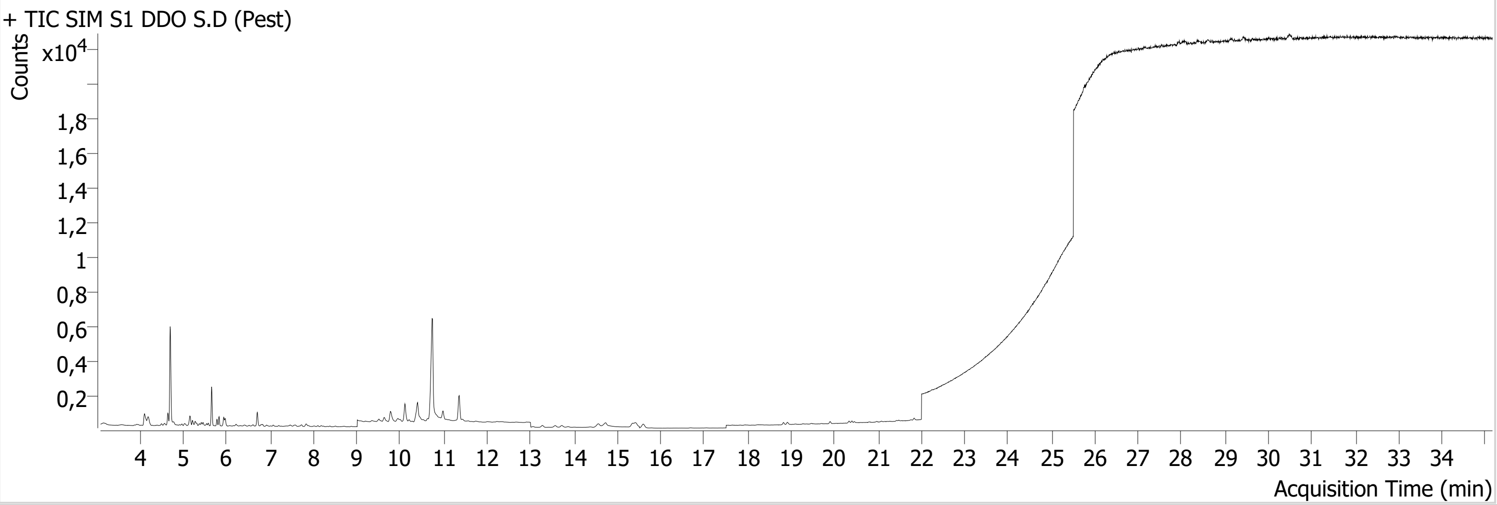

Compound	Transition	RT	Resp.	Final Conc	Units
Lindane	219,0	5,154	0	ND	ng/ml
Hexachlorobenzene	284,0	7,547	0	ND	ng/ml
Aldrine	293,0	10,739	0	ND	ng/ml
Chlorpyrifos	314,0	11,240	0	ND	ng/ml
Endosulfan II	339,0	14,732	0	ND	ng/ml
4,4-DDE	318,0	17,055	0	ND	ng/ml
4,4-DDD	237,0	18,232	36	8,0349	ng/ml
4,4-DDT	237,0	19,473	0	ND	ng/ml

Lindane

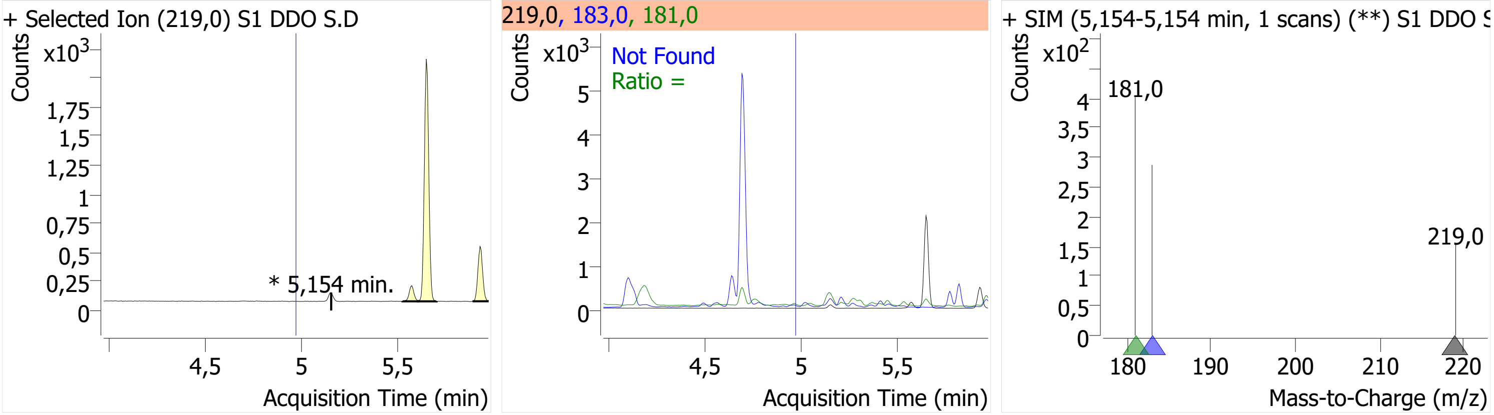

Hexachlorobenzene

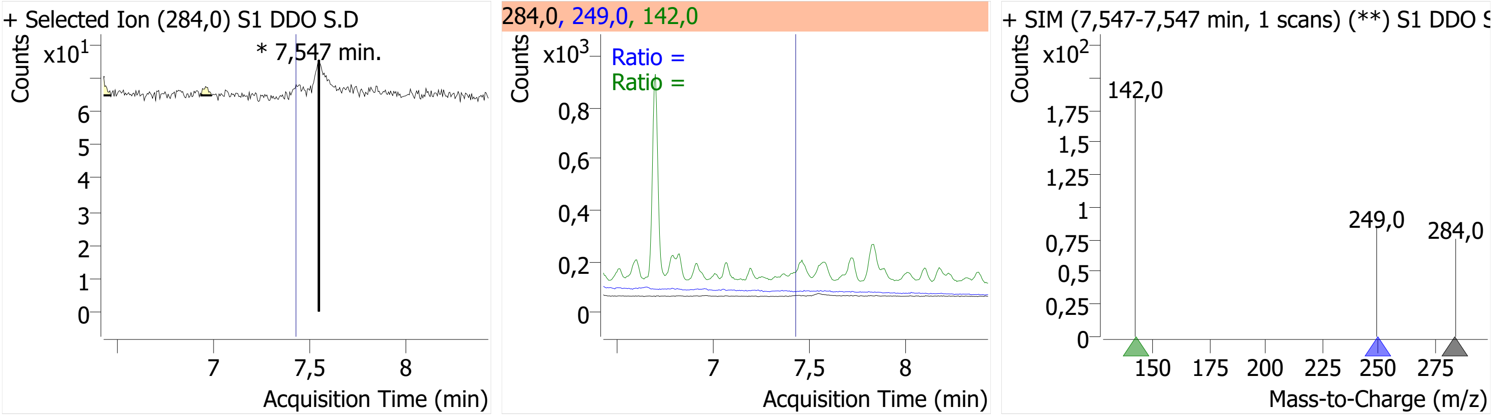

Aldrine

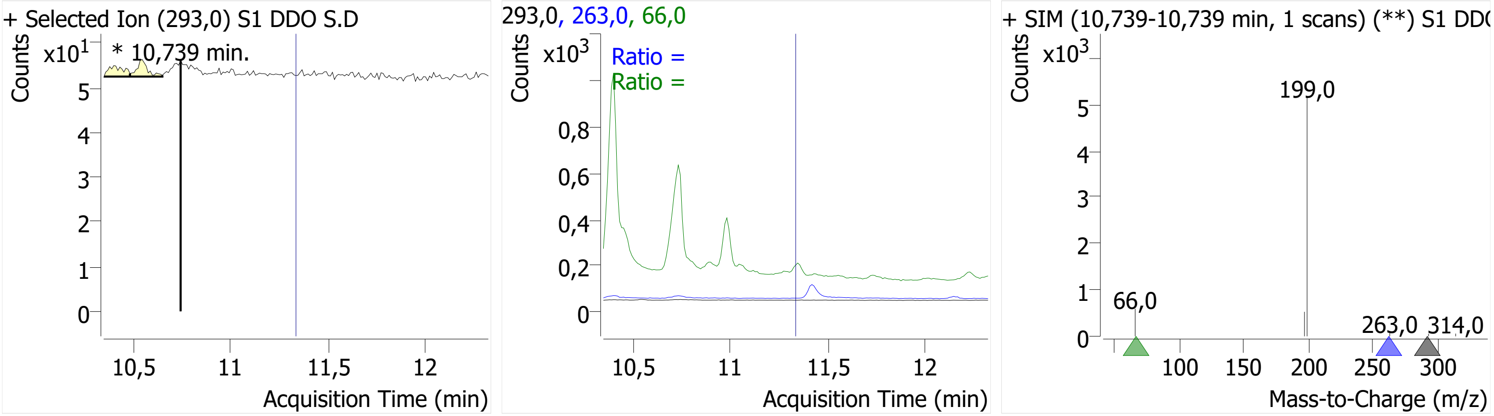

Chlorpyrifos

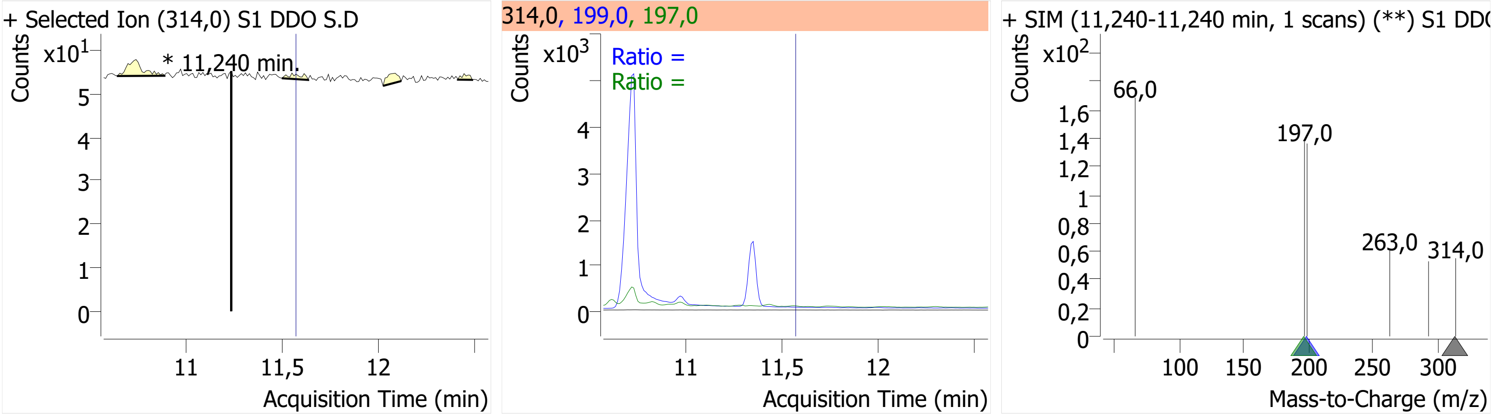

Endosulfan II

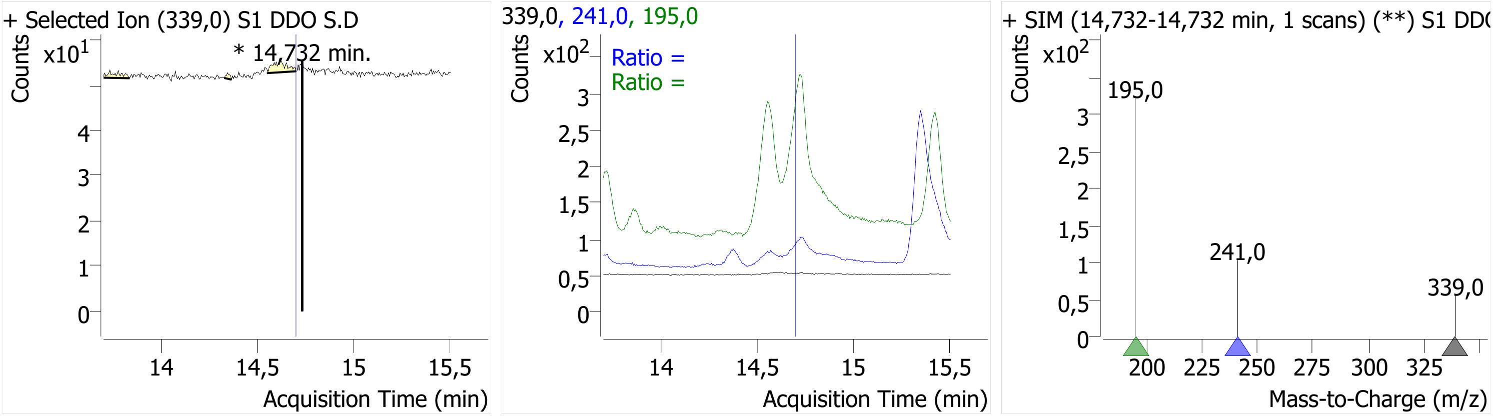

4,4-DDE

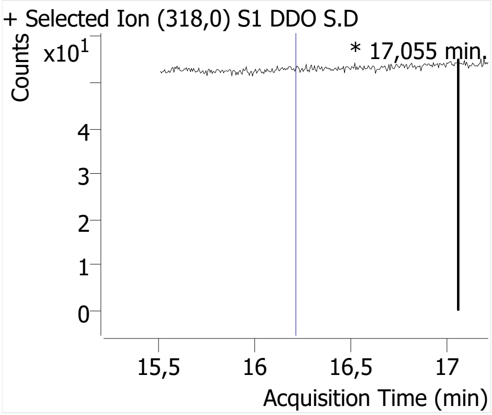

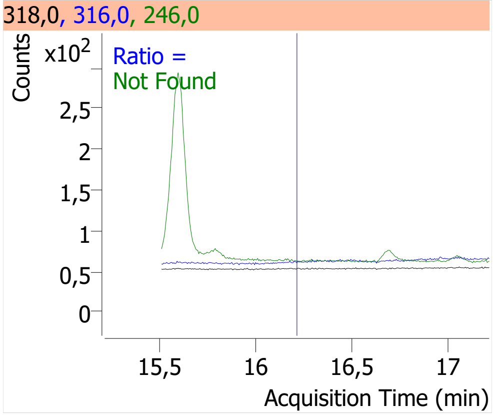

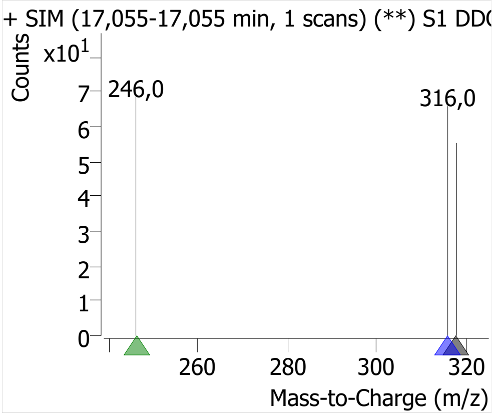

4,4-DDD

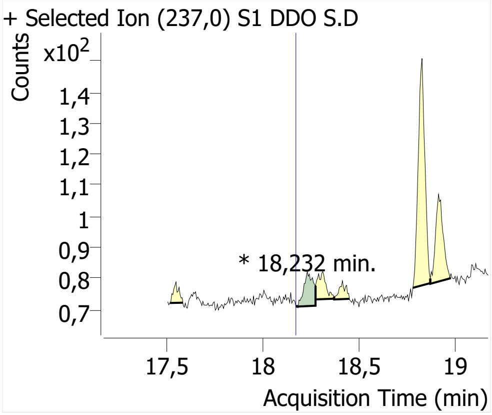

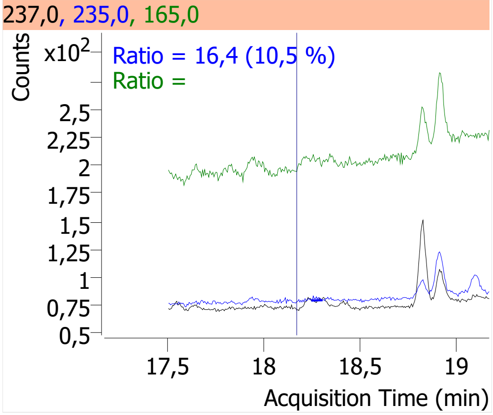

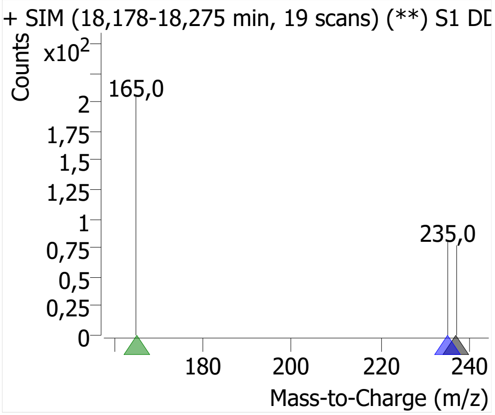

4,4-DDT

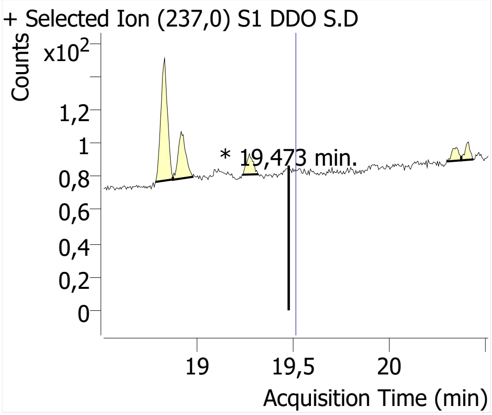

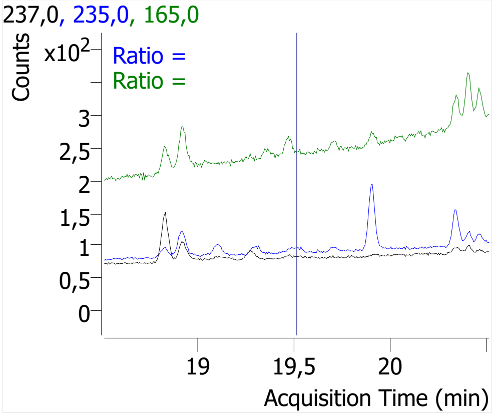

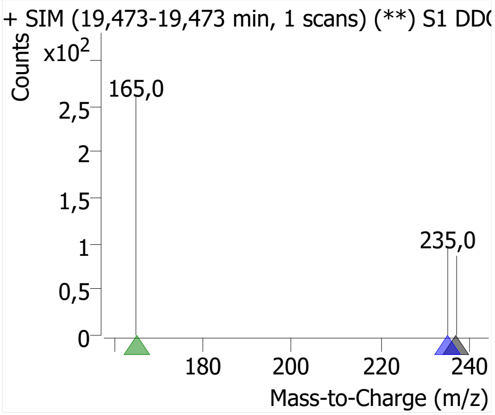

**Batch Path**	C:\Users\USER\OneDrive\Desktop\JU_Pesticide\MA\MA\QuantResults\Mohammed_Pest.batch.bin		
**Analysis Time**	13 Dec 2024 11:34	**Analyst Name**	DESKTOP-MRRPPC4\USER
**Report Time**	13 Dec 2024 11:37:50	**Reporter Name**	DESKTOP-MRRPPC4\USER
**Last Calib Update**	13 Dec 2024 11:34	**Batch State**	Processed
**Quant Batch Version**	10.0	**Quant Report Version**	10.0

**Acq. Time**	13 Nov 2024 16:28	**Data File**	S1 DOC S1.D
**Sample Type**	Sample	**Sample Name**	Pest
**Dilution**	1	**Acq. Method**	pesticide std 12.11.2024

Sample Chromatogram

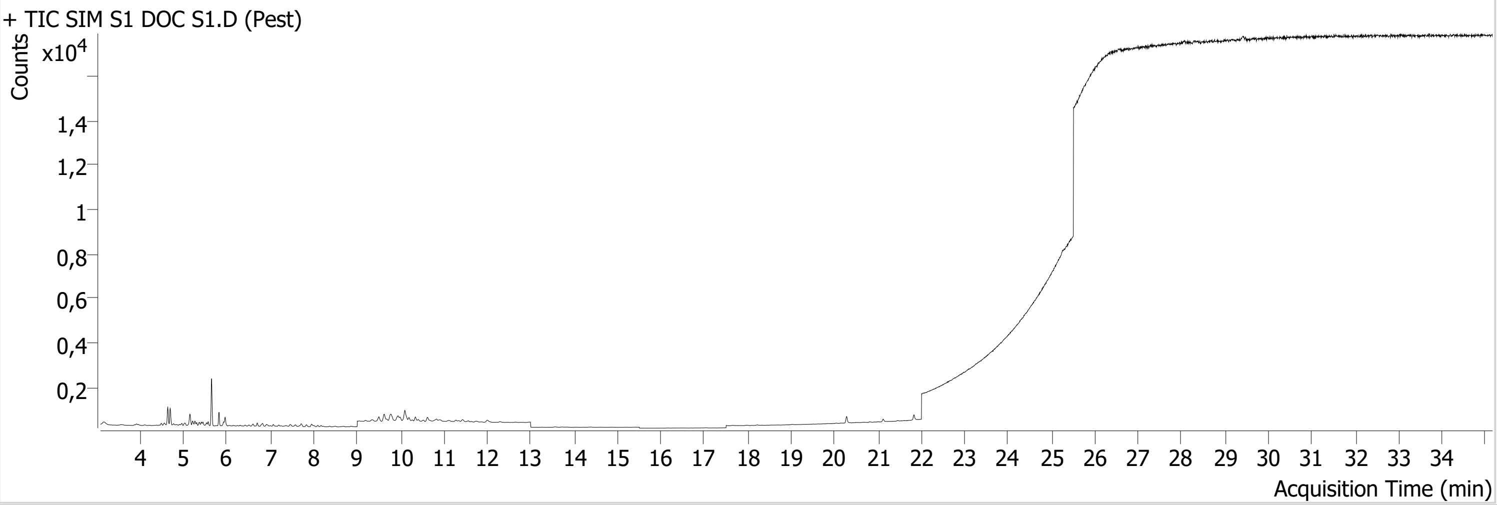

Compound	Transition	RT	Resp.	Final Conc	Units
Lindane	219,0	5,148	0	ND	ng/ml
Hexachlorobenzene	284,0	7,493	0	ND	ng/ml
Aldrine	293,0	11,261	0	ND	ng/ml
Chlorpyrifos	314,0	11,542	0	ND	ng/ml
Endosulfan II	339,0	14,608	14	8,4452	ng/ml
4,4-DDE	318,0	16,937	0	ND	ng/ml
4,4-DDD	237,0	18,221	20	4,9808	ng/ml
4,4-DDT	237,0	19,849	0	ND	ng/ml

Lindane

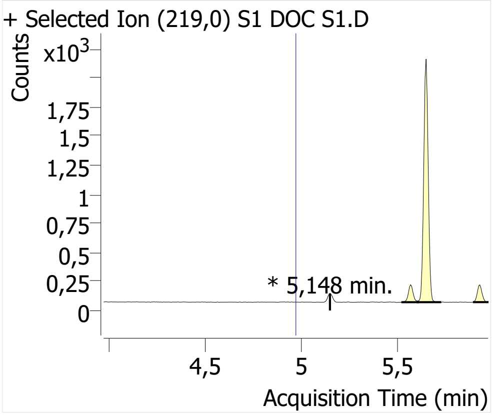

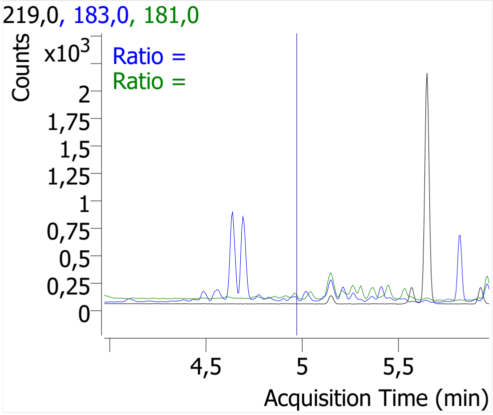

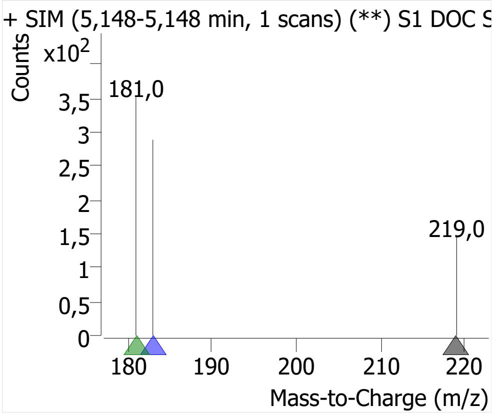

Hexachlorobenzene

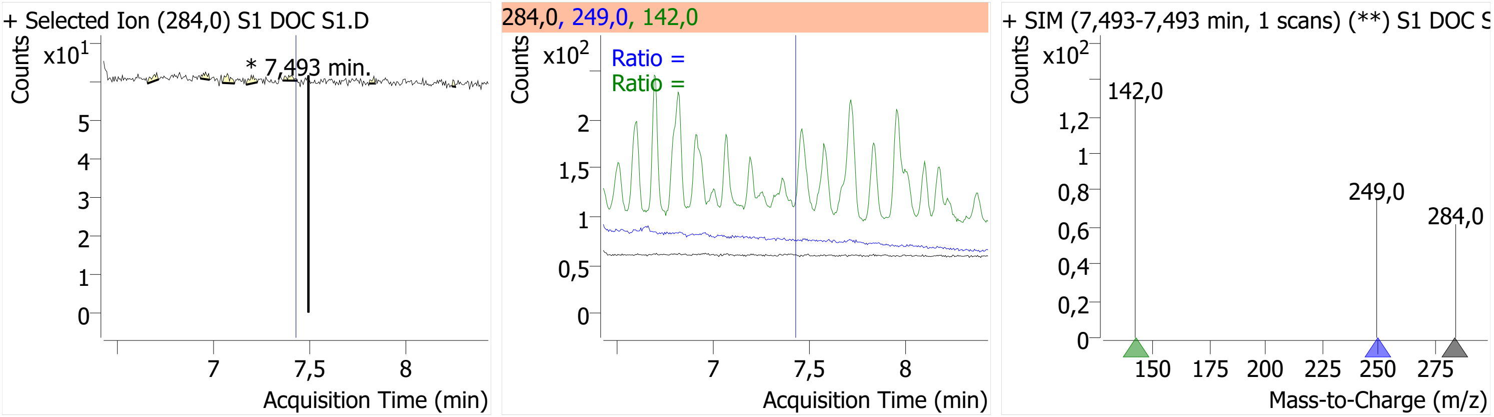

Aldrine

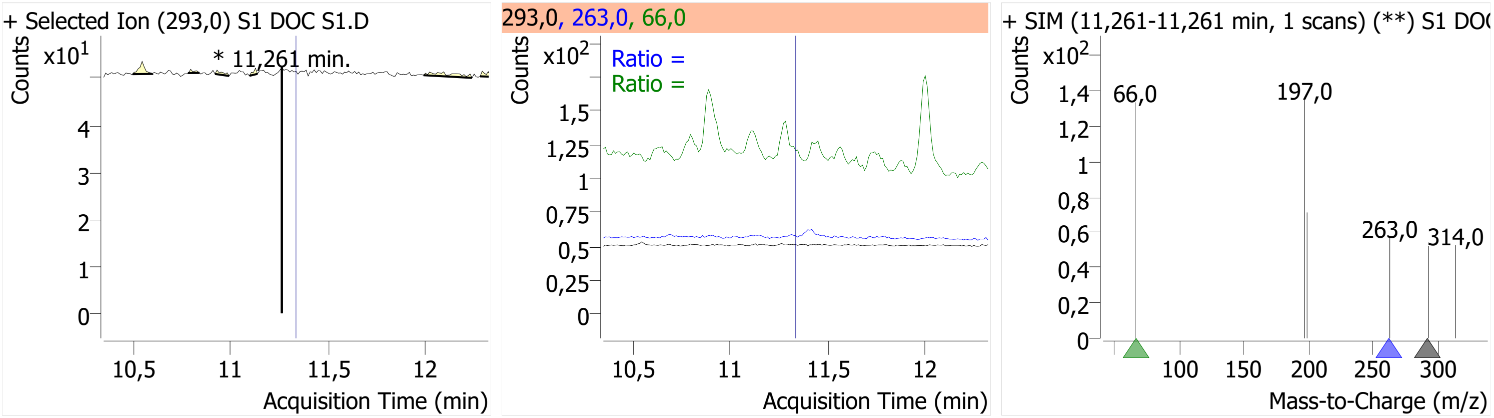

Chlorpyrifos

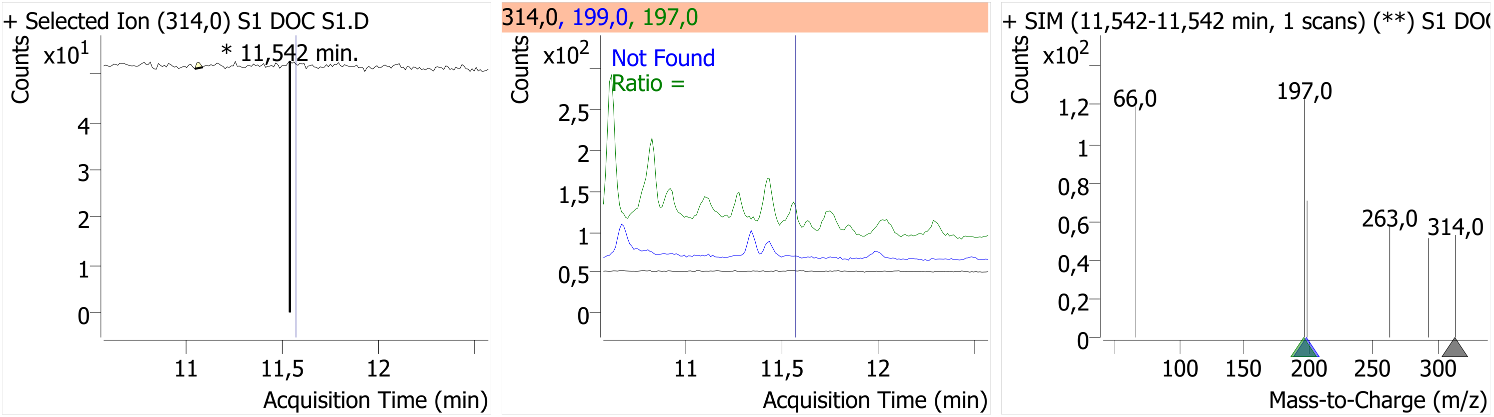

Endosulfan II

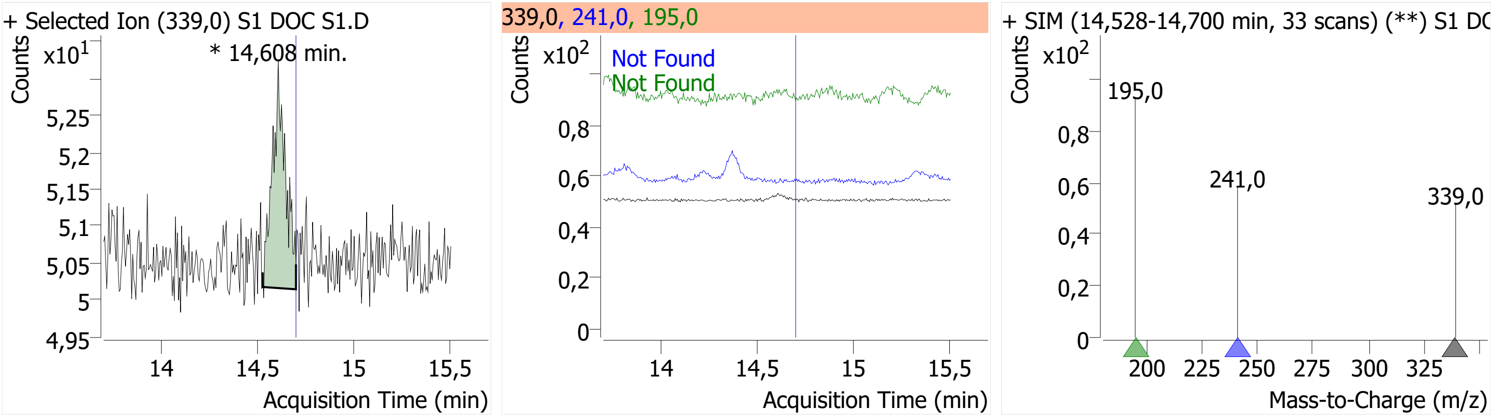

Agilent Trusted Answers

4,4-DDE

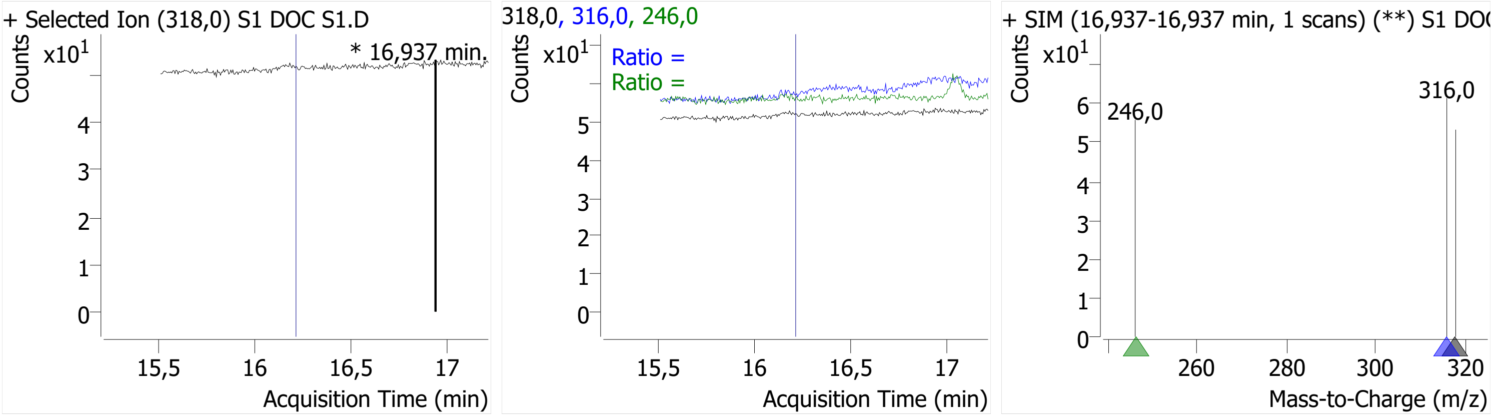

4,4-DDD

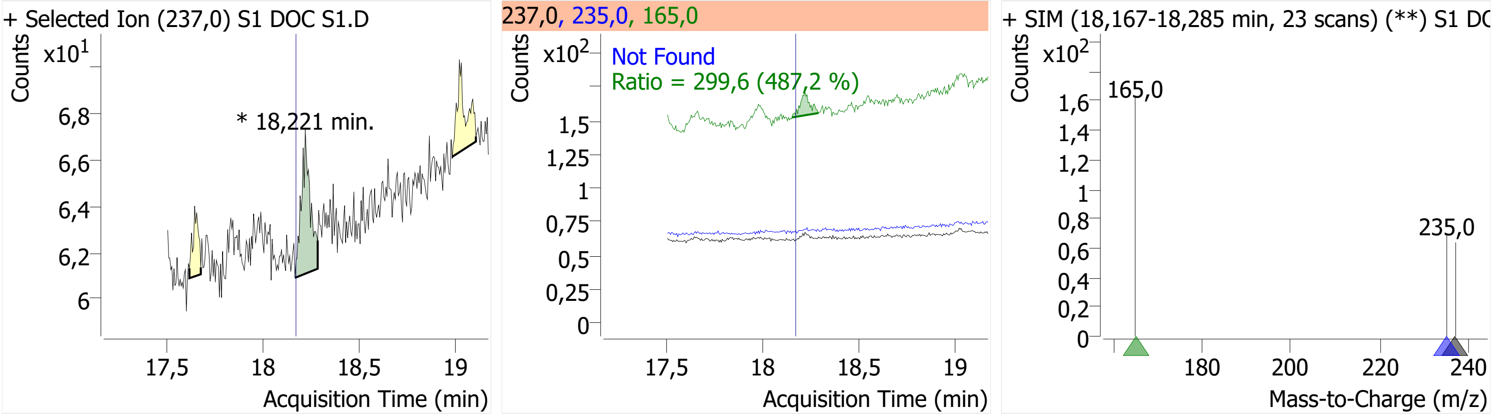

4,4-DDT

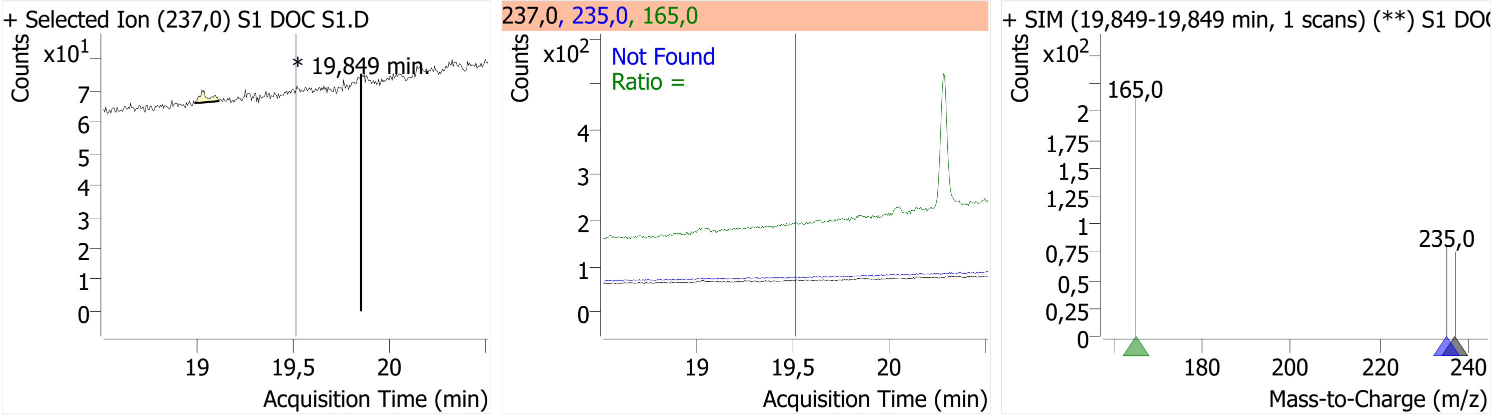

Quantitative Analysis Complete Report

**Batch Path**	C:\Users\USER\OneDrive\Desktop\JU_Pesticide\MA\MA\QuantResults\Mohammed_Pest.batch.bin		
**Analysis Time**	13 Dec 2024 11:34	**Analyst Name**	DESKTOP-MRRPPC4\USER
**Report Time**	13 Dec 2024 11:37:51	**Reporter Name**	DESKTOP-MRRPPC4\USER
**Last Calib Update**	13 Dec 2024 11:34	**Batch State**	Processed
**Quant Batch Version**	10.0	**Quant Report Version**	10.0

**Acq. Time**	13 Nov 2024 17:09	**Data File**	S1 DDP S.D
**Sample Type**	Sample	**Sample Name**	Pest
**Dilution**	1	**Acq. Method**	pesticide std 12.11.2024

Sample Chromatogram

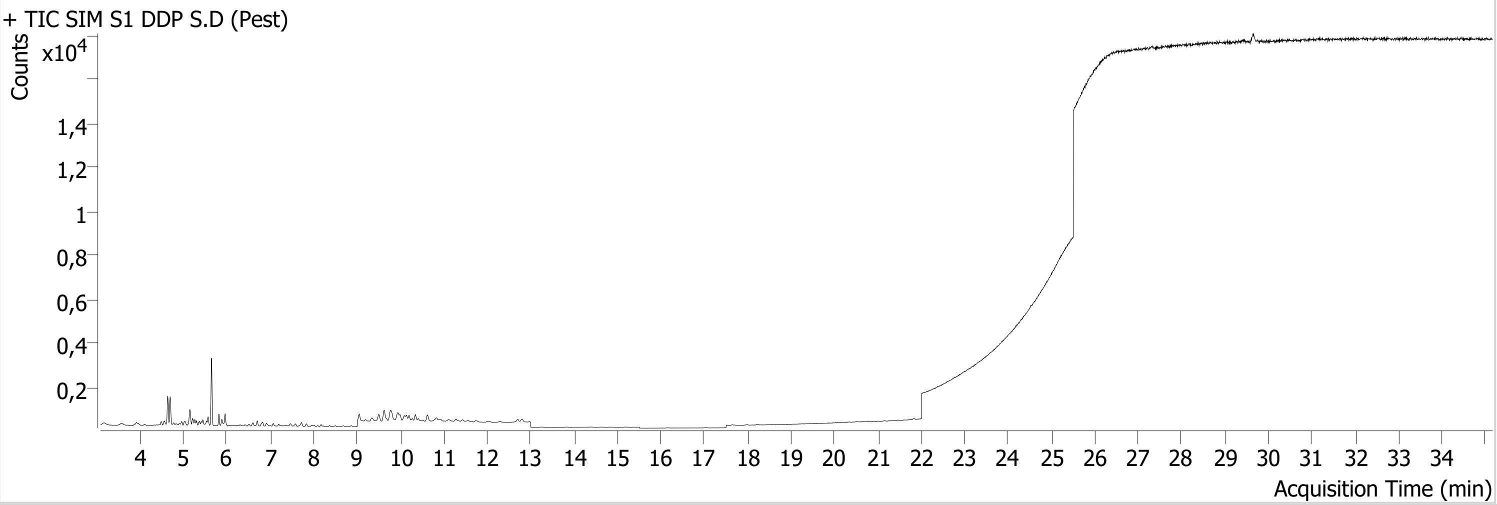

Compound	Transition	RT	Resp.	Final Conc	Units
Lindane	219,0	5,148	0	ND	ng/ml
Hexachlorobenzene	284,0	7,401	22	3,4423	ng/ml
Aldrine	293,0	11,323	9	4,3612	ng/ml
Chlorpyrifos	314,0	11,532	0	ND	ng/ml
Endosulfan II	339,0	14,614	0	ND	ng/ml
4,4-DDE	318,0	16,141	14	2,1735	ng/ml
4,4-DDD	237,0	18,226	24	5,8155	ng/ml
4,4-DDT	237,0			ND	ng/ml

Lindane

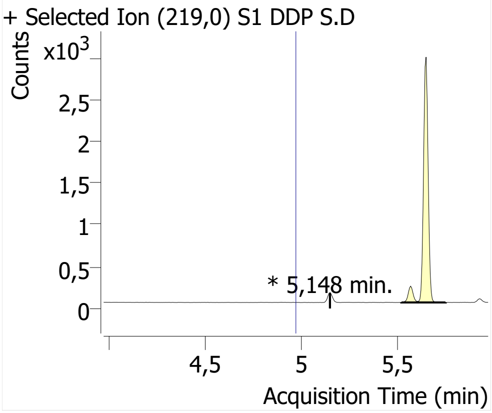

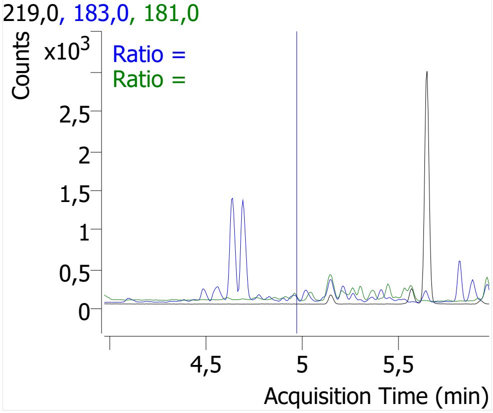

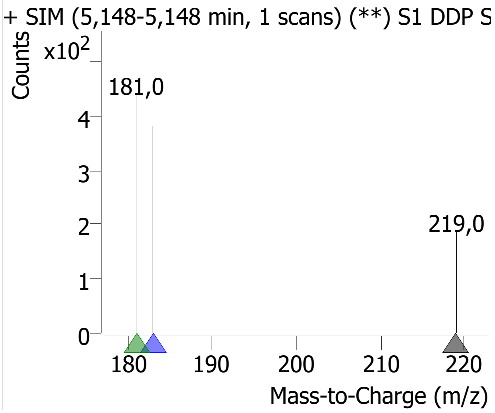

Agilent Trusted Answers

Hexachlorobenzene

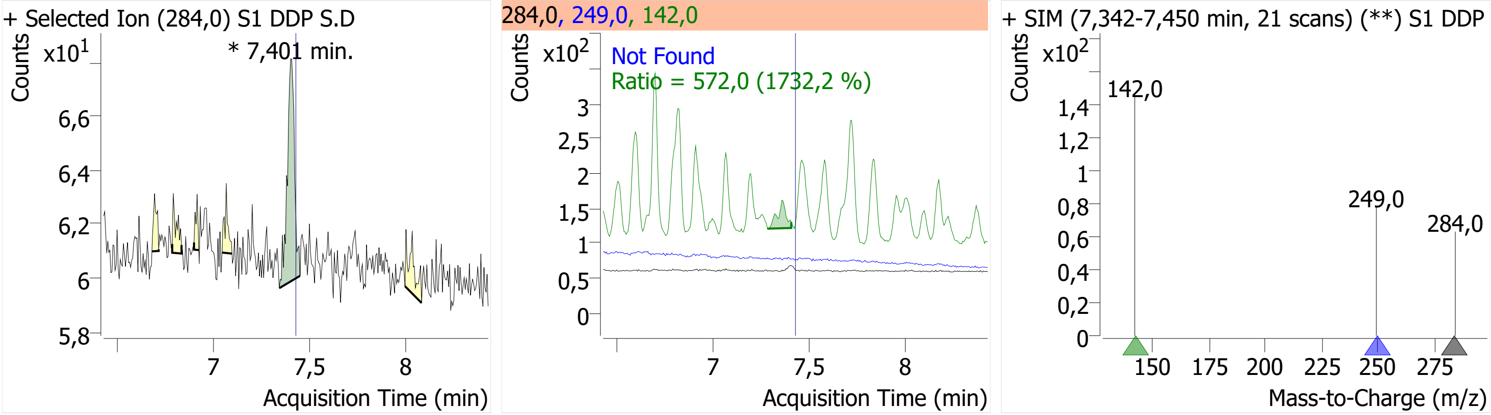

Aldrine

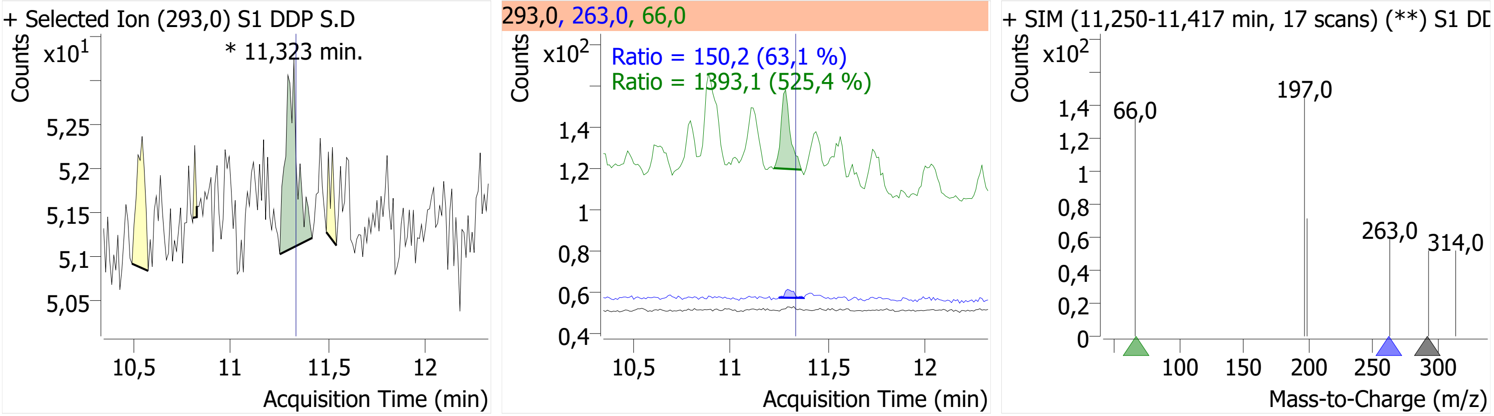

Chlorpyrifos

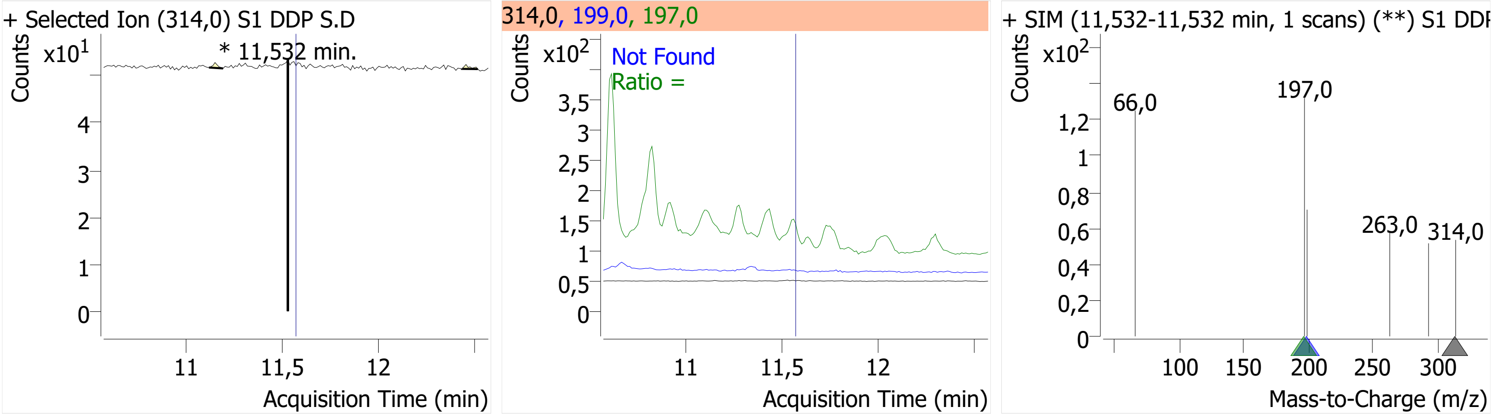

Endosulfan II

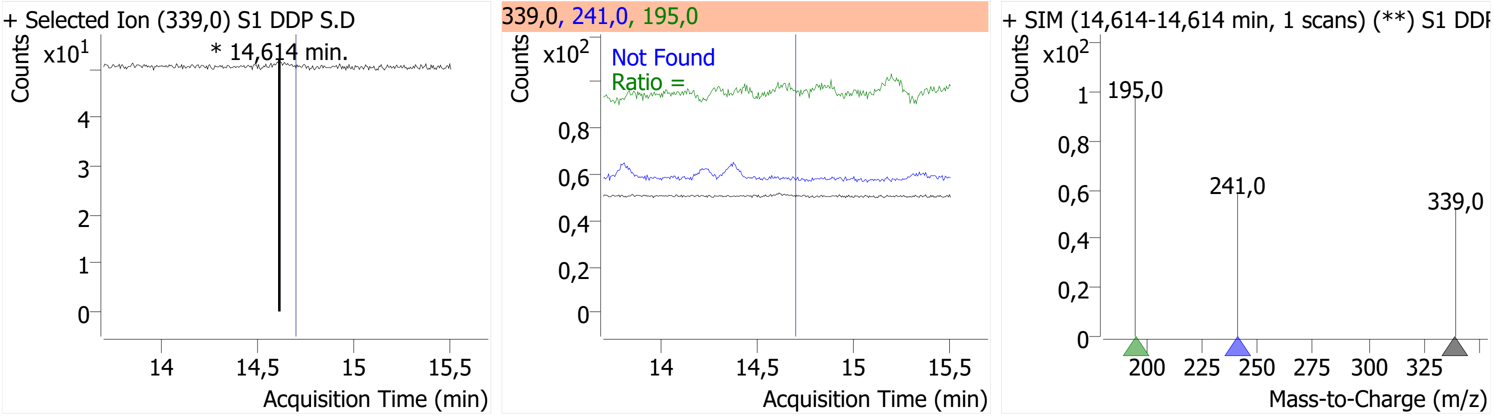

4,4-DDE

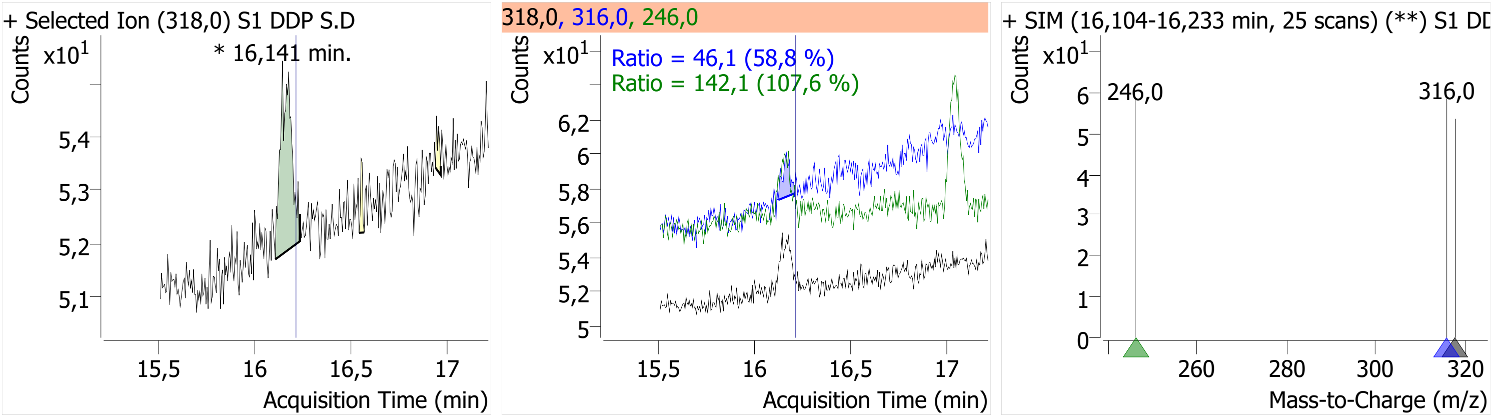

4,4-DDD

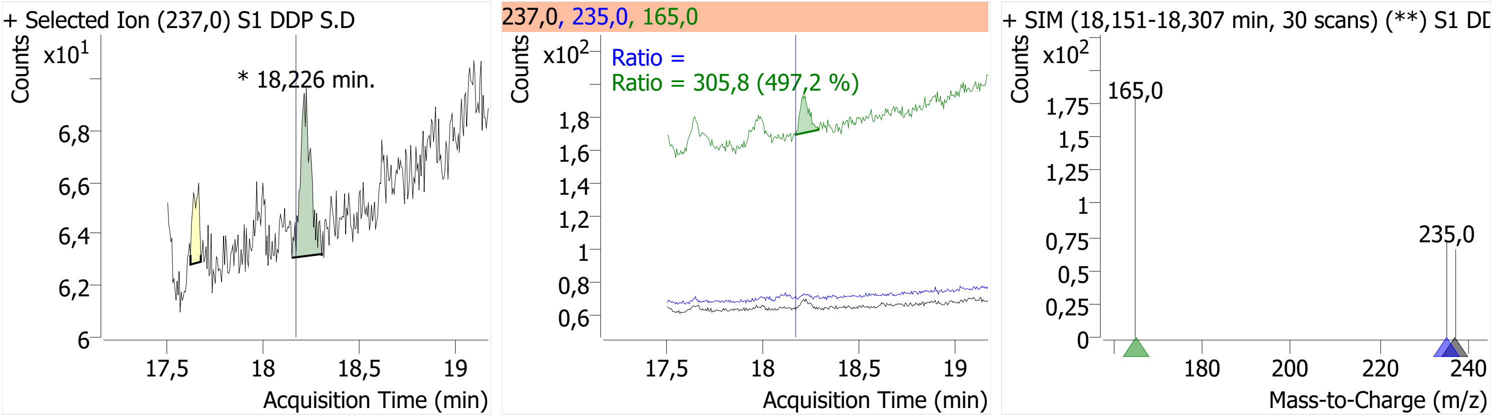

4,4-DDT

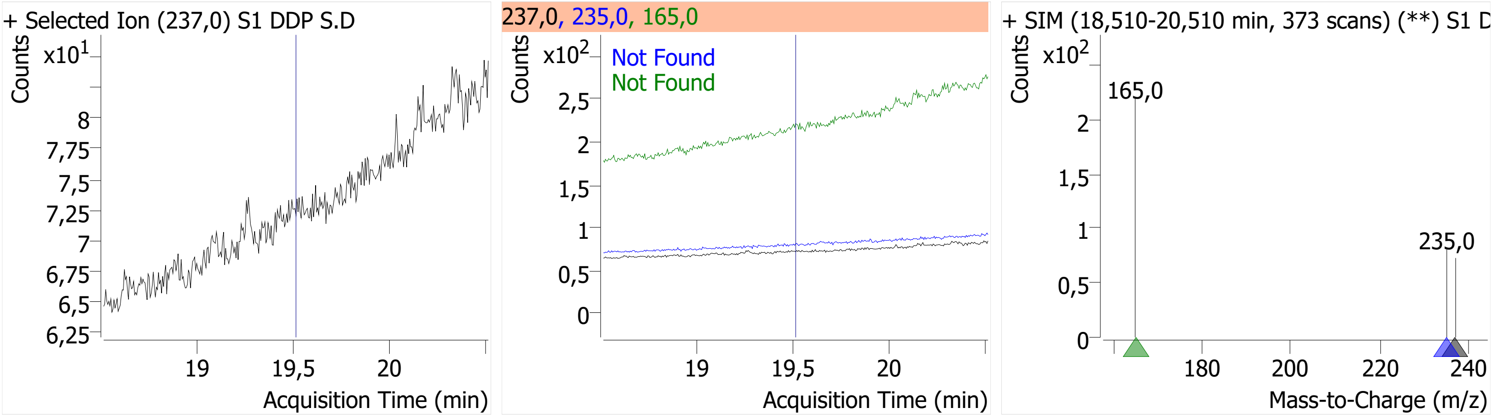

Quantitative Analysis Complete Report

**Batch Path**	C:\Users\USER\OneDrive\Desktop\JU_Pesticide\MA\MA\QuantResults\Mohammed_Pest.batch.bin		
**Analysis Time**	13 Dec 2024 11:34	**Analyst Name**	DESKTOP-MRRPPC4\USER
**Report Time**	13 Dec 2024 11:37:52	**Reporter Name**	DESKTOP-MRRPPC4\USER
**Last Calib Update**	13 Dec 2024 11:34	**Batch State**	Processed
**Quant Batch Version**	10.0	**Quant Report Version**	10.0

**Acq. Time**	13 Nov 2024 17:50	**Data File**	S1 DWT S.D
**Sample Type**	Sample	**Sample Name**	Pest
**Dilution**	1	**Acq. Method**	pesticide std 12.11.2024

Sample Chromatogram

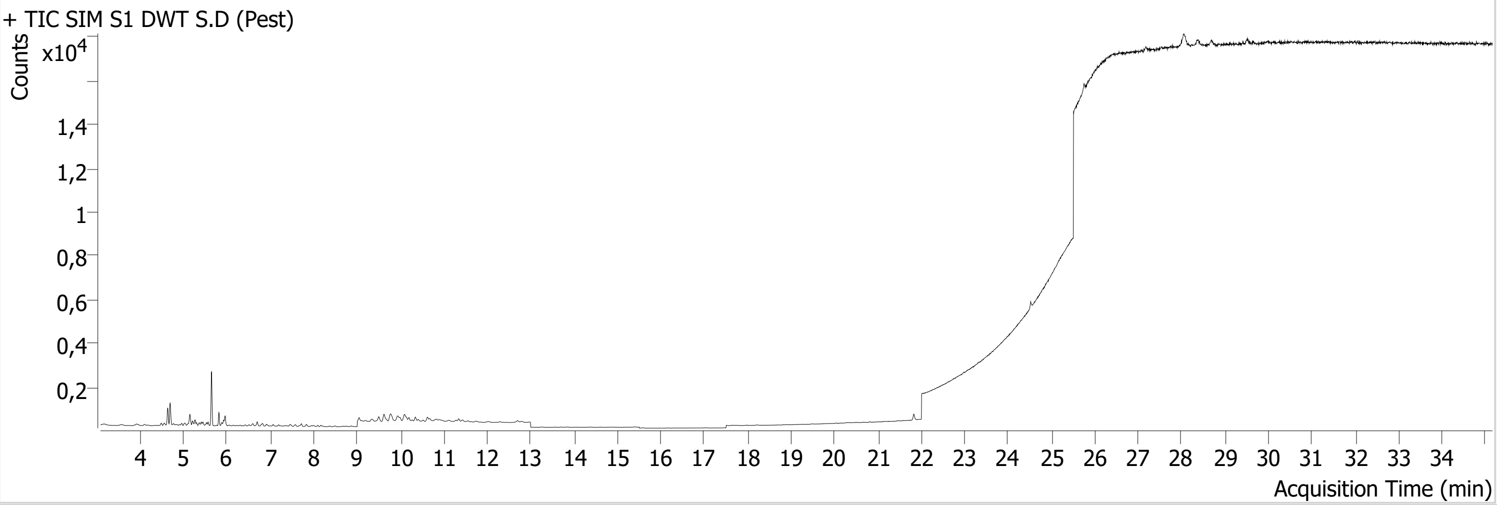

Compound	Transition	RT	Resp.	Final Conc	Units
Lindane	219,0	5,148	0	ND	ng/ml
Hexachlorobenzene	284,0	7,407	5	2,1630	ng/ml
Aldrine	293,0	11,292	0	ND	ng/ml
Chlorpyrifos	314,0	11,543	10	6,8732	ng/ml
Endosulfan II	339,0	14,609	18	11,8424	ng/ml
4,4-DDE	318,0	16,158	6	1,6467	ng/ml
4,4-DDD	237,0	18,210	16	4,3225	ng/ml
4,4-DDT	237,0	18,833	0	ND	ng/ml

Lindane

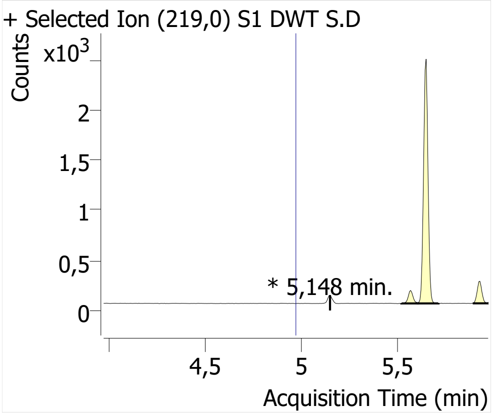

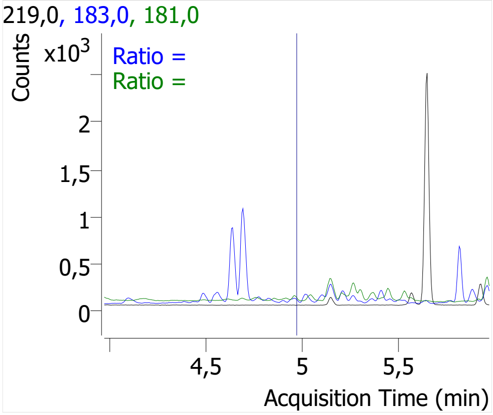

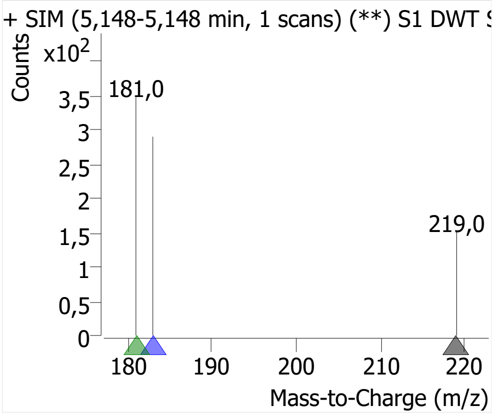

Hexachlorobenzene

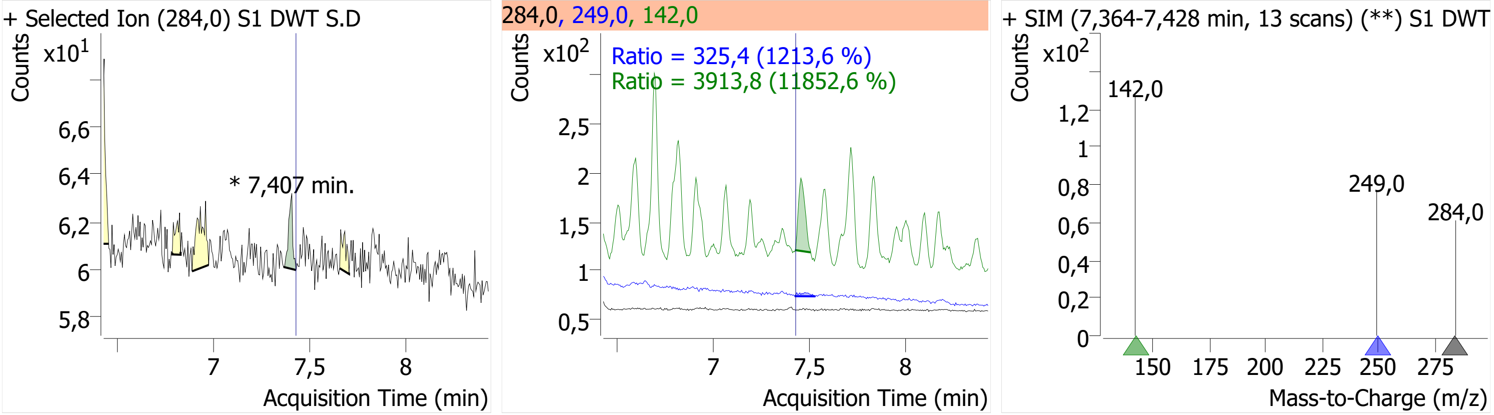

Aldrine

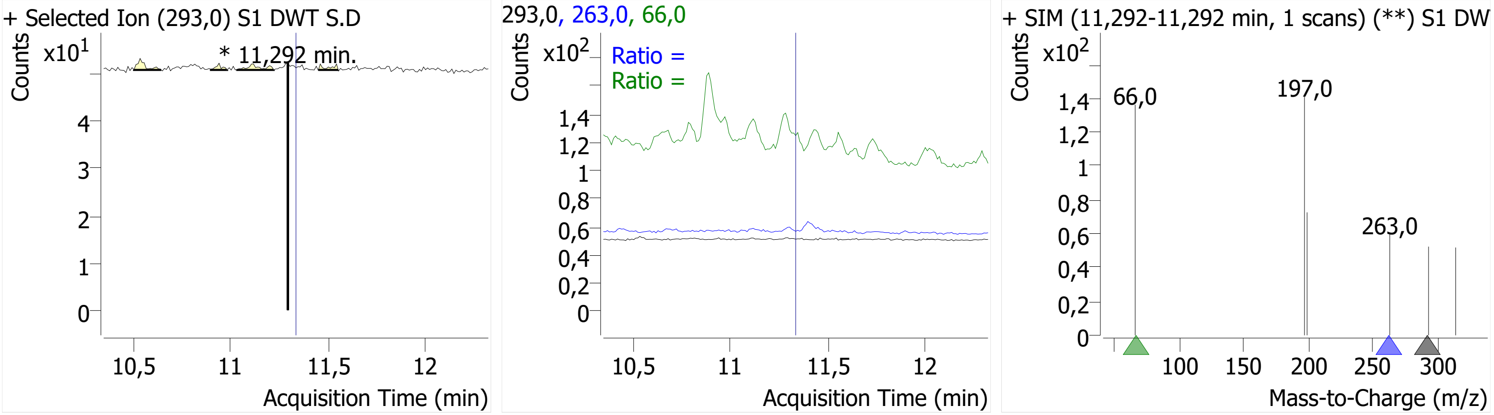

Chlorpyrifos

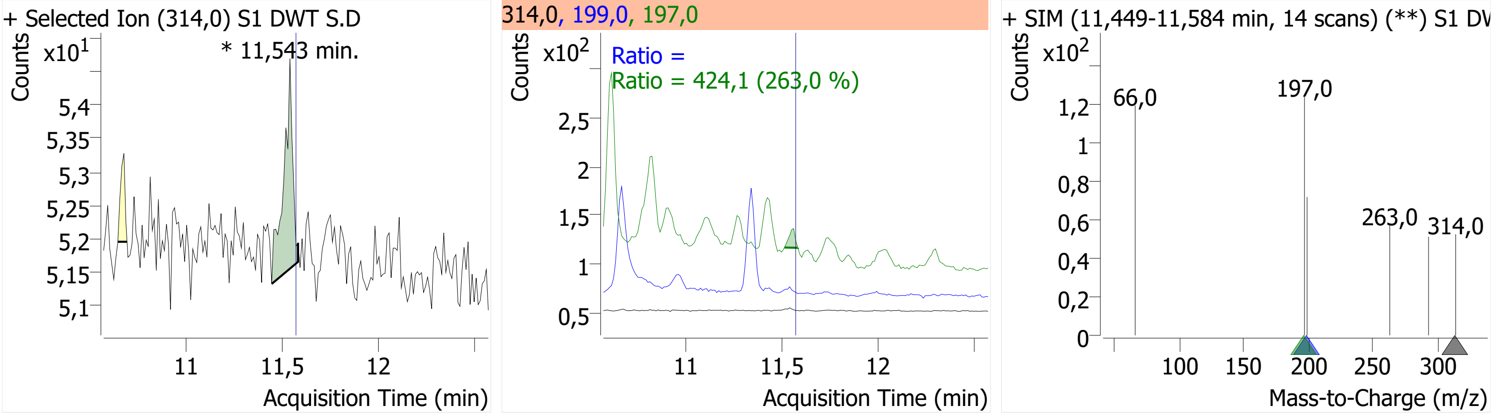

Endosulfan II

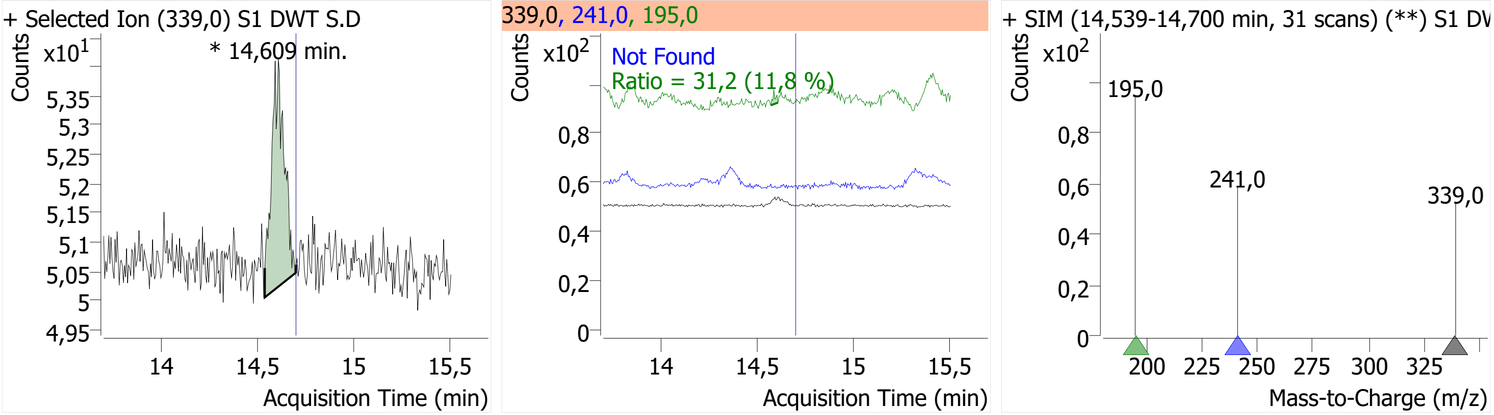

4,4-DDE

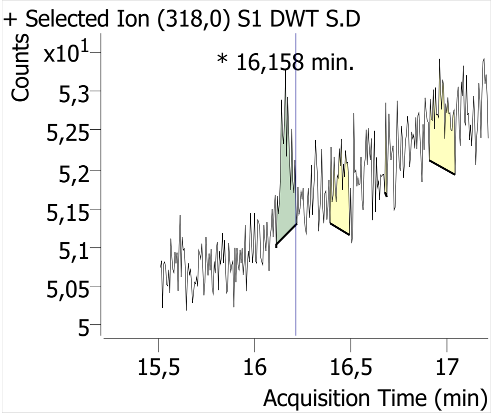

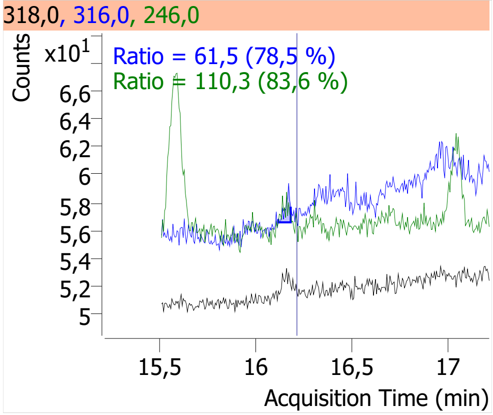

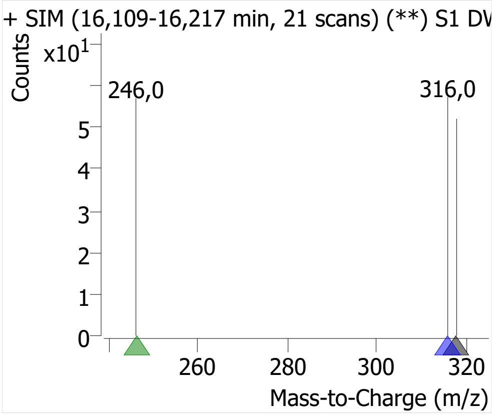

4,4-DDD

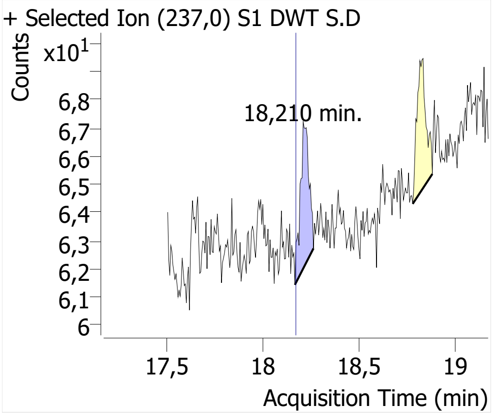

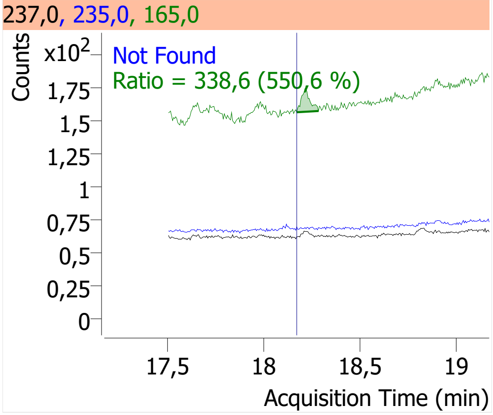

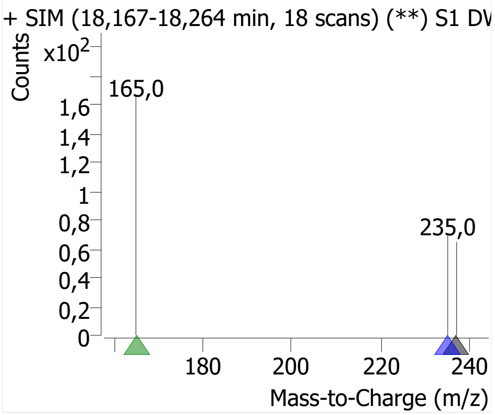

4,4-DDT

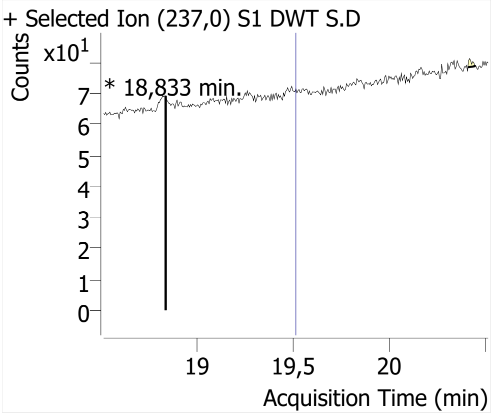

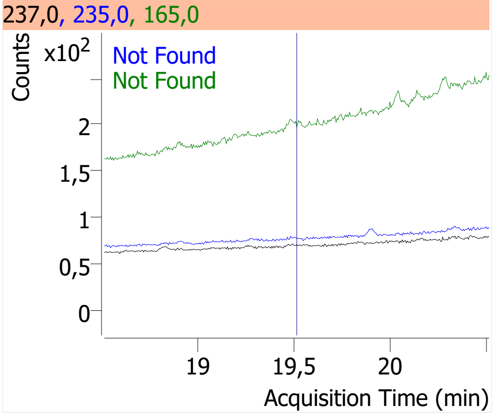

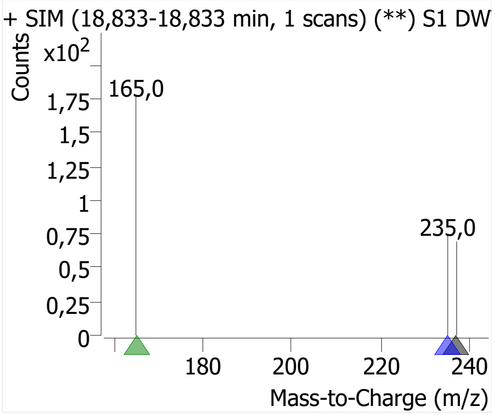

Supplement: S2 File — (PDF) [file pone.0343871.s002.pdf]
